# New *Lepidium* (Brassicaceae) from New Zealand

**DOI:** 10.3897/phytokeys.24.4375

**Published:** 2013-06-17

**Authors:** P. J. de Lange, P. B. Heenan, G. J. Houliston, J. R. Rolfe, A. D. Mitchell

**Affiliations:** 1 Science & Capability Group, Terrestrial Ecosystems, Department of Conservation, Private Bag 68908 Newton, Auckland 1145, New Zealand; 2 Allan Herbarium, Landcare Research, P.O. 69, Lincoln 7640, Canterbury, New Zealand; 3 Ecological Genetics Group, Landcare Research, P.O. 69, Lincoln 7640, Canterbury, New Zealand; 4 Science & Capability Group, Terrestrial Ecosystems, National Office, Department of Conservation, P.O. Box 10420, Wellington 6143, New Zealand; 5 Otago School of Medicine, University of Otago, Christchurch, PO Box 4345, Christchurch 8140, New Zealand

**Keywords:** New Zealand Archipelago, Kermadec Islands, Brassicaceae, *Lepidium*, *L. oleraceum* group, new species, *L. aegrum* sp. nov., *L. amissum* sp. nov., *L. castellanum* sp. nov., *L. crassum* sp. nov., *L. juvencum* sp. nov., *L. limenophylax* sp. nov., *L. oblitum* sp. nov., *L. oligodontum* sp. nov., *L. panniforme* sp. nov., *L. rekohuense* sp. nov., *L. seditiosum* sp. nov., typifications, ecology, conservation

## Abstract

A revision of the New Zealand endemic *Lepidium oleraceum* and allied species is presented. Sixteen species are recognised, 10 of these are new. The new species are segregated on the basis of morphological characters supported by molecular data obtained from three DNA markers (two rDNA and one cpDNA). One species, *Lepidium castellanum* **sp. nov.**, is endemic to the Kermadec Islands where it is sympatric with *Lepidium oleraceum*. The North Island of New Zealand supports four species, with two of them, *Lepidium amissum* **sp. nov.** and *Lepidium obtusatum*, now extinct. The South Island supports six species, that, aside from *Lepidium banksii*, *Lepidium flexicaule* and *Lepidium oleraceum*, are all confined to the south-eastern half of the island (*Lepidium aegrum* **sp. nov.**, *Lepidium crassum* **sp. nov.** and *Lepidium juvencum* **sp. nov.**). One of these, *Lepidium juvencum* **sp. nov.**, extends to Stewart Island. The Chatham Islands support six species (*Lepidium flexicaule*, *Lepidium oblitum* **sp. nov.**, *Lepidium oleraceum*, *Lepidium oligodontum* **sp. nov.**, *Lepidium panniforme* **sp. nov.**, and *Lepidium rekohuense* **sp. nov.**), one of which, *Lepidium oligodontum* **sp. nov.**, extends to the Antipodes Islands group. The remote, subantarctic Bounty Islands group supports one endemic, *Lepidium seditiosum* **sp. nov.**, which is the only vascular plant to be recorded from there. *Lepidium limenophylax* **sp. nov.** is known from islands off the south-western side of Stewart Island/Rakiura, The Snares and Auckland islands. *Lepidium naufragorum*, although not related to *Lepidium oleraceum* and its allies, is also treated because populations with entire leaves are now known. Typification is undertaken for *Lepidium banksii*, *Lepidium oleraceum*, *Lepidium oleraceum* var. *acutidentatum*, var. *frondosum* and var. *serrulatum*.

## Introduction

As currently circumscribed, the New Zealand members of the cosmopolitan genus *Lepidium* comprise 10 indigenous species (Garnock-Jones 1988; Garnock-Jones and Norton 1995; Heenan et al. 2007). Recently, the New Zealand species have been the subject of ongoing research that has resulted in new circumscriptions of the inland species *Lepidium sisymbrioides* Hook.f. and *Lepidium solandri* Kirk (Heenan et al. 2007), as well as the publication of phylogenetic (Mitchell and Heenan 2000; Mummenhoff et al. 2004) and evolutionary studies (Dierschke et al. 2009). There are an additional 16 species naturalised in New Zealand (Garnock-Jones 1988; Webb et al. 1995; Howell and Sawyer 2006; Heenan and de Lange 2011), including species that have previously been assigned to *Coronopus* Zinn and *Cardaria* Desv. (Al-Shehbaz et al. 2002).

**Figure 1.**
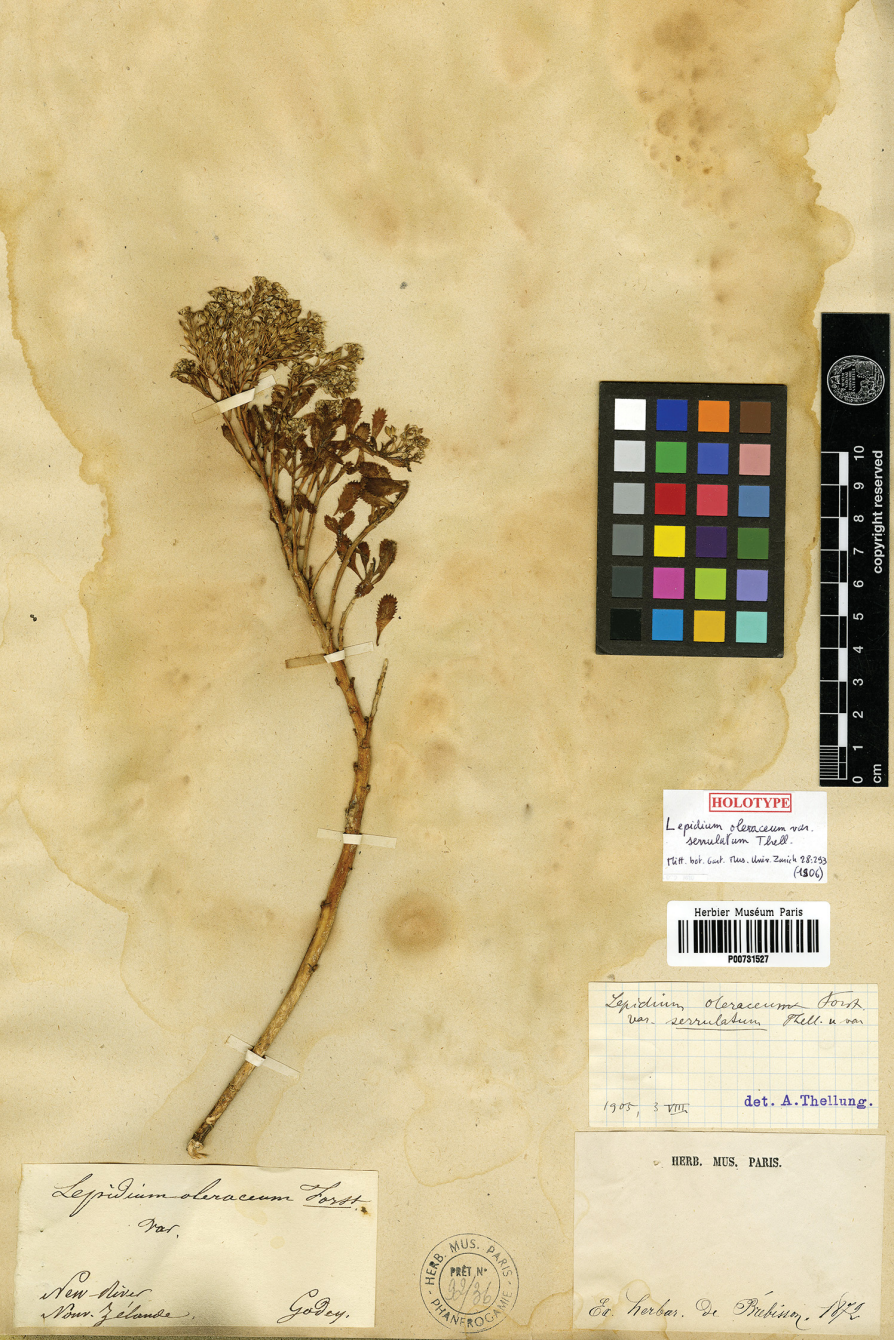
Holotype of *Lepidium oleraceum* var. *serrulatum* Thell.

It has long been recognised that *Lepidium oleraceum* G.Forst ex Sparrm. is a widely distributed and rather variable coastal species (Kirk 1899; Cheeseman 1906, 1925; Allan 1961; Garnock-Jones 1988; Garnock-Jones and Norton 1995; de Lange et al. 2010a). As currently circumscribed, *Lepidium oleraceum* *sensu lato* (s.l.) has a range encompassing the subtropical Kermadec Islands, the main islands of New Zealand (North, South and Stewart islands), Chatham Islands, and the subantarctic Antipodes, Snares and Auckland islands. Although the most recent treatment by Allan (1961) accepted one variable species, three additional named varieties of *Lepidium oleraceum* were discussed: var. *acutidentatum* Kirk, var. *frondosum* Kirk, and var. *serrulatum* Thell. As Allan (1961) noted, the taxonomic status of these varieties has never been particularly clear, though it would seem that the large, bushy, broad-leaved form occurring on the Three Kings Islands and the northern part of North Island is referable to Kirk’s var. *frondosum*, while plants with coarsely serrate and lanceolate leaves comprise his var. *acutidentatum*. *Lepidium oleraceum* var. *serrulatum* is known only from the type at the Paris Herbarium (P!, Fig. 1), described by Thellung (1906) from a single collection made from ‘New River, New Zealand’ a place equated by Allan (1961) with the Riverton Estuary, Southland. The type is a plant with narrow and deeply serrated leaves; comparable forms have been occasionally gathered from the North and South islands.

Various authors have suggested that *Lepidium oleraceum* s.l. requires further taxonomic study to resolve the variation that had been observed throughout its distributional range. In reference to the three varieties, Allan (1961, p. 177) concluded that they, along with *Lepidium oleraceum*, “need much further study”. Later, Given (1981) observed that such a study may now be impossible as *Lepidium oleraceum* was in his view close to extinction and, because of this, many critical populations had by then been lost. In their paper describing *Lepidium naufragorum* Garn.-Jones et D.A.Norton, Garnock-Jones and Norton (1995) discussed the variation of *Lepidium oleraceum*, of which they felt that ‘forms matching named varieties can be recognised, [though] there is considerable variation (possibly clinal) present within *Lepidium oleraceum*. A critical investigation of the whole complex, based on genetic as well as morphological analyses, is required before any taxonomic changes within this species are formalised’.

In this paper we present a taxonomic revision of *Lepidium oleraceum* s.l. (hereafter referred to as the *Lepidium oleraceum* group), along with the allied species *Lepidium banksii* Kirk, *Lepidium flexicaule* Kirk, and the extinct *Lepidium obtusatum* Kirk. This revision began in 1990 (de Lange 2010) and has involved the critical study of type specimens and herbarium material, extensive field work to examine natural variation of wild populations throughout their geographic range, supplemented with the use of DNA sequence data and, where possible, cultivating plants under uniform conditions. Although the current natural distribution of *Lepidium oleraceum* group and its allies comprises small, highly fragmented populations that most probably represent only a small remnant of the original distribution, abundance, and morphological and genetic diversity of the species, sufficient populations remain to enable the taxonomic revision offered here.

In addition to revising the *Lepidium oleraceum* group, we also treat *Lepidium naufragorum*. At the time of its formal naming *Lepidium naufragorum* was regarded as having a relationship to *Lepidium flexicaule* and the *Lepidium oleraceum* group (Garnock-Jones and Norton 1995). It is now known to have no close relationship to these species (Mitchell and Heenan 2000; Heenan et al. 2007). Nevertheless we treat it here because of the recent discovery of some populations that have entire rather than pinnatifid rosette leaves, so necessitating a slight revision of the description provided by Garnock-Jones and Norton (1995).

## Materials and methods

### Plant collections and specimens

The taxonomic revision presented in this paper is based on the critical study of specimens and type material of *Lepidium banksii*, *Lepidium flexicaule*, *Lepidium obtusatum*, *Lepidium oleraceum* group, and *Lepidium naufragorum* held at the following herbaria AK, BM, CANU, CHR, HO, K, MEL, MPN, NSW, OTA, P, UNITEC, WAIK, WELT, and WELTU. Herbarium acronyms follow Thiers (2012). Live specimens of the northern South Island endemic *Lepidium banksii* were studied in the field at Waimea Estuary (41°15'49.96"S, 173°6'5.09"E), Totaranui (40°48'53.32"S, 173°00'53.91"E) and Separation Point (40°46'57"S, 172°59'54.30"E). Plants of the indigenous *Lepidium flexicaule* were examined in the wild in the North Island at Stent Road (39°9'46.72"S, 173°50'15.89"E), in the north-western South Island at Scott’s Beach (41°5'27.95"S, 172°6'14.17"E), Punakaiki (42°5'19.95"S, 171°20'20.30"E) and, on Chatham Island (hereafter referred to as Rekohu) at Point Somes (43°50'18.73"S, 176°52'53.44"W). Plants of *Lepidium oleraceum* group were examined in the field on two islets of the Herald Islets (29°14'43.8"S, 177°51'25.9"W) in the northern group of the Kermadec Islands, and on Cheeseman Island (30°32'16.8"S, 178°34'1"W) and L’Esperance Rock (30°26'1"S, 178°54'W) in the southern Kermadec Islands group; on the Three Kings Islands (34°10'5.63"S, 172°5'40.94"E), Poor Knights Islands (35°28'35.98"S, 174°44'22.27"E), Mokohinau Islands (35°54'23.93"S, 175°7'47.57"E), Ngatutura Point (Waikato) (37°31'5.31"S, 174°44'38.20"E), an unnamed islet near Banks Peninsula (43°53'46.26"S, 172°52'2.54"E), coastal Otago and Otago Peninsula (45°52'47.00"S, 170°37'56.97"E), islands off the coast of Stewart Island (46°51'38.92"S, 168°13'45.79"E), and throughout the Chatham Island group (43°55'58.38"S, 176°29'18.99"W). Additional observations, live plants and specimens of *Lepidium oleraceum* group were also provided by J.W. Barkla and T.C. Greene from Macauley Island, Haszard Islet and Cheeseman Island in the Southern Kermadec Islands group; E.K. Cameron from West Island (part of the Three Kings Islands); by P. Sagar from North East Island, Snares Island group (47°59'49.23"S, 166°33'35.49"E); and J.W. Barkla and G. Loh from various sites around Otago. The late P.I. Knightbridge provided fresh material of *Lepidium naufragorum* from Taumaka, Open Bay Islands (47°59'49.23"S, 166°33'35.49"E), and S. Walls provided live plants of *Lepidium banksii* from Totaranui headland (40°48'53.32"S, 173°00'53.91"E) and Moutere Island (41°10'14.18"S, 173°2'11.28"E).

To this sampling were added further plants of *Lepidium banksii*, *Lepidium flexicaule*, *Lepidium oleraceum* group and *Lepidium naufragorum* collected by New Zealand botanists from a wide range of sites, and held by the Auckland Botanic Gardens, University of Auckland, Oratia Native Plant Nurseries, Motukarara Conservation Nursery and Landcare Research (Lincoln campus) to meet the *ex-situ* objectives of the Department of Conservation Coastal Cress Recovery Plan (Norton and de Lange 1999).

### Molecular phylogenetics

In conjunction with the taxonomic revision of the *Lepidium oleraceum* group and allied species, phylogenetic studies were carried out to determine relationships among the taxa, as well as to gain a better understanding of the population variation in extant populations of *Lepidium* species. For simplicity and clarity we use the new taxonomic names described here throughout the manuscript.

All taxa accepted in the revision presented here were included in the phylogenetic analyses as well as other closely related New Zealand and Australian taxa (Table 1) (Mummenhoff et al. 2004; Heenan et al. 2007). DNA sequence data (nrDNA (ITS1-5.8S-ITS2 and ETS), cpDNA (*trn*L-F spacer regions)) was obtained from 65 representatives of the *Lepidium oleraceum* group and seven from the *Lepidium sisymbrioides* group, which were used as outgroups (Mummenhoff et al. 2004). Not all DNA regions were amplified for each sample because it was difficult to access material. Only one species, the endemic and extinct *Lepidium amissum* de Lange et Heenan, described in this paper, was not included because three attempts failed to amplify DNA from the limited material available (three herbarium sheets > 100 years old). The phylogenetic relationship of both the *Lepidium oleraceum* and *Lepidium sisymbrioides* groups, and so the validity of using taxa from the *Lepidium sisymbrioides* as outgroups,had already been established by Mummenhoff et al. (2004). Our sampling of the *Lepidium oleraceum* groupwas intended to encompass the entire known range and extant variation within the species.

**Table 1.** Herbarium vouchers and GenBank numbers of *Lepidium* study group and outgroup samples used in the present investigation.

**Taxon**	**Nr.**	**Locality**	**ETS**	**ITS**	**trnL**	**Voucher or reference**
*Lepidium aegrum* Heenan & de Lange		New Zealand, South I., Canterbury, Banks Peninsula	KC109387	KC109330	—	AK 283510
*Lepidium aschersonii* Thell.		Australia	—	AJ582426, AJ582483	AY015838, AY015926	Mummenhoff et al. (2004)
*Lepidium banksii* Kirk	1	New Zealand, South I., Nelson, Abel Tasman National Park, Totaranui Point	KC109388	KC109331	KC109368	AK 259119
*Lepidium banksii* Kirk	2	New Zealand, South I., Nelson, Abel Tasman National Park, Totaranui	KC109389	—	KC109369	CHR 515311
*Lepidium banksii* Kirk	3	New Zealand, South I., Nelson, Nguroa I.	KC109390	KC109332	—	AK 259118
*Lepidium castellanum* de Lange & Heenan		Kermadec Is., Macauley I.	KC109391	—	—	AK 306194
*Lepidium crassum* Heenan & de Lange	1	New Zealand, South I., Otago, Bridge Point,	KC109394	—	—	CHR 609797
*Lepidium crassum* Heenan & de Lange	2	New Zealand, South I., Otago Peninsula, Aramoana	KC109395	KC234312	KC109370	AK 234312
*Lepidium crassum* Heenan & de Lange	3	New Zealand, South I., Otago Peninsula, Aramoana	KC109396	—	—	CHR 609772
*Lepidium crassum* Heenan & de Lange	4	New Zealand, South I., Otago Peninsula, Wharekakahu I.	KC109393	—	—	CHR 609801
*Lepidium crassum* Heenan & de Lange	5	New Zealand, South I., Otago Peninsula, Eye talus	KC109392	—	—	no voucher
*Lepidium crassum* Heenan & de Lange	6	New Zealand, South I., Otago, Tairei	KC109397	—	—	no voucher
*Lepidium crassum* Heenan & de Lange	7	New Zealand, South I., Otago, Nugget Point	KC109398	—	—	CHR 609805
*Lepidium desvauxii* Thell.	1	Chatham Is., Rekohu (Chatham I.), Waitangi West	KC109399	KC109334	KC109371	AK 294948
*Lepidium desvauxii* Thell.	2	Australia	—	AJ582429, AJ582486	AY015848, AY015935	Mummenhoff et al. (2004)
*Lepidium fasciculatum* Thell.		Australia	—	AJ582428, AJ582485	AJ582563, AJ582564	Mummenhoff et al. (2004)
*Lepidium flexicaule* Kirk	1	New Zealand, South I., Karamea, Scott’s Beach	—	AJ582430, AJ582487	AY015853	Mummenhoff et al. (2004)
*Lepidium flexicaule* Kirk	2	New Zealand, South I., Karamea, Scott’s Beach	KC109403	—	—	—
*Lepidium flexicaule* Kirk	3	Chatham Is., Rekohu (Chatham I.) Point Somes	KC109400	KC109335	KC109372	AK 289897
*Lepidium flexicaule* Kirk	4	Australia, Tasmania, Port Davy, Elliot Point	KC109401	KC109336	KC109373	HO 402077
*Lepidium flexicaule* Kirk	5	Australia, Tasmania, Port Davy, Gull Reef	KC109402	KC109337	KC109374	HO 26460
*Lepidium foliosum* Desv.	1	Australia, Tasmania, Furneaux, Little Dog Island	KC109404	KC109338	KC109375	HO 525314
*Lepidium foliosum* Desv.	2	Australia, Tasmania, Marion Bay, Vischer Island,	KC109405	KC109339	—	HO 306271
*Lepidium hyssopifolium* Desv.		Australia	—	AJ582435, AJ582492	AY015861, AY015970	Mummenhoff et al. (2004)
*Lepidium juvencum* Heenan & de Lange	1	New Zealand, South I., Otago Peninsula, Long Beach	KC109406	KC109340	—	CHR 609803
*Lepidium juvencum* Heenan & de Lange	2	New Zealand, South I., Otago Peninsula, Green Island	KC109407	—	—	no voucher
*Lepidium juvencum* Heenan & de Lange	3	New Zealand, South I., Otago Peninsula, Green Island	KC109408	—	—	AK 238646
*Lepidium limenophylax* de Lange, B.D.Rance & D.A.Norton		Snares Is, North-East I.	KC109409	KC109341	KC109376	AK 283482
*Lepidium muelleri-ferdinandi* Thell.		Australia	—	AJ582427, AJ582484	AY015870, AY015956	Mummenhoff et al. (2004)
*Lepidium naufragorum* Garn.-Jones & D.A.Norton	1	New Zealand, South I., West Coast, Open Bay Is., Taumaka	AJ532422	DQ989386	AY015958	Heenan et al. (2007)
*Lepidium naufragorum* Garn.-Jones & D.A.Norton	2	New Zealand, South I., West Coast, Open Bay Is., Taumaka	KC109410	—	KC109377	AK 317068
*Lepidium naufragorum* Garn.-Jones & D.A.Norton	3	New Zealand, South I., West Coast, Open Bay Is., Taumaka	KC109411	—	KC109378	AK 317070
*Lepidium naufragorum* Garn.-Jones & D.A.Norton	4	New Zealand, South I., West Coast, Open Bay Is., Taumaka	—	—	AY015872, AY015958	Mummenhoff et al. (2004)
*Lepidium nesophilum* Hewson	1	Lord Howe I., Gower Track	KC253769	KC109342	—	NSW 253769
*Lepidium nesophilum* Hewson	2	Lord Howe I., Little Slope	KC109413	KC109343	KC109379	NSW 492466
*Lepidium oblitum* Houliston, Heenan & de Lange		Chatham Is., Mangere I.	KC109415	—	—	AK 300342
*Lepidium obtusatum* Kirk		New Zealand, North I., Wellington, Miramar Peninsula, Seatoun	KC109414	KC109344	KC109380	CHR 329224
*Lepidium oleraceum* G.Forst. ex Sparrm.	1	New Zealand, Three Kings Is., West I.	KC109435	KC109360	—	AK 288471
*Lepidium oleraceum* G.Forst. ex Sparrm.	2	New Zealand, North I., North Auckland, Te Paki, Motuopao I.	KC109428	KC109354	—	AK 195840
*Lepidium oleraceum* G.Forst. ex Sparrm.	3	New Zealand, North I., North Auckland, Te Oneroa-a-Tohe (Ninety Mile Beach), Matapia I.	KC109424	KC109350	—	AK 212201
*Lepidium oleraceum* G.Forst. ex Sparrm.	4	New Zealand, North I., Poor Knights Is., Archway I.	KC109430	—	—	AK 302001
*Lepidium oleraceum* G.Forst. ex Sparrm.	5	New Zealand, North I., Mokohinau Is, Motuharakeke I.	KC109420	—	—	AK 226984
*Lepidium oleraceum* G.Forst. ex Sparrm.	6	New Zealand, North I., Mokohinau Is., Motukino (Fanal I.)	KC109427	KC109353	—	AK 258785
*Lepidium oleraceum* G.Forst. ex Sparrm.	7	New Zealand, North I., Great Barrier I. (Aotea I.), Mahuki I.	KC109421	KC109348	—	AK 255389
*Lepidium oleraceum* G.Forst. ex Sparrm.	8	New Zealand, North I., Coromandel Peninsula, Aldermen Is., Hongiora	KC109416	KC109345	—	AK 304818
*Lepidium oleraceum* G.Forst. ex Sparrm.	9	New Zealand, North I., Coromandel Peninsula, Aldermen Is., Middle Is,	KC109426	KC109352	—	AK 293250
*Lepidium oleraceum* G.Forst. ex Sparrm.	10	New Zealand, North I., Coromandel Peninsula, Matariki I.	KC109425	KC109351	—	AK 231114
*Lepidium oleraceum* G.Forst. ex Sparrm.	11	New Zealand, North I., South Auckland, Ngatutura Point, Shag Rock	KC109429	KC109355	—	AK 306120
*Lepidium oleraceum* G.Forst. ex Sparrm.	12	New Zealand, North I., Bay of Plenty, Karewa I.	KC109418	KC109347	—	AK 293306
*Lepidium oleraceum* G.Forst. ex Sparrm.	13	New Zealand, North I., Bay of Plenty, Karewa I.	KC109419	—	—	AK 299140
*Lepidium oleraceum* G.Forst. ex Sparrm.	14	New Zealand, North I., South Auckland, Albatross Point, Waioioi Reef	KC109436	KC109361	—	AK 297502
*Lepidium oleraceum* G.Forst. ex Sparrm.	15	New Zealand, North I., Taranaki, Sugarloaf Is., Motumahanga I.	KC109434	KC109359	—	AK 293304
*Lepidium oleraceum* G.Forst. ex Sparrm.	16	New Zealand, North I., Kapiti I.	KC109417	KC109346	—	AK 259125
*Lepidium oleraceum* G.Forst. ex Sparrm.	17	New Zealand, North I., Wellington, Mana I.	KC109423	KC109349	—	AK 293308
*Lepidium oleraceum* G.Forst. ex Sparrm.	18	New Zealand, South I., Marlborough, Cook Strait Takapourewa (Stephens I.)	KC109433	KC109358	KC109381	AK 233809
*Lepidium oleraceum* G.Forst. ex Sparrm.	19	New Zealand, South I., Marlborough, Cook Strait Brothers Is., South Brother	KC109431	KC109356	—	AK 293307
*Lepidium oleraceum* G.Forst. ex Sparrm.	20	New Zealand, South I., Marlborough, Cook Strait Brothers Is., North Brother	KC109432	KC109357	—	no voucher
*Lepidium oleraceum* G.Forst. ex Sparrm.	21	Chatham Is., Mangere I.	KC109422	—	—	AK 295979
*Lepidium oligodontum* de Lange & Heenan	1	Chatham Is., The Sisters (Rangitatahi)	KC109438	KC109363	KC109382	AK 290289
*Lepidium oligodontum* de Lange & Heenan	2	Chatham Is., Rekohu (Chatham I.), Point Somes	KC109437	KC109362	—	AK 292941
*Lepidium oligodontum* de Lange & Heenan	3	Antipodes Is., Antipodes I.	KC109439	—	KC109383	AK 293309
*Lepidium oxytrichum* Sprague		Australia	—	AJ582424, AJ582481	AY015877, AY015963	Mummenhoff et al. (2004)
*Lepidium panniforum* de Lange & Heenan		Chatham Is., Mangere I.,	KC109440	KC109364	KC109384	AK 293305
*Lepidium papillosum* F.Muell.		Australia	—	AJ582425, AJ582482	AY015878, AY015964	Mummenhoff et al. (2004)
*Lepidium pseudohyssopifolium* Hewson		Australia	—	AJ582431, AJ582488	—	Mummenhoff et al. (2004)
*Lepidium pseudopapillosum* Thell.		Australia	—	AJ582423, AJ582480	AY015886, AY015971	Mummenhoff et al. (2004)
*Lepidium pseudotasmanicum* Thell.		Australia	—	AJ582432, AJ582489	AY015887, AY015972	Mummenhoff et al. (2004)
*Lepidium rekohuense* de Lange & Heenan	1	Chatham Is., Rekohu (Chatham I.), Kaiangaroa	KC109441	KC109365	—	AK 259130
*Lepidium rekohuense* de Lange & Heenan	2	Chatham Is., Forty Fours (Motuhara), Chatham Islands	KC109442	KC109366	KC109385	AK 290290
*Lepidium seditiosum* de Lange Heenan, & J.Rolfe		Bounty Is., Funnel I.	KC109443	KC109367	—	OTA 59718
**Outgroups**						
*Lepidium sisymbrioides* Hook.f.	1	New Zealand, South I., Otago, Gards Road	DQ989378	DQ997560	DQ997064	Heenan et al. (2007)
*Lepidium sisymbrioides* Hook.f.	2	New Zealand, South I., Otago, Otematata	DQ989376	DQ997565	DQ997054	Heenan et al. (2007)
*Lepidium sisymbrioides* Hook.f.	3	New Zealand, South I., Otago, Falls Dam	DQ989381	DQ997569	DQ997061	Heenan et al. (2007)
*Lepidium sisymbrioides* Hook.f.	4	New Zealand, South I., Otago, Nevis Bluff	DQ989383	DQ997561	DQ997067	Heenan et al. (2007)
*Lepidium solandri* Kirk		New Zealand, South I., Canterbury, Castle Hill	DQ989389	DQ997553	DQ997060	Heenan et al. (2007)
*Lepidium tenuicaule* Kirk	1	New Zealand, South I., Otago, Shag Point	DQ989404	AJ582421	AY015899	Heenan et al. (2007)
*Lepidium tenuicaule* Kirk	2	New Zealand, South I., Otago, Shag Point	KC109444	—	KC109386	AK 232774

Total genomic DNA was extracted from 0.1–1.0 g of leaf tissue using a QIAGEN DNeasy® Plant Mini Kit or an INTRON Plant DNA kit using fresh material, fresh material stored in Silica Gel, and from herbarium specimens. DNA sequencing of nrDNA (ITS1-5.8S-ITS2 and ETS), and plastid DNA (*trn*L-F spacer regions) were carried out following the methods of White et al. (1990) and Mitchell and Heenan (2000) respectively.

PCR products were purified using either the Perfectprep PCR cleanup kits (Eppendorf), or diluted to approximately 1:4 with ultrapure water. Each sample was sequenced in the sense and antisense direction by the Centre for Gene Technology (University of Auckland), the Allan Wilson Centre Genome Service, (Massey University, Albany), or the Landcare Research Ecological Genetics Laboratory (Tamaki). Sequencing reactions were performed with the same primers as the PCR amplifications and the 3.1 ABI Prism^TM^ Big Dye Terminator Sequencing Kit (Applied Biosystems, Scoresby, Vic.). Sequences obtained in this study have been assigned GenBank accession numbers (Table 1).

### Matrix preparation

Sequence alignment was performed using ClustalX vers. 1.81 (Thompson et al. 1997). Multiple alignment parameters were set to 12 for gap opening penalty and 6.0 for gap extension penalty. No further alignment was required.

Maximum likelihood (ML) analyses were conducted using RaXML (Stamatakis 2006) employing the full search algorithm with Gamma Distribution and General time reversible (GTR) model. In each of the datasets support for nodes was assessed by non-parametric bootstrapping (Felsenstein 1985) using 1000 replicates. Because of the simple nature of the dataset, no further phylogenetic reconstruction was attempted. Bayesian reconstruction in Mr Bayes (Huelsenbeck and Ronquist 2001), and parsimony analysis (Swofford 2006) in Paup* were undertaken, but preliminary analyses yielded similar results (not shown).

Trees were drawn in Figtree version 1.3.1. (Rambault 2008) and labels follow those in Table 1.

## Results

### DNA sequence data

**Figure 2.**
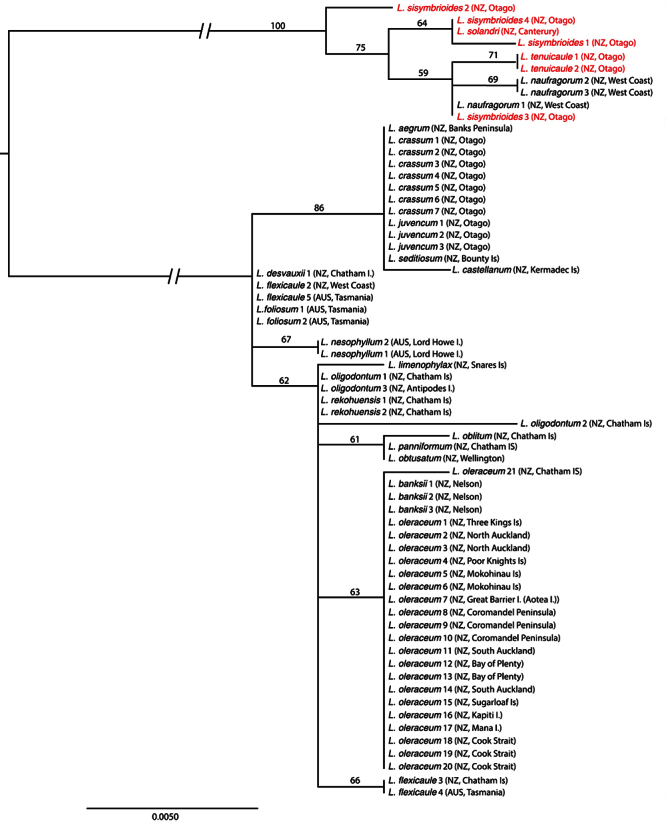
Maximum likelihood phylogeny of ETS as calculated in RaXML, showing the *Lepidium oleraceum* (black text) and *Lepidium sisymbrioides* (red text) groups, including the newly described taxa. Bootstrap support from 1000 replicates is shown for branches where it is greater than 50. Codes for each individual in the phylogeny are shown in Table 1. The main clade branches have been truncated to accommodate the tree within the journal format. AUS = Australia, NZ = New Zealand.

**Figure 3.**
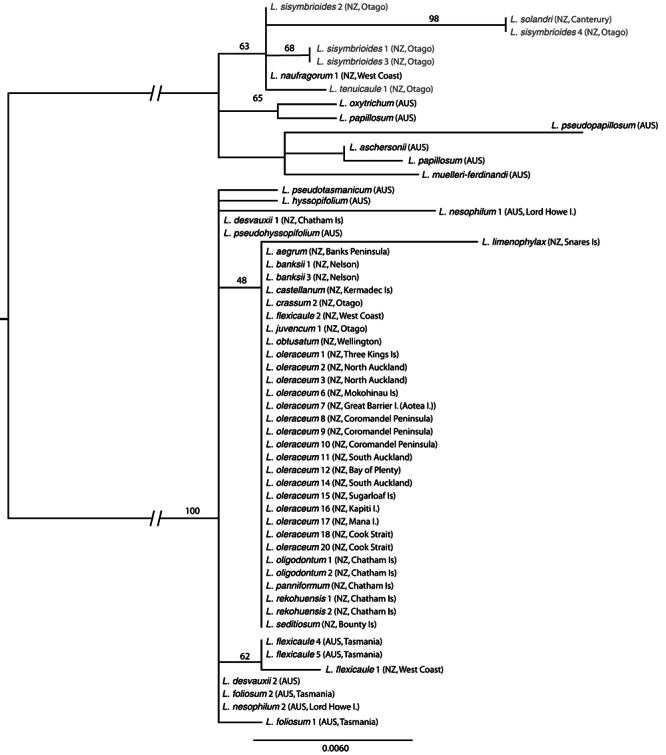
Maximum likelihood phylogeny of ITS as calculated in RaXML, showing the *Lepidium oleraceum* (black text) and *Lepidium sisymbrioides* (red text) groups, including the newly described taxa. Bootstrap support from 1000 replicates is shown for branches where it is greater than 50. Codes for each individual in the phylogeny are shown in Table 1. The main clade branches have been truncated to accommodate the tree within the journal format.

**Figure 4.**
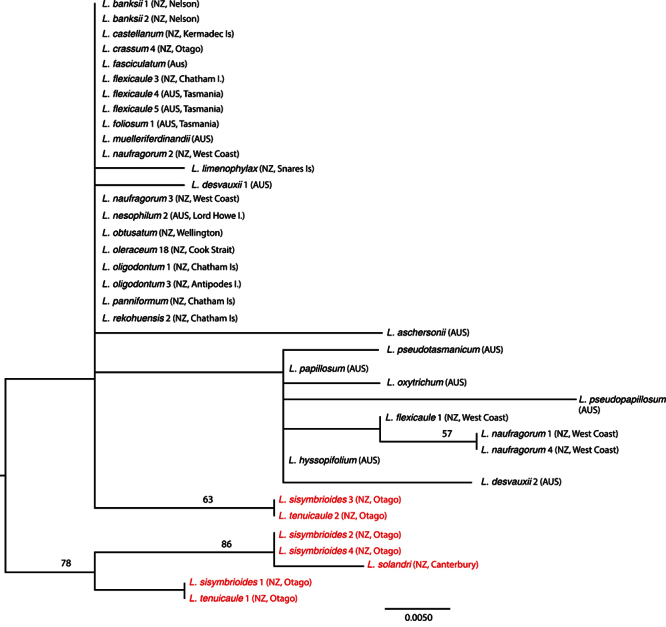
Maximum likelihood phylogeny of *trn*L-F as calculated in RaXML, showing the *Lepidium oleraceum* (black text) and *Lepidium sisymbrioides* (red text) groups, including the newly described taxa. Bootstrap support from 1000 replicates is shown for branches where it is greater than 50. Codes for each individual in the phylogeny are shown in Table 1.

Phylogenetic analyses provided some resolution of relationships, with ETS the most informative (Fig. 2), resolving the *Lepidium oleraceum* and *Lepidium sisymbrioides* groups. Within the *Lepidium oleraceum* group there is a strong north-south dichotomy, with another group present on the Chatham Islands and sub-Antarctic islands. The samples from the North Island comprised only *Lepidium oleraceum* *sensu stricto* (*s.s*.),and the southern group included *Lepidium aegrum* Heenan et de Lange from Banks Peninsula, *Lepidium crassum* Heenan et de Lange from the Otago coast, *Lepidium juvencum* Heenan et de Lange from Green Island and Long Beach Otago, *Lepidium limenophylax* de Lange, B.D.Rance et D.A.Norton from the Snares Islands, and the Kermadec Islands’ *Lepidium castellanum* de Lange et Heenan. Chatham Island and sub-Antarctic species included *Lepidium rekohuense* de Lange et Heenan, *Lepidium oblitum* Houliston, Heenan et de Lange, *Lepidium panniforme* de Lange et Heenan, and *Lepidium oligodontum* de Lange et Heenan from the Chatham Islandsand *Lepidium seditiosum* de Lange, Heenan et J.Rolfe from Bounty Island. ITS also resolved the *Lepidium oleraceum* and *Lepidium sisymbrioides* groups, but within ITS, there was no resolution within the *Lepidium oleraceum* groupincluding *Lepidium banksii* (Fig. 3). The pattern for *trn*L-F was more complex (Fig. 4), and is discussed below, although it again provided no resolution within the *Lepidium oleraceum* groupand *Lepidium banksii*.

### ETS Sequence Characteristics and Phylogeny

Eighteen unique ETS sequences were present among the 65 *Lepidium* samples that were amplified for this region (58 from the *Lepidium oleraceum* group and seven from the *Lepidium sisymbrioides* group) (Fig. 2). Within the *Lepidium sisymbrioides* group, the four samples of *Lepidium sisymbrioides* all had different ETS ribotypes, and *Lepidium naufragorum*, and *Lepidium tenuicaule* Kirk also contained more than one ribotype. Within the *Lepidium oleraceum* group, one described species, *Lepidium flexicaule*, was found to contain more than one ETS ribotype, with the New Zealand and Tasmanian examples of this species differing by two base substitutions.

Two pairs of species (*Lepidium rekohuense* and *Lepidium seditiosum*; *Lepidium oleraceum* and *Lepidium banksii*) shared the same ETS type (Fig. 2). *Lepidium flexicaule* (Tasmanian sample), *Lepidium foliosum* Desv.(Australian endemic) and *Lepidium desvauxii* Thell. (Australia and New Zealand samples) also share identical ETS sequences, however New Zealand *Lepidium flexicaule* have a different ETS type (Fig. 2). The three most southern / central South Island species, *Lepidium crassum*, *Lepidium aegrum* and *Lepidium juvencum*, share an identical ETS type with *Lepidium seditiosum* from the Bounty Islands, while *Lepidium castellanum* from Macauley Island (Kermadec Islands group) is also similar, differing by only one base pair (Fig. 2).

There were three different ribotypes in *Lepidium oleraceum* s.s., with a single sample from Mangere Island differing by a single base pair to the other *Lepidium oleraceum* s.s. samples.

The extinct *Lepidium obtusatum* and morphologically rather different Chatham Islands endemic *Lepidium panniforme* also share an ETS sequence type, which is almost identical to that of another Chatham Islands endemic, *Lepidium oblitum* (Fig. 2). Notably, these three species also differ from the other Chatham Islands endemic, *Lepidium rekohuense*,and the Chatham /Antipodes islands endemic, *Lepidium oligodontum* (Fig. 2). *Lepidium limenophylax*, known from the southern Titi Island of the south-western coastline of Stewart Island, the Snares and Auckland Islands, is sister to these Chatham / Antipodes islands species (i.e., *Lepidium oblitum*, *Lepidium oligodontum*, *Lepidium panniforme*, and *Lepidium rekohuense*) (Fig. 2).

This study also confirms the taxonomic status of the Lord Howe Island endemic, *Lepidium nesophilum* Hewson, which is a species that had previously been treated as *Lepidium oleraceum* (see Green 1990).

### ITS sequence characteristics and phylogeny

The 56 samples of *Lepidium* from which ITS data were collated (48 *Lepidium oleraceum* group, 6 *Lepidium sisymbrioides* group) are shown in Fig. 3. With the exception of *Lepidium limenophylax*, all of the other species within the *Lepidium oleraceum* group, including *Lepidium banksii* and the Chatham Island sample of *Lepidium flexicaule*, shared the same ITS sequence (Fig. 3).

The New Zealand (Karamea) sample of *Lepidium flexicaule* grouped with Australian samples from which it differed by a single base pair (Fig. 3). In addition, the two Lord Howe samples of *Lepidium nesophilum*, had markedly different ITS sequences, though, notably, they clustered with other Australian species and shared an identical ETS sequence.

### trnL-F sequence characteristics and phylogeny

The plastid data (*trn*L-F spacer region, 30 *Lepidium oleraceum* group, 7 *Lepidium sisymbrioides* group included) is interesting for the lack of strong divergence between the *oleraceum* and *sisymbrioides* groups, with two species (*Lepidium sisymbrioides* and *Lepidium naufragorum*) being found in both clades (Fig. 4); see Heenan et al. (2007) for discussion on sequence variation in *Lepidium naufragorum* and *Lepidium sisymbrioides*. This conflict could be explained by hybridisation between representatives of each of the two groups. With the exception of *Lepidium limenophylax* (which differed by one base pair), all of the species previously described in the *Lepidium oleraceum* group shared the same *trn*L-F sequence (Fig. 4).

Although the *trn*L-F spacer region showed only low sequence variation, several patterns were evident. For example, the morphologically distinctive species *Lepidium naufragorum* (New Zealand), *Lepidium flexicaule* (Australia + New Zealand), *Lepidium muelleri-ferdinandi* Thell. (Australia),* L. fasciculatum* Thell. (Australia) and *Lepidium oleraceum* (Kermadec Islands and New Zealand)had identical sequences (Fig. 4), a pattern that suggests a recent origin for these taxa. Further, the two New Zealand samples of *Lepidium flexicaule* (South Island (Karamea) and Chatham Islands) have distinct *trn*L-F sequences (Fig. 4). Morphologically, the Chatham Island race of *Lepidium flexicaule* differs from the majority of New Zealand (North Island and South Island) *Lepidium flexicaule* plants in that they lack the marginal leaf, pedicel and stem denticles that are diagnostic of the species on mainland New Zealand (see Garnock-Jones 1988). Interestingly, the Australian (Tasmanian) samples of *Lepidium flexicaule* had the same *trn*L-F sequence as the Chatham Islands, and they too lack denticles. However, our study has found that, while denticles are usual for New Zealand (North Island, South Island) examples of this species, not all plants from western South Island have them, and, as we have not sequenced examples of these “non-denticulate” plants, further study into this pattern is warranted. The pattern is reversed for the ITS data set, in which the South Island (Karamea) sample of *Lepidium flexicaule* clusters with Australian *Lepidium flexicaule* (Fig. 3).

### Phylogenetic implications

Bootstrap support for the trees was low to moderate, as would be expected for recently diverged taxa where resolution and divergence is low and where there has possibly been recent reticulation. An earlier study has indicated that hybridisation, and biocontinental origin for the New Zealand / Australian *Lepidium* is likely, although the age of this event and whether it is related to the divergence from the rest of the *Lepidium* group is unclear (Mummenhoff et al. 2004). Estimates for times of divergence vary between the *Lepidium oleraceum* and *Lepidium sisymbrioides* groups (0.7–1.3 Myr and 0.3–0.55 Myr respectively; Mummenhoff et al. 2004), although further hybridisation events and potential multiple dispersal events make accurate inference difficult. However, as noted by Mitchell and Heenan (2000), there was good support in the rDNA data for the *Lepidium oleraceum* and *Lepidium sisymbrioides* groups, though this was not found in the cpDNA dataset.

*Lepidium naufragorum* was found in the *Lepidium oleraceum* clade for the plastid data, but is a member of the *Lepidium sisymbrioides* group for both nuclear regions. This incongruence suggests that *Lepidium naufragorum* could have had a hybrid origin, with a parent from each of these groups (see in this respect also Mummenhoff et al. 2004). Although further study is required to address this issue, shared DNA sequences are common within New Zealand taxa. They have usually been interpreted as evidence of genera that have undergone a recent and rapid species radiation (e.g. Wagstaff et al. 1999 (for *Carmichaelia* R.Br.), Wagstaff et al. 2004 (for *Brachyglottis* J.R.Forst. et G.Forst.), Mitchell and Heenan 2002 (for *Sophora* L.), de Lange et al. 2008 (for *Crassula* L.), de Lange et al. 2010b (for *Kunzea* Rchb.), Smissen et al. 2011 (for *Simplicia* Kirk), Prebble et al. 2012 (for *Wahlenbergia* Schrad.)). This explanation is also most likely for the shared ETS ribotypes between *Lepidium rekohuense* and *Lepidium seditiosum*; and *Lepidium oleraceum* and *Lepidium banksii*, which also show this pattern.

In general, the *Lepidium oleraceum* and *Lepidium sisymbrioides* groups formed two distinct clades in both nuclear datasets, but was not strongly supported by the plastid data, as was found by Mummenhoff et al. (2004). Significantly for this study, the three DNA data sets confirm that those informally recognised morphological units within the *Lepidium oleraceum* group (see de Lange 2010; de Lange et al. 2010a) warrant formal taxonomic recognition. This is especially evident when, in our study, such morphologically discrete taxa as *Lepidium banksii* (Garnock-Jones 1988) proved indistinguishable from the very different *Lepidium oleraceum* s.s based on the rDNA and cpDNA sequences used here. That such a distinct species is not distinguished using this data also further supports the idea that much of the New Zealand Flora is in a state of rapid speciation (see above references), and that the reliance of DNA data sets, in isolation, to resolve taxonomic uncertainty is fraught with difficulty. In the case of the sequence data presented here. We conclude that the retention of a single morphologically divergent species, *Lepidium oleraceum* is unacceptable, and that formal taxonomic segregation within the group, at species level is warranted.

## Characters

### Growth habit

*Lepidium oleraceum* *s.s*., its allied species, and the unrelated *Lepidium naufragorum* are all perennial herbs that can be distinguished by their growth habit. This includes plants with an upright, spreading or sprawling, shrubby growth habit (*Lepidium castellanum*, *Lepidium aegrum*, *Lepidium banksii*, *Lepidium crassum*, *Lepidium juvencum*, *Lepidium oleraceum*, *Lepidium panniforme* and *Lepidium seditiosum*) and those with a prostrate or decumbent growth habit (*Lepidium amissum*, *Lepidium flexicaule*, *Lepidium limenophylax*, *Lepidium oblitum*, *Lepidium obtusatum*, *Lepidium oligodontum*, and *Lepidium rekohuense*). *Lepidium obtusatum* is, as discussed by Cockayne (1921), further distinguished from all other members of the *Lepidium oleraceum* group by its uniquely suckering habit. A further separation, based on seasonal growth patterns, is also evident, i.e., whether plants die back to the rootstock or not during winter. Of the extant species, *Lepidium castellanum*, *Lepidium aegrum*, *Lepidium juvencum*, and *Lepidium oleraceum* have persistent stems that do not die back to a rootstock; *Lepidium oblitum* and *Lepidium panniforme* stems are usually long-persistent, though sometimes they die back to a basal rootstock. The remaining extant species all have stems that die back to their rootstock. Herbarium evidence indicates that the extinct *Lepidium obtusatum* also died back in winter but there are insufficient herbarium specimens and no written accounts to enable us to determine whether the extinct segregate *Lepidium amissum* did so as well. Unique amongst the *Lepidium oleraceum* group, well-developed plants of *Lepidium limenophylax* may develop prominent “swellings” around branch nodes; these may also be a point of seasonal die back and vegetative growth and they sometimes produce adventitious roots.

### Leaf characters

**Figure 5.**
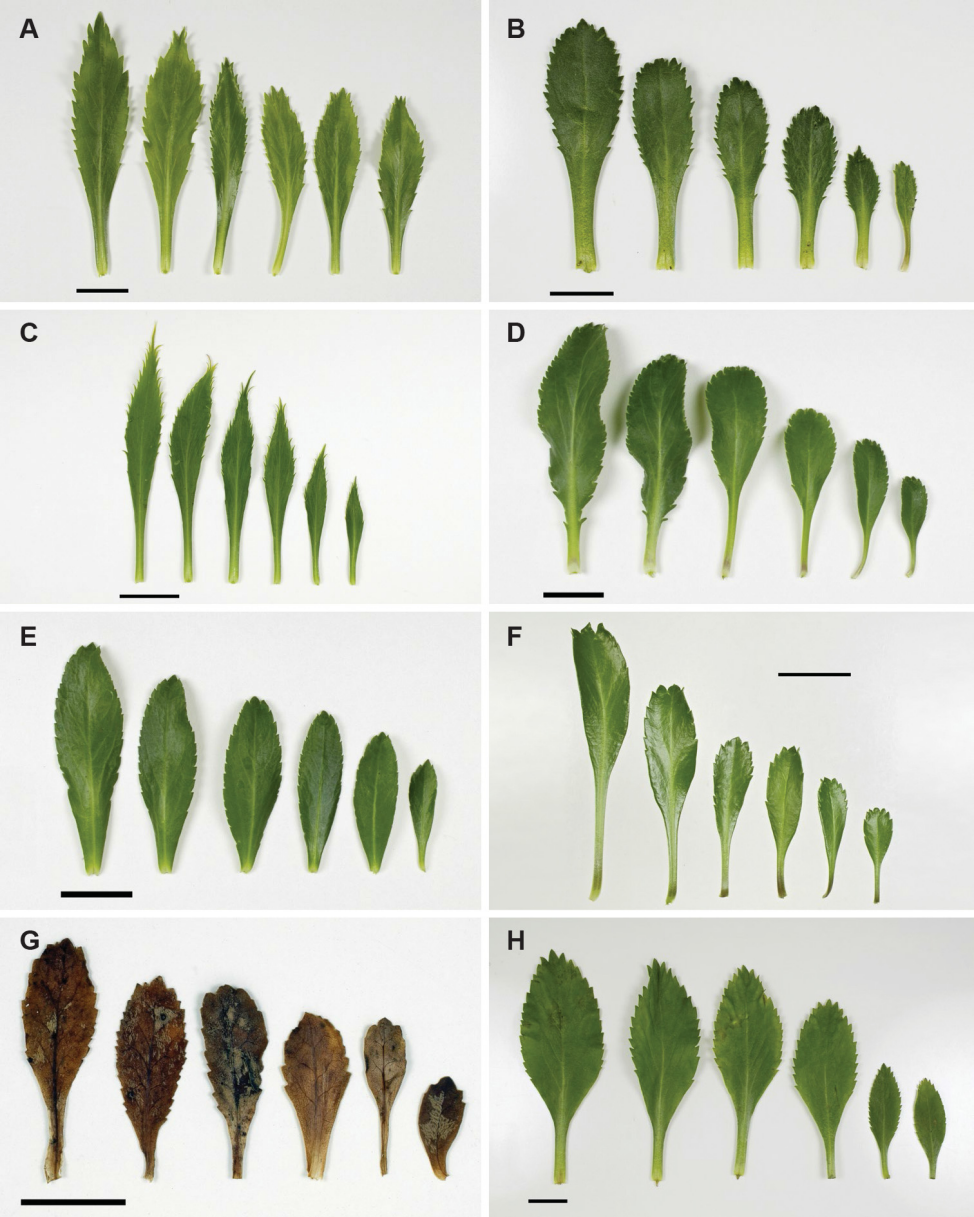
Cauline leaves of (A) *Lepidium aegrum*, (B) *Lepidium banksii*, (C) *Lepidium castellanum*, (D) *Lepidium crassum*, (E) *Lepidium juvencum*, (F) *Lepidium oblitum*, (G) *Lepidium obtusatum*, (H) *Lepidium oleraceum*. Scale bars = 20 mm.

**Figure 6.**
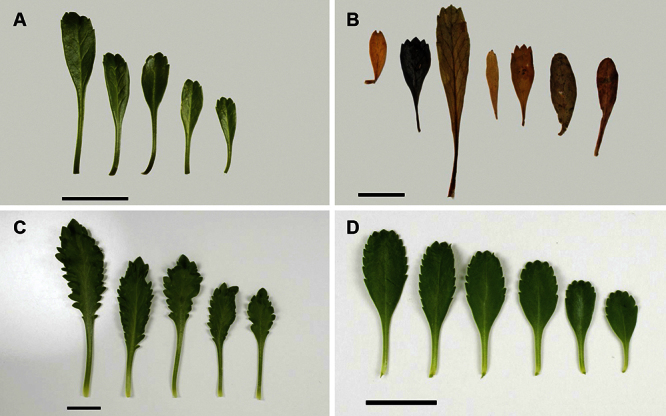
Cauline leaves of (A) *Lepidium oligodontum*—variation within a single plant, (B) *Lepidium oligodontum* - variation amongst plants from different locations: (left to right) The Sisters, Western Reef, Point Somes, Moriori Creek, Star Keys, Rangatira Island, Antipodes Island. (C) *Lepidium panniforme*, (D) *Lepidium rekohuense*. Scale bars = 20 mm.

The leaves of *Lepidium oleraceum* and allied species show a diversity of shape and degree of dentition that has been the basis for the recognition of a number of species and varieties (see Kirk 1899; Thellung 1906). Lamina-shape immediately distinguishes the pinnatifid *Lepidium flexicaule* from the other members of the *Lepidium oleraceum* group but not from the pinnatifid-leaved forms of the unrelated *Lepidium naufragorum*. Among the remaining members of the *Lepidium oleraceum* group, lamina size, shape, and degree of dentition provide important differences between the species (Figs 5, 6).

### Floral and fruit characters

The inflorescences, flowers and fruits of the *Lepidium oleraceum* group provide a number of characters enabling species recognition. The inflorescences of the species in the group are usually leaf-opposed. While in some species (*Lepidium flexicaule* and *Lepidium obtusatum*) the inflorescences tend to be obscured by foliage, they are held well above the surrounding foliage in the majority of species. In most species the inflorescence rachises and pedicels are glabrous, but in *Lepidium castellanum*, *Lepidium banksii*, *Lepidium rekohuense*, *Lepidium seditiosum*, (and very rarely *Lepidium oblitum*), minute, usually sparse, eglandular, clavate hairs are present. The sepals and petals show little variation with respect to size, shape, and degree of investiture. The size, shape and degree of notching of the silicles, and the length and shape of the stigma remnant are also critical characters (see Garnock-Jones 1988).

Stamen number of the *Lepidium oleraceum* group varies between species with two (*Lepidium flexicaule*, *Lepidium limenophylax* and *Lepidium rekohuense*), those with four (*Lepidium castellanum*, *Lepidium aegrum*, *Lepidium amissum*, *Lepidium juvencum*, *Lepidium banksii*, *Lepidium crassum*, *Lepidium juvencum*, *Lepidium naufragorum*, *Lepidium obtusatum*, *Lepidium oleraceum*, and *Lepidium seditiosum*), to those with variable stamen numbers (*Lepidium oblitum*, *Lepidium panniforme*, and *Lepidium oligodontum*). In these three species, stamen numbers may vary from two to four (*Lepidium oblitum*, *Lepidium panniforme*) to a maximum of six in *Lepidium oligodontum*, often within the same inflorescence, or on different individuals of the same species.

**Figure 7.**
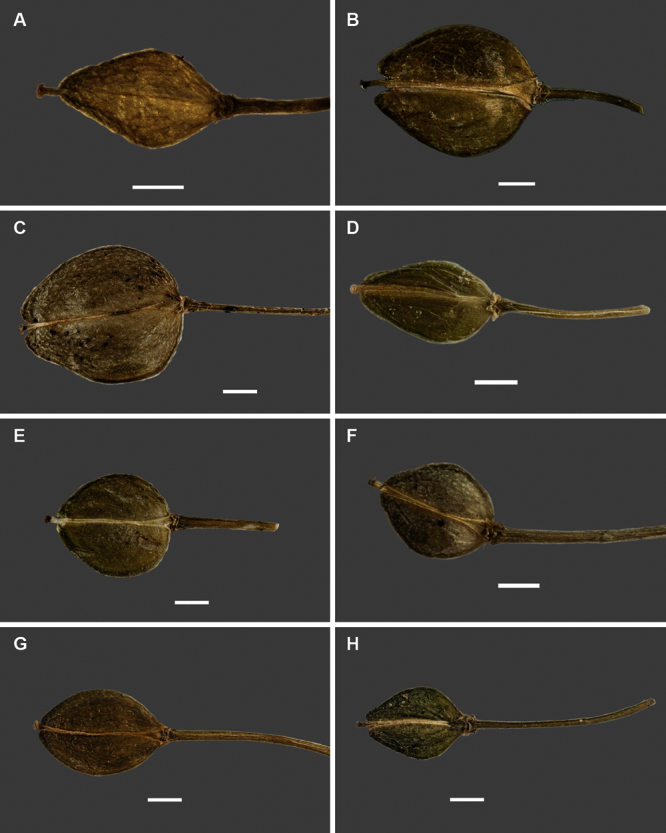
Silicles of (A) *Lepidium aegrum*, (B) *Lepidium amissum*, (C) *Lepidium banksii*, (D) *Lepidium castellanum*, (E) *Lepidium crassum*, (F) *Lepidium flexicaule*, (G) *Lepidium juvencum*, (H) *Lepidium limenophylax*. Scale bars = 1mm.

**Figure 8.**
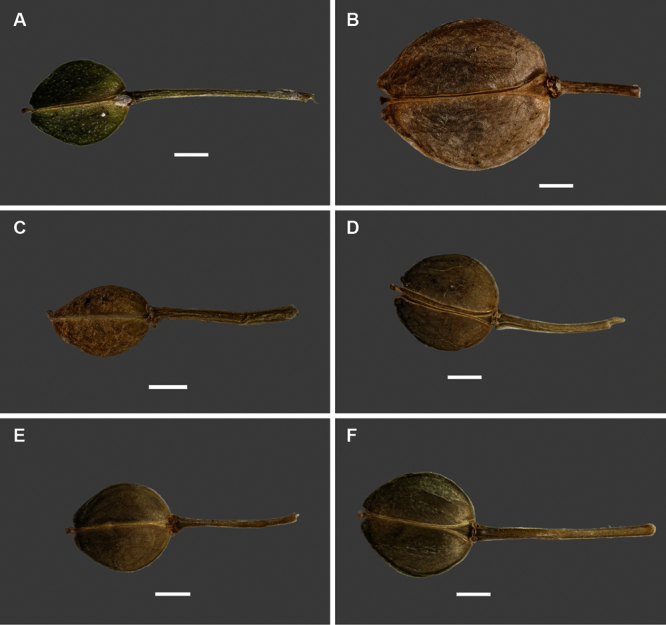
Silicles of (A) *Lepidium oblitum*, (B) *Lepidium obtusatum*, (C) *Lepidium oleraceum*, (D) *Lepidium oligodontum*, (E) *Lepidium panniforme*, (F) *Lepidium rekohuense*. Scale bars = 1 mm.

Silicle shape (Table 1, Figs 7, 8) varies and comprises four main shapes: 1. Silicles mostly elliptic to elliptic-rhomboid (rarely orbicular-rhomboid) (*Lepidium castellanum*, *Lepidium aegrum*, *Lepidium juvencum*, *Lepidium limenophylax*, *Lepidium naufragorum*, *Lepidium oleraceum*); 2. Silicles orbicular-rhomboid only (*Lepidium crassum*, *Lepidium juvencum*); 3. Silicles mostly ovate (ovoid), oval to obovate (*Lepidium amissum*, *Lepidium banksii*, *Lepidium oblitum*, *Lepidium obtusatum*, *Lepidium panniforme*); and, 4. Silicles mostly orbicular (rarely ovoid, obovate) (*Lepidium flexicaule*, *Lepidium oligodontum*, *Lepidium rekohuense*). The silicles of *Lepidium panniforme* can vary from ovate (the usual state) through to elliptic-rhomboid. With respect to silicle size, *Lepidium obtusatum* has the largest silicles (up to 6.4 × 4.9 mm) and both *Lepidium limenophylax* and *Lepidium panniforme* the smallest (up to 3.5 × 3.3 mm) (Table 1). Although silicle characters are unavailable for *Lepidium seditiosum*, of which we have only a single immature fruiting specimen, the few immature silicles present indicate that, as with all the other members of the *Lepidium oleraceum* group, the two valves are dehiscent (see *Lepidium* key).

The presence or absence of an apical notch to the silicle in the New Zealand species was an important character emphasised by Kirk (1899), Cheeseman (1906, 1925), Allan (1961), and Garnock-Jones (1988). In all these treatments the lack of a notch in *Lepidium oleraceum* is noted, and a silicle of that species showing this is clearly illustrated by Garnock-Jones (1988). This is a significant observation, because, of the new species segregated here from *Lepidium oleraceum* s.s. a shallow apical notch is present in eight species (though minute and so scarcely evident in *Lepidium aegrum*, *Lepidium limenophylax*, *Lepidium juvencum* and *Lepidium rekohuense*), absent in two (*Lepidium castellanum* and *Lepidium oleraceum*), and unknown for *Lepidium seditiosum* (see above comments). We also stress that care is needed to observe the presence or absence of a notch. It is best seen in freshly matured silicles because, in older, over mature silicles, the apically initiated disarticulation of the valves can be misleading as it gives the false impression of a notch. Notching was noted by past workers (Kirk 1899; Cheeseman 1906, 1925; Allan 1961; Garnock-Jones 1988) as being especially prominent in *Lepidium banksii* and *Lepidium obtusatum*, as it also is in the new species *Lepidium amissum* (which is segregated here from *Lepidium obtusatum*) and *Lepidium oblitum*. It is odd, therefore, that despite the prominence of the notch in *Lepidium banksii*, Kirk (1899) in his dichotomous key emphasised the cordate silicle base rather than apical notch as diagnostic of *Lepidium banksii* and he did not mention it in his key for *Lepidium obtusatum*. In fact cordate silicle bases are not unique to *Lepidium banksii* as they are also present in *Lepidium oligodontum*. Silicle wings that occur around the margin of the silicle are also important. Of the 16 species for which mature silicles are available, ten have slightly winged silicles and six species (*Lepidium castellanum*, *Lepidium aegrum*, *Lepidium juvencum*, *Lepidium crassum*, *Lepidium juvencum*, *Lepidium oleraceum*) do not.

## Systematics

### Lepidium aegrum sp. nov.

urn:lsid:ipni.org:names:77129253-1

http://species-id.net/wiki/Lepidium_aegrum

A L. oleraceo caulibus gracilibus flexilibus, foliis pallide viridibus membranaceis, lanceolatis elliptico-lanceolatis vel anguste ellipticis, apice prominente acuto vel subacuto, marginibus serratis dentibus prominentibus profundis, et sequentia nucleotidorum DNA distinguenda.

#### Holotype

**Figure 9.**
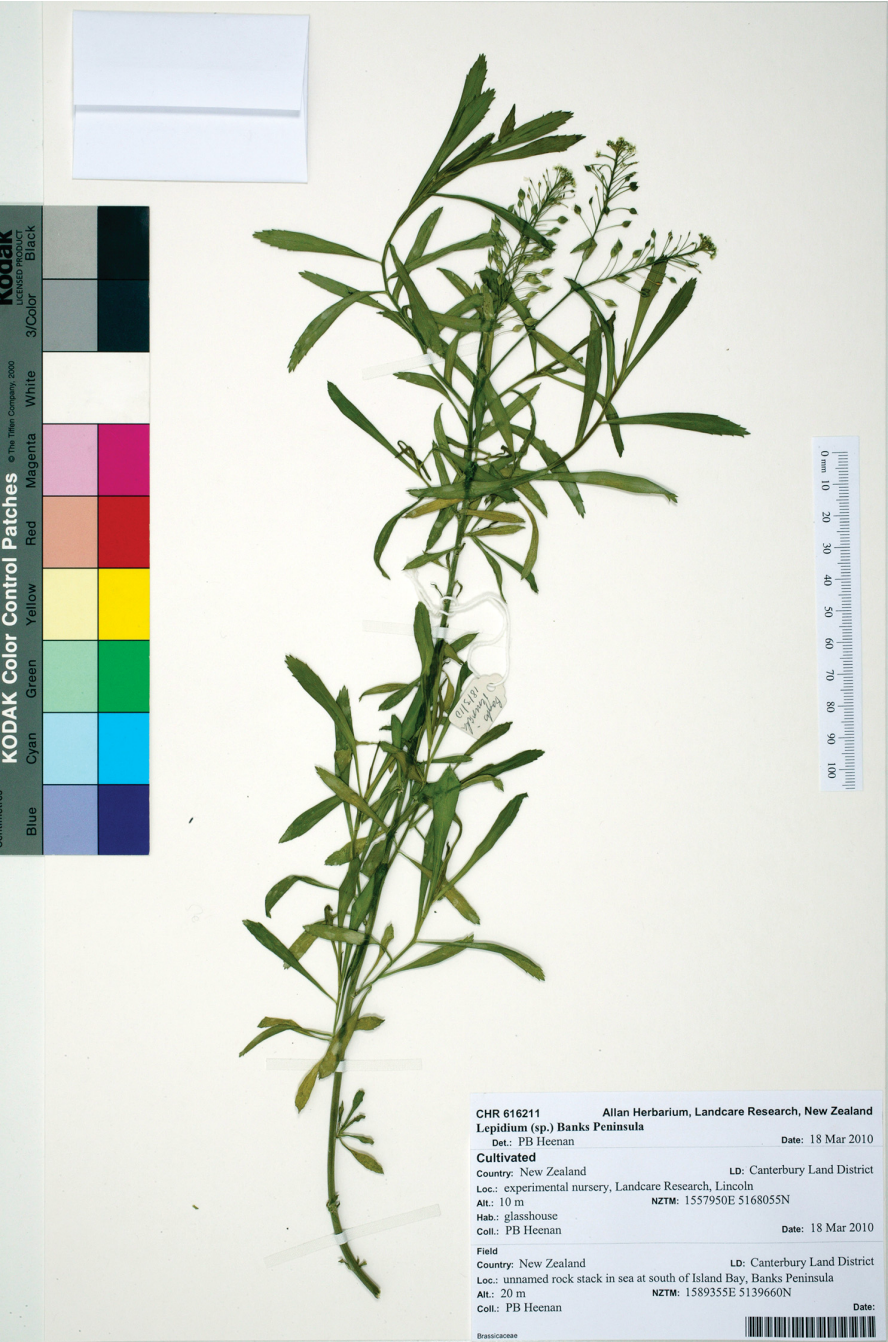
Holotype of *Lepidium aegrum* Heenan et de Lange.

**(Fig. 9). New Zealand:** Canterbury, Lincoln, Landcare Research experimental nursery, ex Banks Peninsula, rock stack near Island Bay, May 2011, P. B. Heenan s.n., CHR 616211!

#### Etymology.

The specific epithet ‘*aegrum*’ is derived from Latin, meaning ‘diseased and/or sick body’ and refers to the condition of the plants in the single known extant wild population on the unnamed rock stack near Island Bay, many of which are infected with turnip mosiac virus (Fletcher et al. 2009).

#### Description

**Figure 10.**
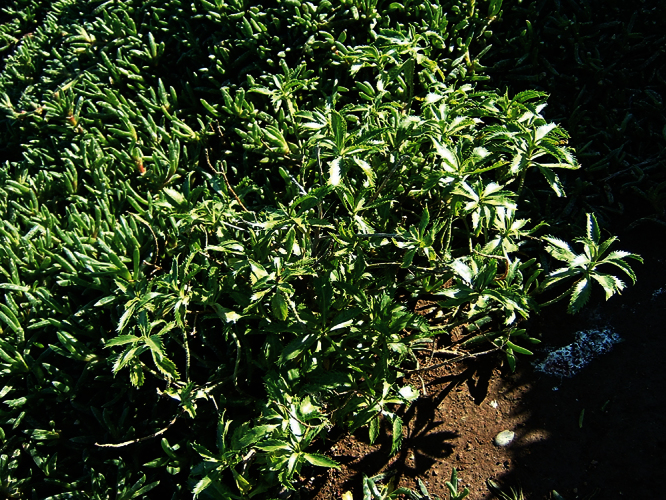
*Lepidium aegrum* plant in the wild showing usual growth habit (*Lepidium aegrum* growing in association with *Disphyma australe* subsp. *australe*) amongst petrel burrows).

**Figure 11.**
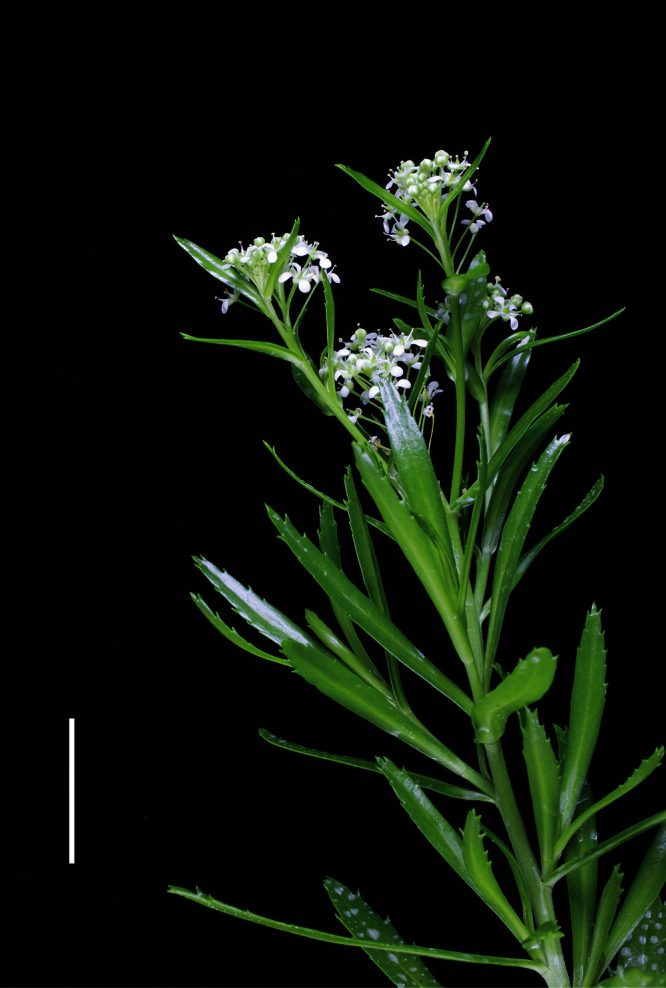
*Lepidium aegrum* stem showing four inflorescences. Scale bar = 20 mm.

**Figure 12.**
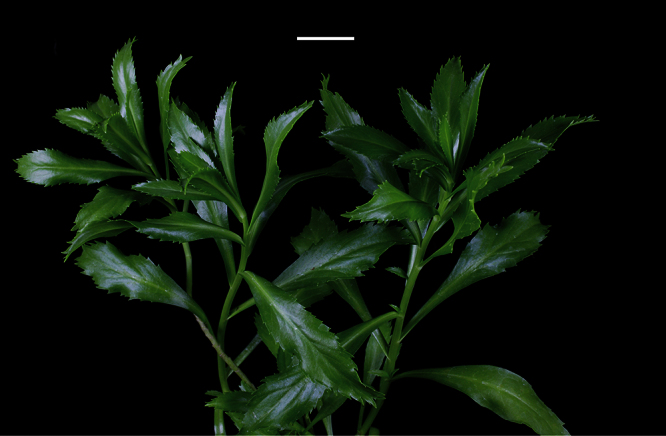
*Lepidium aegrum* leafy stems showing mid-stem and apical foliage. Scale bar = 20 mm.

**Figure 13.**
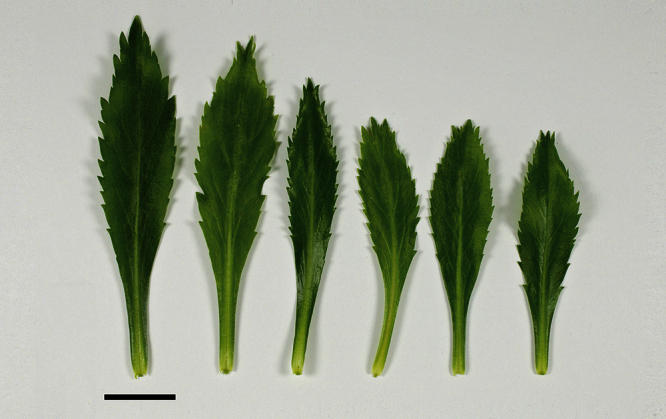
*Lepidium aegrum* (from left to right) basal- to mid-stem foliage. Scale bar = 20 mm.

**Figure 14.**
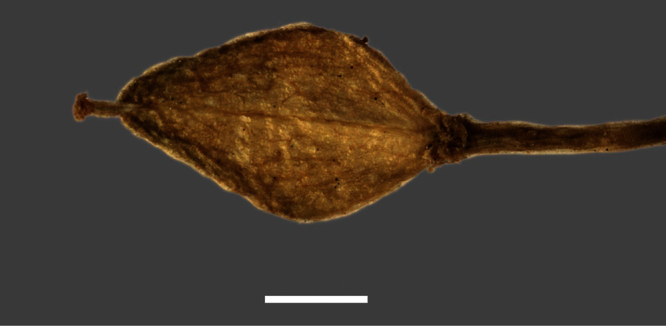
Mature silicle of *Lepidium aegrum*. CHR 222380. Scale bar = 1 mm.

**(Figs 10–14).** Tap-rooted, strongly pungent smelling, perennial herb. Growth habit open, up to 50 cm tall, stems arising from basal woody stems. Stems upright, slender, flexible; mature stems woody, 100–500 × 10–12 mm, often devoid of foliage on middle and lower parts of stems; new stems 80–200 × 3–4 mm, leafy, glabrous. Leaves glabrous, membranous, light green, planar, pellucid glands sometimes scattered on abaxial surface, rosette and stem leaves usually withering, variable in size and shape. Leaves of young and vigorous plants and stems: lamina 35–80 × 10–20 mm, lanceolate, elliptic-lanceolate, narrowly elliptic; apex subacute, with a single prominent tooth; margin singly serrate, with 8–12 pairs of teeth; teeth up to 3.5 mm deep, not overlapping; base attenuate, tapering to distinct petiole. Leaves of mature plants and cauline stems: lamina 30–65 × 4–11 mm, narrowly lanceolate, elliptic-lanceolate, narrowly elliptic; apex acute to subacute, with a single prominent tooth; margin singly serrate in upper and/or lower half, with 2–10 pairs of teeth; teeth up to 1.2 mm deep, not overlapping; base attenuate, tapering to petiole. Inflorescence terminal and lateral, racemose, often branched, up to 95 mm long, rachis up to 2.1 mm diam., glabrous; pedicels 4–10 mm long, erecto-patent, glabrous. Flowers 4.0–4.5 mm diam. Sepals 4, 1.0–1.5 mm long, saccate, green, apex obtuse, margin white, shape dimorphic; lateral sepals broad, 1.0–1.1 mm diam., orbicular, abaxial surface often hairy, hairs entirely eglandular or with glandular tip, 0.2–0.3 mm long; median sepals narrow, 0.8–0.9 mm diam., broadly elliptic, glabrous. Petals white, 2.3–2.5 × 1.2–1.4 mm, spreading, claw 0.7–0.9 mm long; limb broadly elliptic to orbicular, apex obtuse to rounded. Stamens 4; filaments 1.7–2.0 mm long, base 0.3–0.4 mm diam., equal; anthers 0.4–0.5 mm long. Ovary 0.9–1.0 × 0.9–1.0 mm, broadly ovate to broadly elliptic, green, apex usually with shoulders; style 0.3–0.4 mm long, cylindrical; stigma 0.3–0.4 mm diam. Nectaries 4, 0.2–0.3 × c. 0.1 mm, oblong, green. Silicles cartilaginous when fresh, coriaceous when dry, 4.0–4.7 × 3.2–3.5 mm, elliptic-rhomboid to orbicular-rhomboid, apex shallowly notched, valves pale brown,glabrous, not winged; style 0.3–0.4 mm long, exserted. Seeds 1.6–1.7 × 0.9–1.0 mm, narrowly ovoid, brown to orange-brown, not winged. FL Mar. FR Mar.

#### Representative Specimens.

**New Zealand (South Island):** Banks Peninsula, Waikerikeri, n.d., R. M. Laing s.n., (AK 4463); Banks Peninsula, Akaroa, Akaroa Head, Island Bay, Unnamed Islet, July 2002, N. Head s.n., AK 283510. **Cultivated (New Zealand):** Landcare Research experimental nursery, Lincoln, ex Akaroa, Akaroa Head, Island Bay, Unnamed Islet, 5 September 2008, P. J. de Lange 7363 & G. Houliston, (AK 303515);Landcare Research experimental nursery, Lincoln, ex Island Bay, Banks Peninsula, 13 January 2010, P. B. Heenan s.n., (CHR 609820); Landcare Research experimental nursery, Lincoln, ex Island Bay, Banks Peninsula, 13 January 2010, P. B. Heenan s.n., (CHR 609821); Landcare Research experimental nursery, Lincoln, ex Island Bay, Banks Peninsula, 1 April 2009, P. B. Heenan s.n., (CHR 609804); Landcare Research experimental nursery, Lincoln, ex Island Bay, Banks Peninsula, 11 August 2009, P. B. Heenan s.n., (CHR 609792).

**Figure 15.**
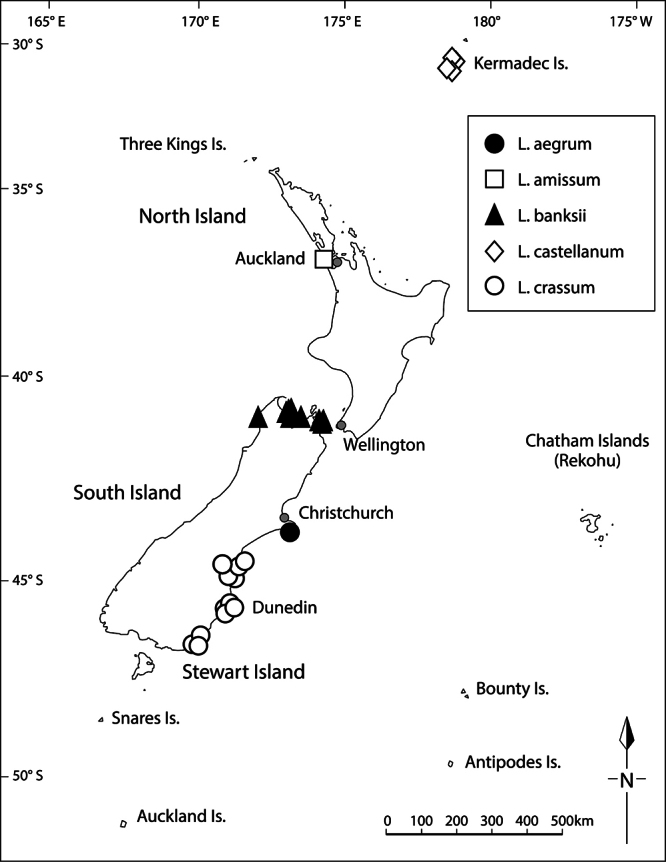
Distribution of *Lepidium aegrum*, *Lepidium amissum*, *Lepidium castellanum* (Kermadec Islands only), *Lepidium banksii*, and *Lepidium crassum*.

**Distribution** **(Fig. 15).** Endemic. New Zealand, South Island, Canterbury, Banks Peninsula, Island Bay, unnamed rock stack.

#### Ecology.

Known from a single wild population on a small and unnamed island near Island Bay. Here it grows in open and disturbed areas among petrel burrows and *Disphyma australe* (W.I.Aiton) N.E.Br. subsp. *australe* (Fig. 6). Additional populations of *Lepidium aegrum* have been established in Canterbury on Motunau Island (Pegasus Bay), Quail Island (Lyttleton Harbour), and at Stony Bay (Banks Peninsula).

**Recognition.** *Lepidium aegrum* is distinguished by its slender flexible stems (Figs 11, 12), light green membranous leaves that are lanceolate, elliptic-lanceolate or narrowly elliptic with a prominent acute to subacute apex, and margins that are serrate with prominent and deep teeth (Fig. 13).

**Conservation Status.** *Lepidium aegrum* is known from a single wild population and three additional populations that have been established with nursery-raised plants. The single wild population and unknown recruitment patterns means that this species is especially vulnerable to stochastic events. Furthermore, turnip mosiac virus, which deforms and retards growth, is common in plants at the wild population, and this will likely reduce reproductive success (Fletcher et al. 2009). Using the New Zealand Threat Classification System (Townsend et al. 2008), *Lepidium aegrum* qualifies as Threatened/Nationally Critical. We recommend appending the qualifiers ‘CD’ (Conservation Dependent – as it has been established and is being managed at Motunau and Quail islands, and at Stony Bay), ‘DP’ (Data Poor – to reflect uncertainty over plant numbers and population trends), ‘OL’ (One location – since it is known from a single wild population).

### Lepidium amissum sp. nov.

urn:lsid:ipni.org:names:77129254-1

http://species-id.net/wiki/Lepidium_amissum

*A L. obtusato habitu suberecto parce ramoso et sine rhizome, caulibus arcutis potius quam flexuosis, foliis rosulae et caulis inferi profunde denticutis persistente oblanceolato-spathulatis ad obovato-spathulatis, foliis caulis superi sparsis lanceolatis ad anguste oblanceolatis vel obdetoideis truncatis et profunde dentatis (raro integris) paribus dentorum usque ad 6 profunde incises et sparsim foliaceis, racemis longioribus quibus non obscuris caulibus vegetativis siliclisque parvioribus et angustioribus differt*.

#### Holotype.

**Figure 16.**
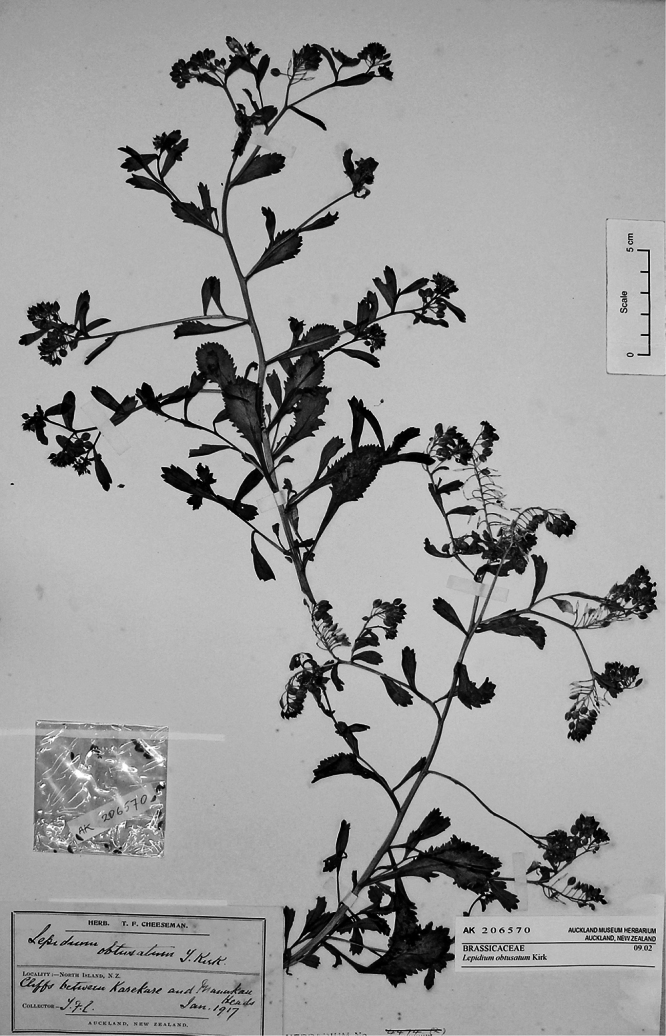
Holotype of *Lepidium amissum* de Lange et Heenan.

**New Zealand (Fig. 16):** Cliffs between Karekare and Manukau Heads, January 1917, T. F. Cheeseman s.n., AK 4474! Isotypes: AK 206570!, WELT SP030095!

#### Etymology.

The epithet ‘*amissum*’ is derived from the Latin ‘*amissus*’ meaning ‘lost’ and is used here to refer to the loss though extinction, of this plant from the New Zealand flora.

#### Description

**Figure 17.**
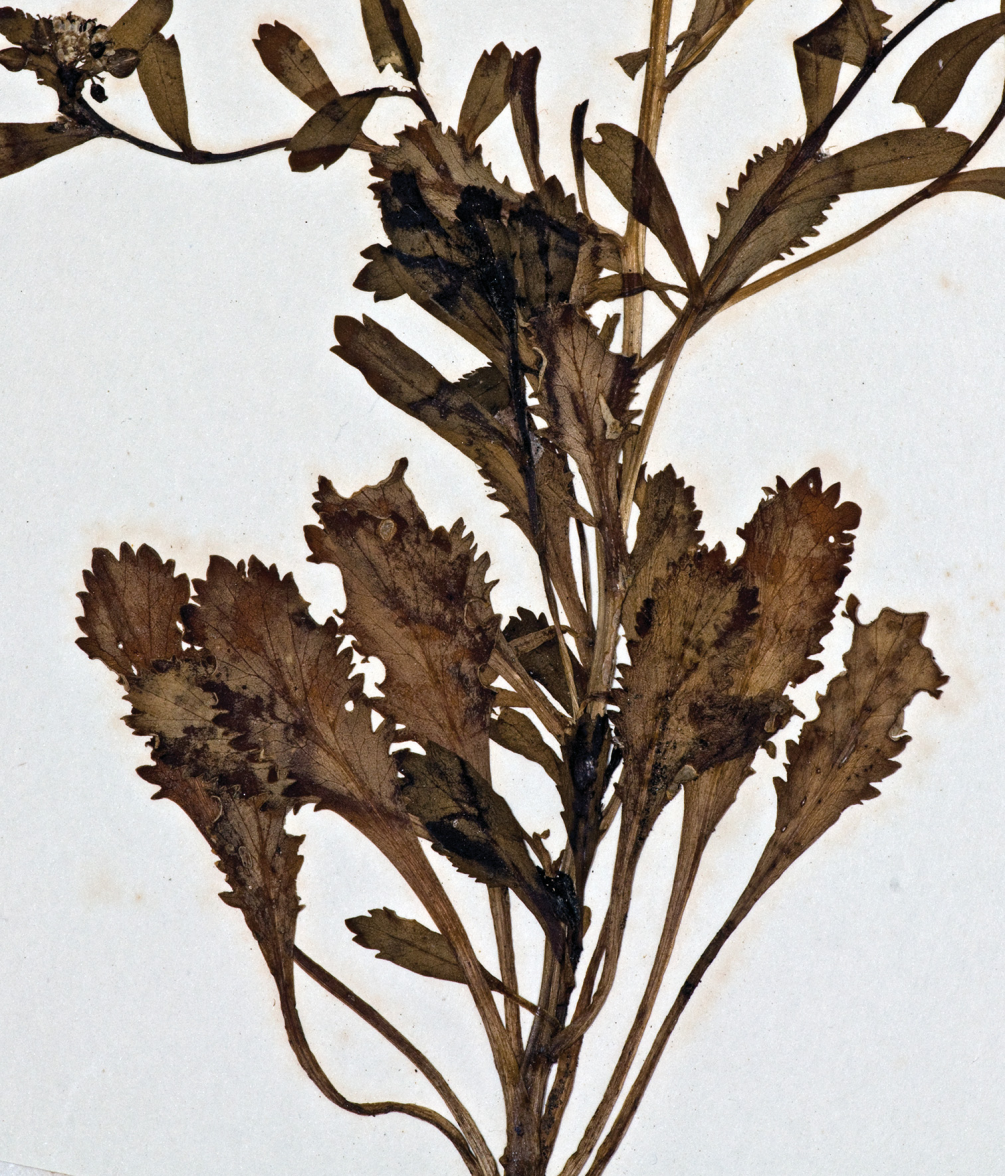
Basal stem leaves of *Lepidium amissum*. AK 4473

**Figure 18.**
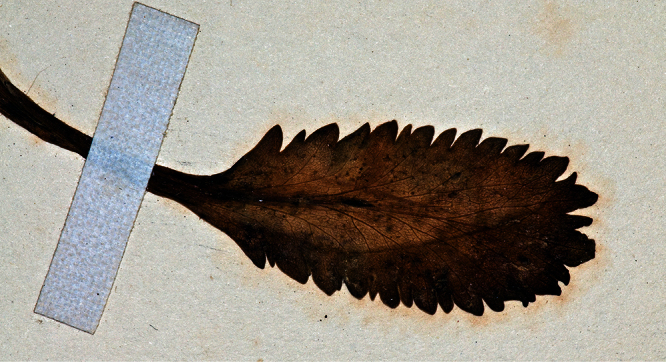
Basal stem leaf of *Lepidium amissum*. AK 4473.

**Figure 19.**
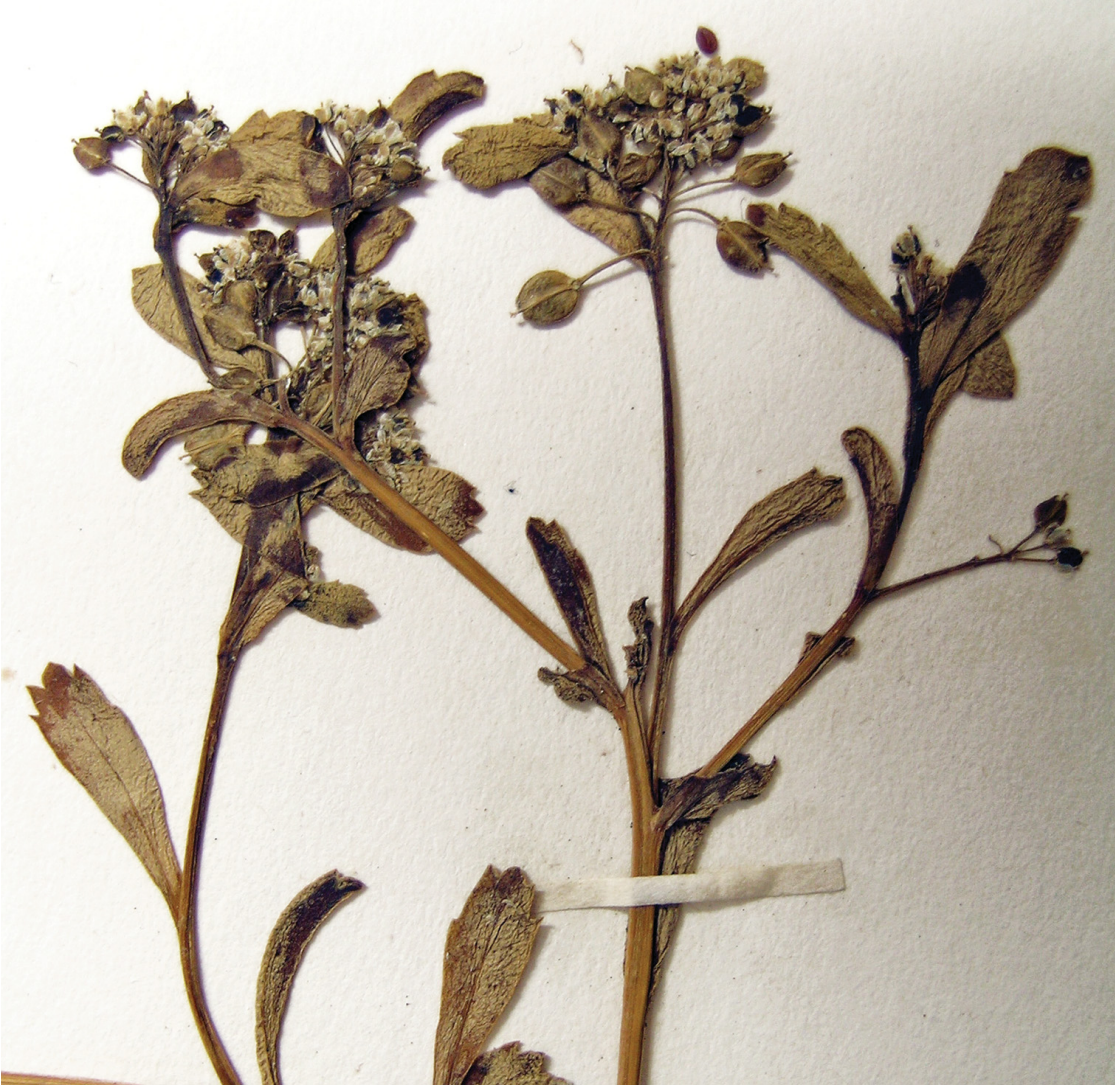
Upper-stem leaves and inflorescences of *Lepidium amissum*. AK 4473.

**Figure 20.**
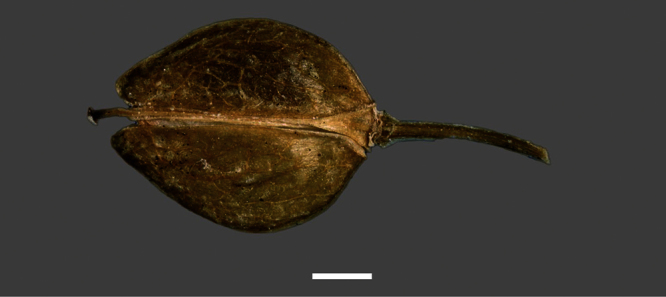
Mature silicle of *Lepidium amissum*. AK 4473. Scale bar = 1 mm.

**(Figs 17–20).** Glabrous, suberect, sparingly branched, succulent, perennial, herb forming open patches up to c. 1 m diam. Rootstock stout, woody, exposed portion rough, covered in old dead stem and rosette-leaf remnants admixed with actively growing stems. Stems arching, widely spreading, possibly with apices weakly ascendent, succulent, mature stems woody, 200–300 × 3–6 mm, densely leafy near base, leaves more widely spaced along upper stems. Leaves glabrous, coriaceous, probably succulent, dark green, planar, variable size and shape. Rosette leaves persistent at fruiting 71.7–95.2 × 12.4–23.2 mm, oblanceolate-spathulate to obovate-spathulate; apex, truncate, praemorse, with 3–8 deeply incised teeth; margin coarsely and often irregularly incised or dentate, often weakly bidentate, with 24–36 pairs of teeth; teeth up to 4.8 mm deep, irregular in size, protruding beyond leaf outline; base narrowly attenuate to cuneate, ± decurrent, petiole distinct, 43.4–55.2 × 1.4–2.3 mm long, slightly winged, or not, channelled. Lower stem leaves similar to rosette leaves, apparently persistent, widely spaced, gradually decreasing in size toward inflorescence; petioles distinct, slightly winged or not. Upper stem leaves much reduced; lamina 10.3–27.5 × 3.4–7.6 mm, lanceolate, narrowly oblanceolate, lanceolate to narrowly obdeltoid, apex truncate, with 3–4 prominent teeth, margins prominently toothed in upper ⅓ of lamina with 4–6 deeply incised pairs of teeth (rarely entire except for apex), base cuneate to broadly cuneate; petiole distinct or indistinct, up to 6 mm long when present, channelled. Inflorescence racemose, terminal and lateral, conspicuous, sparingly leafy and unfettered by associated vegetative leafy stems; racemes 22–68 mm long, rachis 0.9–1.6 mm diam., glabrous; pedicels 2.8–3.2 mm long at flowering, erecto-patent, elongating somewhat after anthesis, glabrous. Flowers 4.3–4.6 mm diam. Sepals 4, saccate, overlapping at base, green, apex obtuse, margin white, shape and size dimorphic; lateral sepals 2.2–2.9 × 2.1–3.0 mm, suborbicular, mostly glabrous, sometimes sparsely hairy, hairs 0.2–0.4 mm long, caducous; median sepals 1.9–2.9 × 1.5–1.7 mm, broadly elliptic to obovate, abaxial surface glabrescent, sparsely hairy, hairs 0.2–0.4 mm long, caducous. Petals white, 1.3–1.8 × 1.3–1.8 mm, erect, claw minute, 0.2–0.3 mm; limb orbicular, apex obtuse. Stamens 4, ± equal lengths, 1.2–1.8 mm long, base 0.6–1.0 mm wide; anthers 0.6–0.8 mm long, yellow, pollen yellow. Ovary 1.3–1.8 × 1.3–1.7 mm, broadly ovate to suborbicular green, apex distinctly notched; style 0.3–0.5 mm long, cylindrical below, spreading at apex; stigma 0.5–0.6 mm diam. Nectaries 4, green, 0.12–0.14 × c. 0.09 mm, narrow oblong, apex obtuse. Silicles 3.4–4.5 × 2.9–3.9 mm, broadly ovate, oval to obovate, apex prominently notched, valves yellow-green (in dried specimens), glabrous, slightly winged; style 0.2–0.5 mm long, not or only slightly exserted. Seeds 2.0–2.7 × 1.8–2.0 mm, obovate, broadly obovate, brown to orange-brown, not winged. FL: Dec–Jan. FR: Dec–Jan.

#### Representative Specimen.

**New Zealand (North Island)**: North Auckland, Cliffs north of the Manukau heads, December 1870, T. F. Cheeseman s.n., (AK 4473).

#### Distribution

**(Fig. 15).** Endemic. New Zealand, North Island, where it was recorded from ‘between Manukau Heads and Karekare’ (Cheeseman 1906, 1925), not the ‘Titirangi’ noted by Kirk (1899). *Lepidium amissum* was last collected from the west Auckland coastline in 1917 and is presumed to be extinct.

#### Recognition.

*Lepidium amissum* had previously been included in *Lepidium obtusatum*, perhaps because the species have no obvious floral differences and both have deeply notched and winged silicles with tapering bases. However, from *Lepidium obtusatum*, *Lepidium amissum* differs by the non-rhizomatous, suberect growth habit; arching, sparingly leafy stems (Fig. 16); long persistent, deeply, and sharply incised rosette and lower stem leaves (Figs 17, 18); upper stem leaves which are lanceolate, narrowly oblanceolate, to narrowly obdeltoid and mostly prominently toothed, with up to 6 pairs of deeply incised teeth, and with a truncate apex bearing 3–4 prominent teeth. The inflorescences of *Lepidium amissum* are larger, only sparsely leafy when mature, and without associated vegetative stems (Fig. 19). The silicles are smaller than those of *Lepidium obtusatum* (Fig. 20).

#### Ecology.

Little is known about the habitat preferences and ecology of *Lepidium amissum*, beyond that it grew on sea cliffs (Cheeseman 1906, 1925).

#### Conservation Status.

*Lepidium amissum* is considered to be extinct. There have been repeated unsuccessful surveys for this species over the last fifty years by various botanists. Aside from the type the species is known from only one collection made in 1917. This collection and the type came from an ill defined area of coastline stretching for some 6 km from the Manukau Heads to Karekare. Today this coastline is protected within the Centennial Park, Waitakere Ranges. However, during the period when this species was gathered, all of the coastline was being modified as a result of kauri (*Agathis australis* (D.Don) Lindl.) logging (see Harvey 2001, 2006; MacDonald and Kerr 2009), and this may have contributed to its eventual extirpation. It does seem that *Lepidium amissum* was already uncommon at the time of its discovery and it is possible that the gatherings made by Thomas Cheeseman where sufficient to cause its extinction as they are copious and comprise many stems.

### Lepidium banksii

Kirk, Stud. Fl. N. Z, 35 (1899)

http://species-id.net/wiki/Lepidium_banksii

#### Type Collection:

“SOUTH Island: Queen Charlotte Sound and Astrolabe Habrour, A. Richard, l.c.”

#### Lectotype

**Figure 21.**
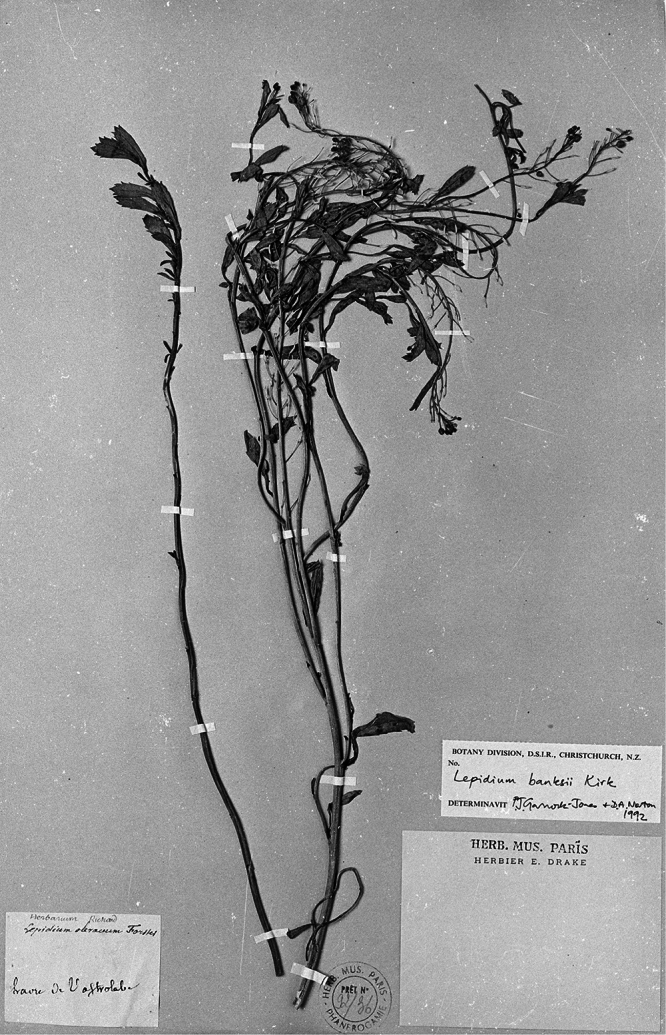
Lectotype of *Lepidium banksii*.

**(Fig. 21, designated here):** Herbarium Richard, Paris!

#### Etymology.

Although Kirk (1899) did not explain his choice of epithet, ‘*banksii*’ commemorates Sir Joseph Banks FLS, FRS (1743–1820) who together with Dr Daniel Solander made the first gatherings of New Zealand plants to be described by European botanists during the Endeavour voyage of discovery (1768–1771).

*= Lepidium banksii* var. *ovatum* Kirk, *Stud. Fl. N. Z*., 35 (1899)

#### Lectotype

**(*fide*Allan 1961):** Northwest Bay, Pelorus Sound, J. Rutland s.n., n.d., WELT SP079012!

#### Etymology.

Although Kirk (1899) did not explain his choice of epithet, the protologue makes it clear that “*ovatum*” was chosen to refer to the shape of the mature silicles.

#### Note:

The key provided by Kirk (1899, p. 34) used the name “*Lepidium forsteri*” instead of *Lepidium banksii*. This is considered to be a nomen nudum.

#### Description

**Figure 22.**
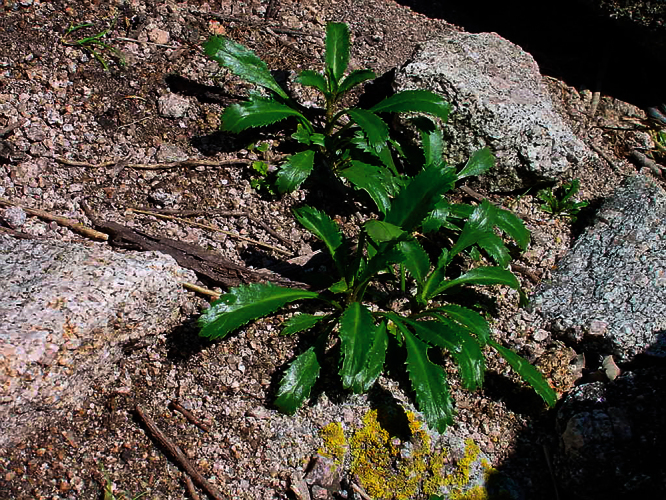
Young wild plant of *Lepidium banksii* (image: S. Walls).

**Figure 23.**
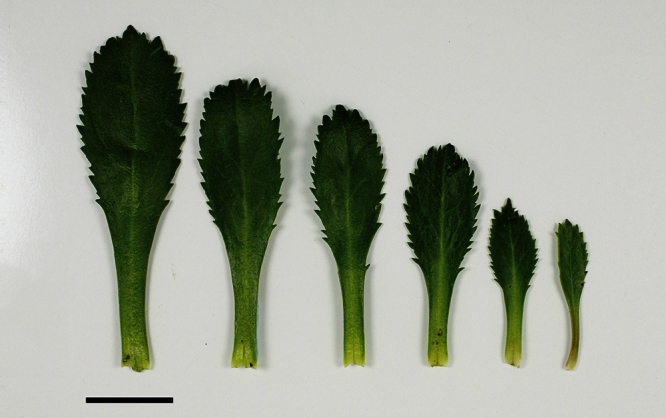
(From left to right) rosette-, basal- and upper-stem leaves of *Lepidium banksii*. Scale bar = 20 mm.

**Figure 24.**
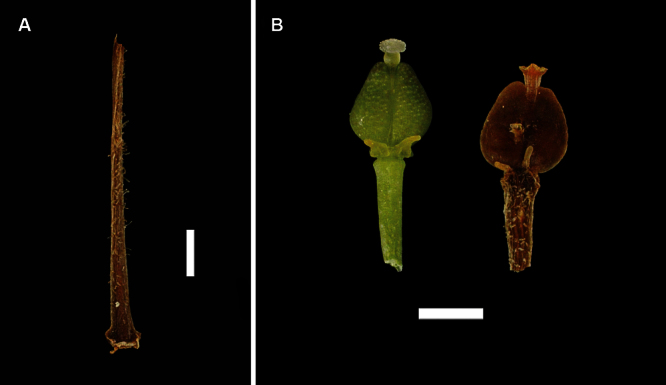
(**A**) Pedicel of *Lepidium banksii* showing hairs, (**B**) emasculated flowers showing nectaries, ovary and stigma. Scale bars = 1 mm

**Figure 25.**
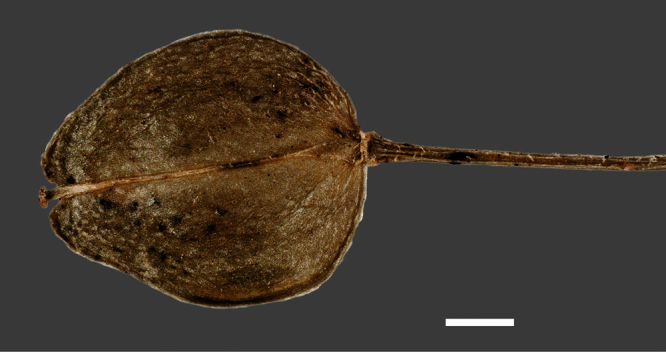
Mature silicle of *Lepidium banksii*. CHR 509034. Scale bar = 1 mm.

**(Figs 22–25).** Tap-rooted, strongly pungent smelling, perennial herb. Growth habit dense, stems closely placed, 20–50 cm tall. Stems upright to spreading, stout, barely flexuous; mature stems woody, 100–500 × 3–8 mm, often devoid of foliage on middle and lower parts of stems. Leaves glabrous, coriaceous, green, planar, rosette and stem leaves usually withering, variable in size and shape. Leaves of young and vigorous plants and stems: lamina 20–40 × 6–15 mm, oblanceolate-spathulate, obovate; apex obtuse, often with up to 3 or 4 teeth; margin coarsely serrate, with 15–21 pairs of teeth; teeth up to 2.0 mm deep, irregular in size, protruding beyond leaf outline; base attenuate to cuneate, petiole distinct; petiole up to 35.0 × 1.3–2.8 mm, channelled. Leaves of mature plants and cauline stems: lamina 8–25 × 3–6 mm, linear-oblanceolate, obovate; apex obtuse to truncate, often with up to 3 or 4 teeth; margin serrate in upper half, up to 7 pairs of teeth; not overlapping, up to 1.5 mm deep, often protruding beyond leaf outline; base attenuate to cuneate, usually tapering to ± distinct petiole, sometimes appearing sessile; petiole up to 8.0 × 1.0–1.8 mm, channelled. Inflorescences terminal and lateral, racemose, 20–80 mm long, rachis 0.6–1.4 mm diam., glabrous or sometimes with pale clavate hairs; pedicels 5–8 mm long, erecto-patent, with pale clavate hairs on adaxial surface, hairs 0.1–0.15 mm long. Flowers 4.0–4.5 mm diam. Sepals 4, saccate, overlapping at base, green, apex obtuse, margin white, shape and size dimorphic; lateral sepals 1.6–2.1 × 1.1–1.5 mm, orbicular, glabrous; median sepals 1.5–1.9 × 0.9–1.1 mm, broadly elliptic, abaxial surface glabrous or sparsely hairy, hairs 0.2–0.4 mm long. Petals white, 1.8–2.0 × 0.1–0.9 mm, erect, claw indistinct; limb narrrowly obovate, elliptic or filiform, often irregular in shape, apex obtuse to subacute. Stamens 4, ± equal lengths, 1.2–1.7 mm long, base 0.6–0.9 mm wide; anthers 0.4–0.7 mm long, yellow or sometimes violet. Ovary 1.4–1.6 × 1.0–1.6 mm, broadly ovate, green, apex round or sometimes weakly shouldered; style 0.2–0.3 mm long, cylindrical below, spreading at apex; stigma 0.45–0.5 mm diam. Nectaries 4, 0.2–0.4 × c. 0.1 mm, oblong, green. Silicles cartilaginous when fresh, coriaceous when dry, 4.5–5.5 × 4.0–5.0 mm, broadly ovate, apex notched, base cordate, valves green maturing yellow-green, glabrous, slightly winged; style 0.2–0.3 mm long, exserted. Seeds 1.8–2.3 × 1.0–1.2 mm, obovate or obovate-elliptic, brown to orange-brown, not winged. FL Nov–Jan. FR Nov–Jan.

#### Representative Specimens.

**New Zealand (South Island):** Tennyson Inlet, Tawa Bay, January 1910, E. Phillips-Turner 93, (AK 100087); Kenepuru Head, Pipi’s Beach, 1917, J. H. MacMahon s.n., (WELT SP030112); Kenepuru, n.d., n.c., , (WELT SP030113); ?Queen Charlotte Sound, n.d., J. H. MacMahon s.n., (WELT SP081929); Boulder Bank, November 1908, F. G. Gibbs s.n., (CHR 81737); Abel Tasman National Park, Totaranui Headland, December 1961, A. E. Esler s.n., (AK 218197); Abel Tasman National Park, Totaranui Headland, 27 January 1963, A. E. Esler s.n., (CHR 181143); Abel Tasman National Park, Totaranui Point, 12 January 1984, D. R. Given 13871 & A. D. Given, (CHR 509034); Abel Tasman National Park, Totaranui, 12 January 1986, D. R. Given 14106, (CHR 420352); Abel Tasman National Park, Totaranui Trig, 9 January 1984, D. R. Given 13505 & B. A. Duncan, (CHR 416178); Abel Tasman National Park, Separation Point, 28 January 1997, P. J. de Lange 3249 & G. M. Crowcroft, (AK 231008); Karamea, 28 January 1985, P. Wardle s.n., (CHR 446772); Bird Island, Waimea Estuary, 1946, C. Baas s.n., (CHR 278958). **Cultivated (New Zealand).** Landcare Research experimental nursery, Lincoln, ex Totaranui, Nelson, 17 November 2005, P. B. Heenan s.n., (CHR 504666); Landcare Research experimental nursery, Lincoln, ex Abel Tasman National Park, 6 August 2006, P. B. Heenan s.n., (CHR 609830).

#### Distribution

**(Fig. 15)**. Endemic. New Zealand, South Island, Marlborough Sounds (Queen Charlotte, Kenepuru and Pelorus Sounds), Nelson (Waimea Estuary, Totaranui, Separation Point, Wainui Inlet), north-west Nelson (Karamea).

#### Recognition.

*Lepidium banksii* is recognised by the clavate hairs on the pedicels (Fig. 24A), mostly filiform petals, styles that spread at the apex into a broad plate (Fig. 24B), and silicles that have a prominent apical notch (Fig. 25). In comparison, the styles of the other *Lepidium* species except for *Lepidium seditiosum*, are cylindrical for their whole length.

#### Ecology.

*Lepidium banksii* is a strictly coastal species favouring rubble slopes, boulder beaches, exposed rocky headlands (Fig. 22), and sparsely vegetated cliff faces usually near penguin colonies or low lying, estuarine shell banks and sand and shell barrier islands used as high tide roosts by wading birds.

#### Conservation Status.

*Lepidium banksii* is currently listed as ‘Threatened/Nationally Critical _CD, EF_’ by de Lange et al. (2009). Both de Lange (2006) and Courtney (2009) describe the conservation management of this species, noting that there are probably no naturally occurring plants of *Lepidium banksii* left in the wild, with all those known the result of deliberate plantings and sowing of seed. As such, the qualifier ‘EW’ (Extinct in Wild) should be added to the current threat listing. The most recent census of the numbers of *Lepidium banksii* present in the ‘wild’ records 113 plants (Courtney 2009). Survival of this species is now completely dependent on direct hands-on management (de Lange 2006; Courtney 2009).

### Lepidium castellanum sp. nov.

urn:lsid:ipni.org:names:77129255-1

http://species-id.net/wiki/Lepidium_castellanum

*A Lepidio oleraceo sympatrico sed foliis in caule medio ad supero anguste lanceolatis ad linearo-lanceolatis dentibus acicularibus, apicibus foliorum decresento-acuminatis, et pedicellis minutis et sparsim puberuli differt, et sequentia nucleotidorum DNA distinguenda*.

#### Holotype.

**Figure 26.**
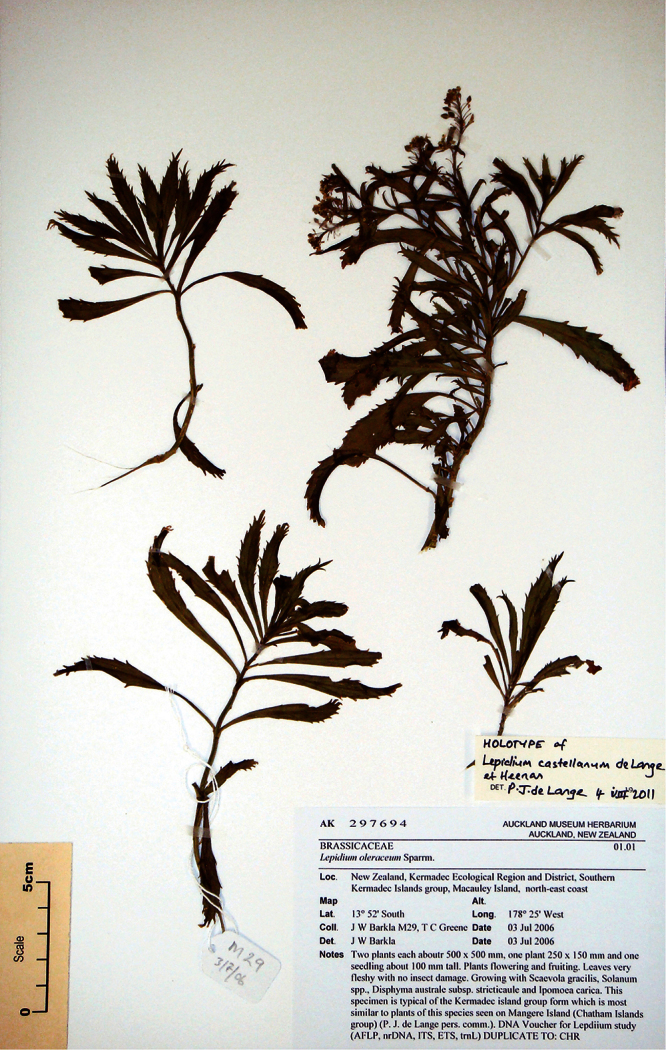
Holotype of *Lepidium castellanum* de Lange et Heenan.

**Kermadec Islands (Fig. 26):** Macauley Island, north-east coast, 3 July 2006, J. W. Barkla M29 & T. C. Greene, AK 297694! Isotype: CHR 552474!

#### Etymology.

The epithet ‘*castellanum*’ from the Latin ‘associated with a fort, fortress or castle’ refers to the remote, craggy, castle-like habitats of this species in the southern Kermadec Islands group on which islands it is endemic.

#### Description

**Figure 27.**
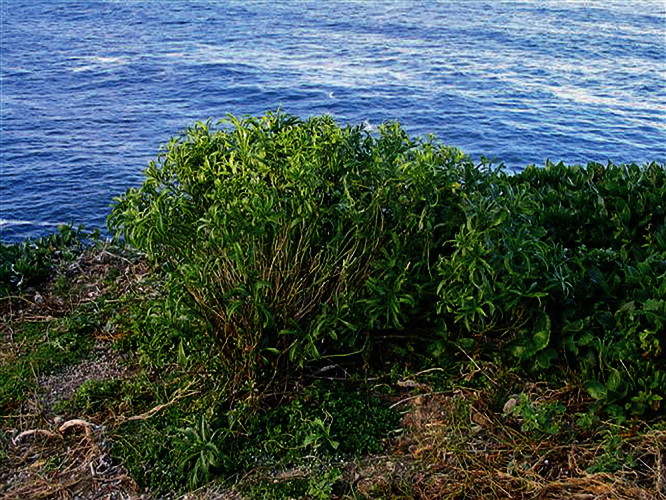
(**A**) Wild plant of *Lepidium castellanum* on cliff top of Macauley Island showing growth habit (plant growing with *Scaevola gracilis*, *Tetragonia tetragonioides* and *Disphyma australe* subsp. *stricticaule*) (image: J.W. Barkla).

**Figure 28.**
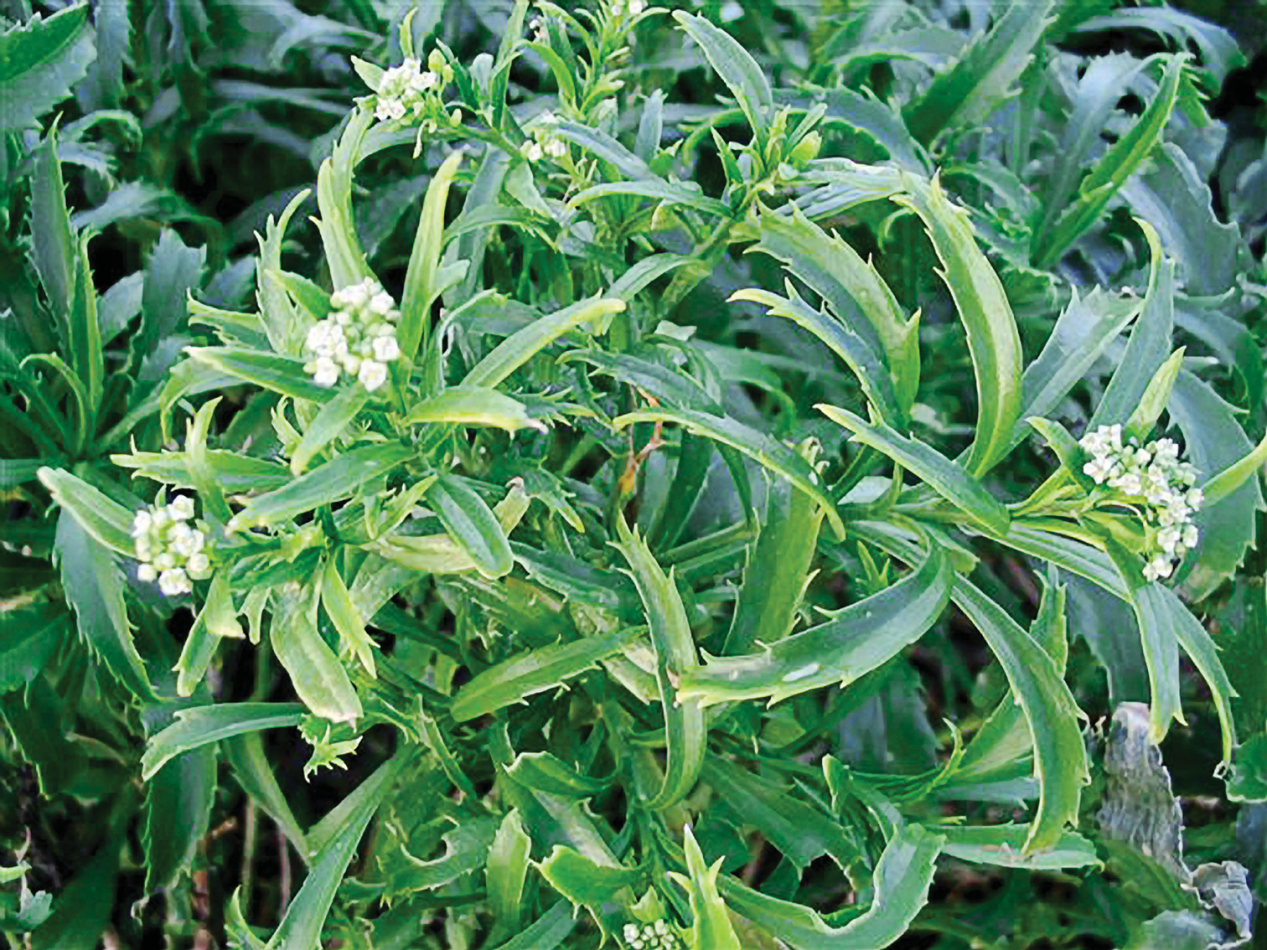
Young inflorescences and upper-stem leaves of *Lepidium castellanum* (image: J.W. Barkla).

**Figure 29.**
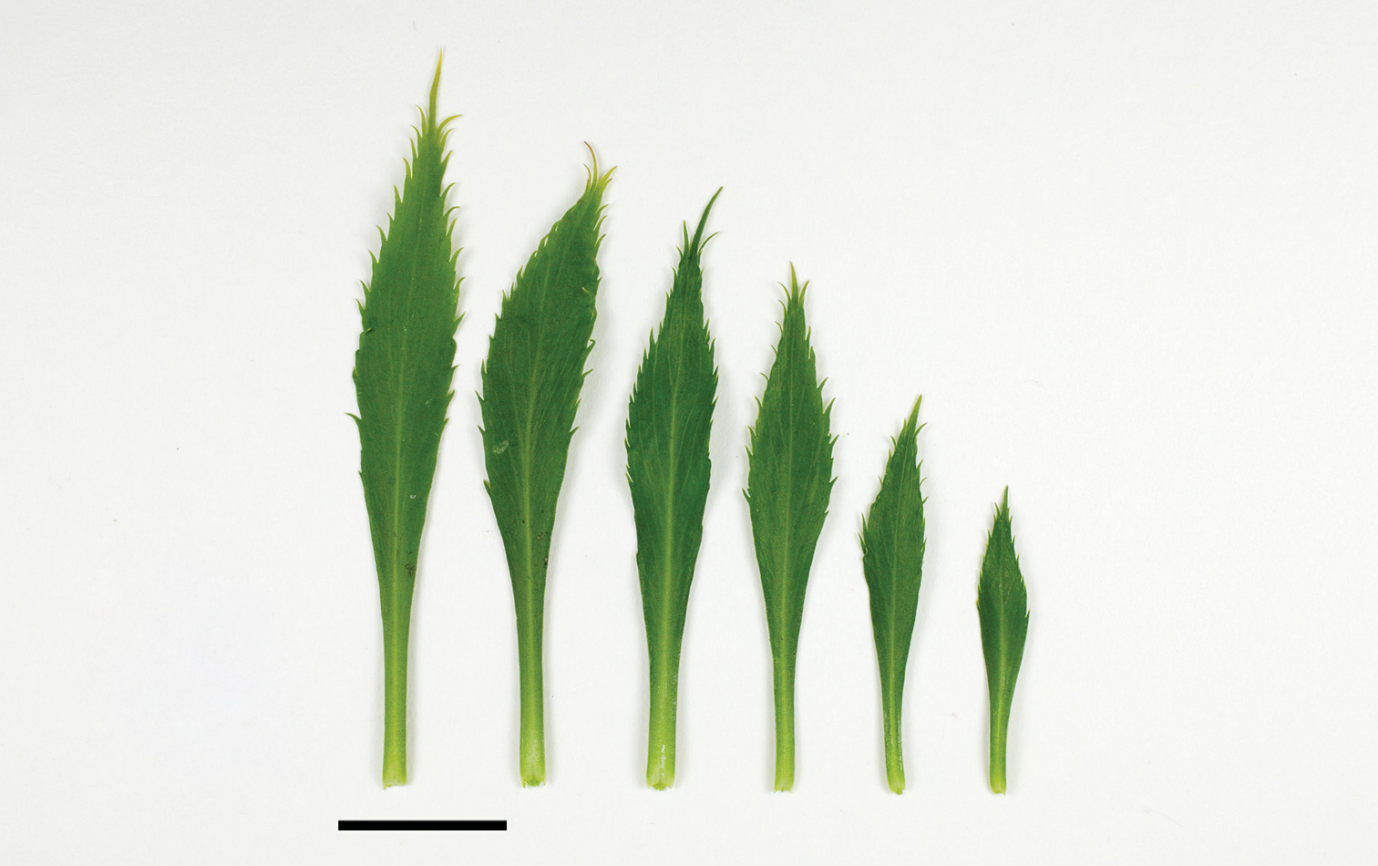
(From leaf to right) basal-, mid- and upper-stem leaves of *Lepidium castellanum*. Scale bar = 20 mm.

**Figure 30.**
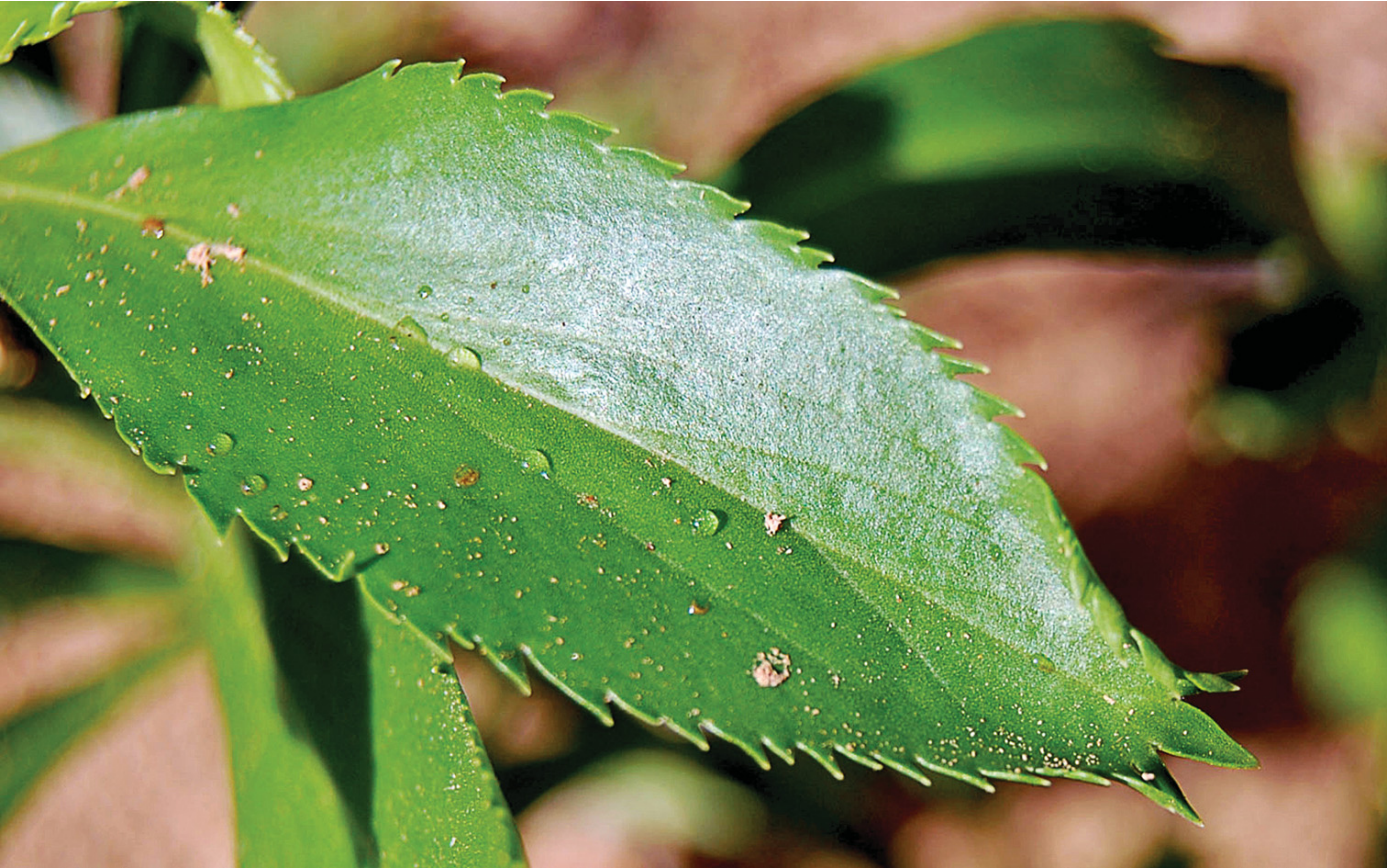
**C**lose up of basal-stem leaf of *Lepidium castellanum* on Cheeseman Island (image: W. Chinn).

**Figure 31.**
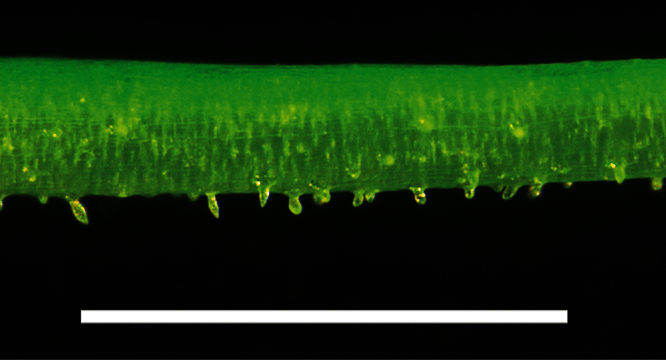
Pedicel hairs of *Lepidium castellanum*. Scale bar = 1 mm.

**Figure 32.**
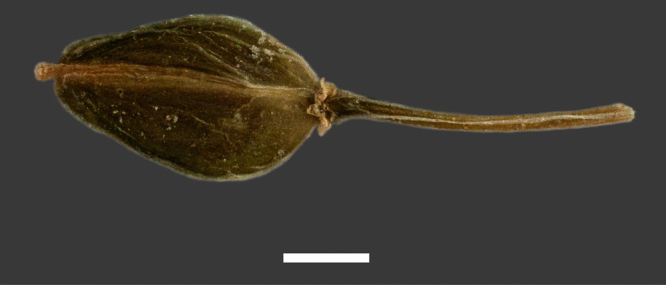
Mature silicle of *Lepidium castellanum*. From holotype (AK 297694). Scale bar = 1 mm).

**(Figs 27–32).** Tap-rooted, strongly pungent smelling, much-branched, leafy perennial shrub, up to 1.8 × 2.0 m. Tap-root up to 800 mm long, ± napiform and/or scarcely branched. Rootstock 6–10 mm diam., woody, exposed portion smooth. Stems persistent, arising from rootstock base and basal portion of main central stem, closely packed, woody, erect, weakly angled to ± terete, glabrous; mature stems 3.8–8.2 mm diam., 0.3–1.8 m long; red-green to yellow-green, brittle, bases bearing numerous leaf abscission scars otherwise mostly leafy from mid-stem to apex at flowering; middle stems dark green to red-green, fleshy and pliant, initially ± square, prominently angled, becoming ± terete with age. Leaves coriaceous, fleshy, green to dark green, rosette-leaves absent, stem leaves withering with age; basal stem leaves 88.5–120.0 × 14.5–30.0 mm, lamina broadly lanceolate to lanceolate, margin ± deeply and ± evenly incised, teeth in 50–90 ± equal pairs, 0.5–2.9 mm long, protruding beyond leaf line, narrowly deltoid, to deltoid, leaf apex truncate-praemorse often deeply lacerate, teeth 3–5, cut 2.4–4.2 mm to lamina, narrowly deltoid, to deltoid, often bidentate, leaf base attenuate extending into a broad petiole wing; petiole distinct, 23.6–31.0 × 3.1–3.3 mm, decurrent, channelled, often with a broadly sheathing base; upper stem leaves 50.0–68.0 × 4.4–12.2 mm, decreasing in size toward inflorescences, lamina narrowly lanceolate to linear-lanceolate, margin ± deeply and ± evenly incised, teeth in 6–14 widely and evenly spaced ± pairs, 1.5–7.2 mm long, protruding beyond leaf line, narrowly deltoid, tapering, acerose, ± acicular to acicular-falcate; lamina apex acute (rarely truncate-praemorse), acuminate, acumen 5.8–11.0 mm long, margins of acumen toothed, teeth often bidentate, 8–10 mm long, acerose, often acicular, or acicular-falcate, leaf base attenuate extending into a narrow petiole wing; petiole distinct, 2.9–8.2 × 1.2–3.3 mm, decurrent, channelled, often with a broadly sheathing base. Inflorescences racemose, 50–100 mm long at fruiting; rachis 0.5–2.25 mm diam., terminal and lateral, leaf-opposed, often long-persistent, sparsely to densely covered in pale, patent, ± clavate hairs or rarely glabrous, hairs 0.1–0.14 mm long; pedicels 5.6–7.2 mm long at fruiting, erecto-patent, with sparse, pale, patent, clavate hairs on adaxial surface, hairs 0.1–0.12 mm long. Flowers 3.0–4.5 mm diam., fragrant. Sepals 4, saccate, ± overlapping at base, lateral sepals broad, 0.5–1.5 mm diam., orbicular, pale to dark green with a broad white, ± undulose margin, apex rounded to obtuse, abaxial surface often hairy, hairs 0.2–0.4 mm long, eglandular or with glandular tip, mostly shedding at anthesis except near base, median sepals 0.5–0.9 mm diam., broadly elliptic, pale to dark green with a broad white, ± undulose margin, apex rounded to obtuse, abaxial surface glabrous. Petals white, 1.1–2.0 × 1.0–1.6 mm, spreading, claw 0.4–0.8 mm long; limb obovate, obovate-spathulate to orbicular, apex obtuse to rounded often slightly emarginate, margins smooth, sometimes weakly undulose. Stamens 4, filaments 1.2–2.0 mm long, white; anthers 0.3–0.5 mm long, yellow. Ovary 1.1–1.8 × 0.6–1.3 mm, ovate, broadly ovate to elliptic, green-brown, apex subacute; style 0.11–0.4 mm long, cylindrical; stigma 0.2–0.5 mm diam. Nectaries 4, 0.2–0.3 × 0.1–0.15 mm, narrow-oblong to deltoid, pale translucent green. Silicles cartilaginous when fresh, coriaceous when dry, 2.4–3.6 × 1.8–2.5 mm, elliptic to rhomboid, apex acute and tapering, valves green maturing grey-white, glabrous, scarcely separating at apex at maturity, not winged; style 0.3–0.7 mm long, exserted. Seeds 2, 1.3–1.9 × 0.8–1.6 mm, narrowly to broadly ovoid, brown to orange-brown, not winged. FL Jul–Jun. FR Sep–Jul.

#### Representative Specimens.

**Kermadec Islands:** Curtis I., 18 July 1969, W. R. Sykes 836/K, (CHR 193789); Haszard Islet, 21 July 2002, T. Greene s.n., P. Dilks & P. Scofield, (AK 258039); Cheeseman Island, 19 November 1971, W. R. Sykes 932/K, (CHR 211838); Cheeseman Island, 26 July 2002, T. Greene s.n., P. Dilks & P. Scofield, (AK 258038);Cheeseman Island, “Te Mimi Paora Rock”, 24 May 2011, P. J. de Lange K850, (AK 326008); Cheeseman Island, “The Castle”, 24 May 2011, P. J. de Lange K851, (AK 326009); Cheeseman Island, “The Castle”, 24 May 2011, P. J. de Lange K852, (AK 326010). **Cultivated (New Zealand):** ex Curtis I. from CHR 193789, 9 January 1970, W. R. Sykes 901/K, (CHR 20188A); ex. Curtis I. from CHR 193789, 25 February 1971, W. R. Sykes 901/K, (CHR 201088B); Lincoln, ex Macauley Island, Landcare Research experimental nursery, 10 July 2008, P. B. Heenan s.n., (CHR 609796); Lincoln, ex Macauley Island, Landcare Research experimental nursery, 2 October 2009, P. B. Heenan s.n., (CHR 609806).

#### Distribution

**(Fig. 15).** Endemic. Kermadec Islands (Macauley Island, Curtis Island, Haszard Islet and Cheeseman Island).

#### Recognition.

*Lepidium castellanum* is distinguished by its very robust, shrubby growth habit (Fig. 27), sometimes up to 1.8 × 2.0 m, erect, often closely packed, usually leafy stems, narrowly lanceolate to linear-lanceolate upper stem leaves, and by the very long, needle-shaped teeth of the leaves which reach well beyond the leaf margin (Figs 28–30). In this species, the pedicels (and often the inflorescence rachises) are minutely hairy (Fig. 31), whilst those of *Lepidium oleraceum* are glabrous. *Lepidium castellanum* is sympatric with *Lepidium oleraceum* on Curtis Island. Elsewhere, in northern New Zealand, especially on the Poor Knights and Hen (Taranga) islands, narrow-leaved forms of *Lepidium oleraceum* are also common. However, these plants have a much smaller growth habit, their leaves lack the distinctive long needle-shaped teeth and long-acuminate leaf apices of *Lepidium castellanum*, and, as with *Lepidium oleraceum* populations elsewhere, their pedicels are glabrous.

#### Ecology.

*Lepidium castellanum* is a sparse associate of the vegetation that has colonised the soft, erosion-prone cliff tops of Macauley Island, the less geothermally active summit slopes of Curtis Island, and the rocky tops of Haszard Islet and Cheeseman Island. On Macauley Island, *Lepidium castellanum* was recorded from open or sparsely vegetated, petrel-burrowed ground where it formed windswept shrubs in association with the creeping *Scaevola gracilis* Hook.f., tangles of *Ipomoea cairica* (L.) Sweet., *Disphyma australe* subsp. *stricticaule* Chinnock and *Polycarpon tetraphyllum* (L.) L. (Barkla et al. 2008; J. W. Barkla and T. C. Greene pers. comm.). On Curtis Island, it has been recorded growing amongst *Disphyma australe* subsp. *stricticaule*, while on the extensively seabird-burrowed Haszard Islet, it is one of the main shrub species, growing there in association with *Myoporum rapense* subsp. *kermadecense* (Sykes) Chinnock, *Cyperus insularis* Heenan et de Lange, *Tetragonia tetragonioides* (Pall.) Kuntze, and *Parietaria debilis* G.Forst. On Cheeseman Island, numerous seedlings and a few adults were seen in 2011 growing on heavily petrel-burrowed soil covered ledges amongst *Disphyma australe* subsp. *stricticaule*, and also as a few sub-adults within an active petrel colony in a narrow gully, growing in association with *Cyperus insularis*, *Tetragonia tetragonioides*, and *Parietaria debilis*.

#### Conservation Status.

*Lepidium castellanum* is confined to the Southern Kermadec Island group (see de Lange et al. 2004), part of the Kermadec Islands Nature Reserve. Within the southern Kermadec Islands group it has been recorded from Macauley Island, Cheeseman Island, Curtis Island, and from Haszard Islet. On Macauley Island it was recorded twice, in 2002 and 2006 (Barkla et al. (2008); T. C. Greene pers. comm.), at that time numbering at best five or fewer individuals. However, it was not seen on Macauley Island in 2011 (de Lange 2012; de Lange in press). In 2002 and 2006, the summit of Haszard Islet (6 ha, of which c.2.1 ha is vegetated) was thoroughly explored during helicopter-assisted landings by ornithologists. During those visits, it was noted that *Lepidium castellanum* was one of the major shrub-forming species on the richly manured and petrel-turbated soils of that islet’s summit slopes (T. C. Greene and K. Baird pers. comm.). While accurate counts of the number of plants present on Haszard Islet were not made, it would seem that on both visits the islet summit supported approximately 20–50 individuals (T. C. Greene and K. Baird pers. comm.). Further south, its status on Curtis Island remains unknown, as there have been no gatherings of *Lepidium* from there since 1969, and several visits during the 1980s and in 2002 did not see it there (G.A. Taylor and T. C. Greene pers. comm.). However, on nearby Cheeseman Island, a 7-ha island (of which c.5.6 ha is vegetated) two plants were recorded in 2002 during a brief helicopter assisted 40 minute visit (T. C. Greene pers. comm.). Later, in 2011, nine adults and an estimated 1000 seedlings were noted on Cheeseman Island during a thorough botanical investigation of that island (de Lange 2012). Therefore, to the best of our knowledge there are between 20 and 50 adults on Hazard Islet and nine mature plants on Cheeseman Island, while the status of this species on Curtis Island and Macauley Island is uncertain. Therefore *Lepidium castellanum* qualifies as ‘Threatened/Nationally Critical’ either because the total number of plants in the wild is < 250 (criterion A(1) of Townsend et al. (2008)) or because the combined area of occupancy is < 1 ha (criterion A(3) of Townsend et al. 2008). To that assessment we add the qualifiers ‘DP’ (Data Poor) and ‘IE’ (Island Endemic). Data Poor (‘DP’) is recommended because of the lack of accurate counts of wild plants from Haszard Islet, absence of any recent survey for the species on Curtis Island, and lack of any trend data.

### Lepidium crassum sp. nov.

urn:lsid:ipni.org:names:77129256-1

http://species-id.net/wiki/Lepidium_crassum

*A L. oleraceo habitu temporali, caulibus ad caudicem lignosum emorientibus, foliis crasse coriaceis, distincte petiolatis, late ellipticis, ellipticis vel obovatis, apicibus obtusis vel truncatis, marginibus saepe duplicato-serratis, siliculis orbiculatis vel orbiculato-rhomboideis, et sequentia nucleotidorum DNA distinguenda*.

#### Holotype.

**Figure 33.**
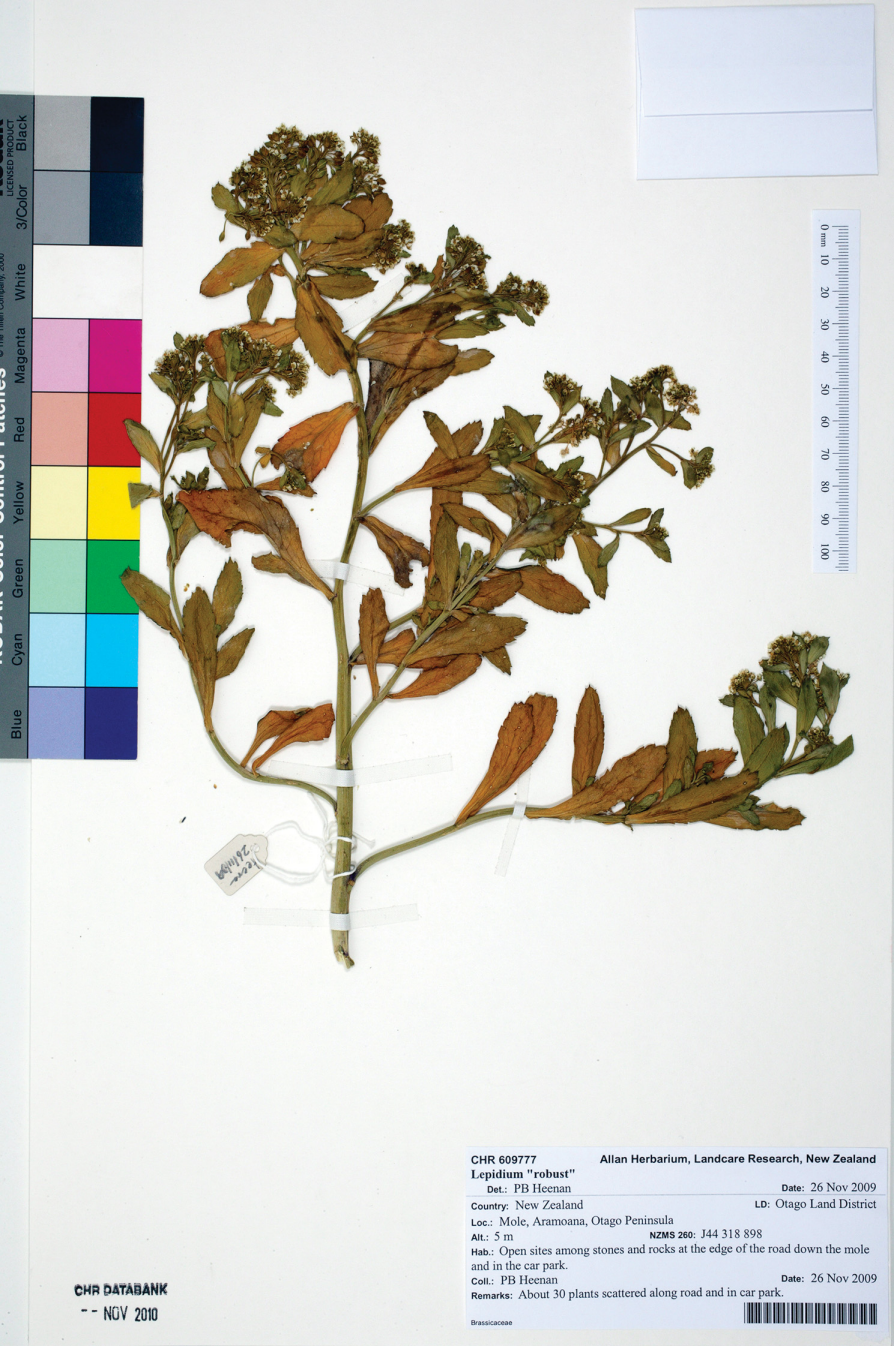
Holotype of *Lepidium crassum* Heenan et de Lange.

**New Zealand (Fig. 33):** Otago Land District, Otago Peninsula, Aramoana, Mole, open sites among stones and rocks at the edge of the road down mole and in car park, 26 November 2009, P. B. Heenan s.n., CHR 609777A! Isotype: CHR 609777B!

#### Etymology.

The specific epithet ‘*crassum*’ from the Latin for ‘thick’ refers to the distinctly thick and fleshy leaves of this species.

#### Description

**Figure 34.**
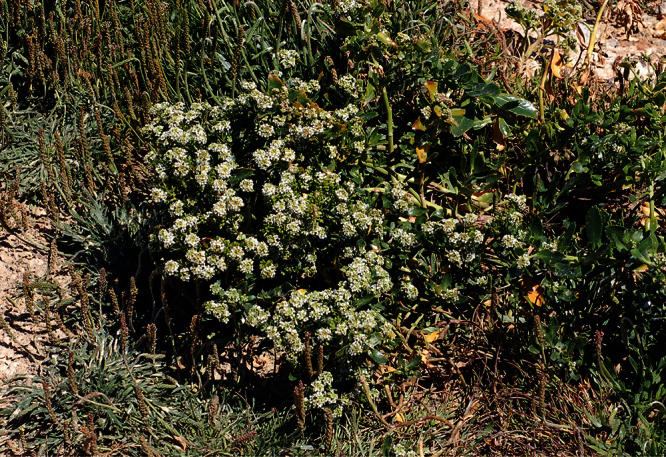
Wild plant of *Lepidium crassum* showing growth habit at Aramoana Mole.

**Figure 35.**
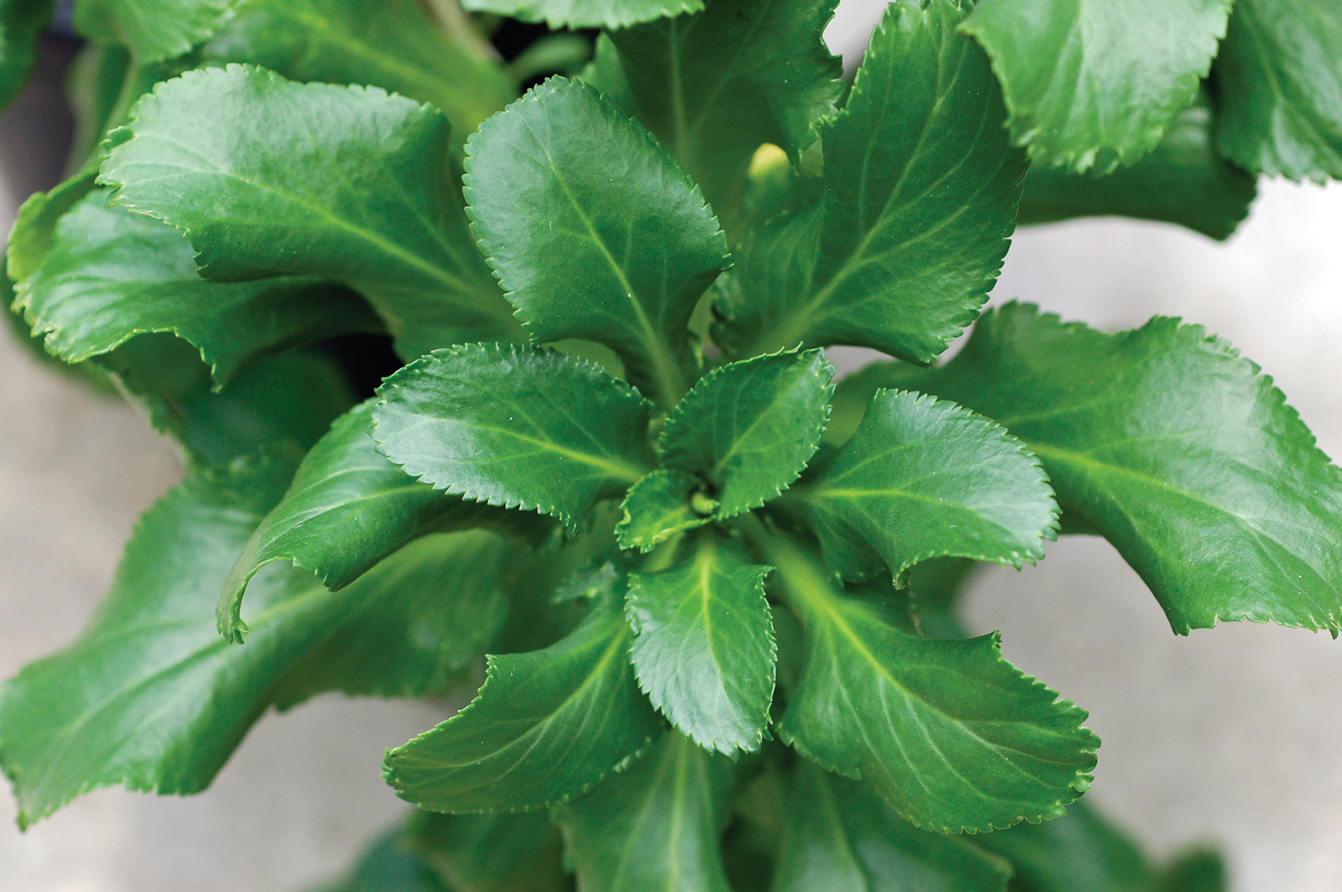
Leafy stem of *Lepidium crassum* showing basal leaves.

**Figure 36.**
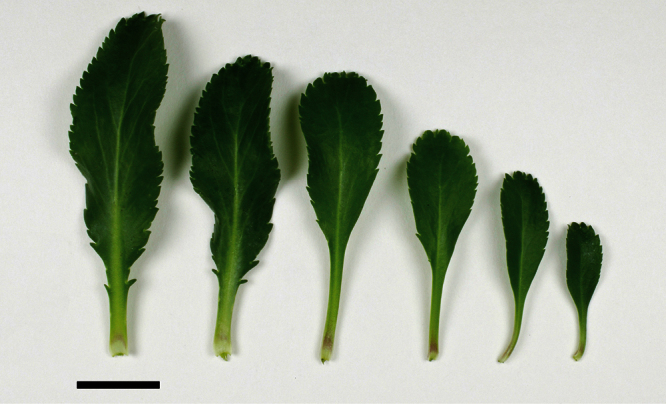
(From left to right) basal-, mid- to upper-stem leaves of *Lepidium crassum*. Scale bar = 20 mm.

**Figure 37.**
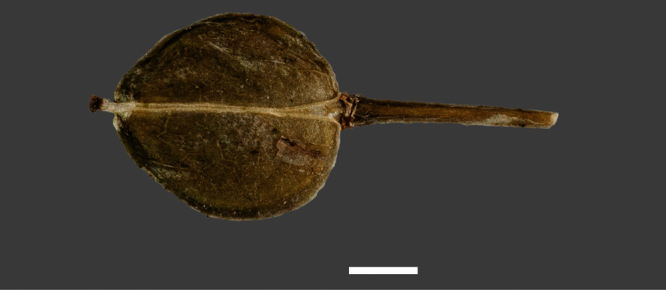
Mature silicle of *Lepidium crassum*. CHR 439956. Scale bar = 1 mm.

**(Figs 34–37).** Tap-rooted, strongly pungent smelling, perennial herb. Growth habit dense, stems closely placed, up to 50 cm tall, arising from underground woody stems. Stems upright to spreading, stout, short, rigid; mature stems woody, 100–400 × 10–12 mm, often devoid of foliage on middle and lower parts of stems, new stems 50–200 × 4–5 mm, leafy, glabrous. Leaves glabrous, coriaceous, green, undulate, rosette and stem leaves usually withering, variable in size and shape. Leaves of young and vigorous plants and stems: lamina 50–90 × 17–35 mm, broadly elliptic, elliptic to obovate; apex obtuse to truncate, often with up to 3 or 4 teeth; margin singly or doubly crenate, with 15–32 pairs of teeth; teeth up to 3.5 mm deep, sometimes overlapping, often protruding beyond leaf outline; base cuneate, petiole usually distinct; petiole up to 35.0 × 3.0–6.0 mm, channelled. Leaves of mature plants and cauline stems: lamina 15–45 × 6–15 mm, broadly elliptic, elliptic-obovate to obovate-oblong; apex obtuse to truncate, often with up to 3 or 4 teeth; margin singly crenate in upper half, teeth often protruding from leaf outline, with 5–10 pairs of teeth; teeth up to 1.2 mm deep, not overlapping, often protruding beyond leaf outline; base cuneate, sometimes narrowly so, usually tapering to ± distinct petiole; petiole 5–12 × 1.6–2.3 mm, channelled. Inflorescence terminal and lateral, racemose, 15–60 mm long, rachis 0.7–1.2 mm diam., glabrous; pedicels 4–7 mm long, erecto-patent, glabrous. Flowers 4.0–5.0 mm diam. Sepals 4, 1.3–1.6 mm long, saccate, overlapping at base, green, apex obtuse, margin white, shape dimorphic; lateral sepals broad, 1.4–1.5 mm diam., orbicular, abaxial surface often hairy, hairs 0.2–0.5 mm long; median sepals narrow, 1.0–1.3 mm diam., broadly elliptic, glabrous. Petals white, 1.8–2.0 × 1.0–1.1 mm, spreading, claw 0.6–0.8 mm long; limb broadly elliptic to orbicular, apex obtuse to rounded. Stamens 4; filaments 1.2–1.6 mm long, base 0.4–0.5 mm diam., equal; anthers 0.4–0.6 mm long. Ovary 1.0–1.6 × 0.9–1.1 mm, broadly ovate to broadly elliptic, green to green-brown, apex round or sometimes weakly shouldered; style 0.15–0.3 mm long, cylindrical; stigma 0.2–0.4 mm diam. Nectaries 4, 0.2–0.3 × 0.1–0.15 mm, oblong to oblong-triangular, green. Silicles cartilaginous when fresh, coriaceous when dry, 3.0–3.7 × 2.6–3.1 mm, orbicular to orbicular-rhomboid, apex obtuse to shallowly notched, valves pale brown,glabrous, not winged; style 0.2–0.3 mm long, exserted. Seeds 1.6–1.7 × 0.9–1.1 mm, narrowly ovoid, brown to orange-brown, not winged. FL Dec–Mar. FR Jan–Jul.

#### Representative Specimens.

**New Zealand (South Island)**: Oamaru, n.d., D. Petrie s.n., (WELT SP027632); Weston, North Otago, n.d., D. Petrie, s.n., (CHR 328313); south of Orore Point, North Otago, March 1982, T. R. Partridge s.n., (CHR 464024); Bridge Point, near Kakanui, North Otago, 13 February 1988, P. N. Johnson 751, (CHR 439956); Dunedin, February 1920, A. Wall s.n., (CHR 329217); Dunedin, St Leonards, 31 December 1896, B. C. Aston s.n., (WELT SP027641, SP027642); near Port Chalmers, Deborah Bay, March 1891, D. Petrie s.n., (WELT SP027633); Aramoana, The Mole, 13 November 1997, P. J. de Lange 3378 & J. W. Barkla, (AK 234312); Sandymount, Otago Peninsula, 12 May 1985, P. N. Johnson 300, (CHR 417850); Tairoa Head, Otago Peninsula, 27 March 1998, J. Barkla s.n. & L. Perriman, (CHR 579981); near mouth of Catlins River, n.d., D. Petrie s.n., (WELT SP027639); The Nuggets, South Otago, 5 Apr 1984, J. Ward s.n., (CHR 415751). South Otago, Waikawa Harbour, North Head, 9 February 1896, B. C. Aston s.n., (WELT SP027640); The Mole, Aramoana, 13 July 2009, P. B. Heenan s.n., (CHR 609772); Bridge Point, Orore Point, Waianakaru Road, 12 July 2009, P. B.Heenan s.n., CHR 609774. **Cultivated (New Zealand)**: Auckland Regional Botanic Gardens, ex. Dunedin, The Mole, 1 December 1996, J. Lord 5/96, (AK 231116); Landcare Research experimental nursery, Lincoln, ex Bridge Point, 10 July 2008, P. B. Heenan s.n., (CHR 609797); Landcare Research experimental nursery, Lincoln, ex Aramoana, 10 July 2008, P. B. Heenan s.n., (CHR 609800); Landcare Research experimental nursery, Lincoln, ex Wharekakahu Island, 10 July 2008, P. B. Heenan s.n., (CHR 609801); Landcare Research experimental nursery, Lincoln, ex Nugget Point, 30 March 2009, P. B. Heenan s.n., (CHR 609805); Landcare Research experimental nursery, Lincoln, ex Wharekakahu Island, 25 January 2010, P. B. Heenan s.n., (CHR 609814).

#### Distribution

**(Fig. 15)**. Endemic. New Zealand, South Island, Otago and Southland. *Lepidium crassum* once ranged from the Waitaki Valley (an inland location) and coastal locations at Oamaru to North Head, Waikawa Harbour in the south Catlins. Today, *Lepidium crassum* is most common on Otago Peninsula, but occurs in small populations from near Kakanui, North Otago to The Nuggets, South Otago.

#### Recognition.

*Lepidium crassum* differs from the related *Lepidium oleraceum* by its usually much smaller stature (Fig. 34) and seasonal growth habit (with plants dying back to a basal rosette overwinter). *Lepidium crassum* has distinctly petiolate, uniformly broadly elliptic, elliptic to obovate, thickly coriaceous, often doubly crenate leaves with obtuse to truncate apices (Figs 35, 36). Its silicles are usually orbicular, sometimes orbicular-rhomboid, and with obtuse to shallowly notched apices (Fig. 37) and by its rDNA ETS sequence.

#### Ecology.

Known from scattered populations that usually comprise fewer than 50 plants in a small geographic area. *Lepidium crassum* usually occurs on coastal headlands and rocky outcrops where it grows in disturbed open areas and among coastal herbfield of *Disphyma australe* subsp. *australe* and *Samolus repens* (J.R.Forst. et G.Forst.) Pers. var. *repens*. This species has also colonised the man made ‘Mole’ at Aramoana (Fig. 34) where it is can be common amongst rubble, concrete blocks and landfill.

#### Conservation Status.

*Lepidium crassum* is known from a small number of wild populations on the Otago coast. As most populations comprise few plants or occur on unstable coastal headlands and rocky outcrops, this species is vulnerable to stochastic events. We estimate there are between 250 and 1000 plants and the largest population comprises fewer than 300 plants. Using the New Zealand Threat Classification System (Townsend et al. 2008), *Lepidium crassum* qualifies as ‘Threatened/Nationally Endangered’ (criterion B(1/1). We recommend appending the qualifiers ‘CD’ (Conservation Dependent – because there have been several populations established by the Department of Conservation and these are being actively managed), ‘DP’ (Data Poor – to reflect uncertainty over plant numbers), ‘EF’ (Extreme Fluctuations – because the largest known monitored population, that on Wharekakahu Island, appears to naturally undergo huge and apparently natural population fluctuations (J. W. Barkla pers. comm.)), and ‘RR’ (Range Restricted – because it is naturally confined to a geographically small part of the New Zealand Botanical Region).

### Lepidium flexicaule

Kirk, Trans. et Proc. New Zealand Inst. 14, 380 (1882)

http://species-id.net/wiki/Lepidium_flexicaule

#### Lectotype.

**New Zealand (North Island) (*fide*Garnock-Jones and Norton 1995):** Onehunga, n.d. T.Kirk 341 WELT SP030080! Isolectotypes: WELT SP030089!, WELT SP027620!

#### Etymology.

The exact meaning of the species ‘*flexicaule*’ was not given by Kirk (1899) but the translation ‘flexuous stems’ accurately characterises the plant.

= *Lepidium incisum* Banks et Sol. ex Hook.f., *Flora Novae-Zelandiae 1*, 15 (1853) *nom. illeg*., non Roth, *Neue Beytr. Bot. 1*: 224 (1802), nec Edgew., *Trans. Linn. Soc. 20*, 33 (1851)

#### Lectotype.

**New Zealand (North Island) (*fide*Garnock-Jones and Norton 1995):** Opuraga, on Beach, rare, n.d., Banks and Solander, BM! Isolectotypes: AK 100095!, WELT SP063696a, b!

#### Etymology.

Banks and Solander evidently chose the epithet ‘*incisum*’ in allusion to the deeply incised leaves of their specimens.

#### Description

**Figure 38.**
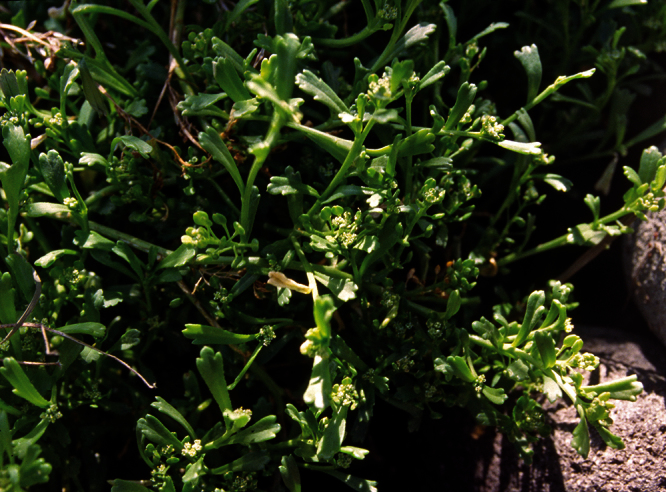
Wild plant of *Lepidium flexicaule* growing on cobble beach showing decumbent growth habit (image: C.C. Ogle).

**Figure 39.**
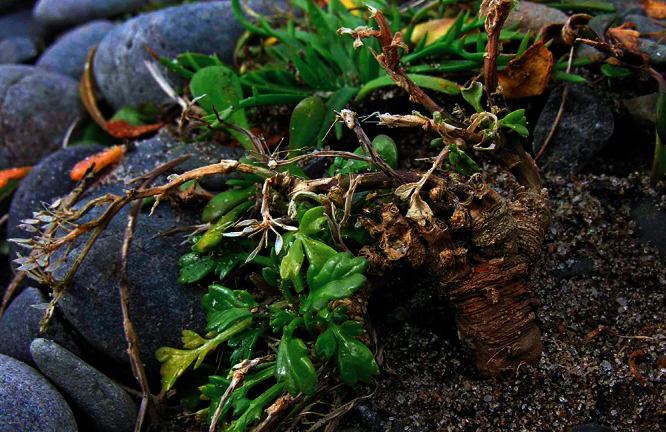
Mature plant of *Lepidium flexicaule* with exposed root stock showing persistent past season’s stems and new season’s growth (image: S. Walls).

**Figure 40.**
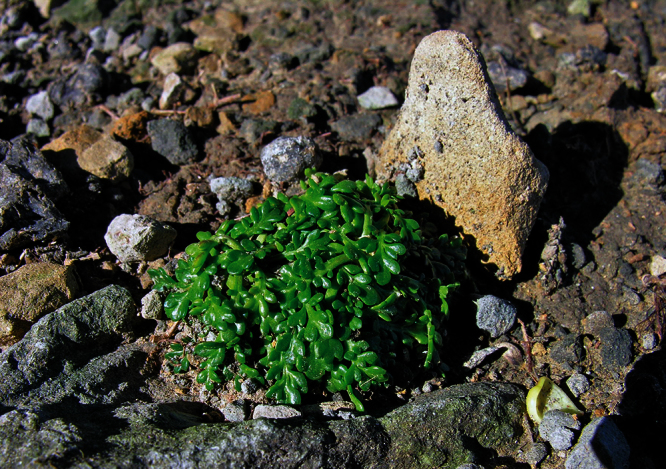
*Lepidium flexicaule* plant at rosette stage of growth (early in growing season) (image: S. Walls).

**Figure 41.**
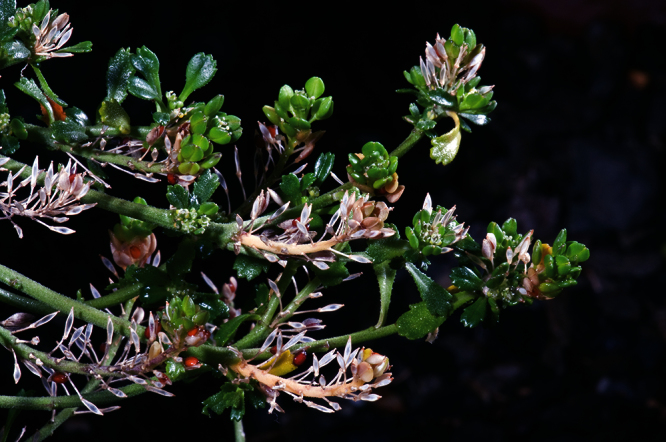
Old and new inflorescences of *Lepidium flexicaule*.

**Figure 42.**
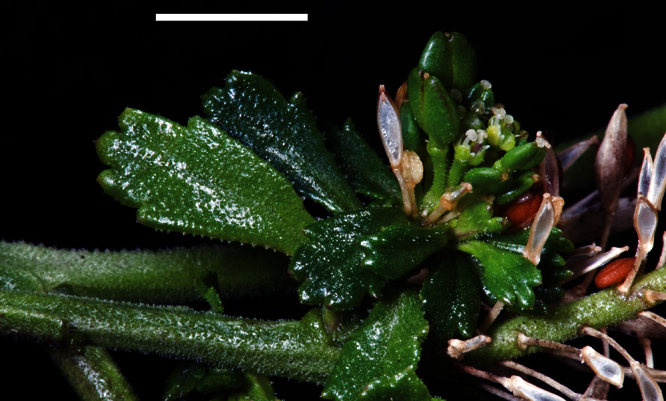
Close up of stems, upper-stem leaves and inflorescences showing denticles of *Lepidium flexicaule*. Scale bar = 5 mm.

**Figure 43.**
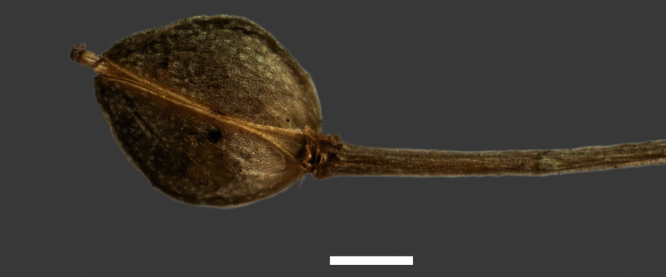
Mature silicle of *Lepidium flexicaule*. AK 294940. Scale bar = 1 mm.

**(Figs 38–43)**. Tap-rooted, strongly pungent smelling, decumbent, summer-green, perennial herb forming densely leafy, patches up to 1.5 m diam., and arising from stout, semi-circular, whitish-grey (when exposed) rootstock 3–10 mm diam. when fresh. Tap root fleshy, yellow to yellow-white when fresh, up to 300 mm long, deeply descending. Plants dying down in winter or in times of adversity to rootstock (in well-grown individuals the new season’s branchlets may die back to the previous season’s stem nodes). Stems prostate, weakly flexuous, divergent to widely spreading, 100–800 mm long, 0.8–5.2 mm diam., woody near base, initially spherical in cross-section, pale yellow-green to dark green, sometimes tinged maroon and finely puberulent with short papillate or tapered hairs; becoming glabrous and prominently ridged and/or grooved with age, and usually bearing numerous leaf scars and withered petioles; upper portion of stems sparingly and openly to heavily branched; branches and branchlets, usually very leafy. Leaves glabrous, firmly fleshy to succulent, glossy dark green to yellow-green, at senescence turning yellow. Rosette and lower stem leaves withering at fruiting; petioles distinct 10–50 × 1–3 mm, slightly concave in cross-section, fleshy; leaves glabrous above, with papillae or denticles along midrib beneath and margins in New Zealand plants, or without denticles (absent in Chatham Islands and most Australian plants); lamina 50–90 × 15–30 mm, pinnatifid, obovate to oblanceolate; pinnae in 1–3 pairs, bluntly toothed or crenate at apex and distal margins. Upper stem leaves glabrous above, usually with triangular denticles, dense on margins and sparse to dense beneath (although absent in Chatham Islands and most Australian plants); mostly apetiolate, petiole if present minute up to 1.5 mm long; lamina 5–30 × 3–10 mm, obovate, oblanceolate, or spathulate, apex bluntly toothed to crenate, base broadly to narrowly cuneate. Racemes 10–50 mm long, elongating up to 60 mm at fruiting, terminal and leaf-opposed; rachis and pedicels puberulent with short tapering hairs, and ± covered in triangular denticles (absent in Chatham Islands and most Australian plants) or glabrous; axillary; rachis and pedicels hairs if present, retrorse to patent, very short, 0.05–0.8 mm long, ± clavate, eglandular – glandular; pedicels, erecto-patent to patent, initially 1.04–1.27(–2.38) mm; elongating to 2.34–5.00(–6.02) mm long at fruiting. Flower buds dark green, apex bearing a conspicuous, caducous, crest of white, eglandular, antrorse hairs up to 0.9 mm long. Flowers sweetly fragrant, 1.4–1.8(–2.3) mm diam. Sepals, broadly ovate to oval, apex broadly obtuse, centrally green with a white margin, deeply concave, adaxially weakly keeled, adaxial midrib invested in conspicuous, caducous, white, eglandular, antrorse, hispid hairs, hairs sometimes scattered across rest of adaxial surface; abaxial surface glabrous; lateral sepals broad, 0.6–1.0 × 0.6–1.2 mm , median sepals narrower 0.4–0.8 × 0.4–0.7 mm. Petals white, 0.3–0.8(–1.0) × 0.2–0.8 mm, erecto-patent or patent, clawed; limb broadly obovate, apex obtuse, retuse or distinctly emarginate. Stamens 2, equal. Anthers c.0.16 mm long. Pollen bright yellow. Nectaries 4, subulate, 0.40 mm long. Silicles cartilaginous when fresh, subcoriaceous when dry, (2.1–)3.3(–4.1) × (2.2–)3.3–3.4(–4.0) mm, orbicular, obovate, to ovate, slightly winged in upper ¼, apex scarcely or shallowly notched, valves green maturing yellow-green, glabrous; style 0.8 (–1.0) mm long, free from the narrow wing, equal to or slightly exceeding the notch; stigma 0.2–0.4 mm diam. Seeds 2, 1.20–1.38 × 0.80–1.10 mm, ovoid to suborbicular, red-brown, dark red-brown or brownish black, not winged. FL. Oct–Feb. FR. Dec–Apr.

#### Representative Specimens.

**Australia (Tasmania)**: Port Davey, Gull Reef, January 1977, M. Allan s.n., (HO 26460); Port Davey, Elliot Point, 20 January 1986, A. Moscal 11876, (HO 402077); 1 km South of Rheuben Creek, 22 February 1985, A. Moscal 9734, (HO 401030); 1 km South of Nye Bay, 6 February 1986, A. M. Buchanan 8210, (HO 98828); Endeavour Bay, 30 January 1984, A. M. Moscal 6017, (HO 74481); Hibbs Bay, 25 January 1984, A. M. Buchanan 2775, (HO 74480); Bruny Island, n.d. L. Rodway s.n., (HO 54049). **New Zealand (North Island):** Great Barrier (Aotea island), Saddle Island, 7 January 1989, E. K. Cameron 5295, (AK 206897, AD, CHR).North Auckland, Waitemata, North Head, n.d., T. Kirk 342,(WELT SP030081); Waitemata, T. Kirk 41, n.d., WELT SP030088 (Duplicate AK 11429); Waitakere, May 1885, Ball s.n., (AK 261800); Bethell’s [Beach], 7 Jan 1934, L. M. Cranwell s.n., (AK 100102); Hauraki Gulf, Rangitoto Island, December 1882, T. F. Cheeseman s.n., (AK 4481); South Auckland, Manukau Harbour, Mangere, n.d., T. F.Cheeseman s.n., (AK 4480); Piako, n.d. J. Adams s.n., (AK 14865, AK 14866); Taranaki, Stent Road, 26 Jan 2010, P. J. de Lange 9279 & G. M. Crowcroft, (AK 317033), Kapiti Island, n.d. J. Buchanan s.n., (WELT SP087437). **New Zealand (South Island):** Between Little Whanganui River and Mokihinui, n.d., ?W. L. Townson 24, (WELT SP044035); Vicinity of Westport, 1903, W. Townson s.n., (WELT SP030082, WELT SP030084); Westport, Orowaiti River Bank, n.d., W. L. Townson s.n., (AK 253094); Westport, Orowaiti River Bank, 31 January 1953, R. Mason & N. T. Moar 2163, (AK 225200, CHR 81618); Cape Foulwind, n.d., W. Townson s.n., (WELT SP30085); Cape Foulwind, 4 February 1913, D. Petrie s.n., (WELT SP30086); Cape Foulwind, Tauranga Bay (Seal Colony), 14 August 1992, P. J. de Lange 1478 & D. A. Norton, (WELT SP079914); Punakaiki, 18 January 1931, L. B. Moore s.n. & L. M. Cranwell, (AK 100103). **Chatham Islands**: Rekohu, Point Somes, 25 January 2005, I. Keenan s.n., (AK 289897); Rekohu, Zimmermans Property, Point Somes, 10 January 2006, P. J. de Lange CH392, (AK 294940, CANB, CHR); Rekohu, Zimmermans Property, Point Somes, 10 January 2006, P. J. de Lange CH395, (AK 294940, CANB, CHR, WAIK); Rekohu, Ocean Bay, Unnamed Point south west of Bay, 14 January 2006, P. J. de Lange CH440 & J. W. D. Sawyer, (AK 295121); Rekohu, Wharekauri Farm Station, Cape Young, 13 January 2006, P. J. de Lange CH425 & J. W. D. Sawyer, (AK 295154).

#### Distribution

**Figure 44.**
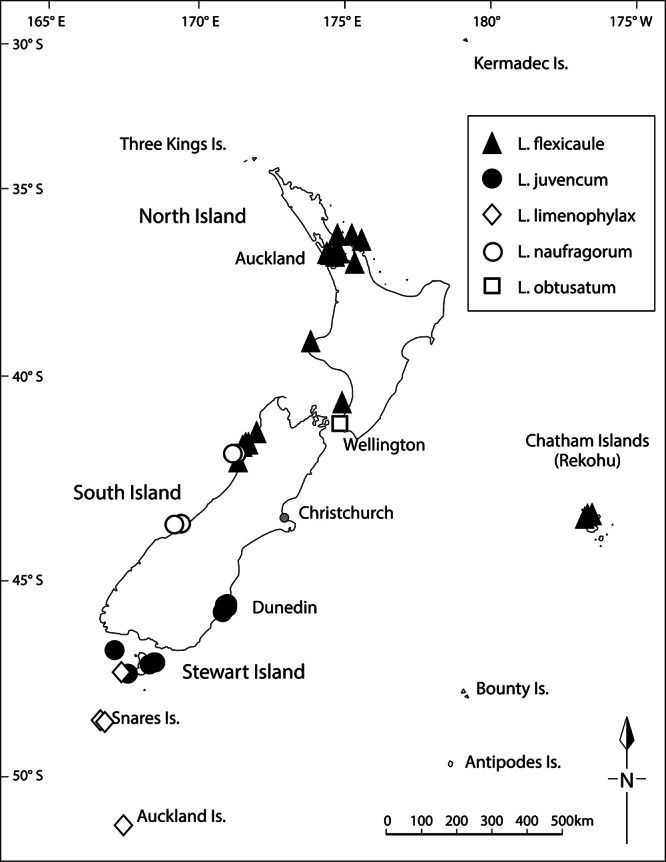
Distribution of *Lepidium flexicaule* (New Zealand distribution shown only), *Lepidium juvencum*, *Lepidium limenophylax*, *Lepidium naufragorum* and *Lepidium obtusatum*.

**(Fig. 44)**. Indigenous. Australia (Tasmania) and New Zealand (North, South, Chatham islands). In the North Island of New Zealand, *Lepidium flexicaule* is now known from only two extant populations, one on Saddle Island off the west coast of Great Barrier Island (Aotea Island) (de Lange and Cameron 2011) and the other at Stent Road north of Cape Egmont, Taranaki (Peet et al. 2003). Historically *Lepidium flexicaule* was present on Rangitoto Island, along the Waitakere Coastline, near North Head, Waitemata Harbour and near Onehunga, Manukau Harbour (Kirk 1899; Cheeseman 1906, 1925; Garnock-Jones and Norton 1995). It was also recorded from the Firth of Thames, at Mercury Bay (Kirk 1899; Cheeseman 1906, 1925; Garnock-Jones and Norton 1995) and near Wellington (Garnock-Jones and Norton 1995). In the South Island, the species is confined to the western coast of North-West Nelson from near Kaihoka to about Point Elizabeth. *Lepidium flexicaule* was discovered on the northern portion of Rekohu in 2005 by Department of Conservation botanists.

#### Recognition.

*Lepidium flexicaule* is easily distinguished from all other indigenous *Lepidium* species by the combination of having a decumbent growth habit, pinnatifid rosette and basal stem leaves (Figs 38–41), and flowers with two stamens. Most North Island and South Island populations are further distinguished by the presence of triangular denticles on branchlet stems, leaf margins and petioles, and on the rachis and pedicels of the inflorescences (Fig. 42, see also Garnock-Jones 1988). Denticles are, however, absent from the Chatham Islands, most Australian collections, and some South Island ones. The absence of denticles prompted the brief listing of Rekohu and Australian plants as an unnamed subspecies (see de Lange et al. 2009). However, as denticles are sometimes absent from South Island plants, formal taxonomic distinction may not be warranted. Nevertheless, it is worth noting that Rekohu plants are consistently larger than those seen from the rest of this species’ range, with well-grown specimens reaching up to 1.5 m diam., and that cpDNA data separates Tasmanian and Rekohu *Lepidium flexicaule* from New Zealand samples (Fig. 4). Further study, including obtaining sequences of non-denticulate South Island *Lepidium flexicaule*, is warranted. Of the indigenous species, it is perhaps most similar to *Lepidium naufragorum*, which differs by its upright bushy shrub-forming habit with ascending to erect stems (Fig. 55), more sharply serrated pinnae of the leaves; racemes that are not so clearly leaf-opposed and are much longer (up to 150 mm, cf. 50 mm long), flowers that have four rather than two stamens, and emarginate petals that are longer than the sepals (see Garnock-Jones and Norton (1995) for other distinctions).

*Lepidium flexicaule* is however, commonly confused in the field with the naturalised *Lepidium didymum* L. and *Lepidium coronopus* (L.) Al-Shehbaz, species with which it sometimes grows. Both *Lepidium didymum* and *Lepidium coronopus* have long been treated in New Zealand under the segregate genus *Coronopus* but Al-Shehbaz et al. (2002) reinstated both species within *Lepidium*. *Lepidium flexicaule* can be distinguished from *Lepidium didymum* and *Lepidium coronopus* by the valves of the silicles, which are smooth and which dehisce into 2 valves to leave a persistent replum. The silicles of *Lepidium didymum* and *Lepidium coronopus* have a reticulate-ridged or warty surface, and do not dehisce along the valves; in *Lepidium didymum* they split into 1-seeded segments and in *Lepidium coronopus* they are indehiscent.

#### Ecology.

The ecology of *Lepidium flexicaule* was described in some detail by Garnock-Jones and Norton (1995). They concluded that it is a strictly coastal species of turf communities, rock crevices and the strandline of bouldery beaches. *Lepidium flexicaule* has also colonised tracksides, the compacted peaty ground of seal colonies, bird roosts, and even bird nests, where plants presumably arose from fruiting material that had been used for nest construction. In most locations, the species is found within the spray zone and always in sites prone to frequent disturbance.

#### Conservation Status.

Previously *Lepidium flexicaule* had been assessed ‘Threatened / Nationally Vulnerable _CD, EF_’ (de Lange et al. 2009). Based on current evidence, this ranking is no longer appropriate, as Rekohu populations of this species (treated in that paper as an unnamed entity *Lepidium* aff. *flexicaule* but now included in *Lepidium flexicaule* (see recognition above)) and all of the monitored New Zealand *Lepidium flexicaule* populations are in decline, while the past total area of occupancy had been grossly under estimated. Based on current knowledge, *Lepidium flexicaule* is more appropriately assessed as ‘Threatened / Nationally Endangered’ (criterion A(3/1) of Townsend et al. (2008)) because the total area of occupancy of the sum of the populations is < 10 ha, and evidence obtained from monitoring indicates that there is an ongoing decline predicted to be up to 50% over the next ten years due to increased plant mortality (causes of which are as yet unknown) and loss of habitat caused by competition from weeds and coastal erosion. To this assessment we append the qualifiers ‘CD’ (Conservation Dependent – because virtually all known populations are being managed), ‘EF’ (Extreme Fluctuation – because monitoring also indicates that this is a species that, despite the decline, is naturally prone to seasonal population fluctuations) and ‘TO’ (Threatened Overseas – because, for the time being, we include Rekohu and Tasmanian plants within *Lepidium flexicaule* (see recognition), and in Tasmania *Lepidium flexicaule* is also seriously threatened (A. Buchanan pers. comm.)).

#### Hybridism.

On the Chatham Islands, *Lepidium flexicaule* is occasionally found growing with *Lepidium oligodontum* and *Lepidium rekohuense*, and putative hybrids have been collected when these species occur together. There is good evidence for the hybrid *Lepidium flexicaule* × *Lepidium oligodontum* (represented by AK 294939, *P. J. de Lange CH391*; AK 294942, *P. J. de Lange CH394*; AK 295119, *P. J. de Lange CH442 & J. W. D. Sawyer*). This hybrid is known from two sites on Chatham (Rekohu) Island where it is found in association with both parents. Like the parents, the hybrids have a decumbent growth habit. They share with *Lepidium flexicaule* pinnatifid rosette leaves, but vegetative features of *Lepidium oligodontum* are evident in the mid to upper stem leaves that are mostly spathulate to cuneiform (but with occasional deeply lobed margins to weakly pinnatifid leaves that are similar to *Lepidium flexicaule*). The flowers of the hybrids have variable stamen numbers ranging from 1–5 per flower, compared to 2 in *Lepidium flexicaule* and 2–4–6 in *Lepidium oligodontum*, while pollen stainability varied from 80 to 85%. Seedlings raised from AK 295119 presented a bewildering array of foliage types grading into either parent. Unfortunately, through mishap these plants failed to reach maturity, so vouchers showing this are unavailable.

Evidence for *Lepidium flexicaule* × *Lepidium rekohuense* is less convincing. This putative hybrid is known from only one gathering (AK 295155, *P. J. de Lange CH426 & J. W. D. Sawyer*) which was collected in January 2006 from Cape Young, Rekohu, at a site where both parents grew. In the field, this specimen resembled *Lepidium flexicaule* closely except that the plant was distinctly leafy and the upper stem foliage was more copious and larger than is usual for *Lepidium flexicaule*. As with all Rekohu gatherings of *Lepidium flexicaule*, this specimen lacked stem and marginal leaf denticles. Furthermore, as with both parents, there are two stamens per flower, and pollen stainability of this gathering was 98%. No fruiting material was present, and on a subsequent search of this site in 2007 the putative hybrid and its parents had gone. This specimen may be a very well grown example of *Lepidium flexicaule* but, as it appears anomalous alongside other Rekohu, New Zealand, and Australian gatherings of that species, we prefer to treat it as a putative *Lepidium flexicaule* × *Lepidium rekohuense* hybrid.

The putative hybrid *Lepidium flexicaule* × *Lepidium oleraceum* has also been recorded once, as a spontaneous plant arising in cultivation at the Auckland Regional Council Botanic Gardens (AK 228296 S. P. Benham s.n., AK 223492 S. P. Benham s.n.). The hybrid grew in a site where both parents (Scots Beach *Lepidium flexicaule* and Stephens Island *Lepidium oleraceum*) were planted together. The hybrid appeared from a seed lot gathered from the cultivated *Lepidium flexicaule* plant. It initially appeared to be different because it was much larger and more vigorous than *Lepidium flexicaule*, which it otherwise closely resembled (S. P. Benham pers. comm.). Over time, the hybrid developed weakly ascendant branches with rhomboid deeply serrated to pinnatifid mid and upper stem leaves, and flowers with 1–6 stamens. The hybrid appeared to be fully fertile, and seedlings raised from it showed clear segregation back to either parent (S. P. Benham pers. comm.). Again through mishap, these plants were lost before they reached maturity and specimens could be taken.

### Lepidium juvencum sp. nov.

urn:lsid:ipni.org:names:77129257-1

http://species-id.net/wiki/Lepidium_juvencum

*A L. oleraceo habitu decumbente vel effuso patuloque, caulibus sparsis effusis gracilibus flexilibus, foliis subcoriaceis, apice obtuso vel truncato, plerumque 2--3 dentibus prominentibus ut videtur irregularibus, floribus fructibusque e plantis juvenibus prodientibus, et propria sequentia nucleotidorum DNA distinguenda*.

#### Holotype.

**Figure 45.**
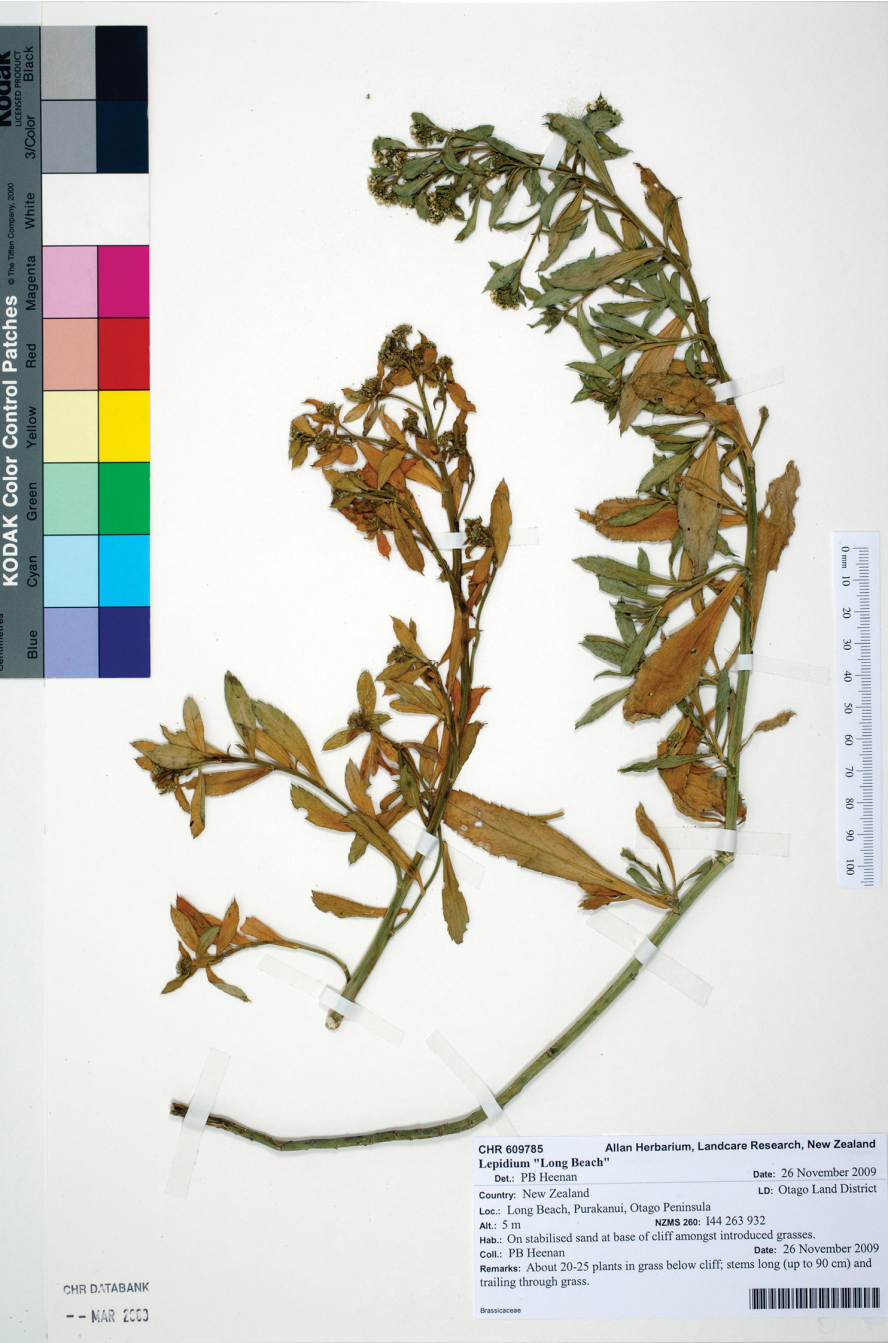
Holotype of *Lepidium juvencum* Heenan et de Lange.

**New Zealand (Fig. 45):** Otago Land District, Otago Peninsula, Long Beach, Purakanui, on stabilised sand at base of cliff amongst introdued grasses, 26 November 2009, P. B. Heenan s.n., CHR 609785B! Isotype CHR 609785A!

#### Etymology.

The specific epithet ‘*juvencum*’ from the Latin for ‘*youthful*’ refers to plants flowering and fruiting only a few months after germinating from seed.

#### Description

**Figure 46.**
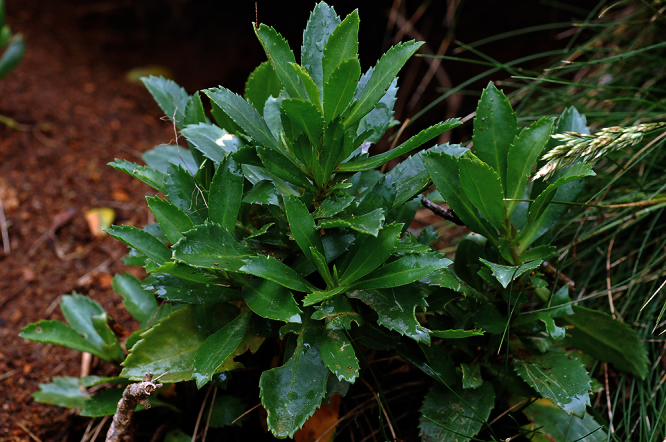
Young plant of *Lepidium juvencum* growing with *Poa astonii* near shearwater burrows.

**Figure 47.**
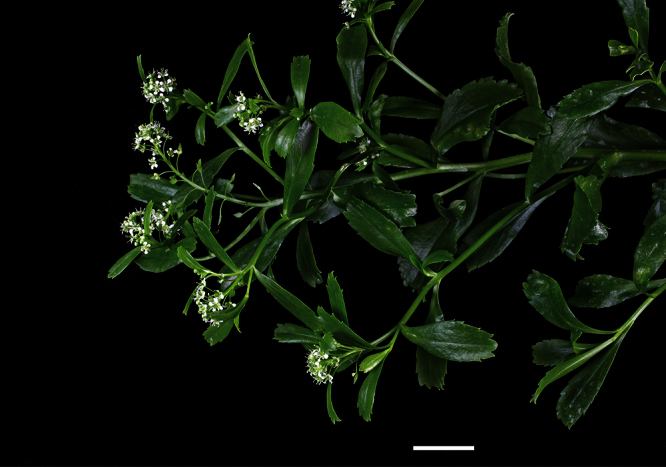
Flowering stem of *Lepidium juvencum* showing multiple inflorescences and trailing growth habit. Scale bar = 20 mm.

**Figure 48.**
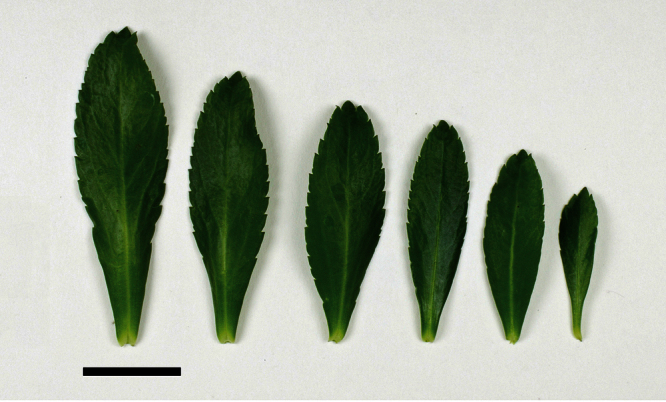
(From left to right) basal-, mid- and upper-stem leaves of *Lepidium juvencum*. Scale bar = 20 mm.

**Figure 49.**
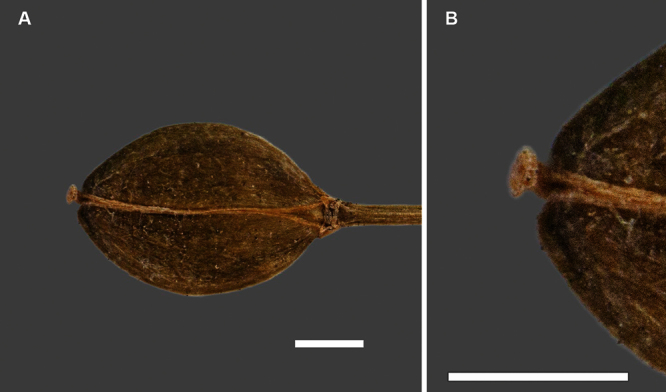
(**A**) Mature silicle of *Lepidium juvencum*; (**B**) close up of silicle apex showing slight notch. AK 297694. Scale bars = 1 mm.

**(Figs 46–49).** Tap-rooted, strongly pungent smelling, perennial herb. Growth habit open, straggly, up to 50 cm tall. Stems usually decumbent to sprawling, slender, flexible, sparse; mature stems woody, 100–1000 × 8–12 mm, often devoid of foliage on middle and lower parts of stems; new stems 100–400 × 3–4 mm, leafy, glabrous. Leaves glabrous, subcoriaceous, green, often undulate, rosette and stem leaves usually withering, variable in size and shape. Leaves of young and vigorous plants and stems: lamina 37–87 × 12–32 mm, elliptic, obovate or elliptic-oblanceolate; apex truncate to obtuse, usually with 2–3 prominent teeth and often appearing irregular; margin singly crenate, with 4–19 pairs of teeth; teeth up to 1.5 mm deep, not overlapping; base attenuate to cuneate, tapering to a distinct or indistinct petiole; petiole up to 23.0 × 2.0–5.0 mm, or sessile. Leaves of mature plants and cauline stems: lamina 10–60 × 3–21 mm, elliptic, elliptic-oblanceolate, obovate to elliptic-obovate; apex subacute, truncate or obtuse, usually with 2–3 prominent teeth and often appearing irregular; margin singly crenate in upper and/or lower half, with 4–19 pairs of teeth; teeth up to 1.3 mm deep, not overlapping; base attenuate to cuneate, tapering to distinct or indistinct petiole, or sessile. Inflorescence terminal and lateral, racemose, 10–60 mm long, rachis 1.0–1.3 mm diam., glabrous; pedicels 4.0–6.0 mm long, erecto-patent, usually glabrous although lower pedicels occasionally sparsely hairy on adaxial surface. Flowers 4.0–5.0 mm diam. Sepals 4, 1.3–1.5 mm long, saccate, overlapping at base, green, apex obtuse, margin white, shape dimorphic; lateral sepals broad, 1.1–1.5 mm diam., orbicular, abaxial surface often hairy, hairs 0.1–0.5 mm long; median sepals narrow, 0.9–1.2 mm diam., broadly elliptic, glabrous. Petals white, 2.0–2.4 × 1.1–1.5 mm, spreading, claw 0.6–1.0 mm long; limb broadly elliptic to orbicular, apex obtuse to rounded. Stamens 4(–5); filaments 1.4–1.7 mm long, base 0.3–0.5 mm diam., equal; anthers 0.3–0.4 mm long. Ovary 1.0–1.5 × 0.9–1.4 mm, broadly ovate to broadly elliptic, green to green-brown, apex usually with shoulders; style 0.15–0.25 mm long, cylindrical; stigma 0.3–0.5 mm diam. Nectaries 4, 0.2–0.3 × c. 0.1 mm, oblong-obovate, green. Silicles cartilaginous when fresh, coriaceous when dry, 3.1–4.2 × 2.5–3.5 mm, elliptic-rhomboid to orbicular-rhomboid, valves light brown,glabrous, apex shallowly notched, not winged; style 0.2–0.3 mm long, exserted. Seeds 1.6–1.8 × 0.9–1.3 mm, narrowly ovoid, brown to orange-brown, not winged. FL Nov–May. FR Nov–Jun.

#### Representative Specimens.

**New Zealand (South Island):** Otago, Long Beach, 30 December 1998, G. S. Stone s.n., (AK 239841); Otago, Long Beach, 25 Jun 1997, D. Nelson s.n. & J. Barkla, (CHR 518601); Otago, Long Beach, 13 July 2009, P. B. Heenan s.n., (CHR 609779);Otago,Long Beach, 14 July 2009, P. B. Heenan s.n., (CHR 609780); Tomahawk Beach, 4 February [1920?], B. C. Aston s.n., (WELT SP027651, WELT SP052362); Tavora Reserve, 13 July 2009, P. B. Heenan s.n., (CHR 609786–609789);Otago Peninsula,Green Island, January 1955, R. A.. Falla s.n., (WELT SP038744); Otago Peninsula, Green Island, January 1957, M. E. Gillham s.n., (CHR 111452); Otago Peninsula, Green Island, 6 July 1983, P. N. Johnson 70, (CHR 404351); Otago Peninsula, Green Island, 27 March 1989, P. N. Johnson 823, (AK 185642, CHR 461272); Otago Peninsula, Green Island, 12 February 1999, G. Loh s.n., (AK 238646); Solander Island, 10 November 1973, P. N. Johnson s.n., (CHR 253066). **New Zealand (Stewart Island/Rakiura):** Halfmoon Bay, Mill Bay, 4 February 1963, P. Hynes s.n., (AK 92212); Womans Island, January 1976, P. N. Johnson s.n., (CHR 261892); Herekopare Island, 20 May 1959, E. A. Willa s.n., (WELT SP050477, AK 249215); Herekopare Island, 4 January 2010, P. B. Heenan s.n, (CHR 609808–609810); Mutton Bird (Titi) Islands, Big Moggy Islands, December 2002, B. D. Rance s.n., (AK 293254). **Cultivated (New Zealand):** Lincoln, ex Herekopare Island, 2 Feb 1980, H. D. Wilson 798-314, (CHR 368843); Lincoln, ex Long Beach, Landcare Research experimental nursery, 10 July 2008, P. B. Heenan s.n., (CHR 609803).

#### Distribution

**(Fig. 44).** Endemic. New Zealand. South and Stewart Island/Rakiura. Known from Otago and islands off the coast of Stewart Island/Rakiura *Lepidium juvencum* is currently known from just three locations: Long Beach and Green Island (Otago Peninsula) and Herekopare Island (east of Stewart Island/Rakiura). However, it is also probably present on some of the south-western Titi Island (south-west of Stewart Island/Rakiura) (B.D. Rance pers. comm.) and it is still likely to occur elsewhere, especially on rat free islands in the vicinity of Stewart Island, as is evidenced by specimens previously collected from Womans (CHR 261892) and Solander (CHR 253066) islands in the 1960s and 1970s.

#### Recognition.

*Lepidium juvencum* is distinguished by open, sprawling, straggling growth habit, and the stems are often trailing on the ground (Figs 46, 47). The leaves are elliptic, elliptic-oblanceolate, obovate to elliptic-obovate, and have attenuate bases; the marginal teeth are usually small and are mostly confined to the distal third of the leaf (Fig. 48). The silicles are most like *Lepidium oleraceum* but differ by possessing a rounded, slightly notched apex (Fig. 49).

#### Ecology.

*Lepidium juvencum* grows in open and often disturbed sites on sandy soil. At Long Beach it grows on stabilised sand at the base of a cliff immediately behind the beach, and at Herekopere Island it occurred on a steep coastal bank comprising sandy loam. On Kaimohu Island, in the south-western Titi group, what is probably *Lepidium juvencum* has been recorded growing with *Lepidium limenophylax* on the margins of coastal scrub. *Lepidium juvencum* has also been established and is self-seeding at the Yellow-eyed Penguin Trust revegetation project at Tavora Reserve (near Palmerston, North Otago). Here it grows among *Euphorbia glauca* G.Forst. and *Ficinia spiralis* (A.Rich.) Muasya et de Lange on sand dunes at the back of the beach.

#### Conservation Status.

*Lepidium juvencum* is presently known from three wild localities, and, while the number of plants at these localities is not known with certainty, it is estimated that there are < 100 in total. It is also acknowledged that *Lepidium juvencum* is likely to be more widespread, especially in the vicinity of Stewart Island/Rakiura, and further field surveys are required. Recruitment is likely to be sporadic, and the small and sparse populations are especially vulnerable to stochastic events, such as storm surges, that could erode its habitat. Based on our current knowledge of this species and using the New Zealand Threat Classification System (Townsend et al. 2008), *Lepidium juvencum* qualifies as ‘Threatened/Nationally Critical’ (either criterion A(1) or A(3) of Townsend et al. (2008) applies). We recommend appending the qualifiers, ‘CD’ (Conservation Dependent – because the species has been deliberately established at Tavora Reserve, where it is actively managed), ‘DP’ (Data Poor – to reflect uncertainty over its distribution), and ‘RR’ (Range Restricted – because it is naturally confined to a geographically small part of the New Zealand Botanical Region).

### Lepidium limenophylax sp. nov.

urn:lsid:ipni.org:names:77129258-1

http://species-id.net/wiki/Lepidium_limenophylax

*A L. oleraceo G.Forst. habitu constanter decumbenti vel procumbenti, nodis caulorum ligneis repullantibus tempo novo et interdum radicibus adventiis, foliis constanter angusto-lanceolatis vel linearibus serrulatis floribus staminibus duobus, et DNA sequentiis singularibus differt*.

#### Holotype.

**Figure 50.**
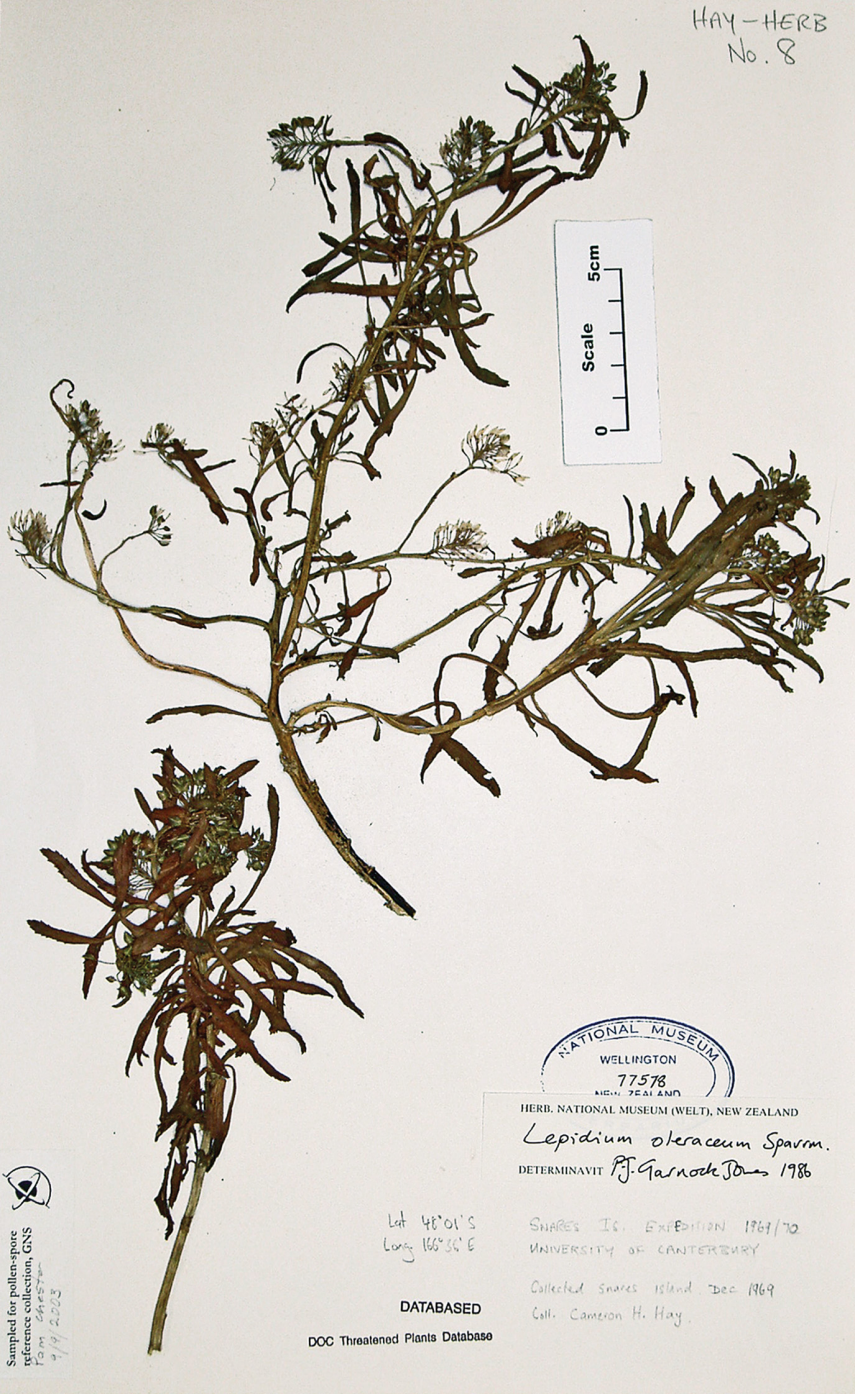
Holotype of *Lepidium limenophylax* de Lange, B.D.Rance et D.A.Norton.

**New Zealand (Fig. 50):** Snares Island, Snares Island Expedition 1969/72, University of Canterbury, December 1969, C. H. Hay s.n., WELT SP077578!

#### Etymology.

The specific epithet ‘*limenophylax*’ derives from Greek (Ό λιμνοφυλαξ) and, though it strictly means ‘lake watcher’ (C. Blackford pers. comm.), it is used here in the sense of the alternative meaning of “harbour watcher or coast-guard”, which Green (1994; p. 535) states is the more appropriate derivation (see his comments on the etymology of the Norfolk Island endemic orchid *Phreatia limenophylax* (Endl.) Benth.). It is given in allusion to the preferred habitat of *Lepidium limenophylax* on the Snares, i.e. the low turf confined to the exposed ends of rocky headlands jutting out into and overlooking the sea. Historically, it was on these exposed headlands that the World War II coastwatchers were posted to keep watch for signs of enemy shipping (Fleming 1948, McEwen 2005).

#### Description

**Figure 51.**
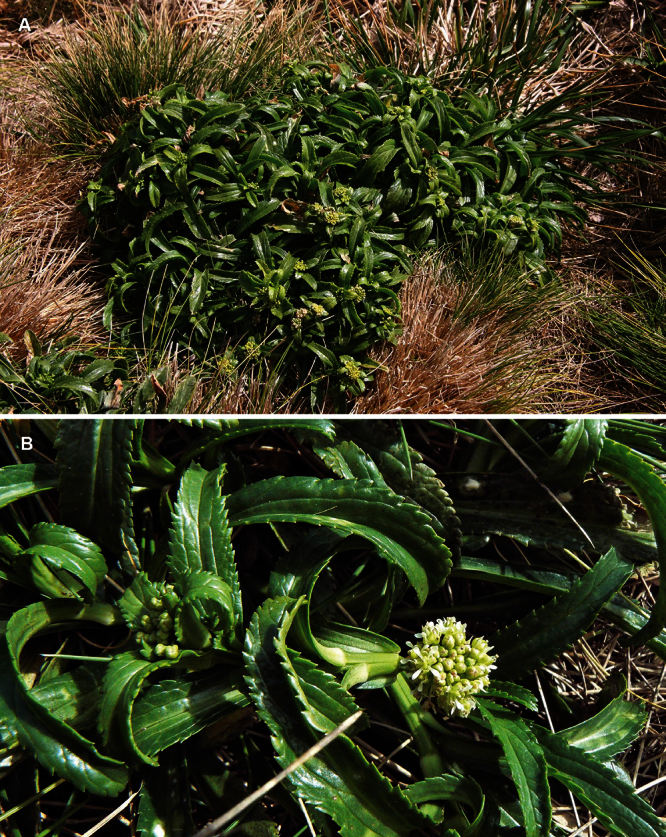
*Lepidium limenophylax*: (**A**) flowering plants on the Snares in *Poa astonii* / *Poa tennantiana* grassland showing decumbent growth-habit (image: R. Ewans); (**B**) close up of young and emergent inflorescence (image: S. Lake).

**Figure 52.**
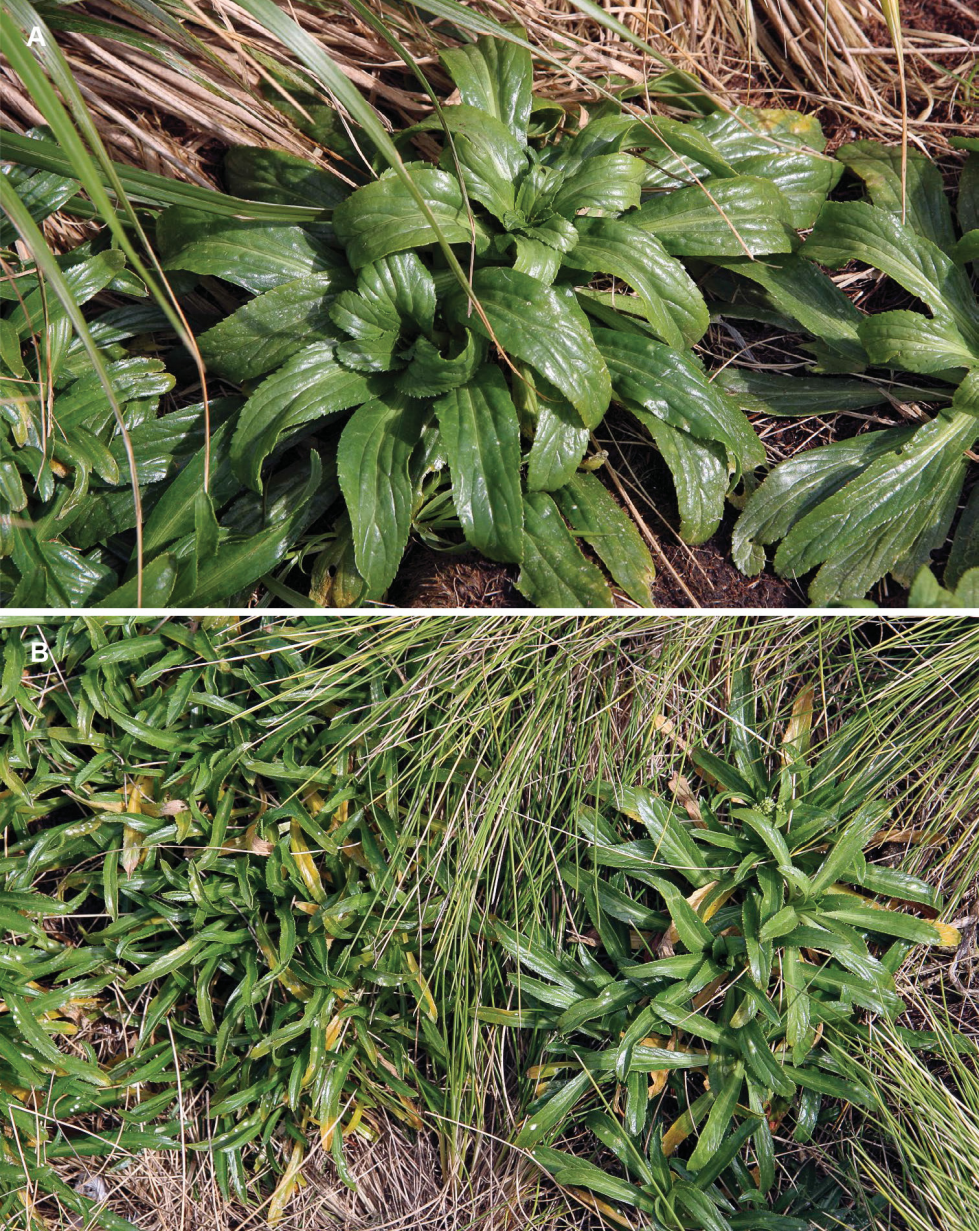
*Lepidium limenophylax*: (**A**) broad-leaved leaf variant (rosette leaves) (image: R. Ewans); (**B**) narrow-leaved variant (image: R. Ewans).

**Figure 53.**
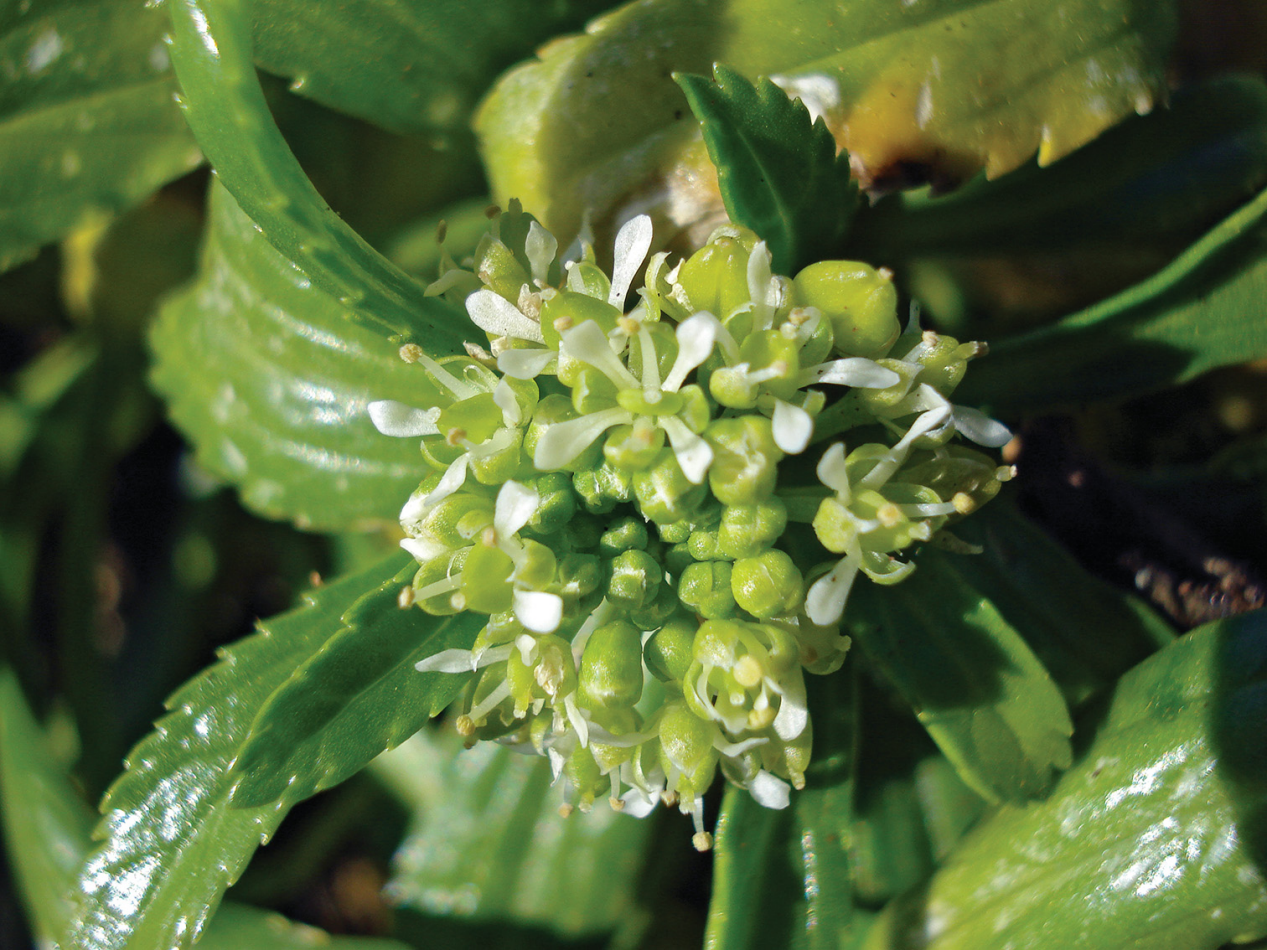
Inflorescence of *Lepidium limenophylax* showing flowers and stamens (image: R. Ewans).

**Figure 54.**
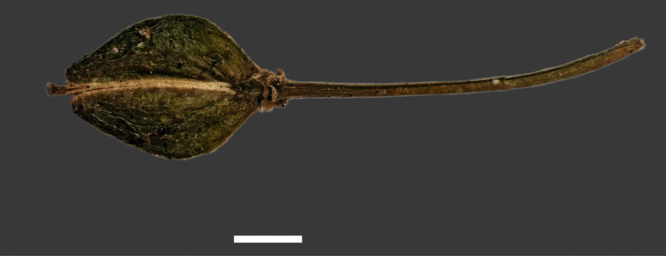
Mature silicle of *Lepidium limenophylax*. WELT SP077578. Scale bar = 1 mm.

**(Figs 51–54).** Tap-rooted, strongly pungent smelling, summer-green, perennial herb, arising from stout rootstock 7.3–10.0 mm diam. Plants dying down to rootstock and previous season’s stem nodes during winter. Stems decumbent, bases often very stout, ± spherical, somewhat woody when mature 10–15 × 10–12 mm, composed of numerous old leaf and stem bases, sometimes producing roots, new season’s grow decumbent, spreading 10–300 × 3–10 mm, glabrous, sometimes with very sparse, appressed, caducous, silky 0.5–1.0 mm long, hairs near stem apices, at fruiting, often devoid of foliage from much of length. Leaves glabrous, firmly fleshy to succulent, usually dark green to green, sometimes yellow-green. Rosette and stem leaves usually withering at fruiting but sometimes with a few long persistent. Petiole distinct, 20–40 × 2–4 mm lamina 50–100 × 5–15 mm, lanceolate, narrowly lanceolate to linear lanceolate; distal ⅓–⅔ finely but sharply serrated or crenate, teeth in 10–20 pairs running to and including subacute, obtuse to rounded apex (teeth not extending beyond leaf outline), base cuneate to narrowly cuneate. Middle stems leaves with petiole indistinct, lamina narrowly linear lanceolate to linear, often recurved to falcate in the distal ⅓ – ½ of leaf length, 50–120 × 3–6 mm; upper ⅔ or occasionally the entire leaf finely but sharply serrated; teeth in 12–30 pairs running to and including the apex, and not extending beyond leaf outline, lamina base tapered, very narrowly cuneate. Upper stem leaves with or without a distinct petiole, petiole if present 40–60 mm, linear to linear-spathulate, occasionally narrowly lanceolate, often recurved or falcate from ½ of leaf length, 30–100 × 2.0–30.0 mm, margins finely but sharply serrated. Racemes 5–15 mm long, terminal and axillary; rachis glabrous; pedicels glabrous, erecto-patent, 2–8 mm long at fruiting. Flowers c. 0.4–1.0 mm diam. Sepals 4, saccate, overlapping at base, green with pale-green to white thickened margin, apex broadly obtuse, shape and size dimorphic; lateral sepals c.0.6–1.0 × 0.6–1.2 mm, broadly ovate to oval, mostly glabrous, sometimes sparsely hairy, hairs 0.2–0.4 mm long, caducous; median sepals 0.9–1.2 × 0.5–1.2 mm, elliptic to obovate, abaxial surface sparsely hairy, hairs 0.2–0.4 mm long, caducous. Petals white to off-white, 1.5–2.0 × 0.3–1 mm, erecto-patent to somewhat spreading, clawed; limb narrowly obovate, apex obtuse, occasionally emarginated. Stamens 2, equal. Nectaries 2, subulate, 0.35 mm long. Silicles cartilaginous when fresh, coriaceous when dry, 2.5–3.5 × 1.5–3.3 mm, elliptic or rhomboid, not winged, apex usually notched (rarely truncate), valves green maturing yellow-green, glabrous; style 0.1–0.3 mm long, exceeding the shallow notch (if notch present); stigma 0.3–0.5 mm diam. Seeds 2, narrowly ovoid, brown, red-brown to orange-brown, not winged, 1.25–1.3 × 0.35–0.60 mm. FL. Nov–Feb. FR. Nov–Feb.

#### Representative Specimens.

**New Zealand (Stewart Island/Rakiura):** Kaimohu Island, 25 February 1965, B. A. Fineran s.n., (CANU 8703). **Snares Islands:** November 1907, B.C. Aston s.n., (AK 4464, WELT SP027621, WELT SP027628); January 1909, B. C. Aston s.n., (WELT SP027623, WELT SP027624, WELT 27625, WELT SP027626); west coast between Signpost Hill and Razor Back, 29 November 1984, G. S. Hardy s.n., (WELT SP078810); on headland just below Signpost Hill, 29 November 1984, G. S. Hardy s.n., (WELT SP078811); December 1947, n. c., R. C. Murphy Expedition, (WELT SP035432); December 1947, F. Newcombe s.n., (WELT SP09710); cliff edge, February 1961, B. Fineran s.n., (CANU 5949); cliff edge, February 1961, B. A. Fineran s.n., (CANU 5950); B. Fineran s.n., Feb 1961, cliff edge, CANU 5994; cliff edge, February 1961, B. A. Fineran s.n., (CANU 5995); cliff edge, February 1961, B. A. Fineran s.n., (CANU 6000A–6000B); Broughton Island, 4. November 1972, D. S Horning s.n., (CANU 18916); July 2002, P. Sagars.n., (AK 283482). **Auckland Islands:** January 1890, T. Kirk s.n., (WELT SP027634).

#### Distribution

**(Fig. 44).** Endemic. New Zealand, South-western Titi (Muttonbird) Islands (Kaimohu Island), The Snares (North East, Broughton islands), and Auckland Islands. *Lepidium limenophylax* is possibly also present on Pohowaitai Island (*B. A. Fineran*, 4 Mar 1965, CANU 8723) another of the south-western Titi (Muttonbird) Islands, although this collection, comprising sterile specimens of a whole young plant and a branch are inadequate to be certain. There are also additional unconfirmed observations of what may be this species from many of the other south-western Titi (Muttonbird) Islands (B.A. Fineran and B.D. Rance pers. comm.).

#### Recognition.

*Lepidium limenophylax* is recognised by the decumbent growth habit (Figs 51, 52), with plants developing a distinct woody network of branches. New season’s growth arises from the nodes left from the previous season’s growth. In this species, the leaves are consistently lanceolate, narrowly lanceolate, linear lanceolate or linear (Figs 50, 51B, 52), though slightly broader in seedlings and basal rosettes. The flowers have two stamens (Fig. 53). It is morphologically most similar to *Lepidium rekohuense*, which also has a decumbent growth habit and flowers with two stamens. *Lepidium limenophylax* has lanceolate, narrowly lanceolate, linear lanceolate or linear leaves, rather than the spathulate to narrowly ovate or elliptic leaves of *Lepidium rekohuense*. Furthermore, the silicles of *Lepidium limenophylax* are elliptic, rhomboidal and unwinged (Fig. 54), rather than orbicular, obovate to ovoid and winged.

#### Ecology.

On the Snares, *Lepidium limenophylax* has been described as an occasional component of cliff top vegetation where it is stated to grow in association with living or dead *Poa astonii* Petrie pedestals (and on occasion in association with *Poa tennantiana* Petrie and *Hebe elliptica* (G.Forst.) Pennell), often near the feeding sites of brown skuas (*Stercorarius lonnbergi* Mathews, 1912) and occasionally around the nests of Buller’s mollymawk (*Thalassarche* *bulleri* (Rothschild, 1893)) or on dry peaty cliff tops (sometimes within the sandy quartz derived from the underlying granite) (Fineran 1969, Hay et al. 2004, Lake and Evans 2011). Further inland, it was also reported by Fineran (1969, p. 3) ‘as uncommon... sometimes present on sites of old abandoned penguin rockeries, where it usually grew with great vigour’. Brian Rance (in litt.), on a visit to the islands in 2000, found that, as with Fineran (1969), *Lepidium limenophylax* was virtually confined to the north-western cliffs. More recently, Lake and Evans (2011) failed to find it at any inland sites. All these authors and individuals noted *Lepidium limenophylax* as uncommon in other habitats, though a few plants have been noted near Boat Harbour growing in association with *Poa astonii*, *Asplenium obtusatum* G.Forst.and *Hebe elliptica* in semi-open situations caused by Hooker’s sea lions (*Phocarctos hookeri* (Gray, 1844)) (Lake and Evans 2011; B. D. Rance pers. comm.). Very little is known about the associations of *Lepidium limenophylax* on Kaimohu Island, though herbarium notes suggest that it grew there in association with what is probably *Lepidium juvencum* (see comments under that species), and B. A. Fineran (pers. comm.) noted that this species was present mostly along ridge crests and above cliff faces, always in association with the feeding sites of skua. Brian Rance (in litt.) has also observed plants apparently matching *Lepidium limenophylax* on several of the Titi (Muttonbird) islands south west of Stewart Island, usually growing along tracks in association with a sparse ground cover of *Apium prostratum* subsp. *prostratum* var. *filiforme* (A.Rich.) Kirk, *Nertera depressa* Banks et Sol. ex Gaertn and *Poa astonii* under semi-open *Olearia angustifolia* Hook.f. / *Olearia lyallii* Hook.f. short forest.

#### Conservation Status.

On the Snares *Lepidium limenophylax* occupies a very narrow habitat range scattered over four key areas and covering an area of 943 m^2^ (Lake and Evans 2011). Lake and Evans (2011) were unable to count or estimate population size stating that ‘it was not possible to estimate the number of plants due to the tendency [of the species] to grow in patches and not [as] isolated plants’. Nevertheless, they reported ‘a good mix of mature and juvenile plants’. Outside these islands, the status of this species on the Auckland and south-western Titi (Muttonbird) Islands is unknown. Previously, *Lepidium limenophylax*, as *Lepidium* aff. *oleraceum* (c) (CANU 5995; Snares), had been listed by de Lange et al. (2009, p. 89) under Appendix 2 Taxonomically Indeterminate Listings, as ‘Acutely Threatened/Nationally Critical’ with the qualifiers ‘IE’ (Island Endemic) and ‘RR’ (Range Restricted) appended. That assessment used data provided by B. D. Rance, on a visit to the Snares Islands in 2000. Lake and Evans (2011) provided a detailed update on the information obtained by Rance during a three week stay on the Snares between September and October 2010. They concluded that the species was in good health, and much more abundant than had been believed previously. While these observations suggest that this is probably a narrow-range species secure within its Snares stronghold, we recommend retention of the current threat listing of de Lange et al. (2009) as a precautionary measure because: 1. Accurate information about the status of this species outside the Snares is absent (i.e. it is not a Snares endemic), 2. The total area of occupancy on the Snares is < 1 ha (criterion A(3) in Townsend et al. (2008)) and 3. There is still no trend data available for the species. However, to reflect the observations of Lake and Evans (2011) and our ongoing uncertainty about population trend and the status of this species outside the Snares we recommend that the addition of the qualifier ‘DP’ (Data Poor) to the current threat listing. It should also be noted that because *Lepidium limenophylax* is known from a number of islands and island groups it does not meet the definition of ‘IE’ in Townsend et al. (2008). *Lepidium limenophylax* also should be qualified ‘CD’ (Conservation Dependent) because the largest known population occurs within a Nature Reserve and World Heritage site (The Snares) where it is vulnerable to the spread of disease, weed and rat incursions, all of which would have a profound impact on its long-term security. Because of these threats, which put at risk not only the *Lepidium* but all other Snares terrestrial biota, access to the islands is strictly controlled by the New Zealand Department of Conservation, which also undertakes regular biosecurity inspections of the island group.

### Lepidium naufragorum

Garn.-Jones et D.A.Norton, New Zealand J. Bot. 33, 43 (1995)

http://species-id.net/wiki/Lepidium_naufragorum

#### Holotype.

**New Zealand:** Open Bay Islands, Taumaka, 15 February 1992, P. J. Garnock-Jones 2121, D. A. Norton & D. R. Given, CHR 470212!

#### Etymology.

The epithet ‘*naufragorum*’, the genitive plural form of ‘*naufragus*’ meaning a ‘castaway or ship wrecked person’, was given by Garnock-Jones and Norton (1995) to commemorate a sealing gang that was set down on the type locality of the species, the Open Bay Islands, in 1810 where they were left for four years until they were rescued.

#### Description

**Figure 55.**
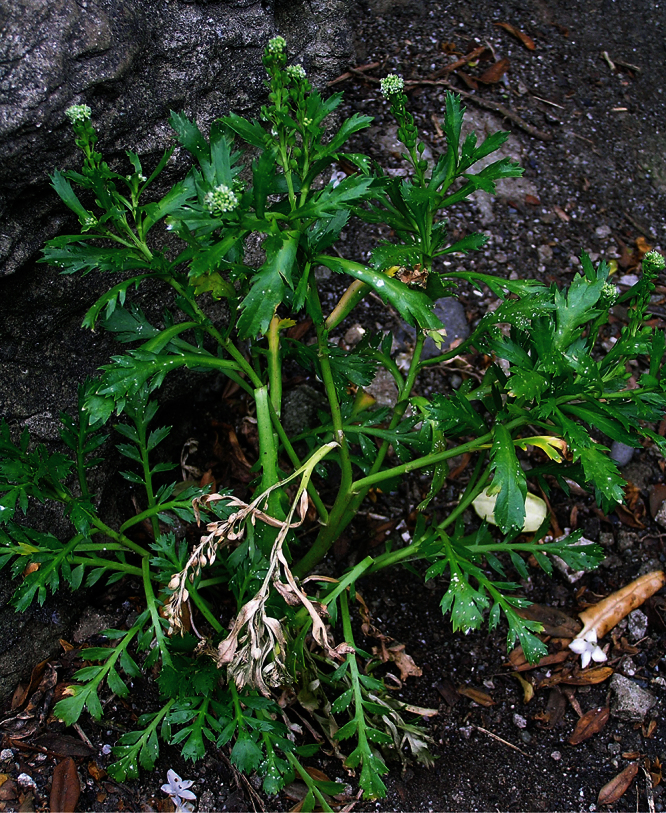
Mature plant of *Lepidium naufragorum* on Taumaka Island (image: P.I. Knightbridge)

**Figure 56.**
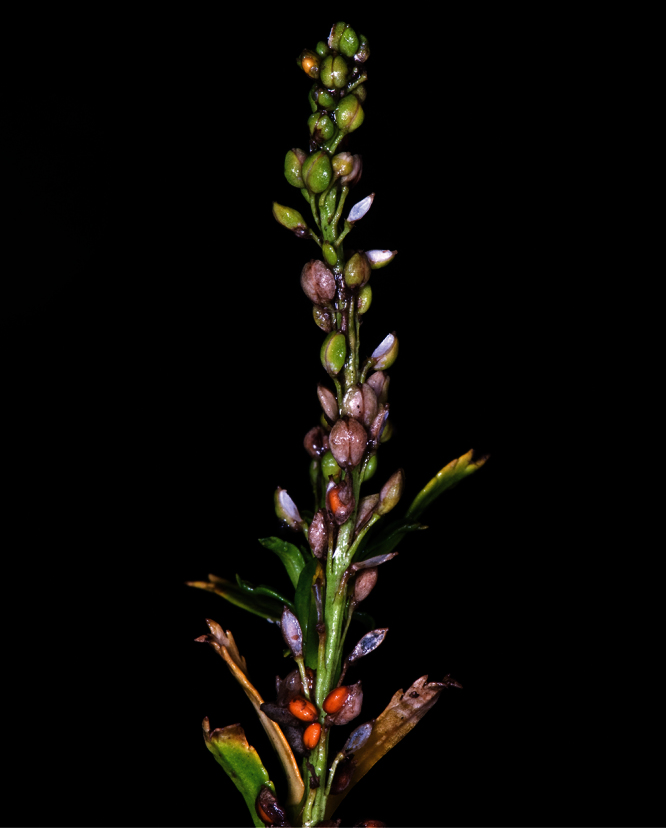
Inflorescence of *Lepidium naufragorum* showing mature and immature silicles.

**(Figs 55, 56).** Tap-rooted, pungent-smelling, summer-green, perennial herb forming a densely leafy, shrub up to 680 mm tall, rootstock stout, 3–10 mm diam. when fresh semi-circular, whitish-grey (when exposed). Tap root fleshy, yellow to yellow-white when fresh, up to 300 mm long, deeply descending. Plants dying down in winter or in times of adversity to rootstock. Stems ascending to erect, 150–680 mm long, glabrous, 2–8 mm diam., woody near base, prominently ridged and/or grooved with age, and usually bearing numerous leaf scars and withered petioles, pale yellow-green to dark green, sometimes tinged maroon; mid to upper portion of stems much branched; branches and branchlets, usually very leafy. Leaves glabrous, fleshy to ± subcoriaceous, glossy dark green to yellow-green, at senescence turning yellow. Rosette and lower stem leaves withering at fruiting; petioles distinct 40–80 × 1–3 mm, slightly concave in cross-section, fleshy, winged; lamina 60–140 × 15–25 mm, pinnatifid (rarely simple), narrow-oblong to narrow-oblanceolate; pinnae when present in 3–8 pairs, sharply toothed toothed at apex and distal margins, lamina of simple leaves, deeply and unevenly serrated, teeth 1.0–2.2(–5.1) mm long, usually not projecting beyond leaf outline. Middle stem leaves similar (rarely simple) or becoming shallowly pinnatifid, sharply serrate. Upper stem leaves 10–50 × 2–10 mm, narrow-obovate to linear oblanceolate, pinnatifid to simple, sharply toothed at apex and at apex of pinnae if present, cuneate at base; petiole minute or absent. Racemes 10–120 mm long, elongating up to 140 mm at fruiting, terminal and axillary, ± leaf-opposed; rachis glabrous or sparsely hairy; pedicels sparsely hairy, erecto-patent, initially1.3–1.6 mm; elongating to 3–5 mm long at fruiting. Flower buds grass green to dark green, apex glabrous or sometimes bearing a conspicuous, caducous, crest of white, eglandular, antrorse hairs up to 0.9 mm long. Flowers sweetly fragrant, 2.8–3.2 mm diam. Sepals, ovate to oval, apex broadly obtuse, green with a white scarious margin, deeply concave, adaxially weakly keeled, abaxial midrib glabrous or invested in conspicuous, caducous, white, eglandular, antrorse, flexuous hairs, hairs sometimes scattered across rest of abaxial surface; adaxial surface glabrous; lateral sepals 0.8–1.2 × 0.8–1.2 mm , median sepals narrower 0.7–1.0 × 0.6–0.9 mm . Petals white, 0.9–1.4 × 0.6–0.9 mm, erecto-patent, clawed; limb obovate, apex emarginate. Stamens 4, equal. Anthers c.0.12 mm long. Pollen bright yellow. Nectaries 4, subulate, 0.25 mm long. Silicles cartilaginous when fresh, subcoriaceous when dry 2.8–4.0 × 2.3– 3.2 mm, broadly elliptic, slightly winged, apex shallowly notched, valves green maturing pale green, glabrous; style 0.1–0.2 mm long, free from the narrow wing, equal to or slightly exceeding the notch; stigma 0.4 mm diam. Seeds 2, 1.6–2.0 × 1.2–1.8 mm, obovoid, orange-brown, not winged, mucilaginous when wet. FL. Oct–Mar. FR. Nov–Apr.

#### Representative Specimens.

**New Zealand (North Island):** Auckland City, Mt Albert, Jesmond Terrace (Naturalised), 9 January 1998, P. J. de Lange 3415, (AK 234450). **New Zealand (South Island):** North Westland, Punakaiki, Seal Island, 29 December 1991, D. A. Norton s.n., (CANU 36438); Punakaiki, Perpendicular Point, 13 February 1992, P. J. Garnock-Jones 2112 & D. A. Norton, (CHR 470203); Motukikie, January 1995, G. Loh s.n., (CANU 37018); Paringa Knight’s Point, 1 January 1990, D. A. Norton s.n. & J. M. Lord, (CANU 35508); Open Bay Island, January 1909, B. C. Aston s.n., (WELT SP030114); Open Bay Islands, Taumaka Island, 15 February 1992, P. J. Garnock-Jones 2117, (AK 229863, CHR 470208); Open Bay Islands, Taumaka Island, 15 February 1992, P. J. Garnock-Jones 2118, D. A. Norton & D. R. Given, (CHR 470209); Open Bay Islands, Taumaka Island, March 2006, P. I. Knightbridge s.n., (AK 317068—pinnate-leaved form); Open Bay Islands, Taumaka Island, 4 January 2006, P. I. Knightbridge s.n., (AK 317070—simple-leaved form).

#### Distribution

**(Fig. 44)**. Endemic. New Zealand, South Island, (Westland) where it is known from seven sites from Cape Foulwind south to the Open Bay Islands (Taumaka and Popotai). With the exception of the Open Bay Islands, *Lepidium naufragorum* is scarce within the mainland part of its range. *Lepidium naufragorum* has also been collected once as a gutter weed in Auckland City, plants having established there from a nearby garden where the species was being cultivated. These naturalised plants persisted for about five years before they died out.

#### Recognition.

*Lepidium naufragorum* is easily distinguished from all other *Lepidium* species in New Zealand by the upright bushy shrub habit (with plants dying down to a central root stock in winter), erect stems, mostly sharply serrated pinnatifid rosette and stem leaves (rarely entire) (Fig. 55), and by the emarginate silicles (Fig. 56). Of the New Zealand species, it is most similar to *Lepidium flexicaule* in which it was included by Garnock-Jones (1988) before field work and further study recognised its distinctiveness. For differences between *Lepidium naufragorum* and *Lepidium flexicaule*, see under *Lepidium flexicaule*.

Although most plants of *Lepidium naufragorum* have pinnatifid foliage, occasional specimens with simple or weakly pinnatifid leaves are also known, and these can at times be locally common (Knightbridge and Newton 2006). It was plants such as these that were the basis for literature records (e.g. Burrows 1972) and collectors such as L. Cockayne and B.C. Aston (WELT!) referring this species to *Lepidium oleraceum* and *Lepidium banksii* (as var. *ovatum*). From *Lepidium oleraceum* these forms can easily be distinguished by the seasonal growth habit (with plants dying back to the rootstock over winter), and by the distinctly emarginate silicles. So far, *Lepidium oleraceum* has not been found within the Westland range of *Lepidium naufragorum*. The confusion with *Lepidium banksii* partly stems from these early collectors’ uncertainty as to what this species is. In any case, non-pinnate leaved forms of *Lepidium naufragorum* are readily distinguished from *Lepidium banksii* by their much smaller (2.8–4.0 × 2.3– 3.2 mm) broadly elliptic and shallowly notched silicles. The silicles of *Lepidium banksii* are much larger (4.5–5.5 × 4.0–5.0 mm), broadly ovate and deeply notched (Fig. 25).

#### Ecology.

The ecology of *Lepidium naufragorum* was described in some detail by Garnock-Jones and Norton (1995) who noted that it preferred sites frequented by seals and nesting sea birds or sea bird roosts. They concluded that the species required these species not only for nutrient enrichment but also because their disturbance kept the habitats of this species open. The authors also noted that *Lepidium naufragorum* was restricted to base-rich substrates.

#### Conservation Status.

*Lepidium naufragorum* has a conservation status of “Threatened / Nationally Vulnerable _CD, RR_” (de Lange et al. 2009). Based on current evidence this ranking is still appropriate.

### Lepidium oblitum sp. nov.

urn:lsid:ipni.org:names:77129259-1

http://species-id.net/wiki/Lepidium_oblitum

*A L. panniformo habitu effuso, foliis saepe nitentibus sparse leviter dentatis ab apicibus surculorum confertis et a serie rDNA ETS differt. A. L. oleraceo habitu effuso aestati-virenti, siliculis valde incisuris et a serie rDNA ETS differt*.

#### Holotype.

**Figure 57.**
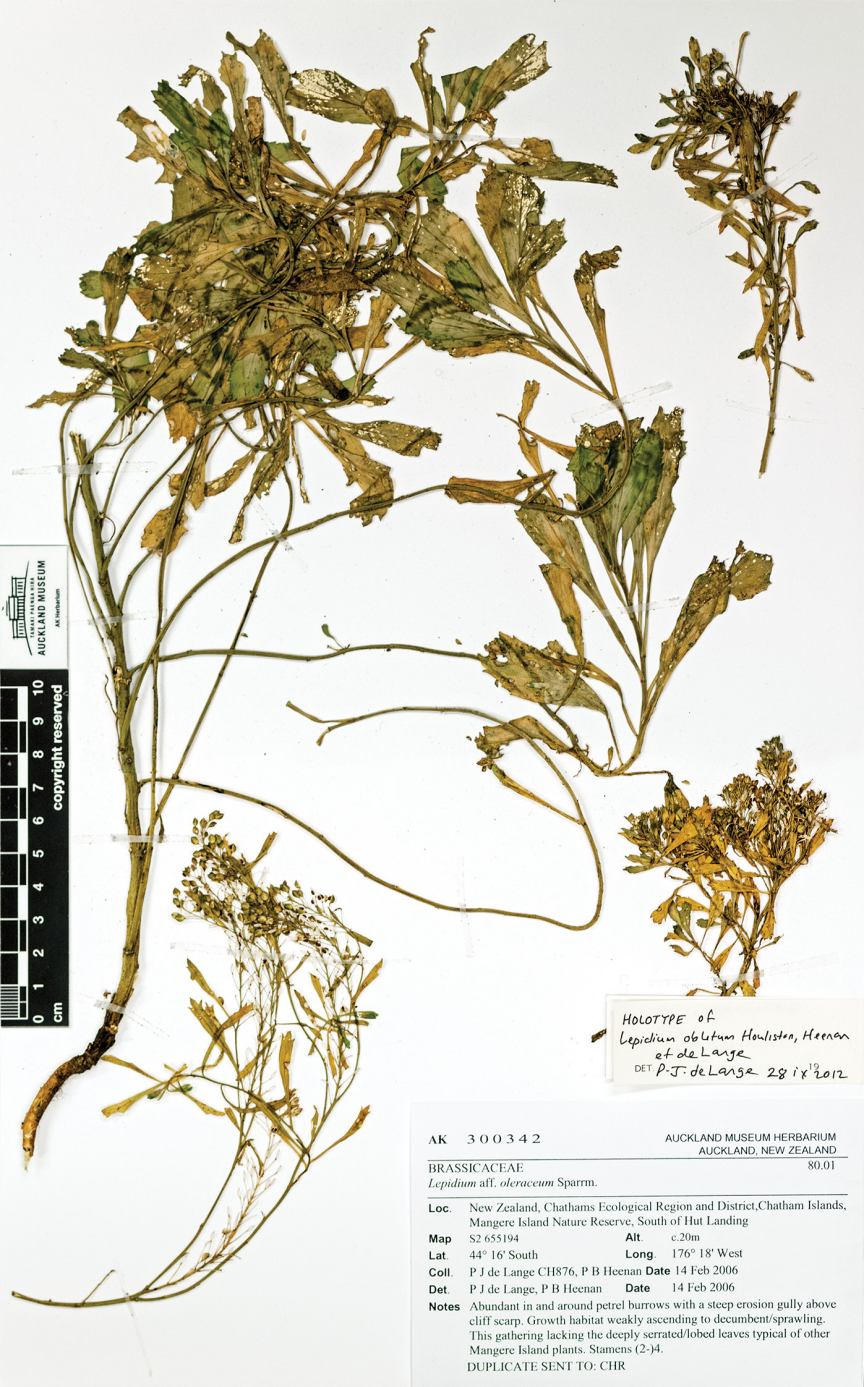
Holotype of *Lepidium oblitum* Houliston, Heenan et de Lange.

**Chatham Islands (Fig. 57):** Chatham Is., Mangere Island, Mangere Island Nature Reserve, South of Hut Landing, 14 February 2006, P.J. de Lange CH876 & P.B. Heenan, AK 300342! Isotypes: CHR 552303A-C!

#### Etymology.

The epithet “*oblitum*” from the Latin meaning “forgotten, disregarded or neglected” refers to the circumstances in which this species was first discovered. At an early stage of this revision *Lepidium oblitum* had been included within the range of *Lepidium panniforme*, with some plants treated as possible hybrids between *Lepidium panniforme* and *Lepidium oleraceum*. It was only following critical analysis of plants grown in New Zealand from samples collected from Mangere Island that the distinctive nature of this species was realized using DNA and morphological data.

#### Description

**Figure 58.**
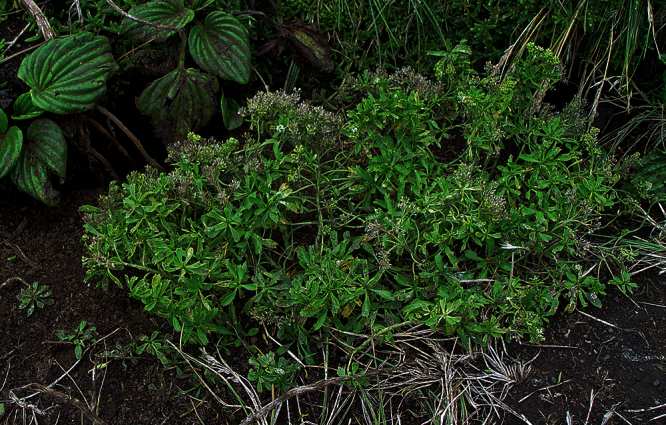
Wild plant of *Lepidium oblitum* showing growth habit of plant associated with *Myosotidium hortensium*, *Disphyma papillatum* and *Festuca coxii* in petrel colony on Mangere Island (image: G.A.S. Taylor).

**Figure 59.**
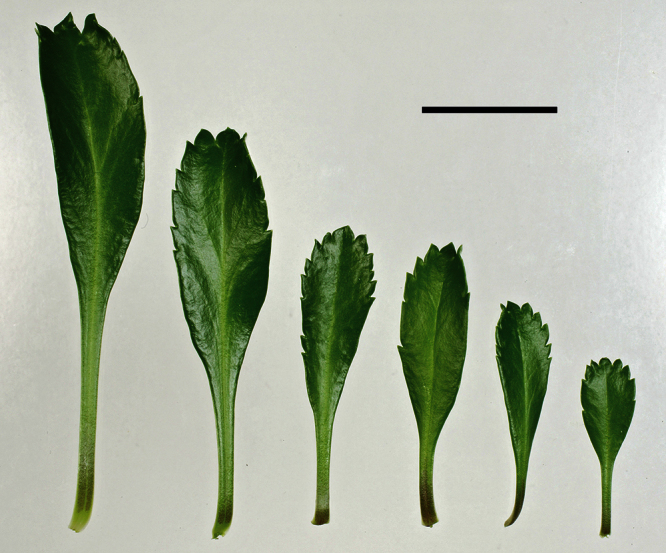
(From left to right) rosette and basal- to mid-stem leaves of *Lepidium oblitum*. (AK 300342). Scale bar = 20 mm.

**Figure 60.**
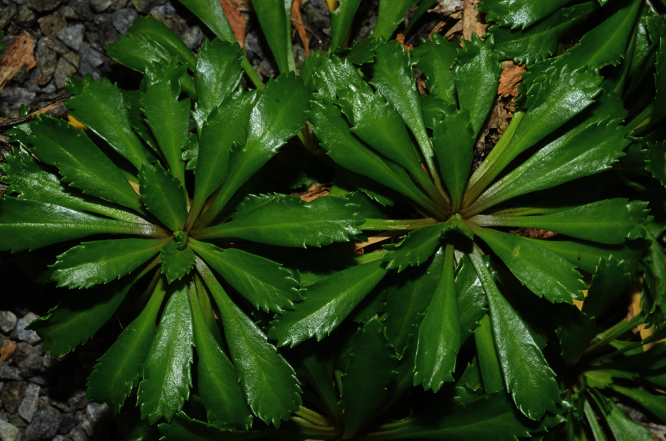
Cultivated plant of *Lepidium oblitum* showing rosette leaves.

**Figure 61.**
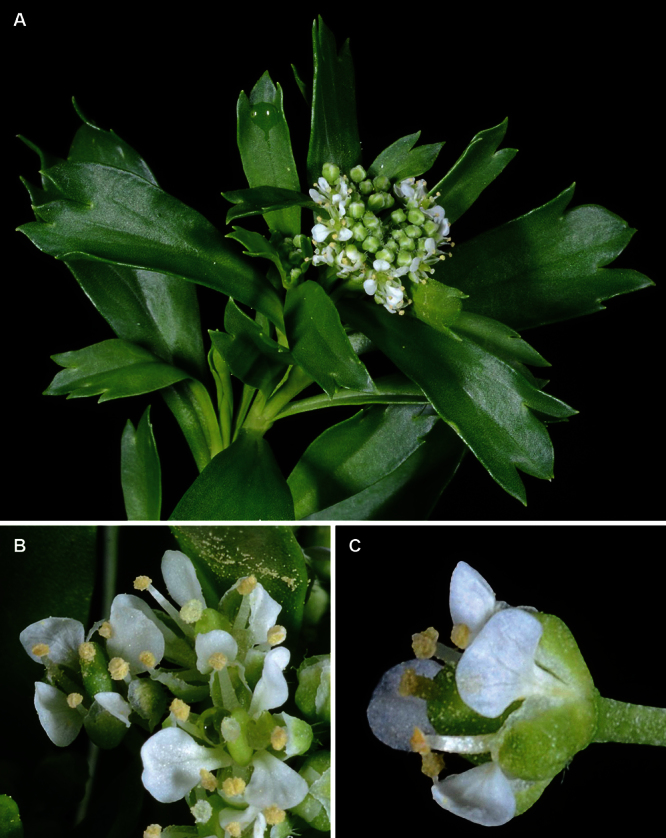
**A** Upper stem leaves and inflorescence of *Lepidium oblitum* **B** flowers showing arrangement of sepals, petals, androecia and gynoecia **C** close-up of flower.

**Figure 62.**
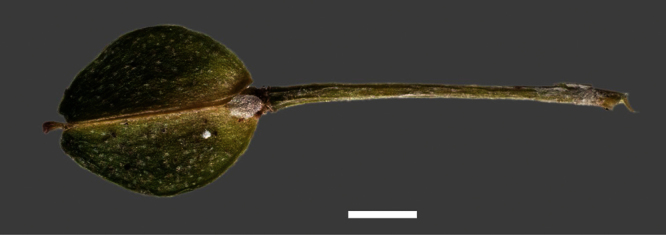
Mature silicle of the holotype of *Lepidium oblitum* (AK 300342). Scale bar = 1 mm.

**(Figs 58–62)**. Tap-rooted, strongly pungent, sprawling, laxly though much-branched, leafy perennial shrub, forming patches up to 1.0 × 0.9 m. Tap-root up to 700 mm long, ± napiform, branched 1–2 or more times. Rootstock 6–14 mm diam., woody, exposed portion ± smooth, often covered in dried leaf remnants and withered branch stubs. Plants usually dying down to rootstock and/or previous seasons stem nodes, towards end of growing season. Stems mostly seasonal, a few ± persistent (i.e. lasting 2 or more years), arising from rootstock base and basal portion of main central stem, widely and unevenly spaced or closely packed, woody, lax and sprawling, sometimes arching and subascendent, weakly angled to ± terete, glabrous; mature stems 2.1–4.6 mm diam., 0.3–0.8 m long; green to yellow-green, brittle, bases bearing numerous leaf abscission scars, otherwise devoid of leaves for at least the first ⅔ with the final ⅓ distinctly leafy and often much branched when vegetative, with the leaves tending to fall at flowering and fruiting; upper stems similar, though distinctly leafy and pliant. Leaves coriaceous, fleshy, green to dark green, often glossy. Rosette leaves 5–14(–20), mostly present in autumn – early spring usually not persisting (very rarely so) at fruiting; petioles distinct, up to 40 × 2 mm, flat or slightly concave in cross-section, succulent; lamina oblanceolate, cuneiform, obovate-oblong to spathulate up to 60 × 22 mm, margins finely to deeply incised in upper ¼–⅓, teeth in 3–6(–8) pairs running to praemorse apex, basal few teeth pair usually asymmetric, base narrowly attenuate. Middle stem leaves mostly persisting at fruiting; petiole up to 32 mm long, mostly flat in cross-section, sometimes slightly concave, usually winged; lamina narrowly oblanceolate, oblanceolate, spathulate, oblong, obdeltoid, 15.0–32.0(–56.2) × 12–16.8(–18.6) mm; margins finely to deeply incised in distal ¼–⅓, teeth in 3–6(–8) pairs running to the usually praemorse apex, basal few teeth pairs usually asymmetric, lamina base narrowly attenuate, extending as a wing (0.8–1.2 mm wide) usually to petiole base. Upper stem leaves petiolate, petiole 12 mm long, flat or slightly concave, usually broadly winged; lamina narrowly oblanceolate, narrowly lanceolate, narrowly spathulate, to linear-cuneiform, 10.2–20.0(–29.0) × 2.4–3.5(–4.8) mm; margins entire or deeply dentate, if dentate then usually asymmetrically tridentate (rarely with 1–4 teeth), lamina base narrowly attenuate, extending as a wing (0.3–1.7 mm wide) almost to petiole base. Racemes (30–)50(–76) mm long, usually congested, elongating up to 100 mm at fruiting, terminal and axillary; rachis and pedicels glabrous (pedicels very rarely bearing a few minute, caducous, glandular hairs near base); pedicels, erecto-patent to patent, 0.4–0.8(–1.0) mm long, elongating to 0.8–1.2(–2.0) mm long at fruiting. Flower buds dark green to green, apex glabrous. Flowers sweetly fragrant, 1.6–1.8(–2.0) mm diam. Sepals 4, saccate, pale to dark green with a broad white, ± undulose margin, pale to dark green with a broad white, ± undulose margin, deeply concave, adaxially weakly keeled or not; lateral sepals 0.9 × 0.6 mm, broadly ovate to oblong, ± overlapping at base, apex rounded to obtuse, adaxial surface glabrous (sometimes diffusely papillate), abaxial surface usually glabrous, sometimes hairy near base, hairs patent, weakly flexuous, 0.1–0.3 mm long, eglandular, shedding at anthesis; median sepals 1.0 × 0.9 mm, broadly ovate to oblong, apex rounded to obtuse, adaxial surface glabrous, abaxial surface glabrous. Petals overtopping sepals, white, 0.9–1.15(–1.3) × 0.5–0.6(–0.8) mm, patent, clawed; limb broadly obovate, apex weakly retuse. Stamens 2–4, equal. Anthers c. 0.10 mm long. Pollen bright yellow. Nectaries 2, subulate, 0.3–0.42 mm long. Silicles cartilaginous when fresh, coriaceous when dry, orbicular, orbicular-ovate to ± rhomboid, (2.8–)3.0(–3.3) × (2.4–)2.6(–3.0), margin winged, notably more so toward apex, apex notched, base obtuse, valves green maturing yellow-green, glabrous, dried surface ± coarsely reticulate; style 0.1–0.2(–0.3) mm long, free from the narrow wing, usually exceeding the notch; stigma 0.18–0.22 mm diam, capitate. Seeds 2, ovoid to ellipsoid, orange-brown to dark red-brown, not winged, 1.8–1.9 × 0.9–0.93 mm. FL Nov–Mar. FR Jan–Jun.

#### Representative Specimens.

**Chatham Islands:** Rabbit Island, 14 February 2006, P.J. de Lange CH878 & P.B. Heenan, (AK 300344). Mangere Island, Mangere Island Nature Reserve, Above Hut, 14 February 2006, P.J. de Lange CH874 & P.B. Heenan, (AK 300340; CHR 552373); Mangere Island, Mangere Island Nature Reserve, ‘Top Plateau’, 22 February 2011, B. Gibb s.n., (AK 331585).

#### Distribution

**Figure 63.**
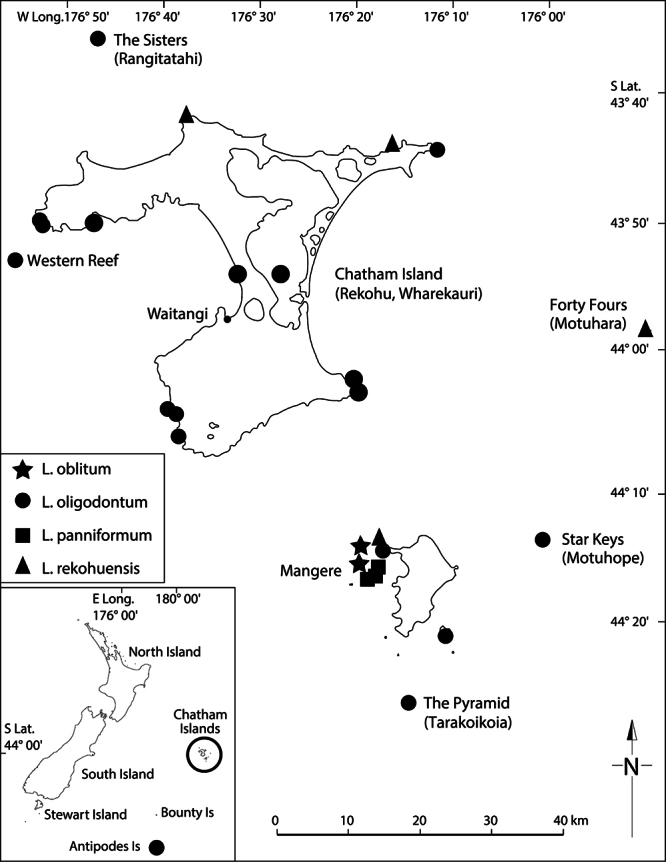
Distribution of *Lepidium oblitum*, *Lepidium oligodontum*, *Lepidium panniforme* and *Lepidium rekohuense*.

**(Fig. 63).** Endemic. New Zealand, Chatham Is., Mangere and Rabbit Islands.

#### Recognition.

*Lepidium oblitum* is recognised by its sprawling growth habit (Fig. 58); by the branches often widely spaced, trailing and mostly devoid of foliage except for the ends which are typically densely terminated by foliage (Fig. 57); and by the prominently notched silicles (Fig. 62). Of those species with which it grows (*Lepidium oligodontum*, *Lepidium oleraceum*, *Lepidium panniforme* and *Lepidium rekohuense*), it can be easily distinguished from *Lepidium oleraceum* by its sprawling growth habit, flowers with 2–4 stamens, and distinctly notched silicles (Figs 58, 61, 62).

From *Lepidium oligodontum*, *Lepidium oblitum* is easily distinguished by it’s more upright, though somewhat, untidy, sprawling growth habit (Fig. 58), and longer lived, persistently leafy branches. The foliage of *Lepidium oblitum* (Figs 59, 60) has leaf margins that are consistently toothed (never sparingly-toothed or entire), while the flowering racemes are distinctly longer ((30–)50(–76) mm cf. (5.0)–9.7(–28.9) mm in *Lepidium oligodontum*)) and on fruiting they may elongate up to 100 m (rather than 60 mm). In *Lepidium oblitum* the flowers have 2–4 rather than 2–4–6 stamens (Fig. 61B, 61C), while the silicles are never fleshy or turgid, rather they are distinctly cartilaginous and prominently (rather than weakly) notched (Fig. 62). *Lepidium oblitum* also has consistently shorter styles ((0.1–0.2(–0.3) mm long) than *Lepidium oligodontum* ((0.2–0.8(1.2) mm long)).

*Lepidium oblitum* and *Lepidium panniforme* grow together on Mangere Island (Fig. 63). As they have similar silicles and flowers with 2–4 stamens but very different foliage, *Lepidium oblitum* was initially thought to be hybrid between *Lepidium panniforme* and *Lepidium oleraceum*. At that time it was not realised how widespread *Lepidium oblitum* is, nor that it comprised stable, true-breeding populations (G. Houliston unpubl. data). It was only following DNA-based investigations undertaken for this paper that the putative hybrid status was rejected and its status as a species recognised. Nevertheless, *Lepidium oblitum* is still closely allied to *Lepidium panniforme*, and both also share a relationship to the extinct *Lepidium obtusatum*, a relationship evident by all three species sharing prominently notched silicles. *Lepidium oblitum* has widely spreading, lax (rather than erect to suberect), sprawling stems and rosette leaves that distinguish it from *Lepidium panniforme* (Figs 82, 83). The leaves of *Lepidium oblitum* are shallowly toothed but they are never as deeply serrated or lacerate as those of *Lepidium panniforme* (Figs 81, 83, 84). DNA data suggests that gene flow between *Lepidium oblitum* and *Lepidium panniforme* has occurred at some sites on Mangere Island (G. Houliston unpubl. data).

*Lepidium oblitum* also grows with *Lepidium rekohuense* on Rabbit Island. From that species it is easily separated by its smaller growth habit; by the glabrous rather than sparsely papillate-hairy upper branch stems, and glabrous rather than finely hairy inflorescences (the pedicels of *Lepidium oblitum* may, very rarely, bear a few caducous glandular hairs). Furthermore, the flowers of *Lepidium oblitum* have 2–4 rather than 2 stamens, while the silicles of *Lepidium rekohuense* are also mostly orbicular (rarely obovate) and slightly larger (up to 4.1 × 4.0 mm cf. 3.3 × 3.0 mm).

#### Ecology.

*Lepidium oblitum* is one of three *Lepidium* species recorded from Mangere and Rabbit islands. On these islands, it is frequently found growing in association with petrel burrows, and on exposed, often wind eroded cliff faces, associated soil blowouts and ephemeral drainages and seepages. On Mangere Island, it is perhaps most common among the boulders and rocks forming the northern summit of the Top Plateau. In these habitats, *Lepidium oblitum* commonly grows with *Asplenium obtusatum*, *Festuca coxii* (Petrie) Hack., *Disphyma papillatum* Chinnock, *Puccinellia chathamica* (Cheeseman) Allan et Jansen and *Senecio radiolatus* F.Muell. subsp. *radiolatus*. On Mangere Island, it is also associated with *Aciphylla dieffenbachii* (F.Muell.) Kirk, *Hebe chathamica* (Buchanan) Cockayne et Allan, *Hebe dieffenbachii* (Benth.) Cockayne et Allan (and hybrids between these two *Hebe*), *Myosotidium hortensium* (Decne.) Baill., and, on occasion, *Lepidium oleraceum* and *Lepidium panniforme*. On Rabbit Island it is associated with *Atriplex australasica* Moq., *Critesion murinum* (L.) Á.Löve subsp. *murinum*, *Malva arborea* L., *Lepidium oligodontum* and *Lepidium rekohuense*.

#### Conservation Status.

*Lepidium oblitum* is so far known only from the Chatham Islands group where it has been collected from Mangere and Rabbit Islands (Fig. 63). On Mangere Island (a Nature Reserve, with strict permit controlled access) it is known from several populations, the largest of which occurs along the northern western cliff faces of ‘Top Plateau’. That population may number in the tens of plants; the only other ones known occur in steep runnels draining the western cliffs south of the main Landing area. When visited by PdL and PBH in 2006, there were fewer than 10 adults in total. Subsequent visits to Mangere Island by Department of Conservation rangers suggest that there has been little change in the population sizes on that island. However, hard data is unavailable and so it would inappropriate to infer from these observations that *Lepidium oblitum* populations on Mangere Island are stable. On nearby privately owned Rabbit Island, two plants of *Lepidium oblitum* were seen in 2006 and the island has not been visited by Department of Conservation staff since.

Therefore, based on available information *Lepidium oblitum* is known from two sites, totalling 4 populations, which collectively are unlikely to exceed 100 mature individuals. Further, the total area of occupancy is considerably less than 1 ha. On the basis of that data, using Townsend et al. (2008), *Lepidium oblitum* qualifies as “Nationally Critical” (using either criterion A(1) or A(3)) because there are fewer than 250 mature individuals known and the total area of occupancy is ≤ 1 ha. We favour criterion A(3) because a precise survey of the numbers of plants of *Lepidium oblitum* has yet to be carried out, and available data (beyond that obtained by PdL and PBH) is unreliable because of understandable past confusion with *Lepidium oleraceum* s.s. and *Lepidium panniforme*.

This conservation assessment should also be qualified ‘CD’ (Conservation Dependent) because Mangere Island is subject to ecological restoration, and ongoing surveillance to ensure it remains predator-free. Should rodents establish on the island, they will affect that island’s *Lepidium* species, both directly through browse and indirectly through predation of the sea birds that maintain this species habitat. Other necessary qualifiers include ‘DP’ (Data Poor) because of the lack of precise information on population size and trend data, ‘IE’ (Island Endemic), and ‘RR’ (Range Restricted) because of its precise habitat requirements and geographically narrow-range.

### Lepidium obtusatum

Kirk, Trans. et Proc. New Zealand Inst. 24, 423, (1892)

http://species-id.net/wiki/Lepidium_obtusatum

#### Holotype.

**Figure 64.**
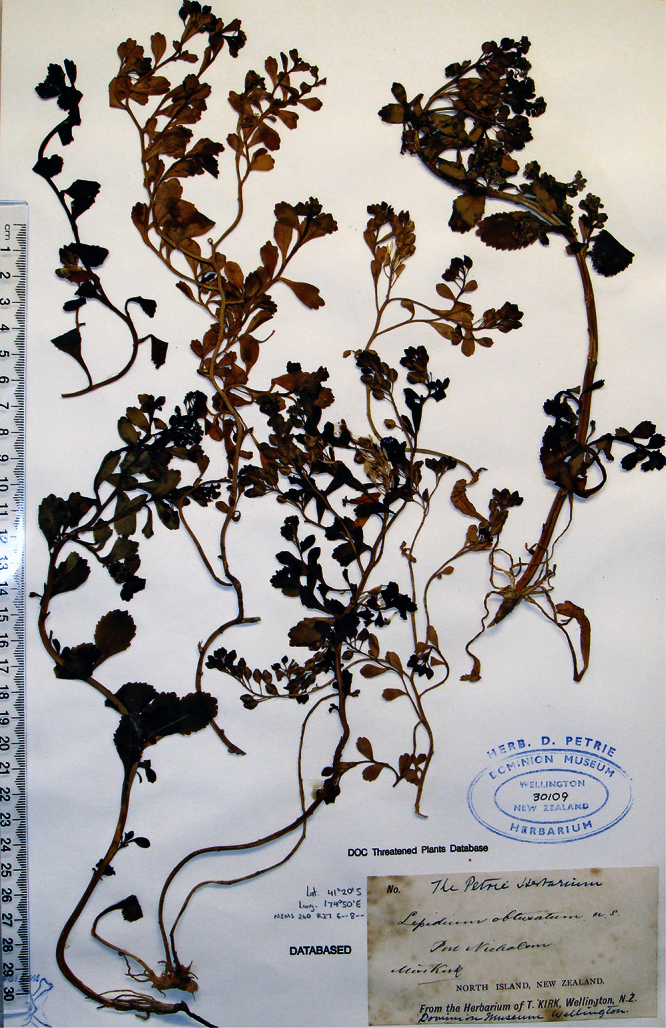
Holotype of *Lepidium obtusatum* Kirk.

**New Zealand (Fig. 64):** Port Nicholson, n.d., Miss Kirk, WELT SP030109!

#### Notes:

Kirk’s protologue for *Lepidium obtusatum* states “Hab. North Island: Maritime rocks at the entrance to Port Nicholson, Miss Kirk” (Kirk 1892). Only one herbarium sheet annotated ‘*Lepidium obtusatum n.s., Port Nicholson, Miss Kirk*’ and so matching the protologue as to name, collector (Miss [Lily] Kirk), type locality, and labelled in the naming authors hand could be located in the WELT (the main herbarium where Kirk material has come to be lodged) so this sheet is regarded here as the holotype.

#### Etymology.

Although the exact meaning of the species “*obtusatum*” was not given by Kirk (1891) it seems that he took the name from the shape of the hypogynous glands (i.e. the nectaries) which he specifically noted (Kirk 1899) are “very short and obtuse”.

#### Description

**Figure 65.**
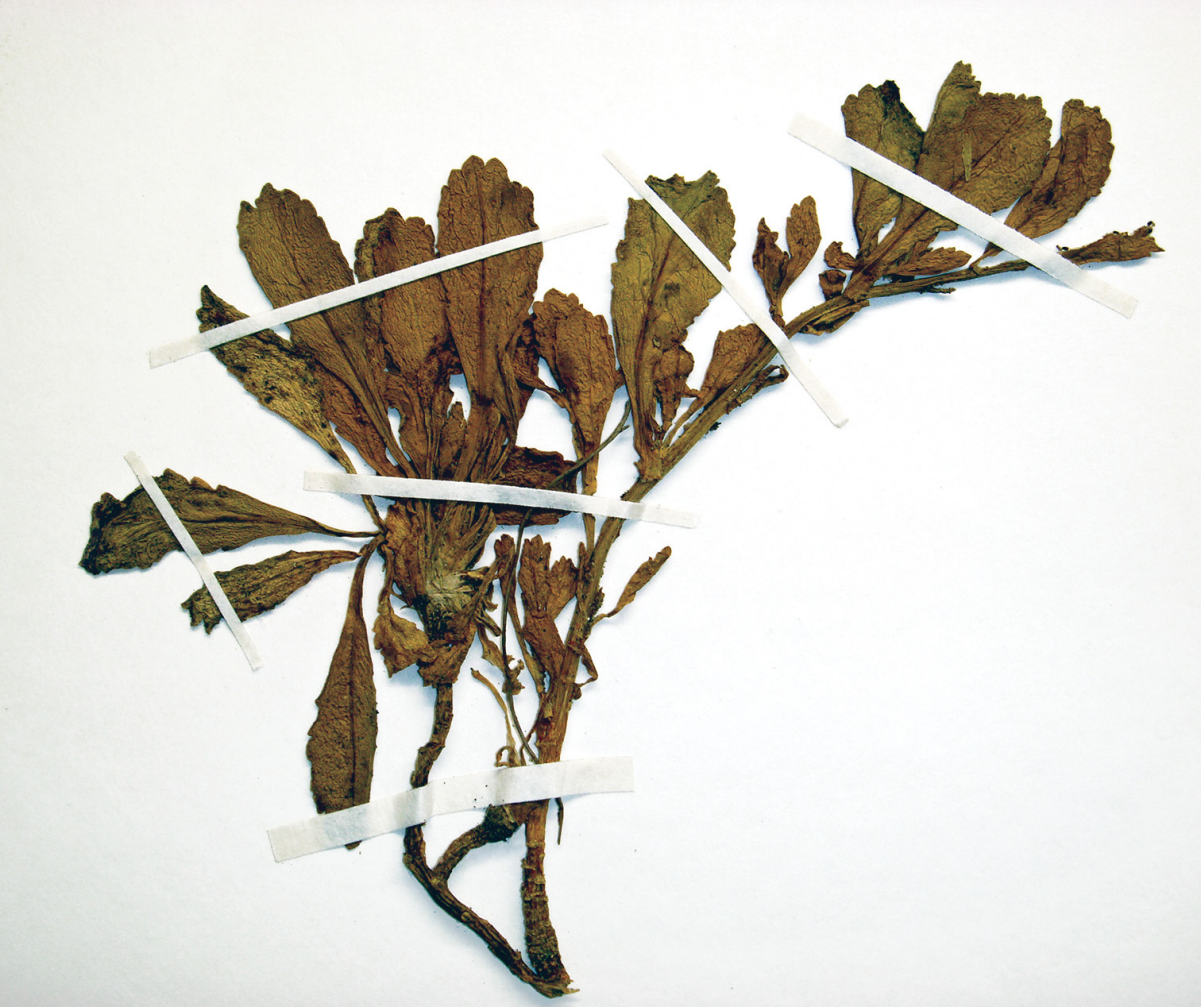
Portion of *Lepidium obtusatum* showing rhizomatous growth and rosette leaves.

**Figure 66.**
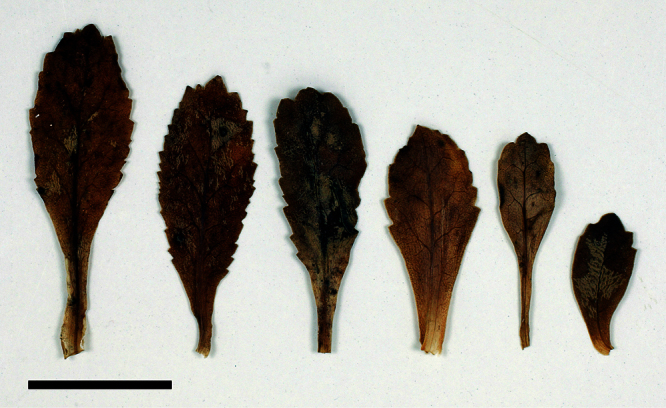
(From left to right) rosette, basal-, mid- and upper-stem leaves of *Lepidium obtusatum*. Scale bar = 20 mm.

**Figure 67.**
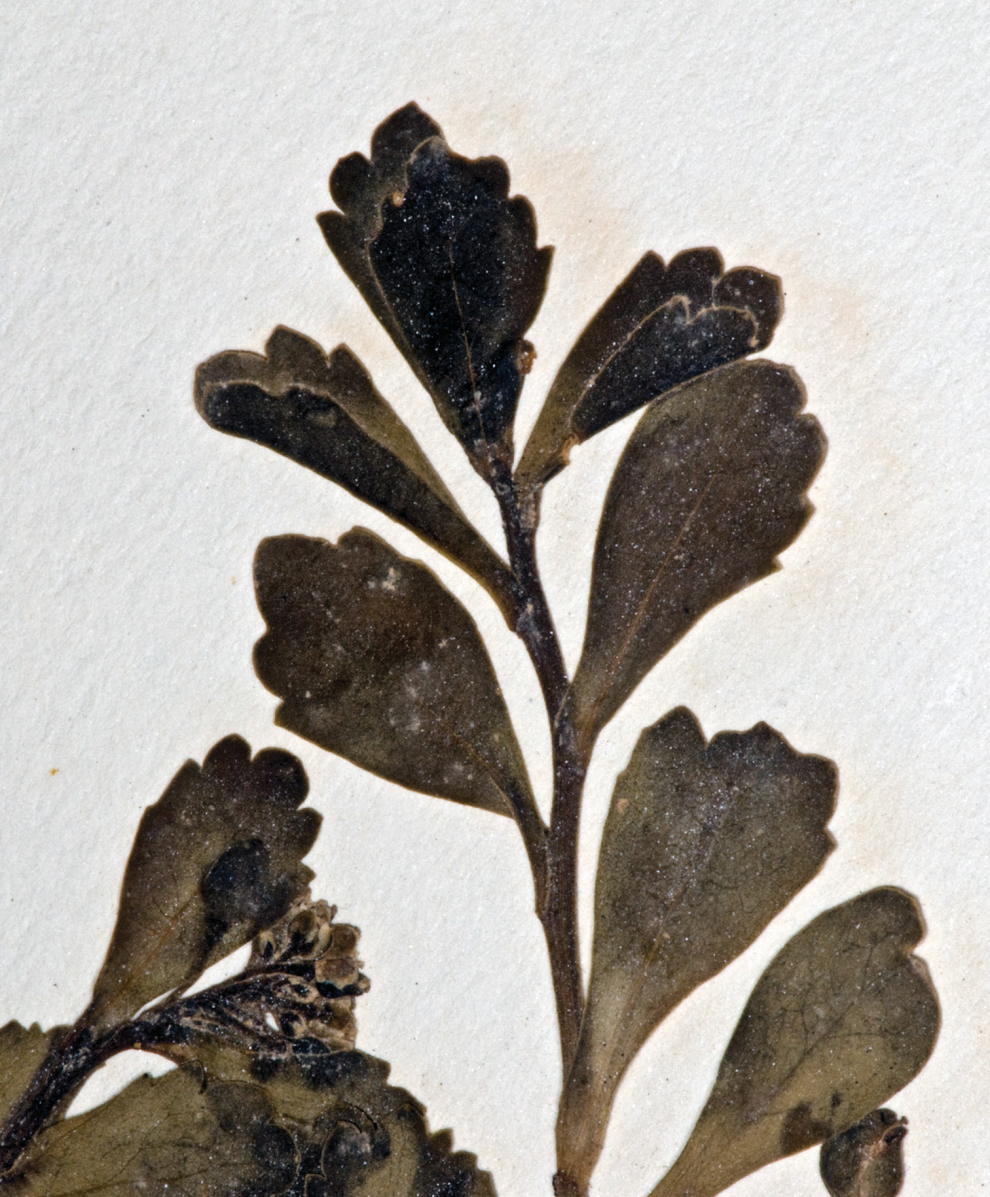
Upper stem leaves of *Lepidium obtusatum*. AK 4476.

**Figure 68.**
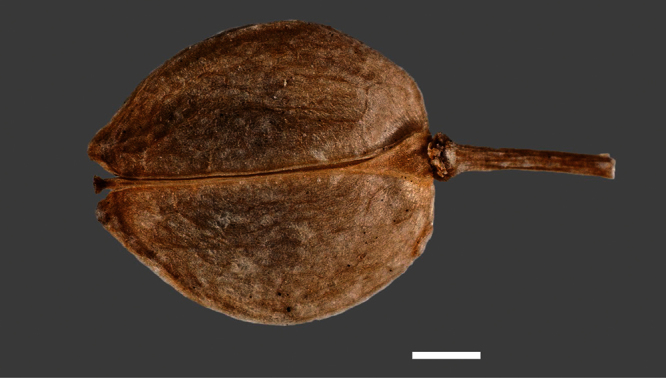
Mature silicle of *Lepidium obtusatum*. CHR 464564. Scale bar = 1 mm.

**(Figs 65–68):** Glabrous, prostrate, much-branched, succulent, rhizomatous, perennial herb, forming patches up to c. 1 m diam. Stems 200–300 × 3–6 mm, prostrate, succulent, flexuous, widely spreading, leafy along stems. Leaves glabrous, thick, succulent and very coriaceous, dark glossy green, variable in size and shape. Rosette leaves 50–80 × 15–20 mm, elliptic to elliptic ovate, oblong, oblong-spathulate; apex, obtuse or rounded, crenate-serrate with 3–4 blunt teeth; margin crenate, crenate-serrate, sometimes weakly bidentate, rarely entire, with 0–24 pairs of teeth; teeth up to 1.8 mm deep, mostly regular size, not protruding beyond leaf outline; base narrowly attenuate to cuneate, ± decurrent, petiole distinct, 32–40 × 1.6–2.9 mm, slightly winged, or not, channelled. Cauline leaves similar to rosette leaves but smaller (up to 42.3 ×13.3 mm), persistent; petioles distinct, and more consistently and conspicuously winged. Upper stem leaves much reduced; lamina 8.8–16.8 × 5.4–8.8 mm, broadly oblanceolate, spathulate, obdeltoid to suborbicular, apex obtuse, usually with 1 prominent and 2 smaller blunt teeth with rounded to obtuse apices (teeth not protruding beyond leaf outline); margin crenate to dentate or entire, if teeth present these in 2–6 pairs, usually blunt ended sometimes subacute, up to 1.2 mm deep, base broadly cuneate to attenuate, petiole distinct, often appearing sessile, usually broadly winged, up to 6 mm long, channelled. Inflorescence terminal and lateral, usually obscured by associated leafy stems, racemose; racemes 9.9–45.6 mm long, rachis 0.6–0.9 mm; pedicels 2.8–3.2 mm long at flowering, erecto-patent, elongating somewhat after anthesis, glabrous. Flowers 4.3–5.0 mm diam. Sepals 4, saccate, overlapping at base, green, apex obtuse, margin white, shape and size dimorphic; lateral sepals 2.2–2.9 × 2.1–3.0 mm, orbicular, mostly glabrous, sometimes sparsely hairy, hairs 0.2–0.4 mm long, caducous; median sepals 1.9–2.9 × 1.5–1.7 mm , elliptic to obovate, abaxial surface glabrescent, sparsely hairy, hairs 0.2–0.4 mm long, caducous. Petals white, 1.3–1.8 × 1.3–1.8 mm, erect, claw minute, 0.2–0.3 mm; limb orbicular, apex obtuse. Stamens 4, ± equal lengths, 1.2–1.8 mm long, base 0.6–1.0 mm wide; anthers 0.6–0.8 mm long, yellow, pollen yellow. Ovary 1.3–1.8 × 1.3–1.7 mm, broadly ovate to suborbicular green, apex distinctly notched; style 0.3–0.5 mm long, cylindrical below, spreading at apex; stigma 0.5–0.6 mm diam. Nectaries 4, green, 0.12–0.14 × c. 0.09 mm, narrow oblong, apex obtuse. Silicles 4.9–6.4 × 4.2–4.9 mm, broadly ovate, oval-rhomboid to obovate, apex prominently notched, valves yellow-green in dried specimens, glabrous, slightly winged; style 0.2–0.5 mm long, not or only slightly exserted. Seeds 2.0–2.7 × 1.8–2.0 mm, obovate, broadly obovate, brown to orange-brown, not winged. FL Jul–Jan. FR Jul–Jan.

#### Representative Specimens.

**New Zealand:** Entrance to Port Nicholson, n.d., T. Kirk s.n., (AK 4476); Entrance to Port Nicholson, between the outer signal station and Worser Bay, 8 March 1892, T. Kirk 363, (WELT SP030102); Port Nicholson, July 1914, B. C. Aston s.n., (AK 4475, WELT SP030108); Seatoun, July 1919, A. Wall s.n., (CHR 329224); Seatoun, 26 November 1920, W. R. B. Oliver s.n., (WELT SP030107); Seatoun, 1931, J. H. MacMahon s.n., (WELT SP081922, WELT SP081925, WELT SP081926); Seatoun, 28 February 1937, W. R. B. Oliver s.n., (WELT SP030105); Seatoun, 3 December 1938, H. H. Allan s.n., (CHR 21596); Wellington Harbour, Point Dorset, 1939, R. K. Ward s.n., (CHR 464546, WAIK 9680); Seatoun, Breaker Bay, near Fort Dorset, 1951, E. Bishop s.n., (AK 250616).

#### Distribution

**(Fig. 44).** Endemic. New Zealand, North Island, Wellington Harbour, Miramar Peninsula, in a small area of coastline between Worser Bay and Breaker Bay.

#### Recognition.

Kirk (1899), Allan (1961), Garnock-Jones (1988) and Garnock-Jones and Norton (1995) considered that *Lepidium obtusatum* was closely related to *Lepidium banksii*. From that species, *Lepidium obtusatum* is distinguished by the prostrate, rhizomatous (Figs 64, 65) growth habit, less deeply serrated (usually crenate to shallowly toothed) rosette and cauline leaves (Figs 64, 66), much smaller broadly oblanceolate, spathulate, obdeltoid to suborbicular, usually crenate margined upper stem leaves (Fig. 67), glabrous pedicles and emarginate shortly-winged silicles (Fig. 68). In this paper we have split west Auckland plants previously treated as *Lepidium obtusatum* into a new species, *Lepidium amissum* (Figs 16–20). From that species, *Lepidium obtusatum* differs by its rhizomatous, prostrate growth habit, flexuose stems (Fig. 64), less deeply toothed (usually crenate-margined) rosette and cauline leaves (Figs 65, 66) which usually wither at flowering and fruiting, broadly oblanceolate, spathulate, obdeltoid to suborbicular, usually crenate margined upper stem leaves (Fig. 67), and inconspicuous inflorescences which are typically obscured by foliage and associated vegetative stems (Fig. 64). However, both species have similar shaped silicles (Figs 20, 68), though those of *Lepidium amissum* are slightly smaller and narrower. *Lepidium obtusatum* and *Lepidium panniforme* have the same ETS sequence but are very different plants: from *Lepidium obtusatum*, *Lepidium panniforme* differs by its non-rhizomatous, upright shrubby growth habit, much larger, deeply lobed to lacerate leaves, and much smaller, unwinged and only slightly notched fruit. Our rDNA ETS data also suggests that *Lepidium oblitum* is related to *Lepidium obtusatum* though, again, they are very different plants (Figs 57–63 (*Lepidium oblitum*) cf. Figs 64–68 (*Lepidium obtusatum*)). *Lepidium oblitum* differs from *Lepidium obtusatum* by its non-rhizomatous growth habit, much larger, prominently toothed (never crenate) leaves and much smaller silicles.

#### Ecology.

Very little is known about the habitat preferences and ecology of this species. It apparently grew on rocky headlands, sea cliffs, coastal rocks and beaches (Kirk 1891, 1899; Cockayne 1921), and some herbarium specimens state that it also grew on sandy and gravel beaches at the high tide mark (e.g., WELT SP030104, SP030107). The species evidently had a most remarkable appearance when fresh, Cockayne (1921) noting that ‘in appearance *Lepidium obtusatum* represents, even when in flower, a huge crusty saxifrage. Its leaves arranged in rosettes are very thick, glossy and rather dark green’. Its thick, fleshy leaves may have the been the basis for a vernacular name ‘sea kale lepidium’ recorded by J. H. MacMahon (see WELT SP081926).

#### Conservation Status.

It is a matter of some irony that when it was described, Kirk (1891) stated, ‘happily for *Lepidium obtusatum*,it grows in a few spots which are inaccessible to sheep, so that it will probably hold its ground for many years’. This prediction did not come to pass; the species was first treated as ‘Presumed Extinct’ by Williams and Given (1981), a status that has been maintained in all subsequent threatened indigenous vascular plant listings to the present (see Cameron et al. 1993; de Lange et al. 2009). The factors leading to the demise of *Lepidium obtusatum* are well documented (Cockayne 1921; Norton et al. 1994; de Lange 2005). The species was a narrow-range endemic whose location on the margin of an actively expanding city meant that it was extremely vulnerable to habitat destruction (see de Lange 2005; de Lange et al. 2010a; de Lange et al. 2010c). Nevertheless it seems that, at least initially, it was very common within the small area it occupied, and Cockayne (1921) mentioned that the quarrying of rock from its cliff habitat, had, temporarily at least, caused an expansion in the population through the spread of ‘underground stems’. Nevertheless, by 1951 it had gone extinct. From available evidence it seems that the biggest factor was not habitat loss but over collection (Norton et al. 1994). Herbarium specimens show that following its formal recognition by Kirk (1891), it was repeatedly gathered by botanists who made copious collections (often gathering whole plants). Most of these gatherings occurred between 1900 and 1939 (and they intensified between 1920 and 1939) ending with the last known collection in 1951 (de Lange 2005). During this period it is also likely that the modification of most of its habitat for housing and defense structures associated with military operations at Fort Dorset would have impacted on the population, as would the spread of weeds, especially those derived from nearby urban gardens. Today, all of the former range of *Lepidium obtusatum* is now choked in a rank growth of weeds, most especially veldt grass (*Ehrharta erecta* Lam.).

#### Hybridism.

A suite of specimens (WELT SP081927A, B, C, *J. H. MacMahon s.n.*, 1931, Seatoun) are tentatively identified here as a putative natural hybrid between *Lepidium obtusatum* and *Lepidium oleraceum*. These specimens, which appear to be pieces split from the same plant seem to have had an erect, shrubby growth habit, with somewhat woody, flexuous branches, while the basal and upper cauline leaves are narrowly elliptic to lanceolate, deeply and sharply incised to weakly pinnatifid. The flowers, though in poor order, have four stamens (pollen stainability 0%). Only a few silicles in the specimens are mature and these measure up to 4.05 × 2.75 mm, are suborbicular to ovate, narrowly winged, with the apex weakly notched. Significantly, they contain no seeds. The putative parentage is based on the knowledge that both *Lepidium obtusatum* (see collections cited above) and *Lepidium oleraceum* were sympatric at Seatoun (WELT SP09712, *W. R. B. Oliver s.n*.) and that no other *Lepidium* species was known to be present in that area at that time. The erect, shrubby growth habit is a feature of *Lepidium oleraceum*, while morphologically the silicles are a close match for *Lepidium obtusatum*. The shape of the cauline leaves fits within the range seen in *Lepidium oleraceum*, though their degree of dentition also approaches that seen in *Lepidium amissum*, *Lepidium banksii* and *Lepidium panniforme*.

### Lepidium oleraceum

G.Forst. ex Sparrm., Nova Acta Soc. Sc. Upsal. iii, 193 (1780)

http://species-id.net/wiki/Lepidium_oleraceum

#### Type Collection:

From cultivated plants, originally collected from New Zealand (see below).

#### Neotype

**Figure 69.**
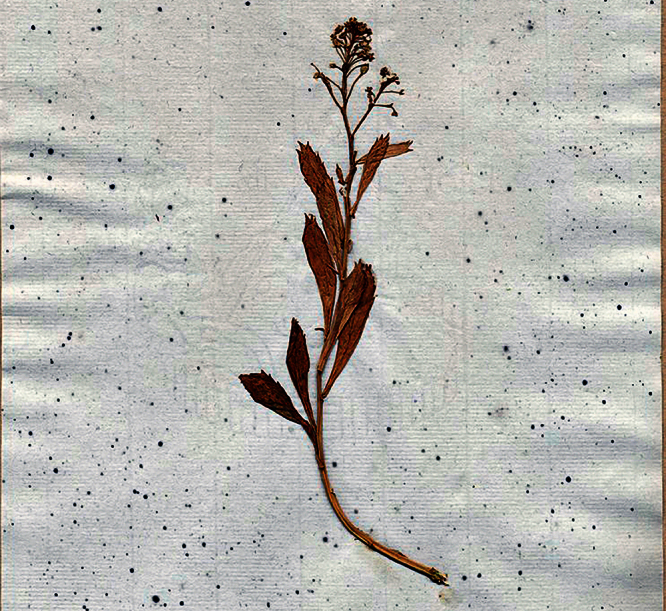
Neotype of *Lepidium oleraceum* G.Forst. ex Sparrm.

**(Fig. 69, designated here):** S, Sparrman, Nova Zelandia.

#### Notes:

Nicolson and Fosberg (2004) were unsure about typification of *Lepidium oleraceum* G.Forst. ex Sparrm., and the name has not been typified. They listed two Sparrman specimens at S, but did not select a type specimen since neither of these appeared to have been gathered from cultivated plants. Given the revised taxonomic treatment presented here for the *Lepidium oleraceum* complex, it is necessary to be certain of the application of *Lepidium oleraceum* G.Forst. ex Sparrm. Therefore, we select one of the Sparrman sheets as neotype.

When describing *Lepidium oleraceum*, Sparrman (1780) indicated that the description was based on cultivated plants: ‘*Prima vero, quam heic ex Forsterianis offerre licet, in praedi patrimoniali Sponga, seminibus ex Nova Zelandia allatis, culta per maximam partem aestatis floruit, Forsteroque Lepidium Oleraceum dicta, quippe qua, cum oleo & aceto, oleraceorum loco, ut & in jusculo cocta, deficientibus alius generis vegetabilibus, in itinere utebamur*’. Indeed, *Lepidium oleraceum* was cultivated in Upsala (Sweden), where Sparrman resided, as it is one of 11 species of *Lepidium* listed by Thunberg (1803) as having been grown there between 1780 and 1800.

It is unusual that this description was based on cultivated plants since it was the practice of A. Sparrman and G. Forster to prepare descriptions from freshly collected material during Captain Cook’s second voyage (Forster and Forster 1776 (see Edgar (1969) for translation). Plant material of *Lepidium oleraceum*, along with other edible, herbaceous coastal plants was frequently collected during the New Zealand part of the voyage to be utilised for their antiscorbutic properties (de Lange and Norton 1996). Therefore, there would have been ample opportunity to prepare the description from freshly collected material. Indeed, as noted by Nicolson and Fosberg (2004), the published description of the later homonym *Lepidium oleraceum* G.Forst. differs from that of *Lepidium oleraceum* G. Forst. ex Sparrm., and it is most likely that the description of the homonym was prepared on the voyage from fresh material.

The wild locality of the neotype of *Lepidium oleraceum* is not known, except that it is from New Zealand. While anchored in Queen Charlotte Sound during May 1773, G. Forster noted ‘the antiscorbutic plants grew on every beach’ and ‘we immediately gathered vast quantities... of a well-tasted scurvy-grass (*Lepidium*)’ (Forster 1777, p. 126). They also collected it from Long Island (Hoare 1982, vol. II, p. 287), and saw it in the vicinity of Maori settlements: ‘near all the places where the Indians have their huts, there grows a kind of Scurvy-Grass or *Lepidium*’ (Hoare 1982, II, p. 297). Therefore, since J. R. Forster, G. Forster, and A. Sparrman would have had ample opportunity to collect *Lepidium oleraceum* in Queen Charlotte Sound it is very likely that the neotype was collected from there. Dusky Sound, Fiordland, is the only other location where landfall was made in New Zealand during Cooks 1773 voyage, but the species is not known from the Fiordland coastline so is unlikely to have been collected from there.

#### Etymology.

The exact meaning of the species ‘*oleraceum*’ was not given by Sparrman (1780). However the epithet derives from the Latin ‘*oleraceus*’meaning ‘used for herbs or vegetables’ (Taylor 2002), a point that Sparrman had alluded to in a brief note he wrote in Latin on the backside of the neotype sheet.

= *Lepidium oleraceum* var. *acutidentatum* Kirk, *Stud. Fl. N.Z*., 35 (1899)

#### Type Collection:

‘NORTH and SOUTH Islands; STEWART Island; the SNARES; AUCKLAND Islands; CHATHAM Islands. In places near the sea.’

#### Neotype

**Figure 70.**
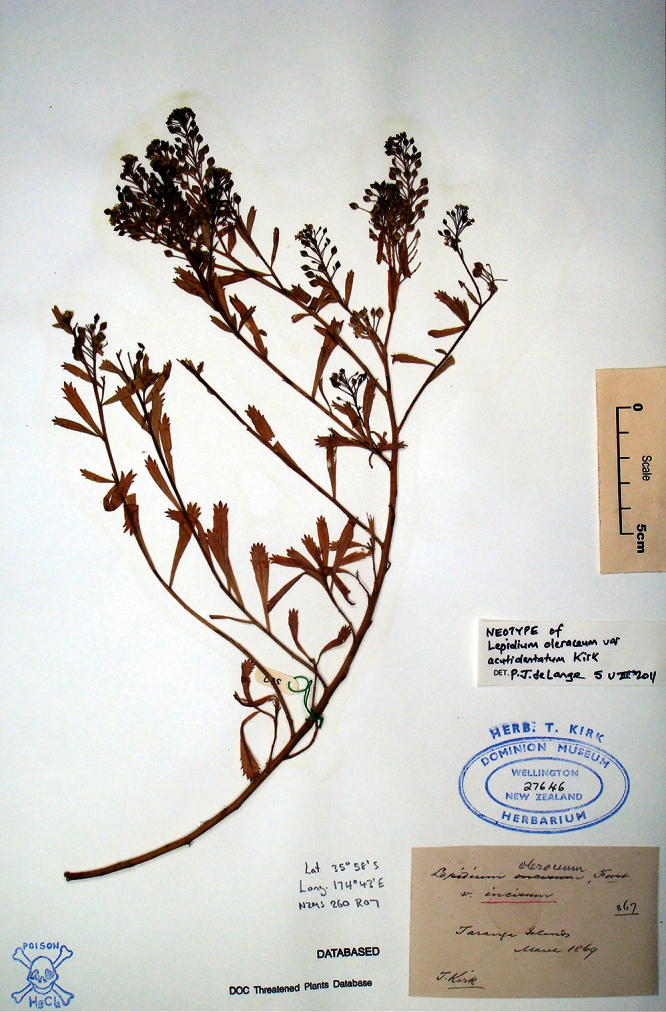
Neotype of *Lepidium oleraceum* var. *acutidentatum* Kirk.

**(Fig. 70, designated here):** T. Kirk 367, March 1869, Taranga Islands – labelled as ‘Lepidium oleraceum var. incisum’ in Kirk’s hand. WELT SP027646!

#### Notes:

Kirk (1899) described var. *acutidentatum* thus ‘stems with slender leafy branches. Leaves 1in.–1½in. long, narrow, cuneate or oblong-spathulate, the upper portion acutely serrate or almost dentate’. Plants matching this vague description span large parts of the range of *Lepidium oleraceum* s.l. Our searches of the herbaria where Kirk traditionally lodged specimens (see comments by de Lange 2007) have failed to find any specimens labelled by Kirk as var. *acutidentatum*. However, there are a range of specimens in WELT (WELT SP027646!, SP027647!, SP027648!, SP030087!) bearing in Kirk’s hand the manuscript names ‘var. erectum’ and ‘var. incisum’, which, being unpublished, have no nomenclatural status whatsoever. These specimens, along with the generally confusing notes provided by Kirk (1899, p. 34–35) in his entry for *Lepidium oleraceum*, suggest that *Lepidium* was a genus he was still working on close to his death on 8 March 1898 (Moore 1973, but see also comments by de Lange 2007 and de Lange and Gardner 2002). At the time of his death, Kirk was using these manuscript names on his herbarium specimens, but he was either undecided on his final choice of epithet or had yet to relabel his specimens as var. *acutidentatum*, a name which was then later published posthumously when his unfinished Flora manuscript was uplifted and published (Kirk 1899). Despite this confusion, Kirk’s description of var. *acutidentatum* matches most closely those WELT specimens (*T. Kirk 367*, WELT SP027646, *T. Kirk 368*, WELT SP027647) that he had collected from Taranga Island, which is the largest of the Hen and Chicken Islands group and labelled ‘var. incisum’. Notably, both specimens match his protologue as to the description of var. *acutidentatum*, e.g., ‘slender leafy branches... Leaves 1in.–1½in. long, narrow, cuneate…upper portion acutely serrate’. Therefore, in the absence of any suitable material for a lectotype we designate *T. Kirk 367* (WELT SP027646) as the neotype of *Lepidium oleraceum* var. *acutidentatum* Kirk.

#### Etymology.

The meaning of the epithet ‘*acutidentatum*’ was not given by Kirk (1899). However, the epithet as indicated by the protologue probably derives from the sharply toothed leaves.

= *Lepidium oleraceum* var. *frondosum* Kirk, *Stud. Fl. N.Z*., 34 (1899)

#### Type Collection:

‘Banks and Sol., MSS and Ic.’

#### Lectotype

**Figure 71.**
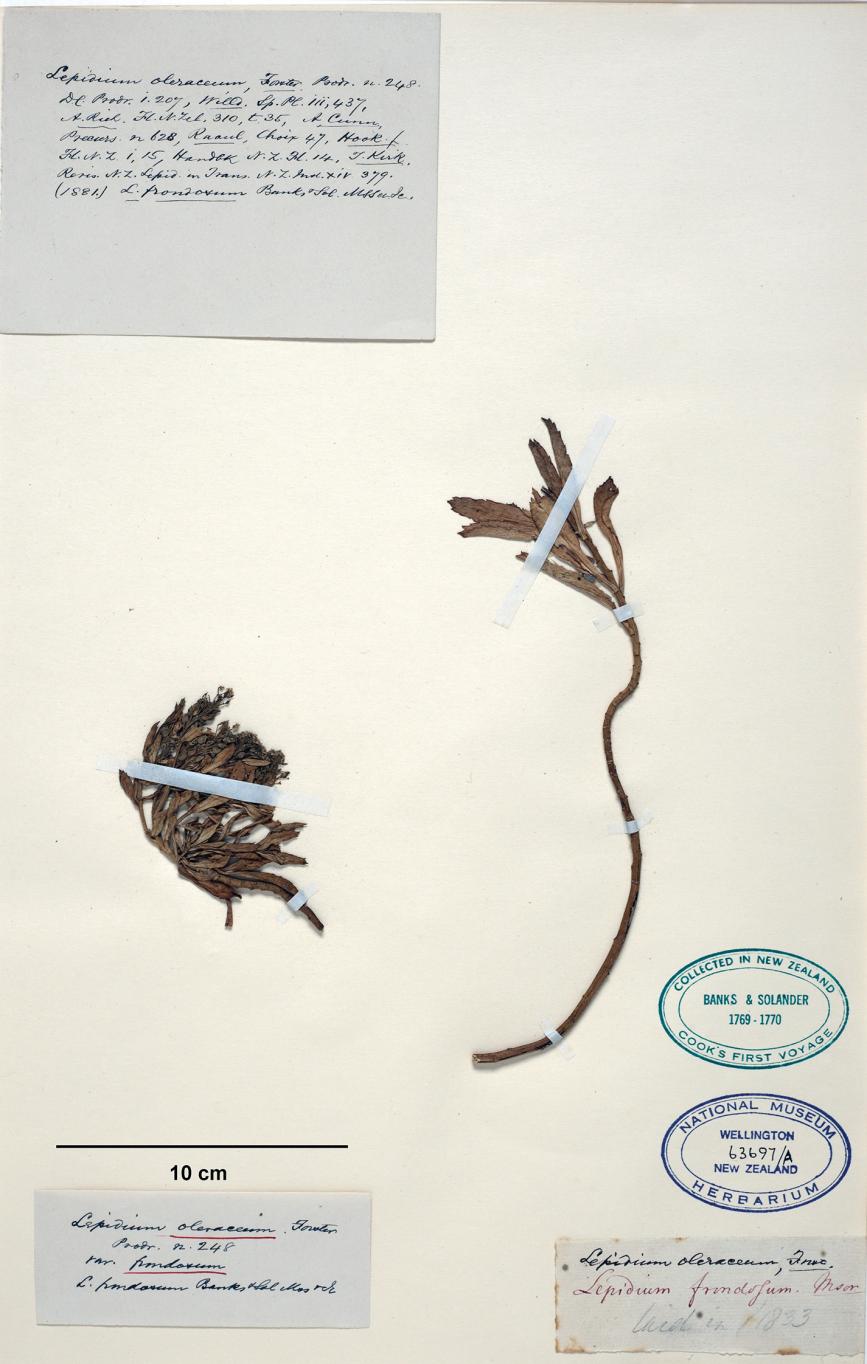
Lectotype of *Lepidium oleraceum* var. *frondosum* Kirk. © Museum of New Zealand Te Papa Tongarewa.

**(Fig. 71, designated here):** WELT SP063976/A! IsolectotypeWELT SP063976/B!

#### Notes:

Kirk (1899, p. 34) despite his rather confused and evidently incomplete treatment of *Lepidium oleraceum* nevertheless clearly described *Lepidium oleraceum* var. *frondosum* in the following manner: ‘var. **frondosum**, (*sp*.), Banks and Sol. MSS. and Ic. Robust, leaves large, fleshy, broadly cuneate-oblong or oblong, sometimes 3in-5in. long and 1in. wide, sessile or narrowed into a broad petiole, serrate’. Although no specimens or locations were cited by Kirk in his protologue, his wording makes it clear that any specimens collected by Banks and Solander and labelled by them ‘Lepidium frondosum’ and any accompanying illustrations made from these specimens constituted the type collections from which he formally recognised his *Lepidium oleraceum* var. *frondosum*. Therefore, we have selected WELT SP063976/A as lectotype because this sheet, comprising two flowering and fruiting, leafy specimens, is labelled both by Kirk ‘*Lepidium oleraceum* Forster Prodr. N. 248 var. *frondosum*’ and also in Solander’s hand as ‘Lepidium frondosum Mscr’.

#### Etymology.

The meaning of the epithet ‘*frondosum* was not given by Kirk (1899) who took the name from the unpublished Banks and Solander manuscript. Nevertheless it seems reasonable to assume that the epithet refers to the large, much tooth, arched leaves typical of the robust forms of *Lepidium oleraceum* that Kirk referred to this variety.

= *Lepidium oleraceum* var. *serrulatum* Thell., *Die Gatt. Lepidium*, 293, (1906)

#### Holotype

**(Fig. 1):** P! ‘*Lepidium oleraceum* Forst. var. *serrulatum* Thell. n. var. 1905 3 VIII, New River, Godey’ (label in A. Thellung’s hand).

#### Notes:

Thellung (1906, p. 293) described *Lepidium oleraceum* var. *serrulatum* from a single undated gathering which, according to the specimen details, came from ‘New River’ where it was collected by Godey. Allan (1961, p. 177) equated this location thus: ‘S[outh Island]. New River estuary, Riverton, Southland’. The holotype bears two labels, one written in the hand of the naming author, Thellung, and the other in an unknown hand, presumably Godey, which reads ‘*Lepidium oleraceum* Forst var. New River, Nouv. Zélande, Godey’. We have been unable to find out who the collector Godey was.

Thellung distinguished his new variety from *Lepidium oleraceum* s.s. thus: ‘*folia obovata, a medio ad apicem regulariter subtiliter et acute serrator*’ meaning ‘foliage obovate, regularly and acutely, finely serrated from middle of the leaf to the apex’.

Significantly the silicles of the holotype are acute rather than notched, a condition seen only in *Lepidium castellanum* and *Lepidium oleraceum*, neither of which are known from the southern South Island. For this reason, we suspect that the gathering was collected from the northern part of New Zealand, rather than the southern South Island, and that Allan’s interpretation of location of ‘New River’ is incorrect. We also suspect that the exact location of the ‘New River’ will now never be known. In all probability this name may have simply been one used locally for some other part of New Zealand.

The obovate leaves of *Lepidium oleraceum* var. *serrulatum* place this variety within *Lepidium oleraceum* (as treated here) rather than *Lepidium castellanum*. Within *Lepidium oleraceum* s.s., there are a few vegetatively similar, obovate-leaved plants with finely serrated leaf margins that approach the extreme condition seen in var. *serrulatum*. These gatherings all come from offshore islands in the northern part of the North Island (e.g., Three Kings Group, *A. E. Wright 6072*,AK 173005; Rock Stack north of Motuopao Island, *R. Parrish s.n*., AK 196229; Motutakapu Island, *R. Parrish s.n*., AK 209112). However none of those obovate-leaved plants have such prominently serrated leaf margins as the holotype of var. *serrulatum*. Based on these observations, we think that var. *serrulatum* was collected from somewhere in the northern part of the range of *Lepidium oleraceum*, and that it represents nothing more than an extreme form of that species, which even following our treatment here remains a highly variable species.

#### Etymology.

Thellung (1906) chose the epithet ‘*serrulatum*’ to reflect the finely serrated / serrulate leaves of the holotype.

#### Description

**Figure 72.**
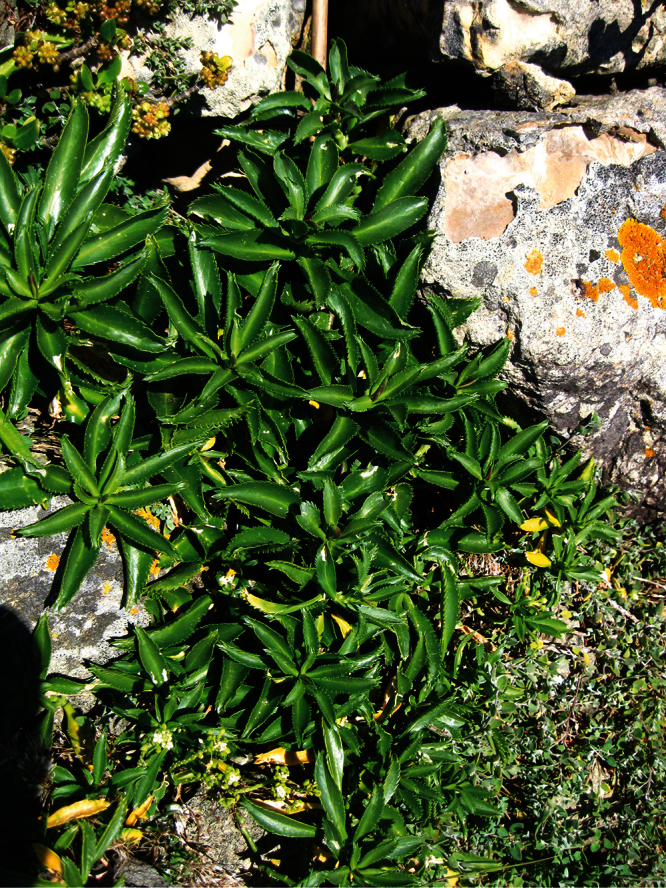
Growth habit of *Lepidium oleraceum* on North Brother Island (image E. Dale).

**Figure 73.**
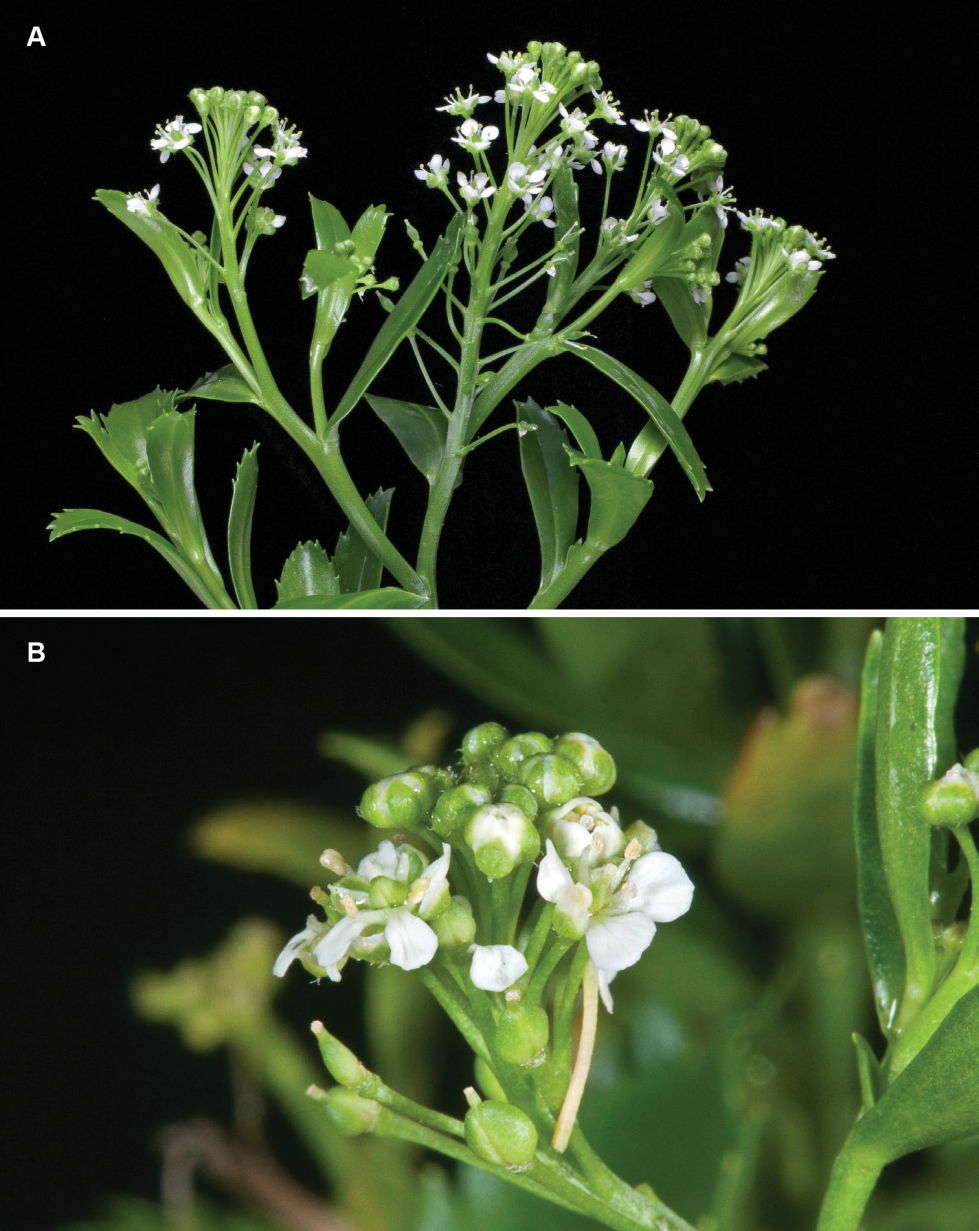
(**A**) Inflorescences of *Lepidium oleraceum*; (**B**) close up of flowers.

**Figure 74.**
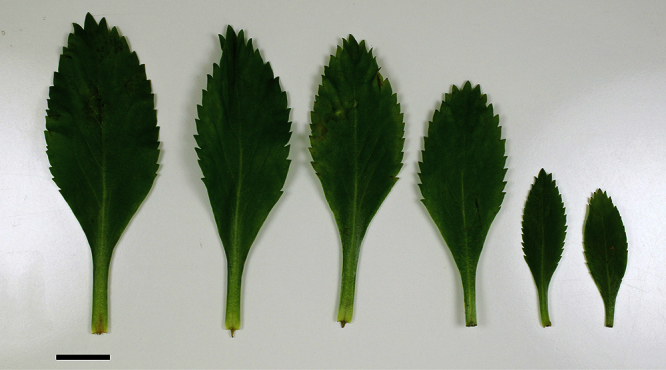
(From left to right) basal-, mid- to upper-stem leaves of *Lepidium oleraceum*. Scale bar = 20 mm.

**Figure 75.**
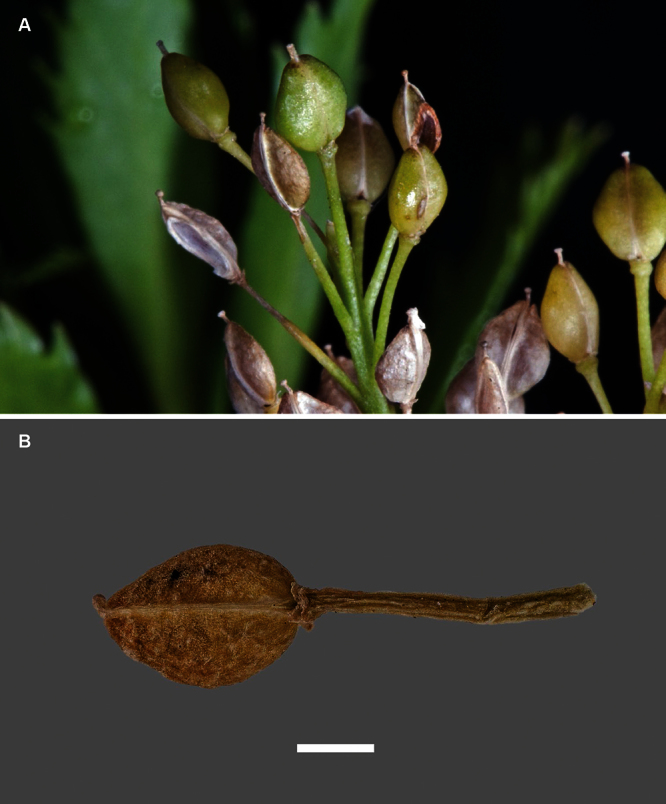
(**A**) Mature and dehisced silicles of *Lepidium oleraceum*. (**B**) Mature silicle beginning to disarticulate at the base and at the apex (causing it to appear to have a notch). Scale bar = 1 mm.

**(Figs 72–75).** Tap-rooted, glabrous, strongly pungent smelling, much-branched, erect, perennial, herb up to 1.2 × 1.3 m, usually less. Rootstock woody, exposed portion smooth, in old specimens usually retaining dead stem remnants admixed with actively growing stems. Stems sparse to closely packed depending on local growing conditions, persistent (only partially dying back in winter), erect to spreading; mature stems 0.2–1.5 m long, 3–15 mm diam. stout, woody near base, weakly angled to terete, devoid of foliage on lower and middle parts of stems; new stems 20.0–800.0 × 2.5–10.0 mm, fleshy, rigid, initially ± square, prominently angled, becoming ± terete with age, bases much covered in leaf abscission scars, middle and upper portion leafy. Leaves coriaceous, fleshy, green to dark green, rosette-leaves absent, stem leaves withering with age, variable in size and shape; petiole distinct, 2.0–50.0 × 1.2–5.3 mm, decurrent, weakly to prominently channelled, sometimes broadly winged, often with a broadly sheathing base; lamina variable 16.2–120.0 × 3.0–46.3 mm decreasing in size toward inflorescences, linear, linear-lanceolate, lanceolate, obovate, oblong, obdeltoid, oblanceolate to broadly elliptic, rarely spathulate; apex acute, subacute to obtuse, usually tridentate, rarely praemorse; margin coarsely and regularly to irregularly dentate, dentate to biserrate, sometimes deeply incised, finely denticulate or subentire; teeth usually protruding beyond leaf outline; in 10–60 pairs, up to 4 mm deep, increasing in size toward apex, sometimes with sporadic larger teeth randomly appearing from about the middle portion of lamina margin, or with teeth arranged ± evenly along lamina margin; base broadly cuneate tapering, sometimes extending into a broad petiole wing. Inflorescences racemose, 20.4–90.5 mm long at fruiting, rachis 0.7–2.35 mm diam., terminal and lateral, usually leaf-opposed, often long-persistent, pedicels 3–10 mm long at fruiting, erecto-patent. Flowers 3.0–4.2 mm diam., fragrant. Sepals 4, saccate, pale to dark green with a broad white, ± undulose margin; lateral sepals broad, 0.5–1.4 mm diam., orbicular, obovate to broadly obovate, ± overlapping at base, apex rounded to obtuse, abaxial surface often hairy, hairs 0.1–0.4 mm long, eglandular or with glandular tip, mostly shedding at anthesis except near base, median sepals 0.5–0.9 mm diam., broadly elliptic, pale to dark green with a broad white, ± undulose margin, apex rounded to obtuse, abaxial surface glabrous. Petals white, 1.3–2.2 × 1.6–2.3 mm, spreading, claw 0.4–0.9 mm long; limb obovate, obovate-spathulate to orbicular, apex obtuse to rounded often slightly emarginate, margins smooth, sometimes weakly undulose. Stamens 4, filaments 1.2–2.0 mm long, white; anthers 0.3–0.5 mm long, yellow. Ovary 1.1–1.8 × 0.6–1.3 mm, ovate, broadly ovate to elliptic, green-brown, apex round or subacute; style 0.11–0.4 mm long, cylindrical; stigma 0.2–0.5 mm diam. Nectaries 4, 0.2–0.3 × 0.1–0.15 mm, narrow-oblong to deltoid, pale translucent green. Silicles cartilaginous when fresh, coriaceous when dry, 2.6–4.8 × 1.7–3.4 mm, elliptic, rhomboid (rarely orbicular-rhomboid), apex acute to rounded, valves green maturing grey-green to straw-yellow, glabrous, not winged; style 0.3–0.6 mm long, exserted. Seeds 2, 1.2–1.9 × 0.8–1.4 mm, narrowly to broadly ovoid, brown to orange-brown, not winged. FL Aug–Jun. FR Sep–Jul.

#### Representative Specimens.

**Kermadec Islands**: Herald Islets, Dayrell Island, 17 May 2011, P. J. de Lange K911, (AK 326657); Herald Islets, Napier Island, 18 May 2011, P. J. de Lange K894, (AK 326586);Curtis Island, n.d., R. E. A. Shakespear s.n., (AK 4465); Curtis Island, November 1900, F. Shakespear s.n., (AK 128467); 18 July 1969, Curtis Island, W. R. Sykes 842/K, (CHR 193786); Curtis Island, 18 July 1969, W. R. Sykes 843/K, (CHR 193787); L’Esperance Rock, 26 May 2011, P. J. de Lange K859, (AK 326039). **New Zealand (Three Kings Islands).** Three Kings, November 1889, T. F. Cheeseman s.n., (AK 4466);Great Island (Manawa Tawhi), 1 January 1948, G. T. S. Baylis s.n., (AK 24128); South-West Island, 10 January 1950, G. T. S. Baylis s.n., (OTA 3804); Hole in the Wall Rock, 1 December 1983, A. E. Wright 6072, (AK 173005);Hinemoa Rock, 30 November 1983, A. E. Wright 6062, (AK 172998); Arbutus Rock, 1 December 1983, A. E. Wright 6074, (AK 173007);West Island, 29 November 1983, A. E. Wright 6060, (AK 172996). **New Zealand (North Island).** Motuopao Island, 11 May 1994, L. J. Forester s.n., (AK 294641); Matapia Island, 21 May 1993, L. J. Forester s.n., (AK 212201); Poor Knights Islands, Tawhiti Rahi, Hope Point, 26 April 1991, A. E. Wright 11492, (AK 201743); Poor Knights Islands, Aorangi Island, Crater Bay, 13 January 1978, G. N. Park s.n., (CHR 385039, WELT SP077730); Poor Knights Island, Tunnel Island (Aorangaia), 17 November 1933, L. B. Moore s.n. & L. M. Cranwell, (AK 100093); Mokohinau Islands, Stack “H”, 10 November 1993, P. J. de Lange 2643, (AK 226975, CANU, CHR, WAIK); Mokohinau Islands, Maori Bay Island (Hokoromea), 2 January 1984, E. K. Cameron 2664, (AK 273235); Mokohinau Island, Knights Group, Stack “D”, 13 November 1993, P. J. de Lange 2620, (AK 226964, CHR); Mokohinau Islands, Motuharakeke Island, 15 November 1993, P. J. de Lange 2662, (AK 226984 CHR, WAIK); Mokohinau Islands, Fanal Island (Motukino), 22 May 1979, A. E. Wright 3152, (AK 150595 WELT SP077068); Hen & Chicken Islands, Hen Island (Taranga), May 1880, T. F. Cheeseman s.n., (AK 4469); Little Barrier Island, December 1898, T. F. Cheeseman s.n., (AK 4470); Little Barrier Island, West Landing, 6 Oct 1945, B. Molesworth s.n., (AK 100091); Great Barrier Island (Aotea Island), Grey’s Archipelago, January 1868, T. Kirk s.n., (AK 11428, WELT); Great Barrier Island (Aotea Island), Broken (Pig) Islands, Mahuki Island, 2 January 1985, A. E. Wright 6883, (AK 171283); Oaia I, October 1953, Mr [sic] Wightman s..n., (AK 37510). Cuvier Island (Repanga), October 1895, T. F. Cheeseman s.n., (AK 4467, AK 221114, AK 221115, AK 247259); Mercury Island, Middle Island, 15 November 1983, E. K. Cameron 2536, (AK 272908); Mercury Bay, Motukorure (Centre Island), 5 March 1989, G. A. Taylor s.n., (AK 229759); Alderman Islands, Hongiora, May 1972, D. J. Court s.n. & A. Hardacre, (AK 131204); Motukaramarama Island, 30 August 1983, A. E. Wright5800, (AK 167081, CHR); South Auckland, Ngatutura Point, Shag Rock, 3 September 1988, P. J. de Lange 53, (CHR 462622);South Auckland, Albatross Point, Waioioi Reef, 15 September 2006, A. M. Brandon s.n. & D. W. Smith, (AK 297502); Bay of Plenty, Karewa Island, 26 April 2007, P. B. Cashmore s.n., S. J. Crump & J. Heaphy, (AK 299140); Bay of Plenty, Mt Maunganui, 1942, M. E. Sexton s.n., (AK 249218); East Cape Island (Whangaokeno), n.d.,L. Cockayne 9214, (AK 100089); East Cape, Waihau Bay, Gable-end Foreland, n.d., Hill s.n., (WELT SP023196). Taranaki, Sugar Loaf Islands, Motumahunga (Saddleback I.), 24 January 1989, G. A. S. Taylor s.n., (AK 295695); Hawke’s Bay, Cape Kidnappers, Black Reef, 19 November 1933, W. R. B. Oliver s.n., (WELT SP027655); Porirua, Mana Island, 1916, B. C. Aston s.n., (WELT SP027653); Porirua, Titahi Bay, n.d., B. C. Aston s.n., (WELT SP027652);South Wellington Coast, Cape Terawhiti, November 1908, B. C. Aston s.n., (AK 4468, AK 221113, WELT SP027657); South Wellington Coast, Mouth of the Karori Stream, 1 March 1931, W. R. B. Oliver s.n., (WELT SP027656); South Wellington Coast, Tongue Point, n.d., W. R. B. Oliver s.n., (WELT SP09712), Wellington, Seatoun, 28 February 1937, W. R. B. Oliver s.n., (WELT SP09711). **New Zealand (South Island)** Wharariki Beach, Archway Islands, Richard Seddon Island, 10 Febraury 2003, S. Courtney s.n., (CHR 551339); Wharariki Beach, north Nguroa Island, 10 February 2003, S. Courtney s.n., (CHR 551338). Marlborough, Pelorus Sound, Duffers Reef, 23 May 2001, S. Courtney s.n., (CHR 552377); Marlborough, Pelorus Sound, Bird Island, 21 May 2001, S. Courtney s.n., (CHR 552381); Marlborough, Titi Island, 27 January 1981, G. Y. Walls s.n., (CHR 416807); Marlborough, Stephens Island, 13 May 1957, M. E. Gillham s.n., (CHR 111512); Marlborough, Chetwode Islands, The Haystack, 29 March 1984, A. E. Wright 6435, (AK 174701); Marlborough, Chetwode Islands, Nukuwaiata Island, 29 March 1984, A. E. Wright 6450, (AK 174716); Marlborough, Chetwode Island, Sentinel Rock, 26 March 1984, A. E. Wright 6347, (AK 174639); Northern Brother Island, June 1963, G. Collett s.n., (CHR 141950); Queen Charlotte Sound, n.d.,J. H. Macmahon s.n., (AK 4471). **Chatham Islands.** Mangere Island, Mangere Island Nature Reserve, 14 February 2006, P. J. de Lange CH564 & P. B. Heenan, (AK 295979).**Cultivated (New Zealand)**: Lincoln, ex Ngatutura Point, Landcare Research experimental nursery, 26 January 2010, P. B. Heenan s.n., CHR 609811.

#### Distribution

**Figure 76.**
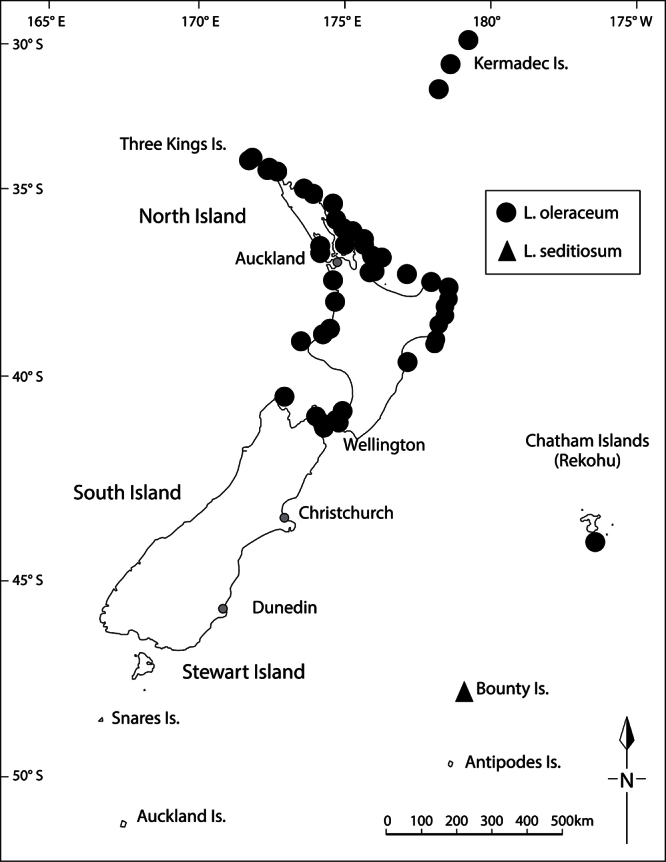
Distribution of *Lepidium oleraceum* and *Lepidium seditiosum*.

**(Fig. 76).** Endemic. Kermadec Island (Dayrell, Napier and Curtis islands). New Zealand, Three Kings, North, South (North-west Nelson and Marlborough Sounds and Chatham (Mangere Island) islands.

#### Recognition.

Although extremely variable with respect to stature, leaf shape, size and dentition, *Lepidium oleraceum* is consistently recognised by the glabrous pedicels and by the mature silicles which are elliptic, rhomboid (very rarely orbicular-rhomboid) and with an acute to rounded apex that leaves the style remnant standing proud above the silicle apex (Fig. 75). Silicles often give the false impression of being notched, something that happens when the valves are over mature and so have started to dehisce at the apex. *Lepidium oleraceum* often forms a widely spreading but erect bushy plant whose stems are long persistent and, while slowing in growth over winter, do not die back to the rootstock as do all other New Zealand members of *Lepidium oleraceum* complex except *Lepidium castellanum* and, on occasion, *Lepidium oblitum* and *Lepidium panniforme*. *Lepidium oleraceum* also lacks the distinct rosette leaves seen in all other members of the *Lepidium oleraceum* complex except *Lepidium castellanum*.

#### Ecology.

Much has been written about the ecology of *Lepidium oleraceum*, of which the summary provided by Norton et al. (1997) still applies despite our segregation here of 10 new species from it. *Lepidium oleraceum* is intimately associated with sea-bird trails, roosts and nesting grounds. Plants are dependent on these birds not only for the habitats they create through disturbance but also the nutrients they bring from the sea in the form of guano and discarded or regurgitated food, and because these birds assist with seed dispersal. Seed dispersal is most readily facilitated by birds because *Lepidium oleraceum*, as well as growing around bird nests and burrows, is often used for nesting material. In this way, the seeds, which are mucilaginous when wetted, can easily stick to the feathers and feet of birds.

*Lepidium oleraceum* tends to be short-lived, with individual plants rarely persisting for more than three years in the wild and up to five in cultivation. It has also been observed that some wild populations are prone to sudden crashes, and may even completely die out, only to reappear some years later. It is unknown whether the reappearance is from a residual seed bank or from seeds dispersed by birds from another site. Plants may even behave as annuals in some locations because, despite its predilection for open, drought-prone coastal habitats, *Lepidium oleraceum* does not relish drought.

Norton et al. (1997) stressed the importance of sea birds in providing the nutrient regime necessary to sustain the species, arguing that the loss of sea bird breeding grounds from large parts of the country best explains the rapid demise of *Lepidium oleraceum*. However, while that view is probably still valid, some anomalies remain to be explained. For example, *Lepidium oleraceum* appears to have always been scarce on the Three Kings Islands, which are naturally predator-free and harbour large sea bird breeding colonies. On those islands, *Lepidium* is confined to white-fronted tern (*Sterna striata* Gmelin, 1789) and red-billed gull (*Chroicocephalus scopulinus* (Forster, 1844)) nesting grounds, and is absent from all other sea bird colonies, including the heavily burrowed ground left by breeding black-winged petrel (*Pterodroma nigripennis* Rothschild, 1893). This requires further study, as on many Hauraki Gulf islands *Lepidium oleraceum* is usually absent from tern and gull nesting sites, and more usually associated with petrels such as grey-faced (*Pterodroma macroptera gouldi* (F.W.Hutton, 1869)), black-winged, and Pycroft’s (*Pterodroma pycrofti* Falla, 1933), or with shearwaters (*Puffinus* spp.), white-faced storm petrels (*Pelagodroma marina* Latham, 1790) and common diving petrels (*Pelecanoides urinatrix* (Gmelin, 1789)). On some islands supporting these birds and gannets (*Morus serrator* Gray, 1843), *Lepidium oleraceum* is found only near gannet colonies. Interestingly, *Lepidium oleraceum* was regarded as one of the most common plants on the foreshore of Aorangi Island, Poor Knights until the removal of pigs in 1936 (Reynolds 1988; de Lange and Cameron 1999), yet now, despite the massive sea bird colonies covering that island, this species is scarce, being mostly confined to cliff habitats and recent slip scars (de Lange and Cameron 1999).

These patterns suggest that the overriding need for this species is a combination of nutrient rich soils and frequent habitat disturbance to keep sites free from competition. Yet why *Lepidium oleraceum* is so uncommon on some naturally predator free islands which appear to meet these criteria, and why it seems to show preferences for particular bird nesting associations that can vary from island to island remains to be elucidated.

It is also possible that *Lepidium oleraceum* was deliberately cultivated and utilised as a pot herb by Māori who knew the species (and probably those allied to it) as “nau” (see de Lange and Norton 1996). The early writings of Banks, Solander and Forster make frequent mention of its abundance near Māori dwellings and settlements and that these people often directed Cook’s shore foraging crews to places where it could be collected (Beaglehole 1962, 1967, 1968; Forster 1777; Hoare 1982; de Lange and Norton 1996). While it could be argued that *Lepidium oleraceum* flourished around coastal human habitations because these were sites of frequent disturbance and nutrient enrichment, the fact that Māori had a specific name for the plant and knew that it was edible suggests that they may also have cultivated it, a possibility that needs further critical ethnobotanical investigation.

#### Conservation Status.

Prior to this revision, *Lepidium oleraceum* was assessed as ‘Threatened/Nationally Vulnerable _CD, EF, RR, Sp_’. Following the segregation of *Lepidium oleraceum* s.l. in this paper a new threat listing of *Lepidium oleraceum* s.s. is now necessary. As circumscribed here, *Lepidium oleraceum* is now confined to the Kermadec Islands, North Island (and adjacent offshore islands), northern South Island and Chatham Islands (Mangere Island only). Within this area, *Lepidium oleraceum* is only “common” (i.e. occurring in numbers greater than 200 individuals) on a very few islands and islets, notably Mahuki Island west of Great Barrier (Aotea Island), Karewa Island in the Bay of Plenty, Waioioi Reef off the west coast of Albatross Point, and on several islands (notably Stephens Island) within the Marlborough Sounds. It is now a very uncommon species elsewhere within this range, with most known populations comprising 50 or less mature individuals. At all known sites, populations are naturally prone to the sudden collapse and boom cycles briefly described by Norton et al. (1997) and Norton and de Lange (1999). Also, because there are few places where the *Lepidium oleraceum* s.s. is being closely monitored, available data on natural population fluctuations is insufficient from which to provide an overall general assessment of total population size and stability. Irrespective, the species is still very widespread in northern New Zealand and the Marlborough Sounds, and whilst most of the remaining populations are small (i.e. < 50 mature plants) this may reflect a natural state of affairs, as past accounts of this species’ abundance do seem to have been exaggerated (see comments by de Lange and Norton 1996). Nevertheless, in the absence of key data on trend and rate of decline, it is difficult to provide a meaningful threat assessment, though the situation is not so difficult as to recommend a conservative assessment of ‘Data Deficient’. Sufficient information exists to attempt a threat listing here, from which we recommend a conservation listing of ‘Threatened/Nationally Vulnerable’ applying criterion ‘C’ of Townsend et al. (2008, p. 20–21). This assessment is based on data collected and stored by the Department of Conservation-sponsored coastal cress recovery team (P. I. Knightbridge pers comm.), from which we estimate the total population for this species at c. 3000–3500, and a rate of decline of c.10% over the next 10 years. As few wild plants last longer than three years, we have opted for (as recommended by the manual (Townsend et al. 2010)) the rate of loss rather than generation time. To that assessment the qualifiers ‘CD’ (Conservation Dependent), ‘EF’ (Extreme Fluctuations), ‘RR’ (Range Restricted) and ‘Sp’ (Sparse) still apply but we recommend the addition of ‘DP’ (Data Poor) to reflect the overall absence of trend data.

#### Hybridism.

Putative hybrids have between *Lepidium oleraceum* and *Lepidium flexicaule* and *Lepidium obtusatum* are discussed under those species.

### Lepidium oligodontum sp. nov.

urn:lsid:ipni.org:names:77129260-1

http://species-id.net/wiki/Lepidium_oligodontum

*A L. oleraceo habitu renascenti, habenti periodo distincto rosulato (folia rosularum anguste spathulata, cuneata, obdeltoidea vel obovata – raro ellipto-lanceolata), habitu plantae maturae decumbenti ramulis extensis foliis integris vel leviter dentatis (dentes solum distales), floribus 2–4(–6)-staminatis, siliculis manifeste turgidis plerumque late orbiculatis leniter alatis emarginatis vel integris ad apicem et serie DNA differt*.

#### Holotype.

**Figure 77.**
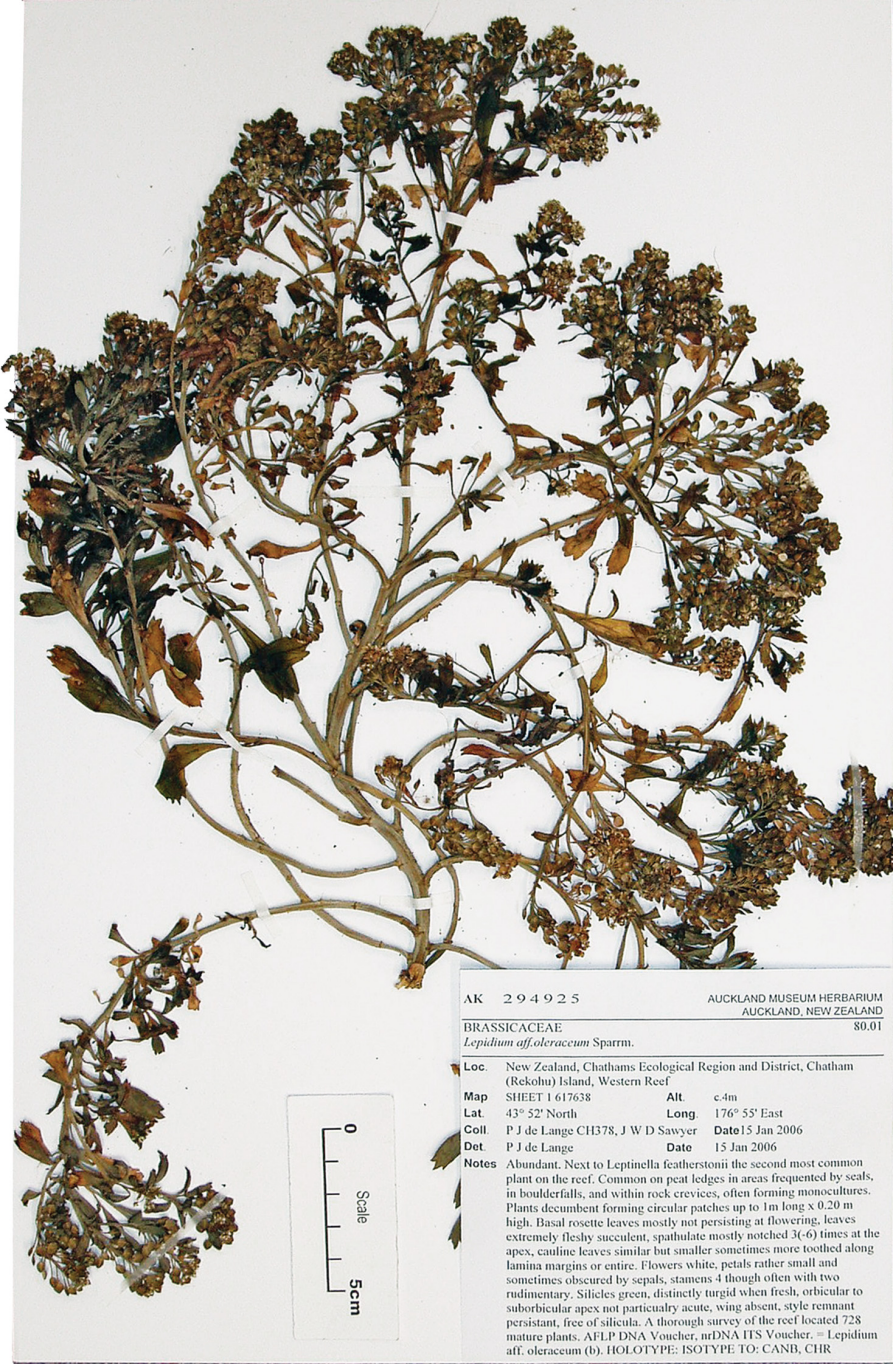
Holotype of *Lepidium oligodontum* de Lange et Heenan.

**Chatham Islands (Fig. 77):** Chatham (Rekohu) Island, Western Reef, 15 January 2006, P. J. de Lange CH378 & J. W. D. Sawyer, AK294925! Isotypes CANB!, CHR!

#### Eytmology.

The name ‘*oligodontum*’ from the Latin meaning ‘few teeth’ refers to the leaves of this species which may be deeply toothed, sparingly toothed near the apex or entire, sometimes with all three conditions on the same plant. Plants with deeply toothed leaves are uncommon, whilst those with sparingly toothed leaves are more usual.

#### Description

**Figure 78.**
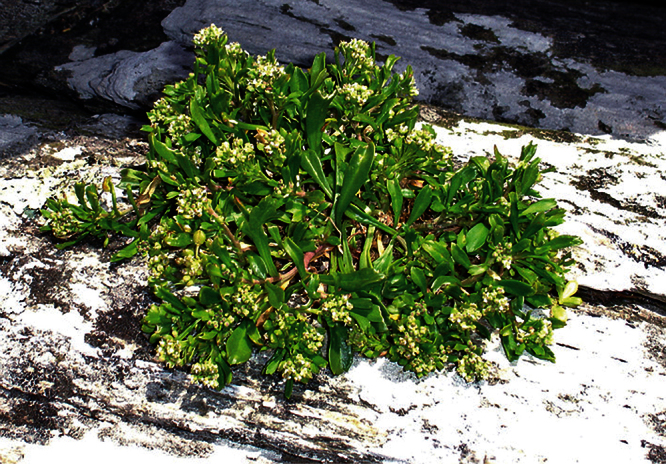
Wild plant of *Lepidium oligodontum* on Te Wakaru Island, showing decumbent growth habit.

**Figure 79.**
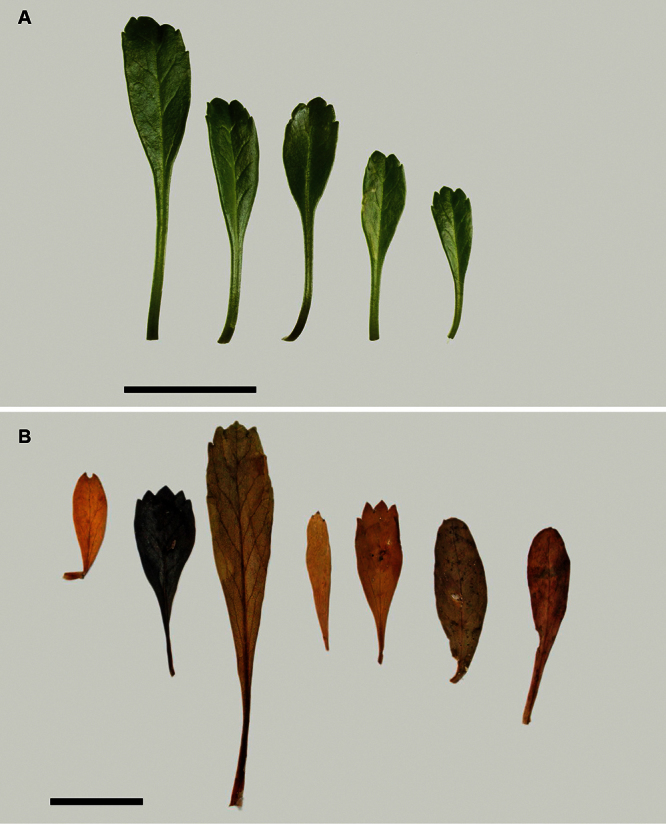
(**A**) (from left to right) variation in rosette and basal-stem leaves from a single plant of *Lepidium oligodontum*; (**B**) (from left to right) leaf variation within populations from The Sisters (Rangitatahi), Western Reef, Point Somes, (Rekohu), Moriori Creek (Rekohu), Star Keys (Motuhope), Rangatira and Antipodes Island. Scale bars = 20 mm.

**Figure 80.**
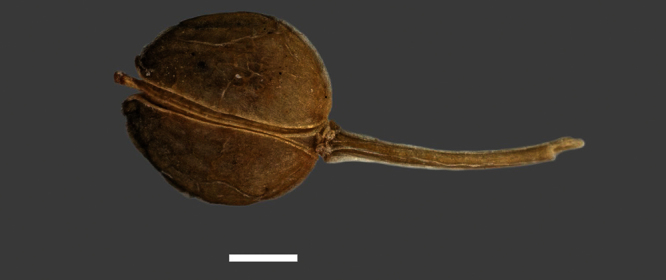
Mature silicle of *Lepidium oligodontum*. AK 295634. Scale bar = 1 mm.

**(Figs 78–80).** Tap-rooted, decumbent, rather flaccid, pungent-smelling, summer-green, succulent, perennial herb forming sparse to densely leafy, circular ± flat masses up to 1 m diam., and arising from stout, semi-circular, greyish-white or reddish-grey (when exposed) rootstock 6.0–30.0 mm diam. Tap root woody, up to 0.2 m long, deeply descending. Plants dying down to rootstock and/or previous seasons stem nodes, at fruit set or soon thereafter. Stems decumbent, widely spreading, up to 0.8 m long and 3.20–4.56(–5.20) mm diam., ± woody throughout, ± square to somewhat angular-spheroidal in cross-section and prominently ridged on angles (this especially conspicuous when dry), dark reddish-green to dark green when fresh, drying dull grey; stems usually heavily branched from base, branches and branchlets numerous, prostrate, widely spreading, and usually very leafy; basal portion of stems, glabrous. Leaves glabrous, succulent, dark green, green to yellow-green at senescence turning yellow. Rosette leaves 5–10(–14), mostly present in autumn – early spring but not persisting (very rarely so) at fruiting; petioles distinct up to 70 × 2 mm, flat or slightly concave in cross-section, succulent; lamina narrowly spathulate, cuneiform, obdeltoid, obovate or rarely elliptic-lanceolate, up to 60 × 22 mm, margins entire, or sparingly dentate in upper ⅓, if teeth present then in 1–3(–5) pairs running to and including the usually tridentate apex, basal teeth often asymmetric, base narrowly cuneate, cuneate to attenuate. Middle stem leaves persistent or not at fruiting; petiole usually distinct (rarely not) up to 14.0 × 1.13 mm, mostly flat in cross-section, sometimes slightly concave, succulent; lamina spathulate, cuneiform, linear-cuneiform, oblanceolate, narrowly ovate, to narrowly obovate, or rarely orbicular, 10.6–22.8(–33.2) × 5.4–9.2 (–17.5) mm; margins entire, or sparingly dentate with 1–2–3(–5) pairs of teeth in upper ⅓ apex usually tridentate, basal teeth often asymmetric, lamina base narrowly attenuate, attenuate, cuneate or rarely acute. Upper stem leaves usually without a distinct petiole, petiole if present 1.0–3.6 mm, flat or slightly concave; lamina narrowly cuneiform, oblanceolate, or narrowly obdeltoid, 7.6–10.8(–11.9) × 2.7–3.1(–3.5) mm; margins entire or weakly dentate to deeply incised, if dentate or incised then with 1(–2) often asymmetrical teeth present in the upper ⅓, apex entire or tridentate, lamina base cuneate to narrowly cuneate. Racemes (5.0)–9.7(–28.9) mm long, usually congested, elongating up to 60 mm at fruiting, terminal and axillary; rachis and pedicels glabrous; pedicels, erecto-patent to patent,0.82–1.00(–2.08) mm, 2.1–3.5(–6.1) mm long at fruiting. Flower buds dark green to green, apex glabrous. Flowers sweetly fragrant, 1.2–1.5(–2.2) mm diam. Sepals 4, saccate, pale to dark green with a broad white, ± undulose margin, pale to dark green with a broad white, ± undulose margin, deeply concave, adaxially weakly keeled or not; lateral sepals 0.9–1.4 × 0.8–1.2 mm, broadly ovate to oblong, ± overlapping at base, apex rounded to obtuse, adaxial surface mostly glabrous sometimes diffusely papillate, abaxial surface often hairy, hairs patent, weakly flexuous, 0.1–0.4 mm long, eglandular, mostly shedding at anthesis except near base; median sepals 1.0–1.4 × 0.9–1.2 mm, broadly ovate to oblong, apex rounded to obtuse, adaxial surface glabrous, abaxial surface usually glabrous, rarely sporting a small tuft of patent, eglandular, flexuous hairs 0.1–0.2 mm long. Petals usually present (occasionally absent) usually equal to or slightly overtopping sepals (rarely > sepals), white, 0.8–1.6(–1.9) × 0.6–1.2(–1.8) mm, patent, clawed; limb broadly obovate, apex obtuse, rarely retuse. Stamens 2–4(–6), equal. Anthers c.0.14 mm long. Pollen bright yellow. Nectaries 2, subulate, 0.32 mm long. Silicles somewhat fleshy and distinctly turgid when fresh, on drying collapsing to form a coarse reticulum, broadly orbicular to orbicular, (3.0)–3.5(–3.8) × (2.8–)3.0–3.2(–3.8), margin slightly winged, sometimes more so toward apex, apex not or very slightly notched, base cordate (rarely truncate), valves green maturing yellow-green, glabrous; style 0.2–0.8(1.2) mm long, free from the narrow wing, equal to or slightly exceeding the notch; stigma 0.4–0.6 mm diam. Seeds 2, ovoid to suborbicular, light red-brown or red-brown, not winged, 0.84–0.98 × 0.80–1.00 mm. FL. Dec–Mar. FR. Dec–Apr.

#### Representative Specimens.

**Chatham Islands:** Rangitatahi (Sisters) 29 January 2005, R. M. Bellingham s.n., (AK 290289); Rekohu, Point Somes, 10 January 2006, P. J. de Lange CH393, (AK 294941); Rekohu, Rock Stacks, Point Somes, 10 January 2006, P. J. de Lange CH390, (AK 294938); Rekohu, ‘the Clears’ near Owenga, April 1979, M. A. Ringer s.n., (AK 150010); Rekohu, between Point Gap and Tuku, November 1980, A. M. Ringer s.n., (AK 170636); Rekohu, Coastal cliffs south of Tuku River, November 1987, G. A. S. Taylor s.n., (AK 235255); Rekohu, Otauwae Point, 12 January 2006, P. J. de Lange CH417, (AK 295634, CANB, CHR); Rekohu, South Moriori Creek, 12 February 2006, P. J. de Lange CH801, (AK 299967); Rekohu, Point Gap, 12 February 2006, P. J. de Lange CH802, (AK 299966); Rekohu, Te Wakaru Island, 13 January 2006, P. J. de Lange CH431 & J. W. D. Sawyer, (AK 295160, CHR, MEL); Rekohu, unnamed point south-west of Ocean Bay, 14 January 2006, P. J. de Lange CH441 & J. W. D. Sawyer, (AK 295120); Western Reef 15 January 2006, P. J. de Lange CH380 & J. W. D. Sawyer, (AK 294927); 15 January 2006, P. J. de Lange CH379 & J. W. D. Sawyer, (AK 294926); Star Keys (Motuhope), 15 December 2005, A. Baird s.n., (AK 295162); Rabbit Island, 14 February 2006, P. J. de Lange CH675 & P. B. Heenan, (AK 296753, CHR); Rangatira (South-East Island), 1 January 1970, B. G. Hamlin 1703, (WELT SP042706, WELT SP049930A, WELT SP049930B); Tarakoikoia (The Pyramid) 27 February 1993, G. A. S. Taylor s.n., (AK 228962). **Antipodes Islands:** Antipode Island, January 1909, B. C. Aston s.n., (WELT SP027627); Antipode Island, Anchorage Bay, 7 November 1995, G. A. S. Taylor s.n., (AK 233772).

#### Distribution

**(Fig. 63).** Endemic. New Zealand, Chatham and Antipodes islands. On the Chatham Islands, it is known from Rekohu and South West (Rangatira) Island, and from all of the outlying islets and vegetated rock stacks except Mangere, Little Mangere and the Forty-fours. It is apparently absent from Rangiauria (Pitt Island). On the Antipodes Islands, *Lepidium oligodontum* is known only from Antipodes Island, where it is has a very restricted occurrence at the northern end of that island (Godley 1989).

#### Recognition.

*Lepidium oligodontum* is recognised by the combination of having a decumbent rather than erect growth habit (Fig. 78), glabrous stems, branchlets and pedicels, leaves which are either sparingly toothed or entire (Fig. 79), flowers with 2–4(–6) stamens, and silicles mostly broadly orbicular to orbicular, slightly winged, mostly cordate-based, and that are distinctly swollen (turgid) when fresh and whose apices are slightly notched (Fig. 80). Of the New Zealand species, *Lepidium oligodontum* is unusual for the variability of stamen number. In some populations plants are found that have either two or four stamens, whilst others may show a range from two to four (rarely six). Some populations consistently have four stamens whilst others show a grade from two to six.

#### Ecology.

*Lepidium oligodontum* is a strictly coastal, island endemic inhabiting richly manured, frequently disturbed habitats. On Rekohu it is extremely uncommon and now virtually confined to coastal cliffs, near-shore rock stacks, and, very occasionally, coastal turf communities, especially in places frequented by New Zealand fur seals (*Arctocephalus forsteri* Lesson 1828). In these habitats it is usually associated with *Disphyma papillatum*, *Apium prostratum* subsp. *denticulatum* P.S.Short, *Crassula moschata* G.Forst., *Dichondra* spp., *Hebe chathamica*, *Leptinella potentillina* F.Muell., and *Puccinellia chathamica*. In a few sites, such as at Point Somes and Ocean Bay, *Lepidium oligodontum* grows intermingled with *Lepidium flexicaule* and hybrids between both species have occasionally been collected where they co-exist. Habitats occupied by *Lepidium oligodontum* vary on the outer islands, islets and rock stacks of the Chatham archipelago. It is an occasional associate of coastal turf and cliff vegetation on islands such as South East (Rangatira). On the other outer islands (e.g., The Sisters (Rangitatahi), Star Keys), islets (e.g., The Pyramid (Tarakoikoia)), reefs and rock stacks *Lepidium oligodontum* is a prominent species of the distinctive guano-dependent vegetation that has developed in the presence of New Zealand fur seals, and sea birds such as the albatrosses (*Diomedea sanfordi* Murphy, 1917, *Thalassarche eremita* (Murphy, 1930), *Thalassarche bulleri*), northern giant petrel (*Macronectes halli* (Mathews, 1912)), Chatham Island fumar prion (*Pachyptila crassirostris pyramidalis* C.A.Fleming, 1939), and white-fronted tern (*Sterna striata* Gmelin, 1789) (Aikman and Miskelly 2004). In these guano-enriched habitats, *Lepidium oligodontum* is usually found growing in close association with the woody shrub, *Leptinella featherstonii* F.Muell., and herb, *Senecio radiolatus* subsp. *radiolatus* (see de Lange and Sawyer 2008). On Rabbit Island, *Lepidium oligodontum* was found growing at the entrance to petrel (*Pterodroma* spp.) and shearwater (*Puffinus* spp.) burrows, sometimes in association with *Lepidium oblitum* and *Lepidium rekohuense*, and other coastal turf plants such as *Disphyma papillatum*. Detailed information on the habits of *Lepidium oligodontum* on the Antipodes is lacking, although herbarium specimens and people’s observations (G. A. Taylor and S. P. Courtney pers. comm.) note that it is seemingly restricted to a few coastal headlands where it grows at the top of steep cliffs in sites kept free of taller vegetation by wind and salt burn. Oddly, despite the abundance of sea birds and seals on the Antipodes, *Lepidium oligodontum* was not observed growing anywhere near sites frequented by these animals, leading us to infer that the habitat is similar to that occupied along the southern coastal cliffs of Rekohu, e.g., Moriori Creek and Otauwae Point.

*Lepidium oligodontum*, more than any other member of the *Lepidium oleraceum* complex, has a very seasonal growth pattern, with most vegetative growth occurring from late winter to summer. *Lepidium oligodontum* plants are scarcely visible during autumn and early winter because growth virtually ceases and most of the foliage, branches, branchlets and inflorescences wither away. During this time of ‘dormancy’, plants persist as minute leafy shoots clustered around the rootstock apex, or as a single leafy rosette.

It has also been observed that the annual growth cycle of *Lepidium oligodontum* seems to be dictated, perhaps more than any other New Zealand *Lepidium*, by the onset and spread of the reproductive phase of the oomycete *Albugo candida* (Pers.) Kuntze. The spore-bearing pustules of *Albudo candida* appear on wild and cultivated plants within weeks of the initiation of spring growth, and by late January/February have usually erupted across most of the stem leaves, flowers and fruits. Further vegetative growth from the host plant is either completely retarded or aborted. Observations of wild population of *Lepidium oligodontum* suggest that these seasonal eruptions of *Albugo candida* have a tremendous impact on this species’ vigour, and we have found that the intensity of these *Albudo candida* infections make it impossible to maintain *Lepidium oligodontum* in cultivation. It is not clear if the severity of these attacks in *Lepidium oligodontum* populations is natural or a consequence of other, as yet undetermined, external factors, or even if the strain of *Albudo candida* infecting *Lepidium oligodontum* is endemic to it (E. H. McKenzie pers. comm.). Whether indigenous, endemic or naturalized, this oomycete is now, at least, a critical constraint on the growing season, flowering and fruiting of this species, especially in what appear to be ecologically suboptimal sites. Field observations suggest that *Lepidium oligodontum* is more abundant and the plants clearly thriving in sites where nutrient levels remain high, such as on the guano-enriched Pyramid (Tarakoikoia), or within the seal colonies of the Star Keys and Western Reef (de Lange and Sawyer 2008). Despite the presence of *Albudo candida*, their growth season is longer and the plants more persistent than at any of the other Chatham Islands’ *Lepidium oligodontum* sites that we have been able to visit.

#### Conservation Status.

*Lepidium oligodontum* as *Lepidium* aff. *oleraceum* (b) (AK 208579; Antipodes – Chatham Islands) was listed in Appendix 2 of de Lange et al. (2009, p. 89) as “Threatened/Nationally Critical _DP, EF, RR_”. With its formal description and recognition, a reassessment of that conservation listing is now appropriate.

Aside from a few, small, isolated islands, islets and rock stacks in the Chatham archipelago and possibly Antipodes Island, trend data gathered by the New Zealand Department of Conservation over the last 15 years confirm that *Lepidium oligodontum*,though naturally prone to extreme population fluctuations, is now in final stages of a major terminal decline on Rekohu (A. Baird unpubl. data).

Based on our knowledge of this species’ ecology, its loss from Rekohu appears to be the result of an initial loss of habitat as key sea bird and seal populations on the larger islands of the group went extinct over the last 600 or so years (King 1989). Habitat loss has accelerated since the early 19th century following the settlement of Rekohu by Maori and Europeans whose impacts on the remaining seal and sea bird breeding grounds have been severe (Wills-Johnson 2008; King 2008). Land clearance and ongoing modification of the main Chatham Islands has also facilitated the spread of competing plants that were previously unknown there and which had been either deliberately or inadvertently introduced (de Lange et al. 2008; de Lange et al. 2011).

Another factor in the decline of this species is the oomycete *Albugo candida*,discussed above. It remains unclear whether *Albugo candida* truly poses a serious threat to indigenous New Zealand Brassicaceae, and in particular *Lepidium oleraceum* s.l. (Armstrong 2007). Nevertheless, our field observations and the remarks made by collectors on herbarium specimens note that *Albugo candida* strongly retards the growth of *Lepidium oligodontum* and that it continues to be a major factor in the decline and loss of many of the smaller populations on Rekohu. *Lepidium oligodontum* seems to be buffered from the severity of *Albugo candida* outbreaks only on less modified outer islands, islets and rock stacks of the Chatham Island archipelago and, presumably, the Antipodes Islands, where there are intact, functional guano- and marine mammal-based ecosystems (de Lange and Sawyer 2008). Furthermore, *Albugo candida* has prevented *Lepidium oligodontum* from being successfully cultivated beyond a single season or translocated to new sites (A. Baird, S. Benham, G. Davidson, J. Santos, T. Silbery, and R. Smith pers. comm.).

Aside from *Albugo candida*, some of the Rekohu populations of *Lepidium oligodontum* are also subjected to a diverse range of threats. These include browse by feral sheep (*Ovis aries* Linnaeus, 1758), cattle (*Bos primigenius taurus* (Linnaeus, 1758)) and possum (*Trichosurus vulpecula* (Kerr, 1792)). Possums, in particular, have been found avidly browsing *Lepidium oligodontum* plants growing on near-shore rock stacks at Point Somes and Te Koparuparu Bay on Rekohu.

Following extensive surveys of the Chatham Islands by the Department of Conservation during the summer of 2005/2006, 14 functional subpopulations were recognized, down from 28 known in 1996 (a decline rate of 50 % over 10 years). These gave a total population for that island group of 1321 adults, of which 748 (57%) were confined to one site, Western Reef. These figures exclude population data for The Sisters and The Pyramid (Tarakoikoia), which are privately owned and inaccessible to the Department of Conservation. To date, the few reports of *Lepidium oligodontum* from these islands suggest that it is “common” (R. Emberson and P. Schofield pers. comm.). Monitoring at Te Wakaru Island, which has an intact guano and marine mammal-based ecosystem, shows that the population there fluctuates greatly from year to year. Therefore, trend data needs to be gathered over several years before any evidence of decline can be distinguished from natural population “boom/bust” cycles. Little is known about the species’ status on the Antipodes. In part because these remote islands are infrequently visited, and then usually by ornithologists at times when our data for the Chatham Islands suggests *Lepidium oligodontum* was already undergoing its seasonal decline. For example, an ornithological party considered *Lepidium oligodontum* to be very uncommon there in 1995, seeing perhaps only a few tens of plants (G. A. S. Taylor pers. comm.). These were restricted to one site at Reef Point, where, because of their close proximity to a former castaway hut and the unusual habitat they occupied, it was though that the few plants seen may have been introduced there (G. A. S. Taylor pers. comm.). This contrasts somewhat to the observations published by Godley (1989) (a botanist) who visited the islands in 1969. Godley recorded *Lepidium oligodontum* (as *Lepidium oleraceum*) as being ‘common there [at Reef Point] on coastal rocks and occasionally on cliffs’, though he then went on to state that ‘it was not seen by any of the 1969 party at other possible localities’. Nevertheless, based on what we now know of the *Lepidium oleraceum* group, the possibility that *Lepidium oligodontum* is introduced to the Antipodes seems unlikely, and based on the historical observations summarised by Godley (1989, p. 546) it would seem that, if anything, observers were witnessing different stages of the ‘boom/bust’ cycles observed elsewhere on the Chatham Islands. Nevertheless, as the Antipodes Islands are so infrequently visited, it is important that future visits focus on gathering better data and setting up long-term monitoring of that island group’s *Lepidium oligodontum* population.

In summary, we consider that *Lepidium oligodontum* is more appropriately assessed as ‘Threatened/Nationally Vulnerable’ (criteria B(1/1) of Townsend et al. (2008). This is based on a population size that is likely to be between 1000 and 5000 mature individuals spread across the Chatham and Antipodes island groups (noting that our best available data (field season 2005/2006) recorded 1321 plants from those accessible locations on the Chatham Islands; that outside those areas on other islands in that group, such as The Sisters (Rangitatahi), Star Keys and The Pyramid (Tarakoikoia) there are likely to be at least as many individuals; and that there no reliable population data for the Antipodes Islands). Further, it would seem that, aside from the ongoing decline of what are effectively non-functional, residual populations on Rekohu, the species is secure on the outlying islands, islets and rock stacks of the Chatham Islands group where the ornithocoprophilous ecosystem remains intact and functional. Accepting the absence of hard data for the Antipodes Islands, observations by field parties suggests that *Lepidium oligodontum*, while less abundant there, is probably also secure due to the intact nature of the ornithocoprophilous ecosystem there.

To this recommended threat listing we suggest appending the following qualifiers ‘DP’ (Data Poor – to reflect uncertainty over the status of the species on the Antipodes Islands), ‘EF’ (Extreme Fluctuations – to reflect the wide population fluctuations experienced throughout a normal growing season), and ‘RR’ (Range Restricted – because *Lepidium oligodontum* is naturally confined to a geographically small part of the New Zealand Botanical Region).

### Lepidium panniforme sp. nov.

urn:lsid:ipni.org:names:77129261-1

http://species-id.net/wiki/Lepidium_panniforme

*A L. oleraceo habitu renascenti, habenti periodo distincto rosulato, foliis valde dentatis vel laceratis, siliculis late orbiculatis vel orbiculato-rhomboideis minute alatis ed emarginatis et serie DNA differt. A L. oligodonto et L. rekohuensi habitu suberecto vel erecto fruiticoso, foliis semper dentatis vel laceratis, floribus (2–)4-staminatis et serie DNA differt*.

#### Holotype.

**Figure 81.**
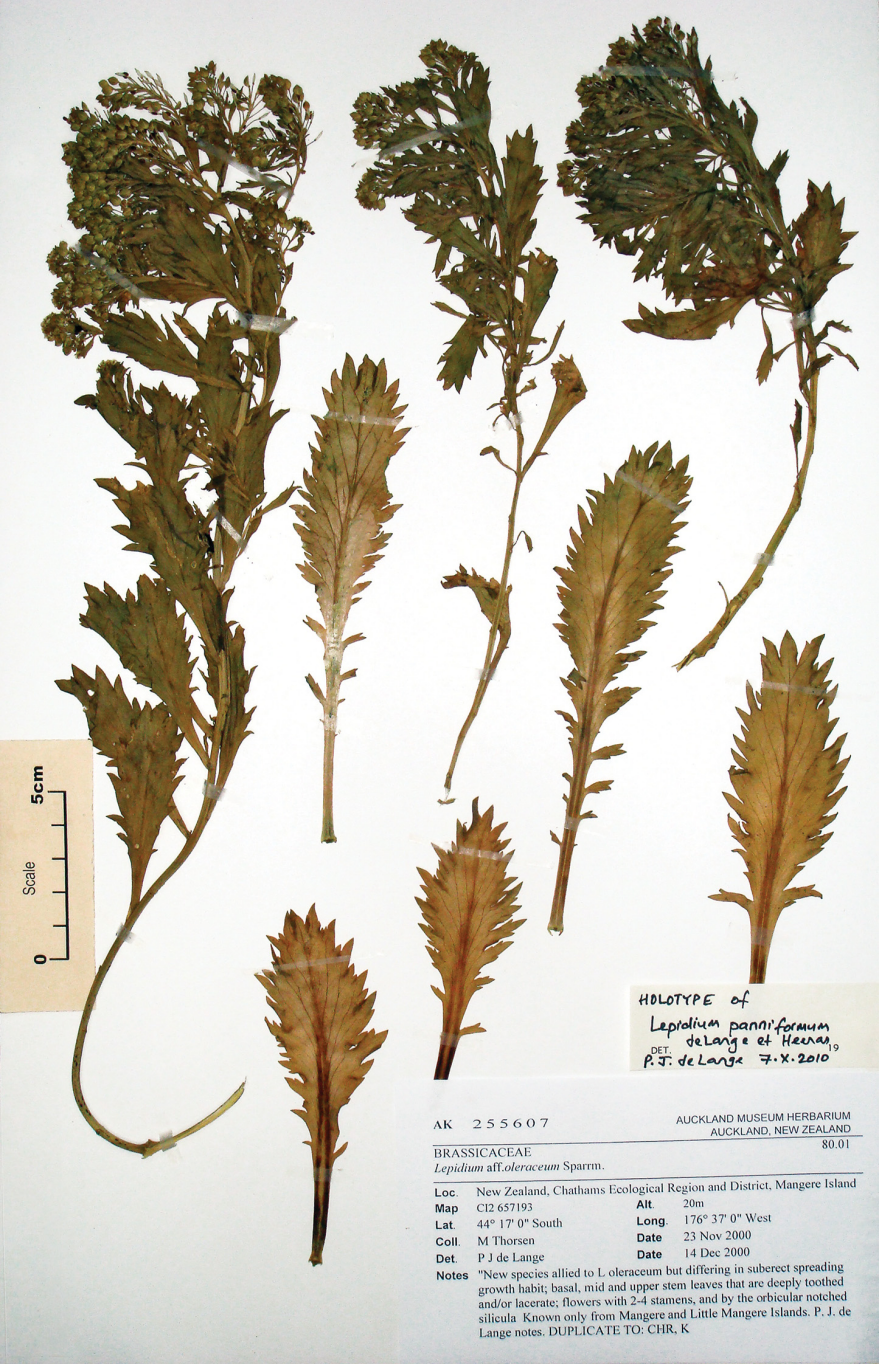
Holotype of *Lepidium panniforme* de Lange et Heenan.

**Chatham Islands (Fig. 81):** Mangere Island, Mangere Island Nature Reserve, Mangere Island, 23 November 2000, M. Thorsen, AK 255607! Isotype: CHR!, K!

#### Etymology.

The name “*panniforme*” from the Latin meaning “like a shredded rag” (N. G. Walsh pers. comm.) refers to the ragged appearance of the deeply lacerate, tattered basal and lower stem leaves characteristic of this species.

#### Description

**Figure 82.**
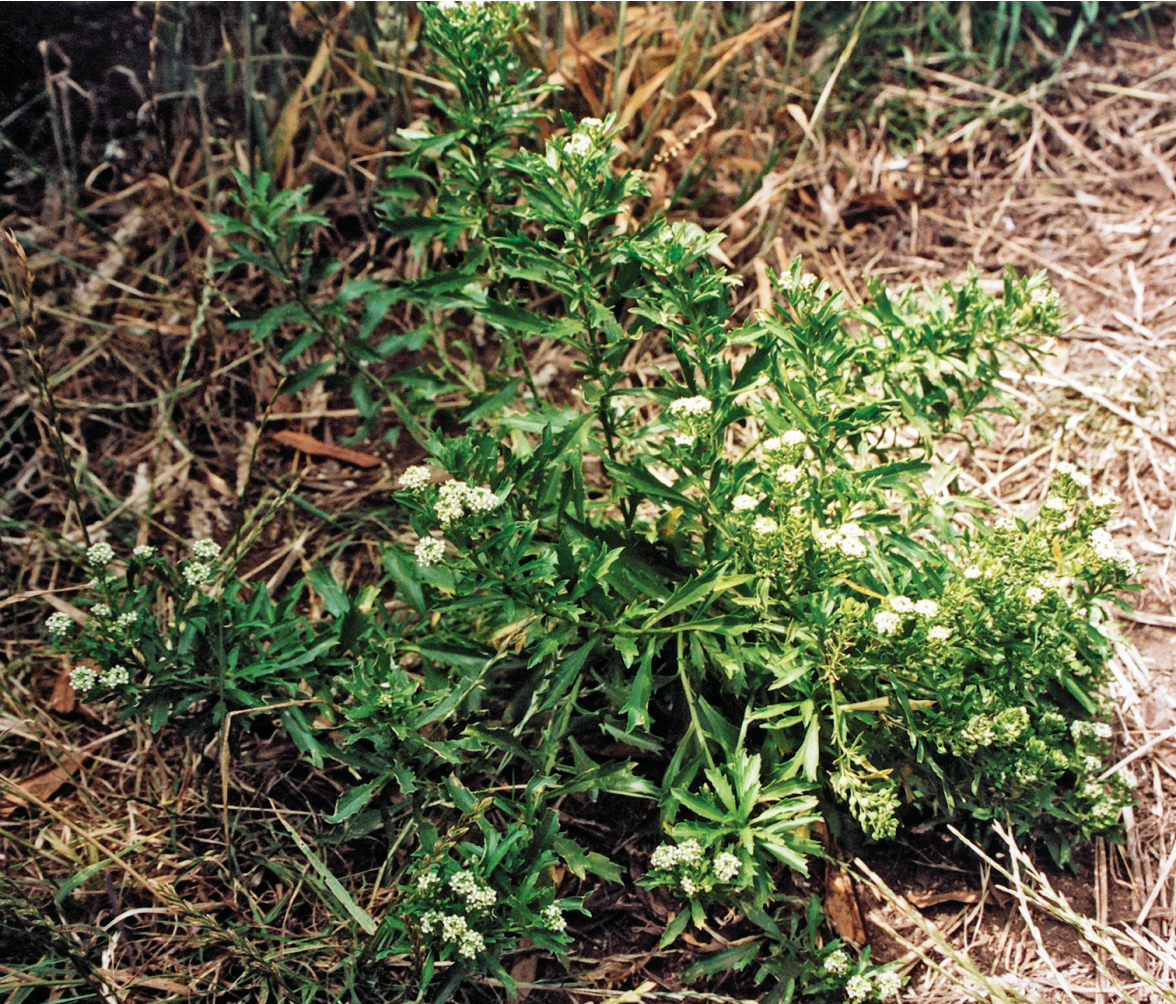
Wild plant of *Lepidium panniforme* on Mangere Island showing growth habit (image: B. Gibb).

**Figure 83.**
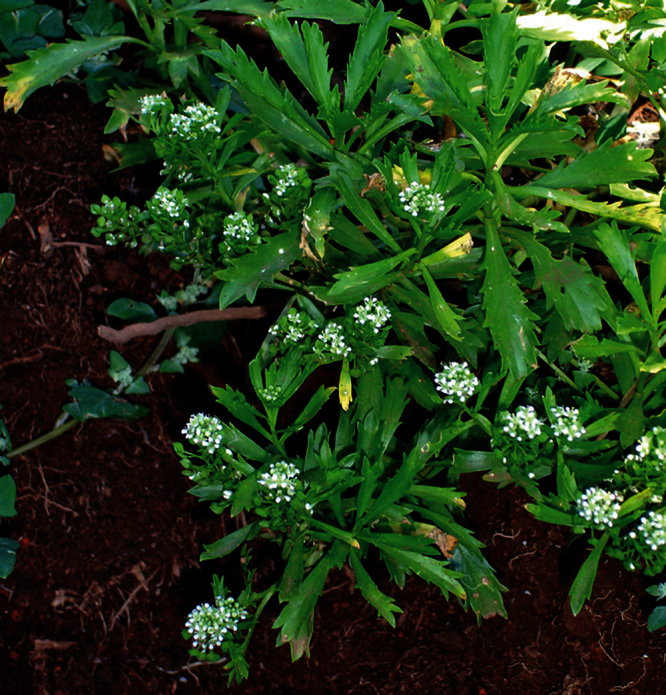
Vegetative stems and inflorescences of *Lepidium panniforme*.

**Figure 84.**
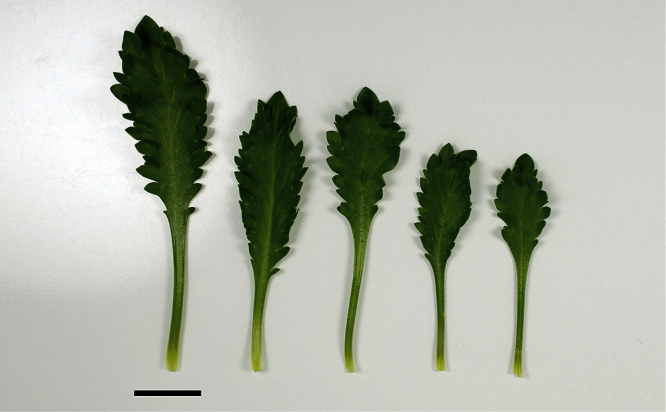
(From left to right) basal- and mid-stem leaves of *Lepidium panniforme*. Scale bar = 20 mm.

**Figure 85.**
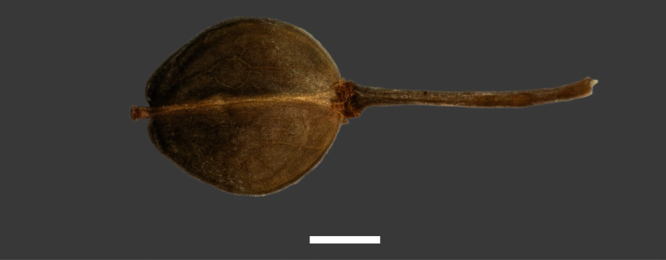
Mature silicle of *Lepidium panniforme* from holotype (AK 255607). Scale bar = 1 mm.

**(Figs 82–85).** Tap-rooted, pungent-smelling, summer-green, perennial herbarising from stock rootstock 7.3–8(–10) mm diam. Growth habit, erect to suberect, loose to densely branched plants up to 1 m across. Stems ± persistent, sometimes dying down to rootstock over winter or in times of adversity; upright to spreading, bases often very stout, ± spherical, somewhat woody when mature 10–15 × 10–12 mm, comprised of numerous old leaf and stem bases, sometimes producing roots, new seasons grow erect to suberect, glabrous, sometimes with very sparse, appressed, caducous, silky 0.5–1 mm long, hairs near stem apices; at fruiting stems often devoid of foliage for much of length. Leaves glabrous, firmly fleshy to succulent, usually dark green to green, sometimes yellow-green. Rosette and stem leaves usually withering at fruiting but sometimes with a few long persistent. Petiole distinct, 20–35(–40) × 2–9 mm. Lamina oblanceolate, lanceolate, narrowly lanceolate (rarely spathulate), 50–100 × 10–30 mm; either deeply toothed and/or lacerate or with distal ⅔ to ⅓ deeply toothed and/or deeply lacerate, teeth in 10–18(–20) pairs, blunt to sharp, running to and including apex and usually extending beyond leaf outline, base cuneate to narrowly cuneate. Middle stems leaves usually with indistinct petioles, these 10–30 mm long; lamina narrowly linear-lanceolate to linear, often recurved to falcate from or near ½ to ⅓ of leaf length, 50–80(–120) × 3–6 mm; margins deeply lacerate and/or toothed, teeth usually prominent, often confined to the upper ⅔, in 12–18(–30) pairs, these running to and including the apex; lamina base tapered, very narrowly cuneate. Upper stem leaves with or without a distinct petiole, petiole if present 40–60 mm, linear to linear-spathulate, occasionally narrowly lanceolate, usually toothed and/or lacerate, patent or recurved and/or falcate for upper ½ of leaf length, 30–50(–100) × 2.0–3.0(–30) mm. Racemes (5–)10–15 mm long, terminal and axillary; rachis glabrous; pedicels glabrous, erecto-patent, 2–5(–8) mm long at fruiting. Flowers c.0.4–0.8(–1.0) mm diam. Sepals glabrous or finely pubescent, or with both states within the one flower, green, broadly ovate to oval, c.0.6–1.0 × 0.6–1.2 mm, with pale-green to white thickened margin, apex broadly obtuse. Petals white, 1.5–2.0 × 0.3–0.8(–1) mm, erecto-patent to somewhat spreading, clawed; limb narrowly obovate, apex obtuse, occasionally emarginated. Stamens (2–)4, equal. Nectaries 2, subulate, 0.35 mm long. Silicles cartilaginous when fresh, coriaceous when dry, orbicular, orbicular-rhomboid (2.5–)3.0–3.5 × (1.5–)1.8–2.5(–3.3), slightly winged in upper ⅓, apex minutely notched, based obtuse to ± cordate, valves glabrous, dried surface often distinctly reticulate; style 0.1–0.2(–0.3) mm long, free from the narrow wing, exceeding the shallow notch; stigma 0.2–0.3 mm diam., capitate. Seeds 2, narrowly ovoid, brown, red-brown to orange-brown, not winged, 1.25–1.3 × 0.35–0.60 mm. FL. Nov–Feb. FR. Nov–Feb.

#### Representative Specimens.

**Chatham Islands:** Mangere Island, Mangere Island Nature Reserve, August 2005, D. Prendeville s.n., (AK 293305); Mangere Island, Mangere Island Nature Reserve, February 2006, B. Gibb s.n., (AK 295945); Mangere Island, Mangere Island Nature Reserve, 14February 2006, P. J. de Lange CH877 & P. B. Heenan, (AK 300343); Mangere Island, Mangere Island Nature Reserve, 14 Feb 2006, P. J. de Lange CH874 & P. B. Heenan,(AK 300340); Mangere Island, Mangere Island Nature Reserve, 14 Feb 2006, P. J. de Lange CH875 & P. B. Heenan, (AK 300341). Rekohu Petre Bay, Waitangi Village, near Council Buildings, (Naturalised) 19 September 2007, P. J. de Lange CH976 & P. B. Heenan, (AK 300992). **Cultivated (New Zealand):** Auckland, Manurewa, ex Mangere Island, Auckland Botanic Gardens, July 2002, P. J. de Lange 5513, (AK 258042); Lincoln, ex Mangere Island, Landcare Research experimental nursery, 10 July 2008, P. B. Heenan s.n., (CHR 609807); Lincoln, ex Mangere Island, Landcare Research experimental nursery, 25 January 2010, *P. B. Heenan s.n*., (CHR 609812).

#### Distribution

**(Fig. 63).** Endemic. New Zealand, Chatham Islands, where it is only known from Mangere and Little Mangere Island. In 2007, *Lepidium panniforme* was collected once growing in the loose gravel of a car park near the Chatham Island Council Buildings, Waitangi, Rekohu. Those plants had probably established from seed that was accidentally discarded there by PdL and PBH while pressing freshly gathered fruiting specimens in 2006.

#### Recognition.

*Lepidium panniforme* has an erect, suberect, or spreading growth habit (Figs 82, 83), which immediately separates it from the decumbent *Lepidium oligodontum*, and *Lepidium rekohuense*. It is further separated from these taxa by the long persistent, much larger, usually deeply toothed or lacerate and often rather tattered basal and lower stem leaves (Figs 81–84), and from *Lepidium oligodontum* and *Lepidium rekohuense* by the flowers which consistently have (2–)4 stamens. In growth habit, the species is most similar to *Lepidium oblitum* and *Lepidium oleraceum*,species with which it grows on Mangere Island. The key distinctions between *Lepidium oblitum* and *Lepidium panniforme* are described under *Lepidium oblitum*, however; in brief, the deeply toothed and/or lacerate leaves of *Lepidium panniforme* serve to readily distinguish it from *Lepidium oblitum*. From *Lepidium oleraceum* it is also easily separated, especially when fruiting when the notched rather than acute silicle apex can be seen, but also when vegetative, as the leaves of *Lepidium panniforme* are diagnostically deeply toothed and/or lacerate. This is a condition seen otherwise only in the Bounty Islands endemic *Lepidium seditiosum* which differs by having hairy inflorescences (Fig. 96) and distinct rDNA ETS sequence. *Lepidium panniforme* shares the same rDNA ETS sequence as the morphologically different *Lepidium obtusatum*. From that species it is recognised by its upright shrub-habit, deeply lobed to lacerate leaves and much smaller, minutely notched silicles.

#### Ecology.

On the highly modified landscape that is Mangere Island, *Lepidium panniforme* is known only from a very few sites where it grows in coastal herbfield along cliff tops, in rough pasture, shrubland, regenerating forest and sites kept artificially open, such as track sides. Because Mangere Island is being actively restored to coastal forest, few of these habitats are natural, and as such it is difficult to determine what the real habitat preferences of *Lepidium panniforme* are. However, in less modified parts of the island, plants are mainly confined to the steeper, often heavily seabird burrowed slopes and cliff margins. Here, plants mostly grow amongst coxella (*Aciphylla dieffenbachii*), *Hebe chathamica*, Chatham Island forget-me-not (*Myosotidium hortensium*), Chatham Island sow thistle (*Embergeria grandifolia* (Kirk) Boulos) and, in places, *Lepidium oblitum*. Little is known about the habitat preferences of *Lepidium panniforme* on Little Mangere Island. The only records of its presence there are a series of photographs taken by the late D.V. Merton in February 2006 which show it present on the forest floor within canopy gaps in the summit forest. One assumes that, as with nearby Mangere Island, it also grows on cliff faces, rock ledges are within other suitably open sites within the coastal shrubland and herbfield of that precipitous island.

#### Conservation Status.

It is notable that while *Lepidium oligodontum* and *Lepidium rekohuense* are present in the earliest *Lepidium* collections made by Travers, Cockayne and Martin from the Chatham Islands, *Lepidium panniforme* is absent. The first records of it are those gatherings and photographs taken by staff of the former Wildlife Service from Mangere and Little Mangere Island during the 1960s and 1970s. Based on these observations, *Lepidium panniforme* is naturally confined to Mangere and Little Mangere Island. On Mangere Island it is known with certainty only from the vicinity of the hut and along the track from there across to the isthmus. In 2006, c.60 individuals were seen in these areas.

Subsequent field work by Department of Conservation staff on Mangere Island indicates that *Lepidium panniforme* is a very uncommon species, which is potentially further threatened by the re-vegetation of that island. This is in part a natural process, though it is one which has been augmented by deliberate plantings as part of that island’s long term restoration management as a wildlife refuge for threatened endemic fauna (D. Houston pers. comm.). Nevertheless replanting within the *Lepidium panniforme* siteshas been carefully managed to date, and the species remains locally abundant along the disturbed and open track margins from the hut to the isthmus. However, with such a small population *Lepidium panniforme* remains vulnerable to any change in management, and, over time as the surrounding shrubland matures to forest, some decline through natural succession is to be expected. Therefore, this remarkable species will need to be closely monitored to ensure that it is not lost from the island. There is also a need to survey for the species on the almost inaccessible, closely adjacent Little Mangere Island, as the species is still known from there only from those few images taken by the late D.V. Merton.

Therefore, with < 250 mature plants known, and a total area of occupancy of < 1 ha, using the New Zealand Threat Classification System (see Townsend et al. 2008), *Lepidium panniforme* is rated as ‘Threatened/Nationally Critical’. In this case criterion A(1) or A(3) equally apply. To that listing we recommend appending the qualifiers, ‘CD’ (Conservation Dependent – as the species is being actively managed), ‘DP’ (Data Poor – because accurate information about its status on Little Mangere Island is still needed), ‘IE’ (Island Endemic), ‘OL’ – because the species occurs on two closely associated islands such that it is at risk of elimination through catastrophe), and “RR” (Range Restricted – because of the narrow geographic range this plant occupies in relation to the rest of the New Zealand Botanical Region).

### Lepidium rekohuense sp. nov.

urn:lsid:ipni.org:names:77129262-1

http://species-id.net/wiki/Lepidium_rekohuense

*A L. oleraceo habitu renascenti, habenti periodo distincto rosulato, ramis sparse papillato-pilosis, floribus 2-staminatis, pedicellis minute pilosis, siliculis orbiculatis, minute alatis et emarginatis et serie DNA differt. A speciebus Lepidii ceteris Insularum Chathamicarum (L. panniformo et L. oligodonto) caulibus minute papillato-pilosis, floribus semper 2-staminatis, foliis serratis et siliculis orbiculatis (raro obovatis) differt. A L. panniformo praeterea habitu decumbenti et serie DNA recedit*.

#### Holotype.

**Figure 86.**
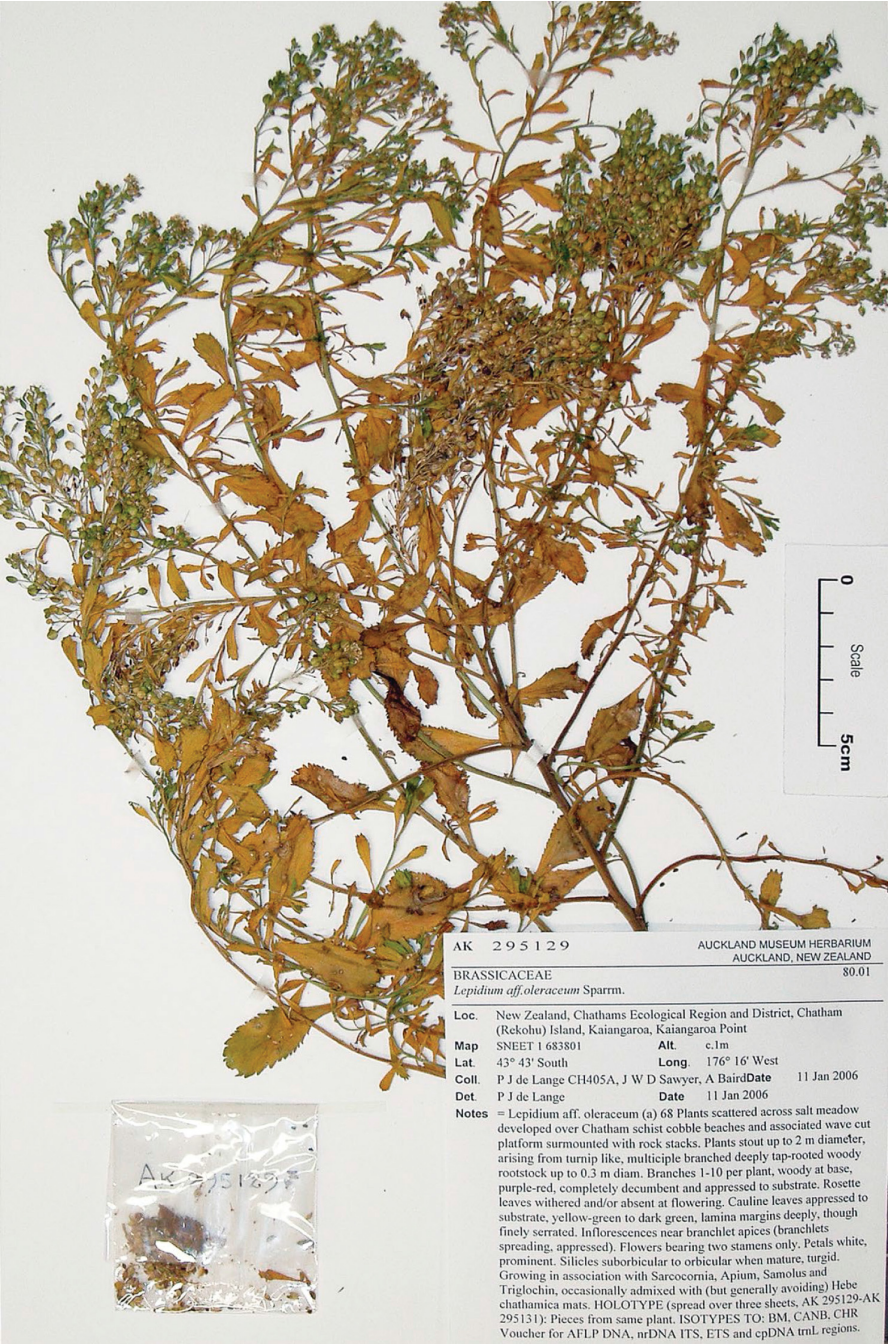
Holotype of *Lepidium rekohuense* de Lange et Heenan (sheet **A**).

**Figure 87.**
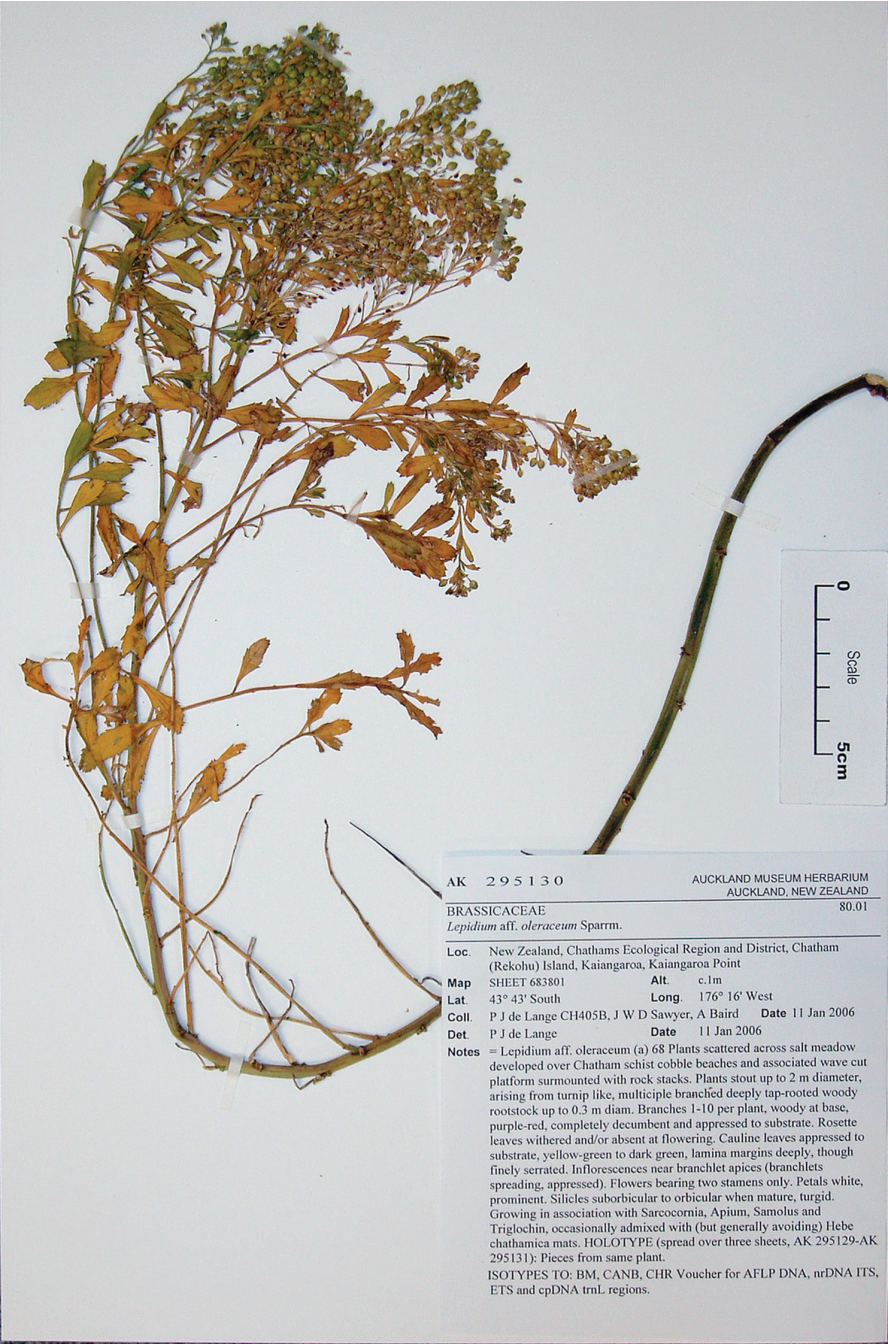
Holotype of *Lepidium rekohuense* de Lange et Heenan (sheet **B**).

**Figure 88.**
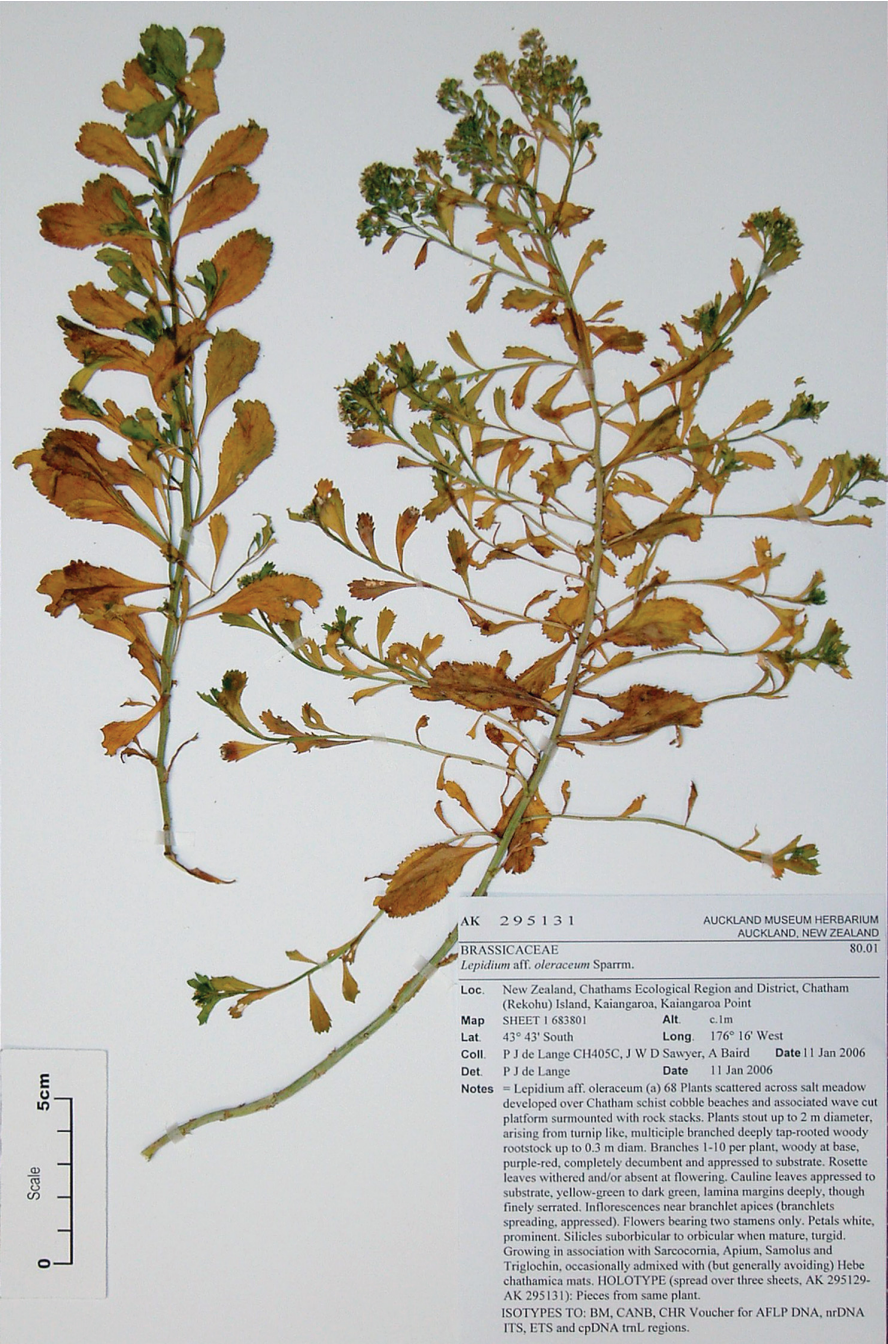
Holotype of *Lepidium rekohuense* de Lange et Heenan (sheet **C**).

**Chatham Islands (Figs 86–88):** Chatham (Rekohu) Island, Kaiangaroa, Kaiangaroa Point, 11 January 2006, P. J. de Lange CH405, J. W. D. Sawyer & A. Baird, Collection over three sheets comprising four pieces (one sterile) taken from the same plant. (AK 295129-AK 295131! Isotypes: BM!, CANB!, CHR!

**Etymology.** The epithet ‘*rekohuense*’ is derived from ‘Rekohu’, the Moriori name for Chatham Island which is said to mean ‘land of misty skies’ (King 1989). This name was chosen to reflect the endemic status of this species on the Chatham Islands group.

**Figure 89.**
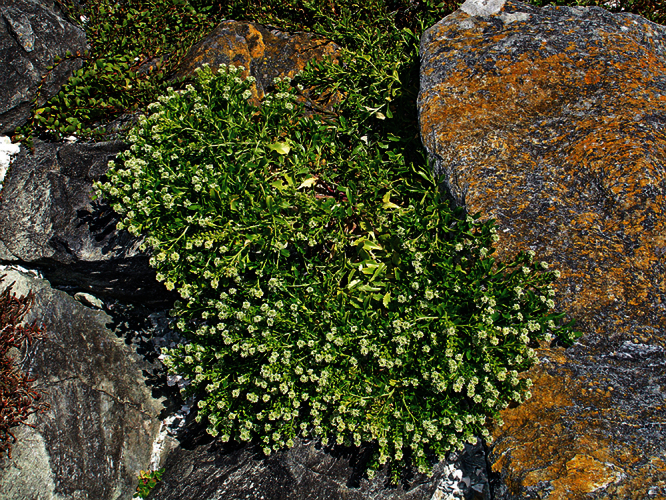
Wild plant of *Lepidium rekohuense* showing decumbent branches and inflorescences, plant growing amongst Chatham Schist boulders at Kaiangaroa, Rekohu (boulders are 1–2 m diameter)

**Figure 90.**
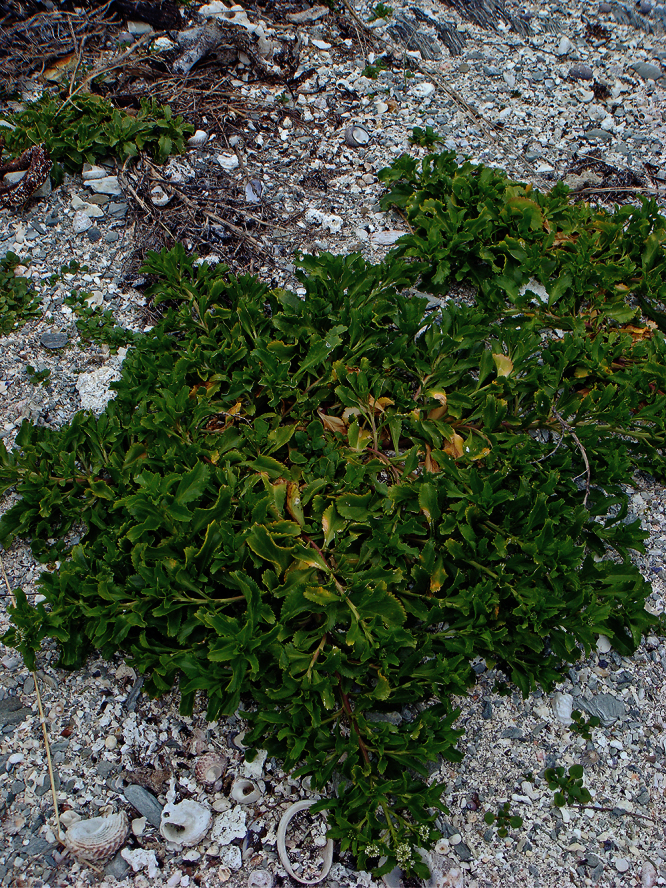
Decumbent vegetative stems of *Lepidium rekohuense*.

**Figure 91.**
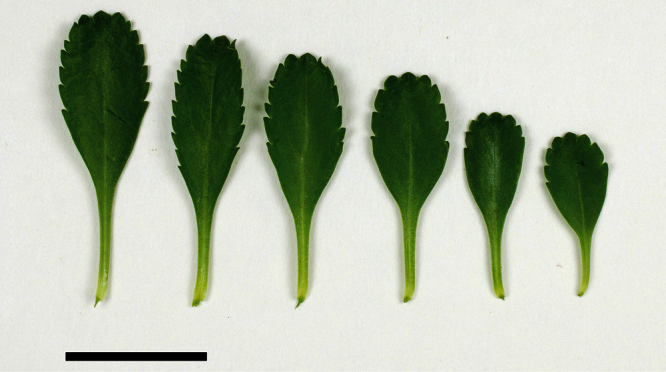
(From leaf to right) rosette-, basal- and mid-stem leaves of *Lepidium rekohuense*. Scale bar = 20 mm.

**Figure 92.**
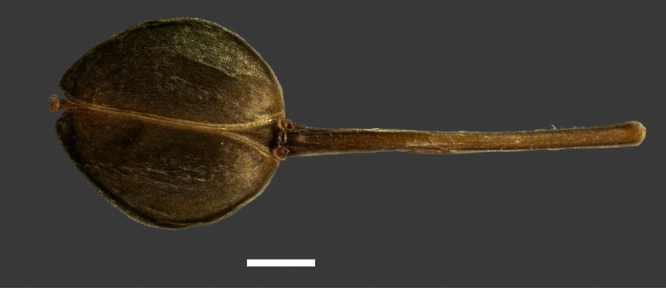
Mature silicle of *Lepidium rekohuense* from holotype (AK 297694). Scale bar = 1 mm.

**Description (Figs 89–92):** Tap-rooted, pungent-smelling, decumbent, summer-green, perennial herb forming densely leafy masses up to 2 m diam., and arising from stout, semi-circular, dark reddish-grey (when exposed) rootstock 100–500 mm diam. Tap root woody, up to 1.5 m long, deeply descending. Plants dying down to rootstock and/or previous seasons stem nodes, over winter or in times of adversity. Stems decumbent, widely spreading, up to 2 m long and 30 mm diam., woody, ± spherical in cross-section, prominently ridged and/or grooved (especially when dry), dark reddish-green to dark green, usually scarred throughout with numerous old leaf bases; stems heavily branched in upper ⅔, branches and branchlets numerous, prostrate, widely spreading, very leafy; basal portion of stems, ± glabrous, otherwise finely and sparsely papillate-hairy, especially along leaf decurrencies, and within stem grooves, hairs very short 0.01–0.3 mm long, white, glandular-pustulate, rather sticky when fresh. Leaves glabrous, firmly fleshy to succulent, dark green to green, at senescence turning yellow. Rosette leaves persistent at fruiting; petioles distinct up to 50 × 3 mm, slightly concave in cross-section, fleshy; lamina narrowly spathulate to spathulate-oblong, up to 30.0 × 13.3 mm, margins usually denticulate, crenulate, if denticulate then with 10–18(–26) pairs of blunt teeth running to and including apex, base broadly attentuate. Middle stems leaves persistent at fruiting; petiole distinct up to 15 × 2 mm, mostly flat in cross-section, sometimes slightly concave, fleshy; lamina elliptic, narrowly elliptic to oblong, 18.86–26.18(–35.00) × 9.64–16.20 (–18.00) mm; margins sharply and regularly serrate-dentate with 10–16(–22) pairs of teeth running to and including the apex, lamina base broadly cuneate to cuneate. Upper stem leaves with or without a distinct petiole, petiole if present 2.14–5.60 mm, flat; lamina 9.46–10.58 (–17.00) × 2.03–3.48(–6.14) mm, narrowly oblanceolate, oblanceolate to obdeltoid, apex often tridentate, base cuneate to narrowly cuneate; lamina margins deeply dentate, incised, or otherwise entire except for the upper ⅓ which is prominently toothed; teeth if present in 2–6 pairs running to and including the apex. Racemes (10–)26(–60) mm long, elongating up to 90 mm at fruiting, terminal and axillary; rachis and pedicels finely and sparsely covered in retrorse to patent, very short, 0.05–0.8 mm long, ± clavate, eglandular–glandular, hairs; pedicels, erecto-patent to patent,1.04–1.27(–2.38) mm, 2.34–5.00(–6.02) mm long at fruiting. Flower buds dark green, apex bearing a conspicuous, caducous, crest of white, eglandular, antrorse hairs up to 0.9 mm long. Flowers sweetly fragrant, 1.4–1.8(–2.0) mm diam. Sepals, broadly ovate to oval, c.0.6–1.0 × 0.6–1.2 mm, apex broadly obtuse, centrally green with a white margin, deeply concave, adaxially weakly keeled, adaxial midrib invested in conspicuous, caducous, white, eglandular, antrorse, hispid hairs, hairs sometimes scattered across rest of adaxial surface; abaxial surface glabrous. Petals white, 0.3–0.8(–1.0) × 0.2–0.8 mm, erecto-patent or patent, clawed; limb broadly obovate, apex obtuse, retuse or distinctly emarginate. Stamens 2, equal. Anthers c.0.16 mm long. Pollen bright yellow. Nectaries 4, subulate, 0.40 mm long. Silicles cartilaginous when fresh, coriaceous when dry, orbicular to obovate, (2.8)–3.3(–4.1) × (2.2–)3.3–3.4 (–4.0), narrowly winged, apex shallowly, minutely, notched, base cordate, valves dark green to green maturing straw-yellow, glabrous; style 0.8 (–1.0) mm long, free from the narrow wing, equal to or slightly exceeding the notch; stigma 0.2–0.4 mm diam. Seeds 2, 1.20–1.38 × 0.80–1.10 mm, ovoid to suborbicular, red-brown, dark red-brown or brownish black, not winged. FL. Nov–Feb. FR. Jan–Apr.

#### Representative Specimens.

**Chatham Islands:** n.l., n.d., [H. H.] Travers 105, (MEL 301452); Rekohu, Kaingaroa Point, 3 March 1985, D. R. Given 14017, (AK 225198, CHR 417647); Rekohu, Kaingaroa Point, 21 February 1996, P. J. de Lange CH80 & G. M. Crowcroft, AK 230459; Rekohu, Kaingaroa Point, 15 July 2002, P. J. de Lange CH332 & A. Baird, (AK 259130); Rekohu, Kaiangaroa, Kaiangaroa Point, 13 December 2005, A. Baird s.n., (AK 295132); Rekohu, Wharekauri Farm Station, Cape Young, 13 January 2006, P. J. de Lange CH424 & J. W. D. Sawyer, (AK 295153); Rekohu, Waitangi Village, near Council Buildings (naturalised), 19 September 2007, P. J. de Lange CH975 & P. B. Heenan, (AK 300991); Rabbit Island, 14 February 2006, P. J. de Lange CH676 & P. B. Heenan, (AK 296754). Forty Fours (Motuhara) 27 January 2005, R. M. Bellingham s.n., (AK 290290). **Cultivated (New Zealand):** Lincoln, ex Kaiangaroa, Landcare Research experimental nursery, December 2008, P. B. Heenan s.n., (CHR 609795).

#### Distribution

**(Fig. 63).** Endemic. Chatham Islands where it is known only from Rekohu and Rabbit Island, and the Forty Fours (Motuhara). *Lepidium rekohuense* has also been collected once as a casual in a car park in the main settlement of Waitangi. This occurrence of a single plant along with that of *Lepidium panniforme* collected in the same site probably accords with the use of nearby accommodation by PdL and PBH in 2006, during which time fruiting herbarium specimens of both species were processed in that general area.

#### Recognition.

Healthy specimens of *Lepidium rekohuense* can form patches up to 2 m in diameter, which is the largest of the New Zealand endemic *Lepidium* species (Fig. 89). Within the *Lepidium oleraceum* group, *Lepidium rekohuense* is morphologically most similar to *Lepidium oblitum* and *Lepidium oligodontum*. From these species by is easily separated by the flowers which consistently have two rather than 2–4 (*Lepidium oblitum*) or 2–4–6 (*Lepidium oligodontum*) stamens, by its much larger overall stature (up to 2 m diam.), by the sparsely papillate-hairy upper branch stems, and by the presence of retrorse to patent, very short, ± clavate, eglandular–glandular hairs on the inflorescence rachis and pedicels. The silicles of *Lepidium rekohuense* are orbicular (rarely obovate) and consistently, though minutely, notched (Fig. 92), while those of *Lepidium oligodontum*, orbicular to suborbicular and not or scarcely notched.

#### Ecology.

*Lepidium rekohuense* is currently known from salt-marsh and meadow at Kaiangaroa, from steep, eroded basaltic tuff erosion gullies and cliff faces at Cape Young and on Rabbit Island, and from the crevices and ledges of greywacke rock outcrops of the Forty Fours (Motuhara). At Kaiangaroa, *Lepidium rekohuense* is a seasonally conspicuous member of the salt marsh and meadow vegetation that has developed behind the cobble beach and shallow shelving schist shore platform in and around Kaiangaroa Point. Here, plants grow in a variety of situations ranging from fully exposed and eroded habitats to low windswept thickets dominated by *Hebe chathamica*, and *Hebe chathamica* × *Hebe dieffenbachii* hybrids. In the salt marsh plants are usually found growing within dense *Sarcocornia quinqueflora* (Bunge ex Unq.-Sternb.) A.J.Scott. subsp. *quinqueflora*, *Samolus repens* var. *repens*, and *Selliera radicans* Cav. turf. In this habitat, plants are often lost during storm surges or during the winter months, and it would seem, from the presence of seedlings and young plants along drainage channels and in and around eroded sections of salt marsh, that these storm events are necessary to exhume and disperse buried seed. At the back of the salt marsh, where the salt meadows are dominated by taller plants such as *Apodasmia* aff. *similis*, *Ficinia nodosa* (Rottb.) Goetgh. Muasya et D.A.Simpson, and occasional *Myosotidium hortensium*, *Lepidium rekohuense* plants are also present, and here they often grow intermixed with *Apium prostratum* subsp. *denticulatum*, *Selliera*, *Samolus*, and *Leptinella potentillina*. Higher up, where thickets of *Hebe chathamica* and hybrids form the dominant cover, *Lepidium rekohuense* is less common, in part because they are often easily missed as they grow with *Apium prostratum* subsp. *denticulatum* threaded through *Hebe chathamica*. *Lepidium rekohuense* is also occasionally found growing on and around the small schist rock stacks around Kaiangaroa Point.

At Kaiangaroa the highly exposed and dynamic habitat means that many *Lepidium rekohuense* plants, especially the younger plants are often lost through coastal erosion and from storm surges. However, in favourable sites, plants are remarkably resilient and long-lived once established. For example, mature plants first observed in 1996 are still present at the time of writing (2012) 16 years later, making this species easily the longest lived member of the *Lepidium oleraceum* complex in New Zealand. The key to this species success at Kaiangaroa seems to be its remarkable tap root, which, once established, firmly anchors the plant into the substrate such that coastal erosion often leaves mature plants exposed, festooned in driftwood and kelp, while the surrounding salt-marsh turf has been destroyed.

The habitat occupied at Cape Young and on Rabbit Island is markedly different. Here the species grows at the apex of steeply descending, erosion gullies that have developed within the easily eroded basaltic tuff. In these sites it is often the only plant present though, in a few places on Cape Young, it grows with *Lepidium flexicaule*, with which it occasionally hybridises. On Rabbit Island, large plants grew at the head of an erosion gully under a sparse canopy of the introduced tree mallow (*Malva arborea*).

Little is known about its habitats on the remote Forty Fours (Motuhara). From the limited information available (P. N. Johnson pers. comm.) it seems that the species is very uncommon there, and that it grows mainly within crevices and on ledges on the cliff faces of those rock stacks.

#### Conservation status.

The most recent census data that we have (July 2007) recorded 114 adult plants of *Lepidium rekohuense* from just three accessible sites; two on Rekohu (Kaiangaroa and Cape Young) and one on Rabbit Island. The status is uncertain of the species on the Forty Fours, privately owned land from which the Department of Conservation has not been granted visiting rights. Nevertheless, observations made in 2005 by a private landing party of geologists, entomologists and ornithologists suggest that there are probably fewer than 10 plants on the larger of the two main islets making up the Forty Fours. Using the New Zealand Threat Classification System (Townsend et al. 2008), *Lepidium rekohuense* is rated “Threatened/Nationally Critical” using criterion A(1) because there are < 250 adult plants known from the wild. To this threat rating we recommend appending the qualifiers ‘CD’ (Conservation Dependent – due to need for ongoing management of the Kaiangaroa population), ‘IE’ (Island Endemic – because *Lepidium rekohuense* is naturally confined to the Chatham archipelago). It is worth noting that, without management, the largest population of *Lepidium rekohuense* known to the Department of Conservation, that at Kaiangaroa, would probably now be extinct. There, intensive management has built the population up from an apparent low of six plants in 1996 to more than 100 in January 2006. At Cape Young the sole accessible plant seen in January 2006 had disappeared by January 2007 but others observed further down the cliffs in sites inaccessible to human traffic are apparently still present (A. Baird pers. comm.). On Rabbit, eight plants were observed in February 2006 and these are assumed to be still present. Observations at Kaiangaroa suggest that, aside from the losses caused by the naturally dynamic conditions of that exposed coastal shore platform and wetland, predation from the caterpillars of the moth *Epyaxa rosearia* Doubleday, 1843 (identified by J. Dugdale *pers comm*.) is the main threat facing that population. Currently the caterpillars’ of this moth are managed by regular applications of derris dust, without which most (sometimes all) adult *Lepidium rekohuense* plants can be severely damaged, affecting especially flowering and seed set, and killing seedlings and young plants. At Cape Young, *Lepidium rekohuense* may be threatened by hybridisation with *Lepidium flexicaule* though this requires further study. So far only one putative and not completely convincing example of this hybrid has been observed (see *Lepidium flexicaule* above).

### Lepidium seditiosum sp. nov.

urn:lsid:ipni.org:names:77129263-1

http://species-id.net/wiki/Lepidium_seditiosum

*A L. oleraceo rachidibus inflorescentiae et pedicellis hirsutus et a serie rDNA ETS differt. A L. panniformo floribus 4-staminatis praeterea differt*.

#### Holotype.

**Figure 93.**
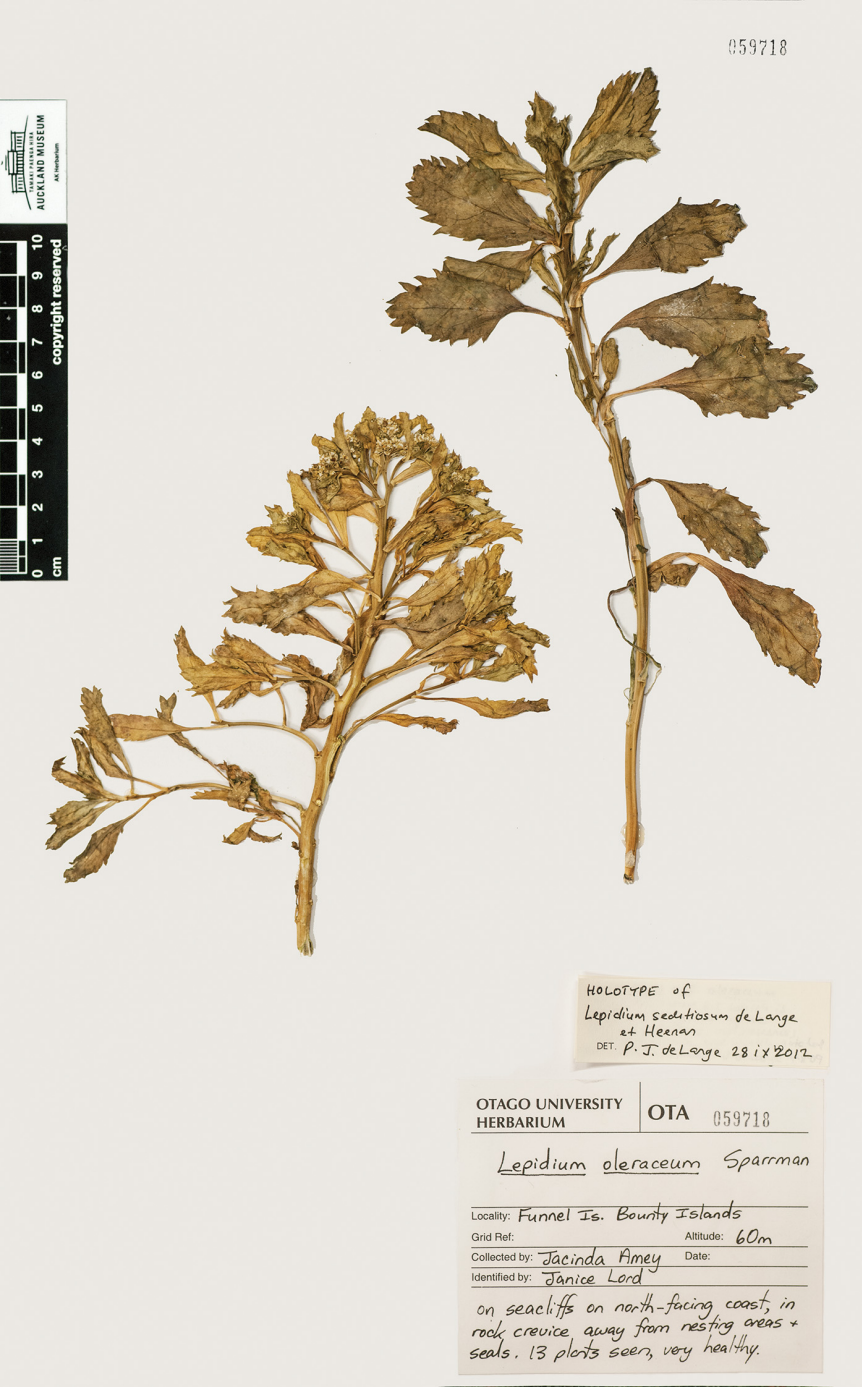
Holotype of *Lepidium seditiosum* de Lange, Heenan et J.Rolfe.

**Bounty Islands (Fig. 93):** Bounty Islands, Funnel Island, n.d.J. Amey s.n, OTA 59718!

#### Notes:

*Lepidium seditiosum* is known only from the holotype collection, which was collected sometime in 2004 (Amey et al. 2007).

#### Etymology.

The epithet ‘*seditiosum*’ derived from the Latin ‘*seditio*’ (meaning: dissension, insurrection, mutiny, rebellion), alludes to the mutiny of the majority of Lieutenant William Bligh’s crew on the 28 April 1789. In 1788, some months before the rebellion, Bligh had discovered and named the Bounty Islands taking the name from his ill-fated ship The Bounty.

#### Description

**Figure 94.**
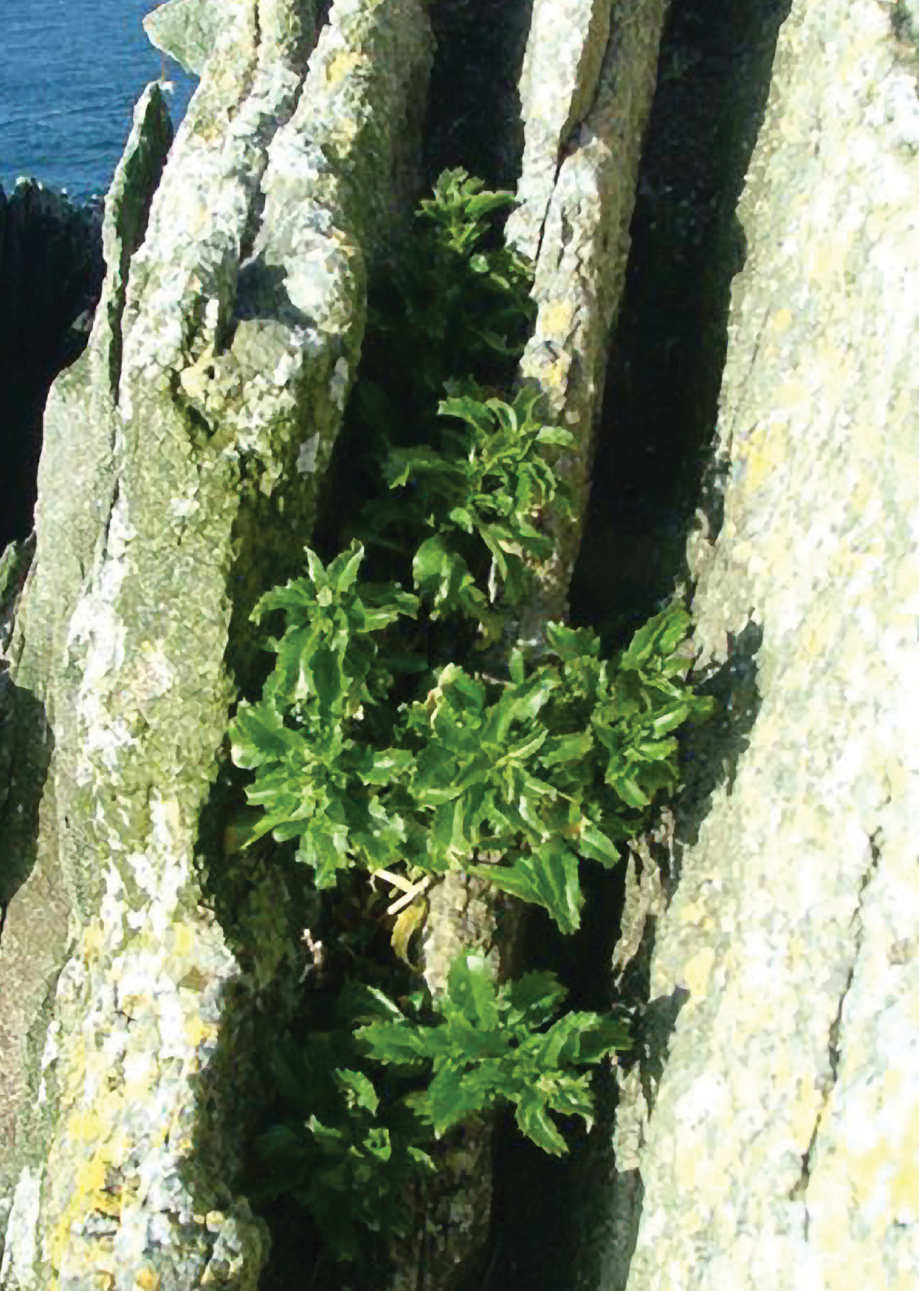
*Lepidium seditiosum* growing in granite crevice on summit of Tunnel Island, Bounty Islands (image: J. Amey).

**Figure 95.**
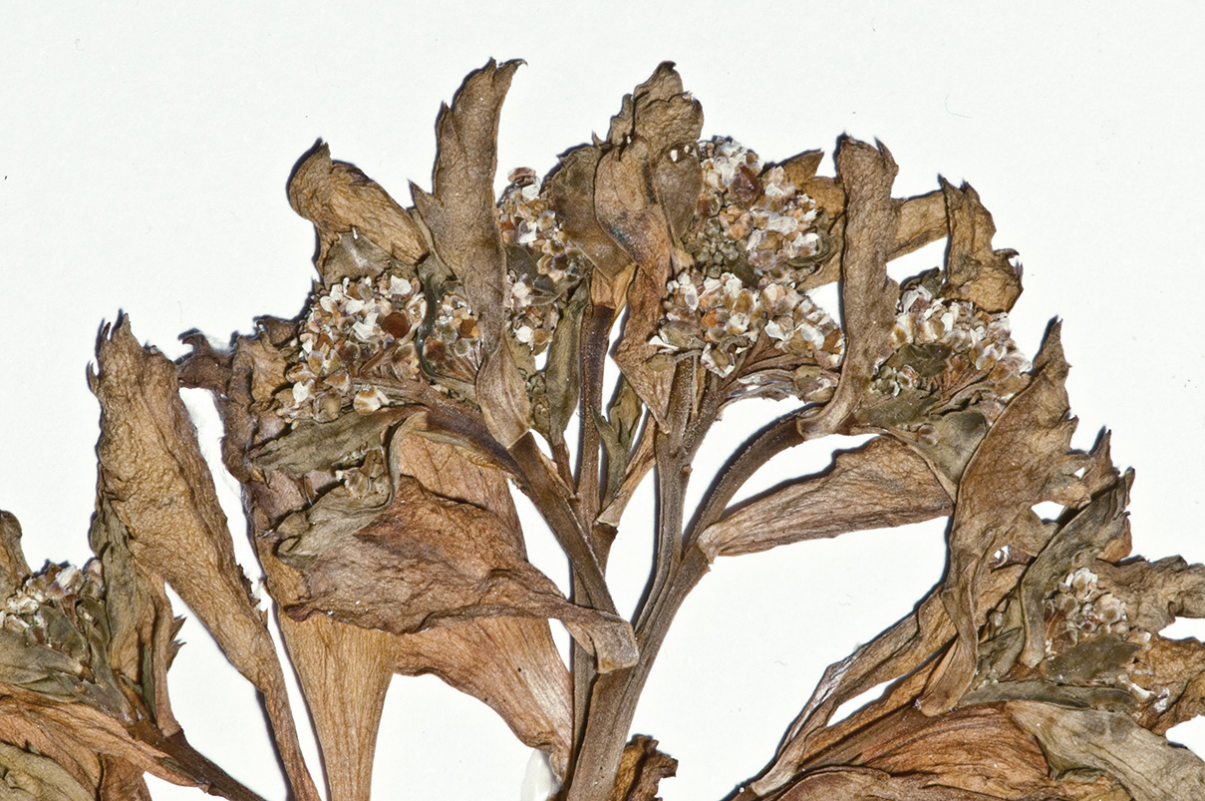
Inflorescence of holotype of *Lepidium seditiosum* (OTA 59718).

**Figure 96.**
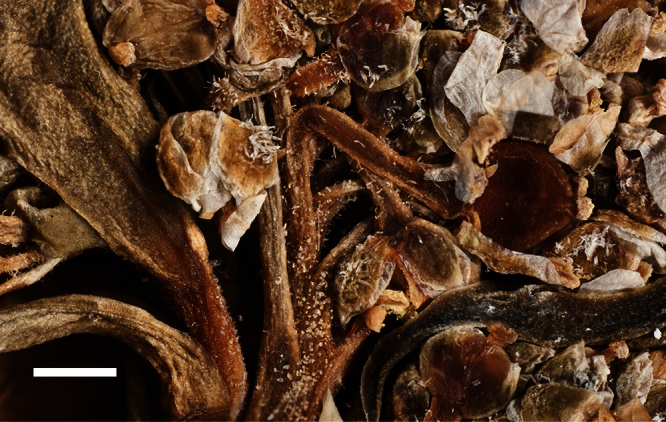
Close up of inflorescence of holotype of *Lepidium seditiosum* (OTA 59718) showing hairy rachises and pedicels, and immature silicles. Scale bar = 1 mm.

**(Figs 94–96).** Erect, perennial herb. Stems sparse, erect; mature stems long, 4.14–4.41 mm diam. stout, woody, rigid, ± square, prominently angled, bases much covered in leaf abscission scars, middle and upper portion leafy. Leaves fleshy, dark green, stem leaves evidently withering with age; petiole distinct, 14–22.0 × 1.9–2.6 mm, decurrent, prominently channelled, broadly winged, with a broadly sheathing base; lamina variable 32.4–45.4 × 21.2–26.4 mm decreasing in size toward inflorescences, broadly elliptic, elliptic to oblanceolate; apex praemorse or tridentate; margin coarsely and ± regularly dentate to deeply incised; teeth protruding beyond leaf outline; in 8–12 uneven pairs, up to 5.3 mm deep, increasing in size toward apex; base broadly attenuate tapering, extending into a broad petiole wing. Inflorescences immature but evidently racemose, rachis 1.3–1.5 mm diam., terminal and lateral, leaf-opposed, densely and mostly circumferentially covered in 0.4–0.6 mm long, white, clavate hairs; pedicels 1.6–2.2 mm long at flowering, erecto-patent, densely and mostly circumferentially covered in 0.4–0.6 mm long, white, clavate hairs. Flowers 2.3–2.6 mm diam. Sepals 4, saccate, dark green usually with a narrow white, ± undulose margin; lateral sepals broad, 0.8–1.2 mm diam., obovate to broadly obovate, ± overlapping at base, apex rounded to obtuse, abaxial surface densely hairy, hairs 0.1–0.4 mm long, eglandular or glandular, mostly clavate, some setose, median sepals 0.8–1.0 mm diam., broadly obovate, dark green, usually with a narrow white, ± undulose margin, apex rounded to obtuse, abaxial surface densely hairy, hairs 0.1–0.4 mm long, eglandular or glandular, mostly clavate, some setose. Petals white, 1.3–2.0 × 1.0–2.3 mm, mostly recurved over stigma some spreading, claw 0.4–0.9 mm long; limb obovate, obovate-spathulate rarely orbicular, apex obtuse or slightly emarginate, margins smooth. Stamens 4, filaments 1.2–1.8 mm long, white; anthers 0.3–0.4 mm long, yellow. Ovary 1.1–1.8 × 0.6–1.3 mm, broadly ovate to elliptic, dark green, apex round or weakly notched; style 0.11–0.4 mm long, cylindrical below, broadly spreading at apex; stigma 0.2–0.4 mm diam. Nectaries 4, 0.2–0.3 × 0.1–0.15 mm, narrow-oblong, pale translucent green. Mature silicles not seen. FL Nov. FR unknown.

#### Distribution

**(Fig. 76).** Endemic. Bounty Islands (Funnel Island, Molly Cap).

#### Recognition.

Amey et al. (2007) initially reported the presence of *Lepidium oleraceum* s.l. from the remote Bounty Islands. In that paper they noted that unpublished rDNA ETS data from their gathering placed the Bounty Islands plant within a southern *Lepidium oleraceum* clade of samples collected from Banks Peninsula south to Stewart Island rather than plants from the nearby Antipodes Islands. On the basis of that information they suggested that the Bounty Islands plant was a recent arrival, probably coming from the Otago coastline of the southern South Island. Subsequently, the rDNA ETS phylogeny presented here confirms that *Lepidium seditiosum* is related to the South Island species *Lepidium aegrum*, *Lepidium crassum*, and *Lepidium juvencum* (Fig. 2). However none of these species are morphologically similar to *Lepidium seditiosum*. Morphologically, the most similar species is the Chatham Islands endemic *Lepidium panniforme*, which also has deeply toothed (though also lacerate leaves) but whose stems and inflorescence rachis are glabrous (only very rarely furnished with sparse silky hairs), and whose rDNA ETS sequence places it as sister to *Lepidium obtusatum*. The Bounty Islands plant differs from *Lepidium panniforme* (and indeed all other members of the *Lepidium oleraceum* complex) by its distinctly clavate-hairy upper stems and inflorescence rachises.

While our material is inadequate to furnish a full species description, the absence of mature silicles and basal leaves should not preclude formal taxonomic recognition. The Bounty Islands are perhaps the most remote outlier of the New Zealand Archipelago, and are very rarely visited (Amey et al. 2007), so the alternative, of waiting for better fruiting material before describing what in our view is clearly a new species is, unacceptable. In the authors’ opinion, there is sufficient data available to justify this plant’s taxonomic recognition at species rank.

#### Ecology.

Prior to the remarkable discovery of *Lepidium* on the Bounty Islands group, these islands had always been considered devoid of any form of terrestrial vegetation beyond a few lichens and algae (Amey et al. 2007). None of the 15 islands, islets and rock stacks comprising the group reach any higher than 88 m a.s.l., and virtually all available dry land is occupied by some of the densest concentrations of nesting seabirds seen anywhere in the world (Amey et al. 2007). Amey et al. (2007) recorded *Lepidium seditiosum* (as *Lepidium oleraceum* s.l.) as growing within rock crevices near the summits of one island (Funnel Island) and an inaccessible (by boat) rock stack (Molly Cap) within the Bounty Islands group. At the time of their discovery, on Funnel Island plants grew on the margins of a colony of nesting seabirds, in a site inaccessible to albatross and penguins but used by Fulmar prions *(Pachyptila crassirostris)* and possibly Cape pigeons *(Daption capense)*. They noted that where the plants grew, a ‘rich friable soil’ had accumulated, and that the crevice walls provided some shelter from the most extreme winds. Because of the inaccessible nature of Molly Cap, no further observations on the plant they had seen from their boat could be made.

#### Conservation Status.

Amey et al. (2007), during a November 2004 visit to the Bounty Islands group, recorded ‘at least 13’ plants at two sites (Funnel Island (12 plants) and Molly Cap (1 plant). On this basis, *Lepidium seditiosum* qualifies as ‘Threatened / Nationally Critical’ (either criterion A(1) or A(3) of Townsend et al. (2008)) as there are fewer than 250 mature plants known and the total area of occupancy is < 1 ha. To this assessment we add the qualifiers ‘CD’ (Conservation Dependent – as the Bounty Islands are a Nature Reserve and World Heritage Site with strict controls in place to regulate landings, and prevent the spread of diseases, weeds and foreign predators), ‘DP’ (Data Poor – because accurate data on the total number of individuals and trend data is not available), ‘IE” (Island Endemic), and ‘OL’ (One Location – because, following the definition in Townsend et al. (2008), the species is confined to the Bounty Islands group, where it grows on an island and rock stack in close proximity, and so the chances of losing the species to a single event are greater than for a species found in one island group on several widely separated islands).

## Revised key to *Lepidium* in New Zealand

* Denotes naturalised in New Zealand.

**Table:** 

1	Plants dioecious	2
–	Plants hermaphrodite	3
2	Cauline leaves with pinnae in 6–25 primary pairs; primary pinnae linear to lanceolate, recurved; terminal pinnae 7.8–30.0 × 0.9–2.9 mm, lateral pinnae 8.0–28.9 × 0.8–2.7 mm; petals present or absent, those of ♂ flowers 1.5–2.2 mm long, those of ♀ flowers 1.2–1.5 mm long	*Lepidium sisymbrioides* Hook.f.
–	Cauline leaves with pinnae in 14–32 primary pairs; primary pinnae linear, obovate to broadly oblong, not recurved; terminal pinnae 3.0–16.0 × 1.0–4.9 mm, lateral pinnae 2.6–11.3 × 0.8–3.9 mm; petals usually absent, if present those of ♂ flowers 1.3–1.5 mm long, those of ♀ flowers 0.8–1.1 mm long	*Lepidium solandri* Kirk
3	Silicle reticulate or warty; valves indehiscent or breaking into two 1-seeded segments (not splitting along valves)	4
–	Silicle smooth or papillate, glabrous or hairy; dehiscing by 2 valves to leave a persistent replum	6
4	Plants rhizomatous; stems erect up to 0.6 m tall; stems and leaves covered in short, arching hairs; leaves simple, glaucescent; pedicel > 3× silicle length; style > 1.0 mm long	*Lepidium draba L.
–	Plants tap-rooted without rhizomes; stems decumbent up to 1m long; stems and leaves glabrous, papillate or sparsely hairy on petioles of basal leaves, hairs if present spreading; leaves 1–2-pinnatifid, dark green, grass green to yellow-green; pedicel < 2× silicle length; style < 0.5 mm long	5
5	Petals 0 or if present < sepals, up to 0.5 mm long; stamens 2–4; pedicel ≥ silicles; silicles 1.4–1.7 mm long, apex notched, dehiscent at maturity, valves reticulate and ridged or finely warty; style ± sessile	*Lepidium didymum L
–	Petals > sepals, 1.0–2.0 mm long; stamens 6; pedicel ≤ silicles; silicles > 2 mm long, apex apiculate, indehiscent, valves coarsely warty, corky; style up to 0.5 mm long	*Lepidium coronopus (L.) Al-Shehbaz
6	Silicle apex acute (not notched)	7
–	Silicle apex notched	8
7	Mid and upper stem leaves acuminate, teeth acicular-acerose; pedicels minutely hairy	*Lepidium castellanum* de Lange et Heenan
–	Mid and upper stem leaves not acuminate, teeth acute or subacute, not acicular-acerose; pedicels glabrous	*Lepidium oleraceum* G.Forst. ex Sparrm.
8	Stamens 6	9
–	Stamens 2 or 4	12
9	Leaves, stems and pedicels glabrous; stamens 2–4–6; leaf base attenuate	10
–	Leaves, stems and pedicels hairy; stamens 6; leaf base amplexicaul	11
10	Cotyledons 3-fid; plant erect; basal and stem leaves pinnatifid; stamens 6; silicles 5.0–9.0 × 3.0–5.0 mm, broadly ovate to elliptic, surface smooth when fresh or dry	*Lepidium sativum L.
–	Cotyledons simple; plant decumbent; leaves simple, toothed; stamens 2–4–6; silicles 3.0–3.8 × 2.8–3.8 mm, broadly orbicular and turgid when fresh, valve surface collapsing on drying to become wrinkled or reticulate	*Lepidium oligodontum* de Lange et Heenan^1^
11	Plants annual; silicle densely papillate; style 0.2–0.5(–0.7) mm long, not or only slightly protruding beyond apical notch of silicles; anthers yellow	*Lepidium campestre (L.) W.T. Aiton
–	Plants perennial; silicle not, or sparsely papillate; style (0.6–)1.0–1.5 mm long, protruding well beyond apical notch of silicles; anthers red or violet	*Lepidium heterophyllum Benth.
12	Stamens 2 or 2–4	13
–	Stamens 4	29
13	Plants suberect to erect	14
–	Plants decumbent or prostrate	25
14	Stems papillate, puberulent, or pubescent	15
–	Stems glabrous at least below, rarely with a few hairs above	20
15	Silicles mostly orbicular or obovate (rarely with some broadly elliptic or broadly ovate)	16
–	Silicles ovate, elliptic or rhomboid	18
16	Upper stem leaves pinnatifid, stem hairs spreading	*Lepidium bonariense L.
–	Upper stems leaves entire or toothed; stem hairs strongly recurved	17
17	Silicles obovate, widest above middle; rachis of raceme with straight cylindrical or clavate hairs; petals absent or rudimentary and 0.3–0.9 mm; cotyledons incumbent	*Lepidium densiflorum Schrad.
–	Silicles orbicular, widest at middle; rachis of raceme with curved hairs; petals 1.0–2.5 mm, rarely rudimentary; cotyledons accumbent or rarely incumbent	*Lepidium virginicum L.
18	Young stems and upper leaf margins with small triangular cartilaginous denticles, often paired from a common base; mid-stem leaves oblanceolate to cuneate, margins serrate to dentate; silicles 2.7–3.0 × 2.0–2.3 mm ovate	*Lepidium desvauxii* Thell. 2
–	Young stems and leaf margins puberulent; mid-stem leaves linear, lanceolate to oblanceolate, entire with marginal hairs, silicles 1.8–5.0 × 1.5–2.5 mm, elliptic, ovate to rhomboid	19
19	Lower and upper stem leaf bases auriculate-sagittate; upper stem leaves linear to lanceolate, usually with lateral 1–2 teeth in upper ⅓, apex acute; silicles 2.5–5.0 × 1.5–2.5 mm, ovate to narrowly rhomboid; petals minute	*Lepidium hyssopifolium Desv.
–	Lower and upper stem leaf bases attenuate; upper stem leaves linear, entire, apex obtuse to subacute; silicles 1.5–3.0 × 1.5–2.3 mm, elliptic; petals absent.	*Lepidium ruderale L.^3^
20	Basal leaves simple, lanceolate to oblanceolate (very rarely with some leaves pinnatifid), margins serrate (sometimes deeply lacerate), occasionally with 1–2 lobes, lobes linear entire.	21
–	Basal leaves pinnatifid or lyrate-pinnatifid, lobes linear, distal margins of lobes serrate or dentate (rarely entire)	23
21	Flowers with (2–)4 stamens; silicles 2.5–3.5 × 1.5–3.3 mm, orbicular to orbicular-rhomboid, minutely notched	*Lepidium panniforme* de Lange et Heenan^4^
–	Flowers with 2 stamens; silicles 2.0–3.5 × 1.8–2.5 mm, oblong, obovate or elliptic	22
22	Stems ridged or grooved when dry, glabrous or sparsely puberulent; silicles 2.0–2.8 × 1.8–2.0 mm, obovate to elliptic	*Lepidium africanum (Burm.f.) DC.
–	Stems smooth when dry, glabrous near base otherwise puberulent; silicles 3.3–3.5 × 2.2–2.5 mm, oblong	*Lepidium divaricatum W.T.Aiton
23	Cauline leaves mostly auriculate-sagittate at base; pedicels adaxially hairy; silicles 2.0–3.0 × 1.5–2.5 mm	24
–	Cauline leaves attenuate at base; pedicels glabrous (very rarely with scattered hairs); silicles 3.5 × 2.1–2.4 mm	*Lepidium pseudohyssopifolium Hewson
24	Mid cauline leaves lanceolate with serrate / serrulate margins; rosette and lower cauline leaves lyrate-pinnatifid with a large broad terminal lobe; silicles 2.0–3.0 × 1.5–2.0 mm, ovate to elliptic	*Lepidium peregrinum Thell.^5^
–	Mid-cauline leaves oblanceolate to cuneate, the margins dentate with acute, ± patent lobes, often with only a single pair below apex; rosette and lower cauline leaves not lyrate-pinnatifid; silicles 2.4–3.0 × 1.8–2.5 mm, ovate to rhomboid	*Lepidium pseudotasmanicum Thell.
25	Young stems finely puberulent; hairs dense to sparse, shortly papillate or tapered	26
–	Young stems glabrous	27
26	Rosette and lower stem leaves pinnatifid; racemes 10–50 mm long, leaf-opposed, obscured by surrounding foliage	*Lepidium flexicaule* Kirk
–	Rosette and lower stem leaves spathulate, oblong or elliptic; racemes 10–90 mm long, not or scarcely leaf-opposed, overtopping surrounding foliage	*Lepidium rekohuense* de Lange et Heenan
27	Leaves narrowly spathulate, cuneate, obdeltoid, obovate (rarely elliptic-lanceolate), entire or sparingly dentate in upper ⅓; teeth (if present) in 1–3(–5) pairs; silicles broadly orbicular and turgid when fresh	*Lepidium oligodontum* de Lange et Heenan^1^
–	Leaves oblanceolate, cuneiform, oblong-obovate, obdeltoid to spathulate, margins finely to deeply incised in upper third to quarter; silicles orbicular, orbicular-ovate to ± rhomboid, cartilaginous (never turgid)	28
28	Plants prostrate; branches numerous, leafy for entire length; leaves finely serrated to crenate, teeth in 10–30 pairs, leaf apices subacute, obtuse to rounded; stamens 2; silicles 2.5–3.5 × 1.5–3.3 mm, elliptic, rhomboid, not winged	*Lepidium limenophylax* de Lange, B.D.Rance et D.A.Norton
–	Plants decumbent to subascendant, widely spreading; branches numerous, leafy only in upper third; leaves finely to deeply incised in upper third to quarter only, teeth in 3–8 pairs, leaf apices praemorse; stamens 2–4; silicles 2.8–3.3 × 2.4–3.0 mm, orbicular, orbicular-ovate to ± rhomboid, winged	*Lepidium oblitum* Houliston, Heenan et de Lange^6^
29	Rosette and/or basal leaves pinnatifid or pinnate	30
–	Rosette and/or basal leavesnot pinnatifid or pinnate	31
30	Plant forming compact, persistent leafy rosettes; flowering stems sparingly leafy, prostrate to ascending; silicles 1.5–2.0 × 1.5–1.8 mm, suborbicular, valves glabrous or sparsely hairy	*Lepidium tenuicaule* Kirk
–	Plant erect, rosettes not compact, often withering at flowering; flower stems erect, leafy; silicles 2.8–4.0 × 2.3–3.2 mm, broadly elliptic, valves glabrous	*Lepidium naufragorum* Garn.-Jones et D.A.Norton^7^
31	Plants decumbent or prostrate	32
–	Plants ascending to erect	38
32	Plants up to 150 mm diameter; stems filiform; leaves 3–6 mm wide, entire, basal leaves dark brown-green (rarely dark green); basal sheath broad, scarious, persistent; silicles 1.5–2.0 × 1.0–1.5 mm	*Lepidium kirkii* Petrie
–	Plants 0.3–1.0 m diameter; stems stout; leaves 5–46 mm wide, serrate, dentate, crenate, lacerate or sometimes entire (*Lepidium oligodontum* only), basal leaves green; basal sheath absent or if present, narrow, not scarious or persistent; silicles 2.1–6.4 × 1.5–5.0 mm	33
33	Plants rhizomatous; stems flexuous; silicles 4.9–6.4 × 4.2–4.9 mm	*Lepidium obtusatum* Kirk
–	Plants not rhizomatous; stems not flexuous; silicles 2.5–4.7 × 1.5–3.9 mm	34
34	Rosette leaves persistent; silicles broadly ovate, ovate to obovate	*Lepidium amissum* de Lange et Heenan
–	Rosette leaves absent, or withering at flowering; silicles broadly orbicular, orbicular, orbicular-rhomboid to elliptic-rhomboid	35
35	Plants upright to spreading or decumbent or sprawling; leaves broadly elliptic, elliptic-oblanceolate, elliptic-obovate, oblanceolate to lanceolate, or obovate-oblong, margins crenate; silicles elliptic-rhomboid to orbicular rhomboid	36
–	Plants decumbent; leaves spathulate, cuneiform, linear-cuneiform, oblanceolate, narrowly ovate, to narrowly obovate (rarely orbicular), entire or margins sparingly, sometimes deeply toothed in upper ⅓; silicles broadly orbicular, orbicular (rarely ovate)	*Lepidium oligodontum* de Lange et Heenan^1^
36	Plants upright to spreading; basal leaf margins deeply toothed and/or lacerate for entire length	*Lepidium panniforme* de Lange et Heenan^5^
–	Plants decumbent to sprawling; basal leaf margins finely to sparingly deeply toothed, if deeply toothed then only in upper third of leaf	37
37	Leaves elliptic, elliptic-oblanceolate, obovate to elliptic-obovate, apex subacute, truncate to obtuse, upper third of leaf margins mostly crenate, or shallowly serrated; stamens 4; silicles 3.1–4.2 × 2.5–3.5 mm, elliptic-rhomboid to orbicular-rhomboid, not winged	*Lepidium juvencum* Heenan et de Lange
–	Leaves oblanceolate, cuneiform, oblong-obovate, obdeltoid to spathulate, apex praemorse margins finely to deeply incised in upper third or quarter, stamens 2–4; silicles 2.8–3.3 × 2.4–3.0 mm, orbicular, orbicular-ovate to ± rhomboid, margin winged	*Lepidium oblitum* Houliston, Heenan et de Lange^6^
38	Pedicels hairy	39
–	Pedicels glabrous	40
39	Inflorescence rachises glabrous (sometimes with sparse, pale, clavate hairs); pedicels hairy on adaxial surfaces only; petals 1.8–2.0 × 0.1–0.9 mm, elliptic or filiform (rarely obovate); plant of northern South Island	*Lepidium banksii* Kirk
–	Inflorescence rachises densely covered in white clavate hairs; pedicels densely and mostly circumferentially covered in white clavate hairs; petals 1.3–2.0 × 1.0–2.3 mm, obovate-spathulate (rarely orbicular); plant of Bounty Islands	*Lepidium seditiosum* de Lange, Heenan et J.Rolfe^8^
40	Plants with slender, upright stems; leaves thin, not fleshy, lanceolate, elliptic-lanceolate (rarely narrow elliptic), margin deeply serrate; silicles 4.0–4.7 × 3.2–3.5 mm, elliptic-rhomboid to orbicular-rhomboid, apex shallowly notched	*Lepidium aegrum* Heenan et de Lange
–	Plants with stout, upright to spreading short, rigid, stems; leaves thick and fleshy, broadly elliptic, elliptic-obovate to obovate-oblong, margin crenate; silicles 3.0–3.7 × 2.6–3.1 mm, orbicular to orbicular-rhomboid, apex obtuse to shallowly notched	*Lepidium crassum* Heenan et de Lange

^1^ *Lepidium oligodontum* plants may have 2, 4 or 6 stamens, though 4 is the usual number. On occasion plants with mostly 2 stamens are known. For this reason this species may key out in three areas depending on the number of stamens present.

^2^ We follow de Lange et al. (2009) and de Lange and Rolfe (2010) in treating *Lepidium desvauxii* as indigenous within the southern part of its New Zealand range (including the Chatham Islands), and naturalised within the more modified parts of central and northern New Zealand.

^3^ *Lepidium ruderale* is accepted by Webb et al. (1995) as naturalised in New Zealand.

^4^ *Lepidium panniforme* usually has 4 stamens but occasional plants with 2 or 2–4 stamens occur, and for this reason this species may key out in two areas depending on the number of stamens present.

^5^ *Lepidium peregrinum* is regarded here as naturalised to New Zealand (see Heenan and de Lange 2011). It is as an uncommon naturalised plant of glauconite and glauconitic limestone rock stacks and islands along the southern shore line of the Kawhia Harbour. *Lepidium peregrinum* is a threatened species in Australia (Scarlett 1999).

^6^ *Lepidium oblitum* may have 2–4 stamens. For this reason this species may key out in two areas depending on the number of stamens present.

^7^ Occasional plants of *Lepidium naufragorum* with simple or weakly pinnatifid leaves are also known, and these could be confused with *Lepidium oleraceum* from which they differ by the seasonal growth habit (with plants dying back to the rootstock over winter), and by the distinctly emarginate silicles

^8^ *Lepidium seditiosum* is known only from a single, imperfect gathering which lacks mature silicles. However, the shape of the ovary apex of immature silicles indicates that, in this species, they are notched.

## Supplementary Material

XML Treatment for Lepidium aegrum

XML Treatment for Lepidium amissum

XML Treatment for Lepidium banksii

XML Treatment for Lepidium castellanum

XML Treatment for Lepidium crassum

XML Treatment for Lepidium flexicaule

XML Treatment for Lepidium juvencum

XML Treatment for Lepidium limenophylax

XML Treatment for Lepidium naufragorum

XML Treatment for Lepidium oblitum

XML Treatment for Lepidium obtusatum

XML Treatment for Lepidium oleraceum

XML Treatment for Lepidium oligodontum

XML Treatment for Lepidium panniforme

XML Treatment for Lepidium rekohuense

XML Treatment for Lepidium seditiosum
